# Antiglioma Natural Products from the Marine-Associated Fungus *Penicillium* sp. ZZ1750

**DOI:** 10.3390/molecules27207099

**Published:** 2022-10-20

**Authors:** Kuo Yong, Sidra Kaleem, Mingzhu Ma, Xiaoyuan Lian, Zhizhen Zhang

**Affiliations:** 1 Zhoushan Campus, Ocean College, Zhejiang University, Zhoushan 316021, China; 2 Zhejiang Marine Development Research Institute, Zhoushan 316000, China; 3 College of Pharmaceutical Sciences, Zhejiang University, Hangzhou 310058, China

**Keywords:** marine *Penicillium* sp. ZZ1750, penipyridinone B, 11*S*-(−)-penilloid A, 11*R*,14*E*-(+)-penilloid A, structure elucidation, antiglioma activity

## Abstract

Marine-derived *Penicillium* fungi are one of the most important sources for the discovery of new bioactive natural products. This study characterized the isolation, structures, and antiglioma activities of twelve compounds, including three novel ones—penipyridinone B (**1**), 11*S*-(−)-penilloid A (**2**), and 11*R*,14*E*-(+)-penilloid A (**3**)—from the marine fungus *Penicillium* sp. ZZ1750. The structures of the novel compounds were determined via extensive nuclear magnetic resonance (NMR) spectroscopic analyses, high-resolution electrospray ionization mass spectroscopy (HRESIMS) data, Mosher’s method, optical rotation (OR) calculations, and electronic circular dichroism (ECD) calculations. Penipyridinone B represents the first example of its structural type and showed potent antiglioma activity, with IC_50_ values of 2.45 μM for U87MG cells and 11.40 μM for U251 cells. The known compounds of questiomycin A (**9**) and xanthocillin X (**10**) also showed antiproliferative activity against both U87MG and U251 cells, with IC_50_ values of 13.65 μM to 22.56 μM. The antiglioma activity of questiomycin A and xanthocillin X may be related to the promotion of reactive oxygen species (ROS) production, the reduction of mitochondrial membrane potential (MMP), and the enhancement of caspase-3 enzyme activity.

## 1. Introduction

Marine-associated fungi from the genus *Penicillium* have been proved to be one of the most important resources for the discovery of novel bioactive natural products [1,2,3]. From 1991 to 2014, 390 new compounds with diverse chemical structures were identified from the marine-derived *Penicillium* fungi, of which 58% showed antitumor, antiviral, antibacterial, and anti-inflammatory activities [1]. An updated catalog of this field from 2015 to 2020 identified 188 secondary metabolites with diverse bioactivities [3]. Recently, more and more novel compounds continued to be discovered from the marine-derived *Penicillium* fungi, such as poloncosidins A-F from the sea cold-seep-derived fungus *Penicillium polonicum* CS-252 [4], penitanzacids A-J from the deep-sea-derived fungus *Penicillium* sp. KWF32 [5], steckwaic acids A-H from the deep-sea coral-derived endozoic *Penicillium steckii* AS-324 [6,7], dicitrinones G-J from the starfish-derived symbiotic fungus *Penicillium* sp. GGF16-1-2 [8], oxalierpenes A and B from the marine *Penicillium oxalicum* HBU-208 [9], and pyrrospirones K-Q from the marine-derived fungus *Penicillium* sp. SCSIO 41512 [10].

**Figure 1:**
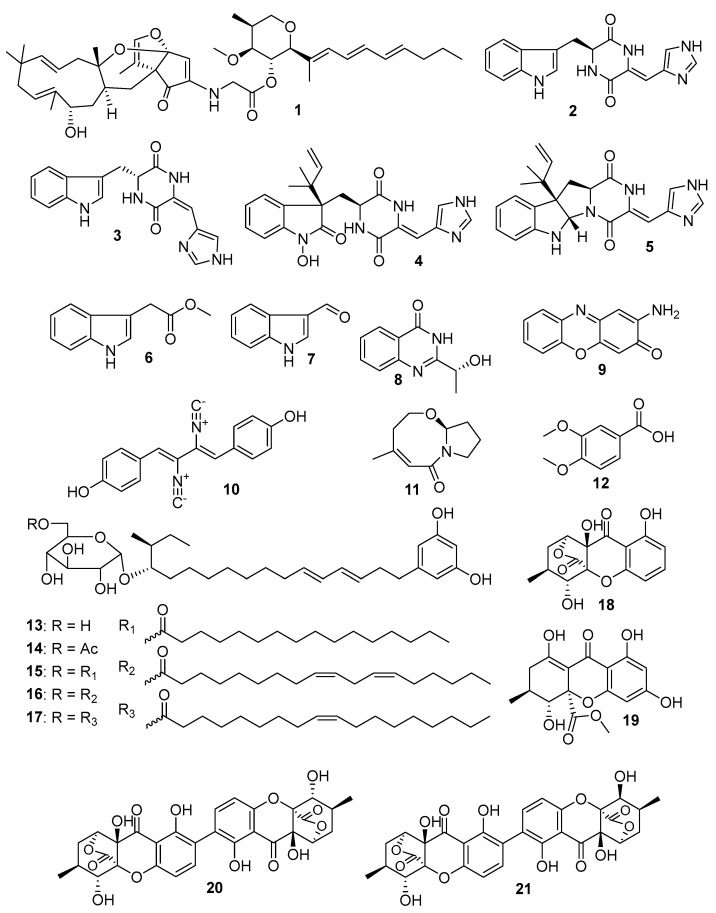
Chemical structures of compounds **1**–**21** from the marine fungus *Penicillium* sp. ZZ1750.

During the course of our ongoing research program for the discovery of novel antiglioma natural products from marine-sourced microorganisms [11,12,13,14,15,16,17,18], an identified fungus strain, *Penicillium* sp. ZZ1750 (Table S1), was isolated from a marine mud sample collected from the Arabian Sea close to Karachi, Sindh, Pakistan. This fungus in rice medium produced twelve novel compounds, including peniresorcinosides A–E (**13**–**17**), penidifarnesylin A, penipyridinone A [17], ergochromes D–G (**18**–**21**) [19], and penipyridinone B (**1**, Figure 1), an unidentified compound with a complicated structure. Interestingly, peniresorcinosides A–E were five rare glycosylated alkylresorcinols produced by the strain ZZ1750 in rice medium for a 30 day culture, while the four polyhydroxanthones of ergochromes D–G were the metabolites of this strain in rice medium for a 90 day culture. In order to deeply explore novel compounds from this fungal strain, we further investigated its secondary metabolites cultured in glycerol arginine (GA) liquid medium, resulting in the isolation of eleven compounds (**2**–**12**, Figure 1), including two new indole alkaloids: 11*S*-(−)-penilloid A (**2**) and 11*R*,14*E*-(+)-penilloid A (**3**). Herein, we report on the isolation and structure of the new compounds **1**–**3**, as well as on the antiproliferative activities of all of the isolated compounds against human glioma cells.

## 2. Results and Discussion

### 2.1. Structure Elucidation of the Isolated Compounds

Compound **1** was isolated from a 30 day culture of the strain ZZ1750 in rice medium and compounds **2**–**12** were isolated from a culture of the strain ZZ1750 in GA liquid medium. Based on nuclear magnetic resonance (NMR) spectroscopic analyses and a comparison with the reported data, nine known compounds were identified: isopenilline A (**4**) [20], isoroquefortine C (**5**) [21], indole-3-methylethanoate (**6**) [22], 1*H*-indole-3-carbaldehyde (**7**) [23], *R*(+)-chrysogenin (**8**) [24], questiomycin A (**9**) [20,25], xanthocillin X (**10**) [26], cladosporilactam A (**11**) [27], and veratric acid (**12**) [28]. Their ^13^C and ^1^H NMR data are summarized in Tables S2–S5.

**Figure 2:**
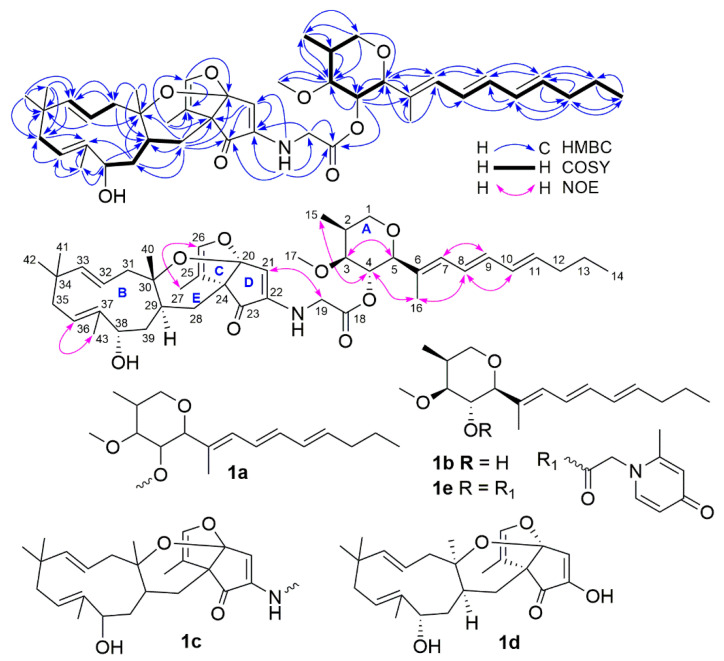
COSY, key HMBC, and NOE correlations of penipyridinone B (**1**) and structures of **1a**–**1e**.

Compound **1** was obtained as white amorphous powder and its high-resolution electrospray ionization mass spectroscopy (HRESIMS) spectrum showed ion peaks at *m*/*z* 720.4473 [M + H] and 742.4292 [M + Na], corresponding to a molecular formula C_43_H_61_NO_8_. Analysis of its ^1^H, ^13^C, distortionless enhancement by polarization transfer (DEPT), and heteronuclear multiple quantum coherence (HMQC) NMR spectra indicated the presence of two carbonyls, seven pairs of double bonds, four oxymethines, one oxymethylene, one methoxy, four nonprotonated carbons, two methines, seven methylenes, and eight methyls. The two carbonyls and seven pairs of double bonds accounted for nine of the fourteen degrees of unsaturation required by the molecular formula; the remaining five suggested that **1** had a structure with five cycles. As shown in Figure 2, two spin systems of H_2_-1/H-2(H_3_-15)/H-3/H-4/H-5 and H-7/H-8/H-9/H-10/H-11/H_2_-12/H_2_-13/H_3_-14 were observed in the ^1^H-^1^H correlation spectroscopy (COSY) spectrum and the heteronuclear multiple bond coherence (HMBC) spectrum (in MeOH-*d*_4_) showed key correlations of H_2_-1 (*δ*_H_ 3.76, 3.61) with C-3 (*δ*_C_ 82.9), C-5 (*δ*_C_ 86.9) and C-15 (*δ*_C_ 11.2); H-3 (*δ*_H_ 3.45) with C-4 (*δ*_C_ 71.0), C-15 and C-17 (*δ*_C_ 56.9); H-4 (*δ*_H_ 5.00) with C-3, C-5 and C-6 (*δ*_C_ 134.0); H-5 (*δ*_H_ 3.57) with C-4, C-6 and C-7 (*δ*_C_ 131.6); H-7 (*δ*_H_ 6.06) with C-5, C-8 (*δ*_C_ 127.3) and C-9 (*δ*_C_ 136.0); H-8 (*δ*_H_ 6.29) with C-10 (*δ*_C_ 132.5); H-9 (*δ*_H_ 6.27) with C-7 and C-11 (*δ*_C_ 136.6); H-10 (*δ*_H_ 6.12) with C-9 and C-12 (*δ*_C_ 36.2); H-11 (*δ*_H_ 5.72) with C-9, C-12 and C-13 (*δ*_C_ 23.7); H_2_-12 (*δ*_H_ 2.06) with C-10, C-11 and C-14 (*δ*_C_ 14.3); H_2_-13 (*δ*_H_ 1.41) with C-11 and C-14; H_3_-14 (*δ*_H_ 0.91) with C-12 and C-13; H_3_-15 (*δ*_H_ 1.07) with C-1 (*δ*_C_ 72.0), C-2 (*δ*_C_ 33.9) and C-3; H_3_-16 (*δ*_H_ 1.72) with C-5, C-6 and C-7; and H_3_-17 (*δ*_H_ 3.34) with C-3. These COSY and HMBC correlations demonstrated that the planar structure of a partial structure (**1a**) in **1** was the same as that of restrictinol (**1b**), a tetrahydropyran derivative [29,30].

In the same way, the planar structure of a partial structure (**1c**) in **1** was also constructed based on the COSY and HMBC correlations (Figure 2). In the COSY spectrum, three spin systems of H-28/H-29/H-39/38, H-31/H-32/H-33, and H-35/H-36 were observed. The HMBC spectrum showed the following key correlations: NH-19 (*δ*_H_ 5.70) with C-21 (*δ*_C_ 114.7), C-22 (*δ*_C_ 145.3) and C-23 (*δ*_C_ 199.3) (Table 1, in DMSO-*d*_6_); H-21 (*δ*_H_ 5.54) with C-22 (*δ*_C_ 147.1), C-23 (*δ*_C_ 201.4) and C-24 (*δ*_C_ 58.8); H-26 (*δ*_H_ 6.15) with C-20 (*δ*_C_ 114.3) and C-25 (*δ*_C_ 111.6); H_3_-27 (*δ*_H_ 1.62) with C-24, C-25 and C-26 (*δ*_C_ 141.3); H_2_-28 (*δ*_H_ 2.10, 1.18) with C-20, C-23, 24, C-25, C-29 (*δ*_C_ 32.6), C-30 (*δ*_C_ 82.1) and C-39 (*δ*_C_ 39.7); H_2_-31 (*δ*_H_ 2.54, 2.34) with C-29 and C-33 (*δ*_C_ 145.3); H-32 (*δ*_H_ 5.30) with C-30 and C-34 (*δ*_C_ 37.4); H-33 (*δ*_H_ 5.26) with C-31 (*δ*_C_ 50.4) and C-35 (*δ*_C_ 43.1); H_2_-35 (*δ*_H_ 2.19, 1.76) with C-34 and C-37 (*δ*_C_ 139.0); H-36 (*δ*_H_ 5.42) with C-35 and C-43 (*δ*_C_ 20.2), H-38 (*δ*_H_ 4.55) with C-29 and C-43; H_2_-39 (*δ*_H_ 1.89, 1.42) with C-28 (*δ*_C_ 29.6), C-30 and C-37; H_3_-40 (*δ*_H_ 1.15) with C-29, C-30 and C-31; H_3_-41 (*δ*_H_ 1.10) with C-33, C-34 and C-35; H_3_-42 (*δ*_H_ 1.05) with C-33, C-34, C-35 and C-41 (*δ*_C_ 29.0); and H_3_-43 (*δ*_H_ 1.74) with C-36 (*δ*_C_ 126.1), C-37 and C-38 (*δ*_C_ 69.0). It was found that the planar structure of **1c** was almost the same as that of neosetophomone (**1d**), a unique meroterpenoid from *Neosetophoma* sp. [31]; their only difference was that the -OH group in **1d** was replaced by an -NN group in **1c**.

Finally, HMBC correlations of H-4 with C-18 (*δ*_C_ 171.2); H_2_-19 (*δ*_H_ 3.75, 3.65) with C-18 and C-22; and NH-19 with C-18 (*δ*_C_ 168.9) and C-19 (*δ*_C_ 44.8) (Table 1, in DMSO-*d*_6_) established the linkage between **1a** and **1c** through a -CH_2_-C(O)- group. Therefore, the whole planar structure of **1** has been determined.

**Figure 3:**
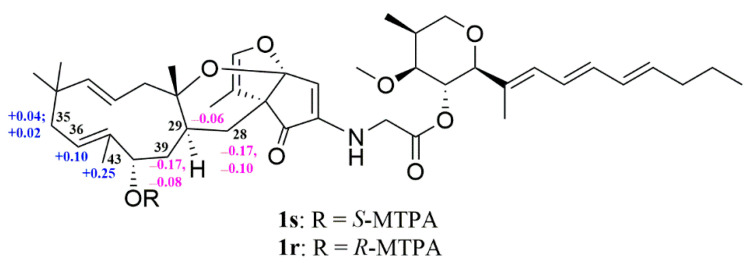
The Δ*δ_S_*_-*R*_ values of the ^1^H NMR chemical shifts of the MTPA esters (**1s** and **1r**) of penipyridinone B (**1**).

**Figure 4:**
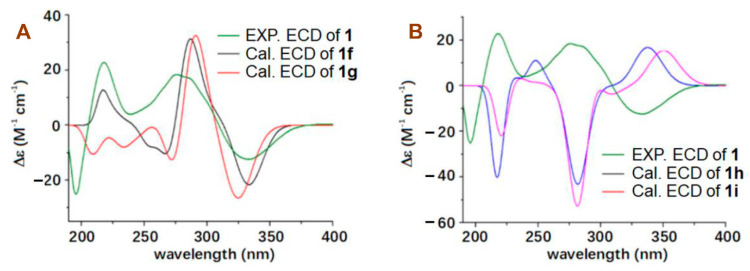
The experimental ECD spectrum of penipyridinone B (**1**) in MeOH and the calculated ECD curves of four model molecules **1f**, *1g* (**A**), **1h** and **1i** (**B**) at the b3lyp/6-311+g (d, p) level in MeOH.

The configuration of **1** was assigned by a combination of the coupling constant of proton, nuclear Overhauser effect (NOE) information, Mosher’s method, and electronic circular dichroism (ECD) calculations. A small coupling constant of 5.5 Hz (^3^*J*_H2/__H3_) and large coupling constants of 9.5 Hz (^3^*J*_H3/H4_ and ^3^*J*_H4/H5_) (Table 1) suggested a *β*-orientation for H-4 and an *α*-orientation for H-2, H-3 and H-5 [32], which were supported by NOE correlations (Figure 2) of H-3 with H-5, as well as H-4 with H_3_-15 and H_3_-16. The *trans*-coupling constants of 15.0 Hz for ^3^*J*_H8/H9_, 15.1 Hz for ^3^*J*_H10/H11_ and 15.8 Hz for ^3^*J*_H32H33_ indicated the 8*E*, 10*E* and 32*E* geometries, respectively; the 6*E*, 21*E*, 25*Z*, and 35*Z* geometries were determined based on the NOE correlations of H-7 with H-9; H-8 with H-10 and H_3_-16; H_2_-19 with H-21; H-26 with H_3_-27; and H-36 with H_3_-43, respectively. The absolute configuration of C-38 in **1** was established by the Mosher ester NMR method. Treatment of **1** with (*R*)-α-methoxy-α-(trifluoromethyl) phenylacetyl chloride (*R*-MTPA-Cl) or *S*-MTPA-Cl yielded its *S*-MTPA ester (**1s**) or *R*-MTPA ester (**1r**). The ^1^H NMR chemical shift differences (Δ*δ_S_*_-*R*_, Figure 3 and Table S6) between **1s** and **1r** in negative values for H-28, H-29, and H-39 and positive values for H-35, H-36, and H-43 were observed, indicating a 38*S*-configuration in **1**. Finally, ECD calculations were used to determine the configurations of C-20, C-24, C-29, and C-30. Due to the relationship between the fused positions of the rings C, D, and E in the structure, C-20 and C-24 had only two possible configurations: 20*S*,24*S* and 20*R*,24*R*. In addition, the absence of NOE correlation of H-29 with H_3_-40 observed in the NOESY spectrum suggested only two possible configurations: 29*S*,30*S* and 29*R*,30*R*. Therefore, four model molecules of 2*S*,3*S*,4*R*,5*S*,20*S*,24*S*,29*S*,30*S*,38*S*-**1** (**1f**), 2*S*,3*S*,4*R*,5*S*,20*S*,24*S*,29*R*,30*R*,38*S*-**1** (**1g**), 2*S*,3*S*,4*R*,5*S*,20*R*,24*R*,29*S*,30*S*,38*S*-**1** (**1h**), and 2*S*, 3*S*,4*R*,5*S*,20*R*,24*R*,29*R*,30*R*,38*S*-**1** (**1i**) were applied for ECD calculations. The ECD calculated results (Figure 4 and Tables S7–S14) showed that the experimental ECD spectrum of **1** was in agreement with the calculated ECD curve of the model molecule of 2*S*,3*S*,4*R*,5*S*, 20*S*,24*S*,29*S*,30*S*,38*S*-**1** (**1f**). Therefore, the signed configurations for the partial structure (**1a**) were the same as those of restrictinol (**1b**) [29,30] and penipyridinone A (**1e**), a reported new compound previously isolated from the same fungal strain *Penicillium* sp. ZZ1750 [17], while the configurations of the partial structure (**1c**) were the same as those of the known meroterpenoid neosetophomone (**1d**) [31]. Based on the foregoing evidence, the structure of **1** was elucidated as a novel compound, named penipyridinone B. The ^13^C and ^1^H NMR data (Table 1) of penipyridinone B (**1**) were assigned by a combination of ^1^H, ^13^C, DEPT, HMQC, HMBC, and NOESY NMR spectroscopic analyses.

**Figure 5:**
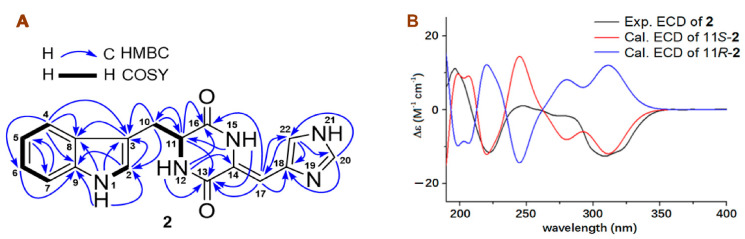
(**A**) COSY, key HMBC, and NOE correlations of 11*S*-(−)-penilloid A (**2**) and the experimental ECD spectrum of 11*S*-(−)-penilloid A (**2**) in MeOH; (**B**) The calculated ECD curves of two model molecules (11*S*-**2** and 11*R*-**2**) at the b3lyp/6-311+g (d, p) level in MeOH.

Compound **2** was obtained as a yellow amorphous solid and had a molecular formula C_17_H_15_N_5_O_2_ deduced from its HRESIMS ion peaks at *m*/*z* 322.1298 [M + H] and 344.1118 [M + Na], as well as from ^13^C NMR data. Careful interpretation of its ^13^C and ^1^H NMR data (Table 2) as well as the COSY and HMBC correlations (Figure 5) demonstrated that **2** had the same planar structure as that of a known indole alkaloid penilloid A [33]. However, the configuration at C-11 of penilloid A was not determined, although its positive optical rotation (OR) value (+218.2, *c* 0.06, MeOH) was reported, which was opposite to the negative OR value (−201.6, *c* 0.16, MeOH) of **2**. Therefore, OR calculations [34,35] were used to assign the configuration at C-11, the only chiral carbon in **2**. The OR calculated results (Tables S15–S18) showed a positive OR value (+697.42) for 11*R* and a negative OR value (−697.60) for 11*S.* Accordingly, a 11*S*-configuration was assigned for **2** because of its negative OR value, and penilloid A should have a 11*R*-configuration. The 11*S*-configuration for **2** was further confirmed by ECD calculated results (Figure 5 and Tables S19–S22), because the experimental ECD spectrum of **2** showed good agreement with the calculated ECD curve of the model molecule of 11*S*-**2**. The geometry of the C_14_-C_17_ can be assigned based on the chemical shift difference (Δ*δ*_C14-C18_) between C-14 and C-18 [20,33]. Usually, an Δ*δ*_C14-C18_ value of over 10 ppm indicated a 14*Z*-geometry; while an Δ*δ*_C14-C18_ value of less 5 ppm was suggestive of an *E*-geometry. As shown in Table 2, the Δ*δ*_C14-C18_ values in MeOH-*d*_4_ and DMSO-*d*_6_ were 13.0 and 11.6 ppm, respectively, indicating a 14*Z*-geometry for **2**. Based on the foregoing evidence, the structure of **2** was elucidated as a new indole diketone piperazine alkaloid, named 11*S*-(−)-penilloid A.

**Figure 6:**
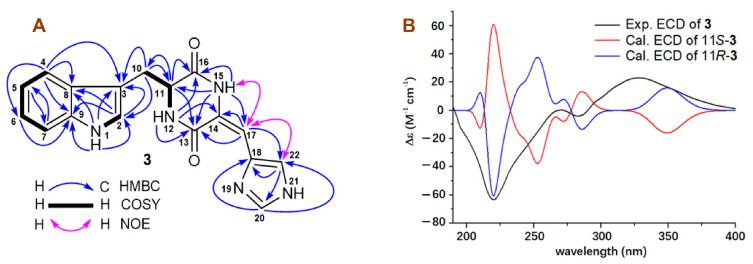
(**A**) COSY, key HMBC, and NOE correlations of 11*R*,14*E*-(+)-penilloid A (**3**); (**B**) The experimental ECD spectrum of 11*R*,14*E*-(+)-penilloid A (**3**) in MeOH and the calculated ECD curves of two model molecules (11*S*-**3** and 11*R*-**3**) at the b3lyp/6-311+g (d, p) level in MeOH.

Compound **3** had the same molecular formula, C_17_H_15_N_5_O_2_, and very similar UV absorptions as those of **2**. Further detailed analyses of the ^13^C and ^1^H NMR data (Table 3), as well as the HMQC, COSY, and HMBC correlations (Figure 6) of **3**, led to the conclusion that both **3** and **2** were isomers of indole diketone piperazine alkaloids. As described for **2**, the chemical shift difference (Δ*δ*_C14-C18_) between C-14 and C-18 can be used to assign the geometry of the C_14_-C_17_ double bond in **3** [20,33]. Therefore, the Δ*δ*_C14-C18_ values of 0.4 ppm in MeOH-*d*_4_ and 1.3 ppm in DMSO-*d*_6_ indicated a 14*E*-geometry in **3**. The NOE correlations (Figure 6) of H-17 (*δ*_H_ 5.68) with NH-15 (*δ*_H_ 10.47) and H-22 (*δ*_H_ 7.71) (Table 3, in DMSO-*d*_6_) also supported the 14*E*-geometry for **3**. Compared with the negative OR value (−201.6) of **2**, compound **3** had a positive OR value (+203.8), suggesting an 11*R*-configuration for **3**, which was the same as that of penilloid A. The 11*R*-configuration for **3** was further confirmed by the OR calculated results (Tables S23–S26) and the ECD calculated results (Figure 6 and Tables S27–S30). Therefore, structure of **3** was elucidated as a new indole diketone piperazine alkaloid, named 11*R*,14*E*-(+)-penilloid A.

### 2.2. Antiglioma Activity Evaluation

A sulforhodamine B (SRB) assay [36] was applied to evaluate the activity of all of the isolated compounds (**1**–**12**) against the proliferation of glioma cells. Doxorubicin (DOX) was used as a positive control. The results (Table 4) indicated that the new penipyridinone B (**1**) had potent antiproliferative activity, with IC_50_ values of 2.45 μM for U87MG cells and 11.40 μM for U251 cells, and the known compounds of questiomycin A (**9**) and xanthocillin X (**10**) also showed moderate antiproliferative activity against both U87MG and U251 cells, with IC_50_ values of 13.65 μM to 22.56 μM, compared with the control drug DOX, with IC_50_ values of 3.76 μM for U87MG cells and 9.85 μM for U251 cells. Other tested compounds were inactive at a concentration of 50 μM.

**Figure 7:**
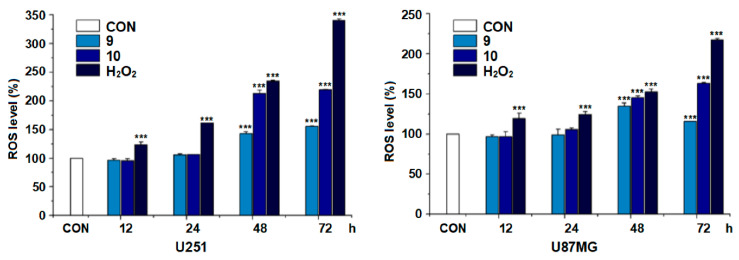
Effect of questiomycin A (**9**) and xanthocillin X (**10**) on the ROS production in glioma U251 and U87MG cells. Glioma U251 and U87MG cells were treated with **9** (20 μM), **10** (20 μM), and H_2_O_2_ (6 μM) at different time points. Data are presented as the mean ± SD, n = 4; *** *p* < 0.001 (vs. CON).

**Figure 8:**
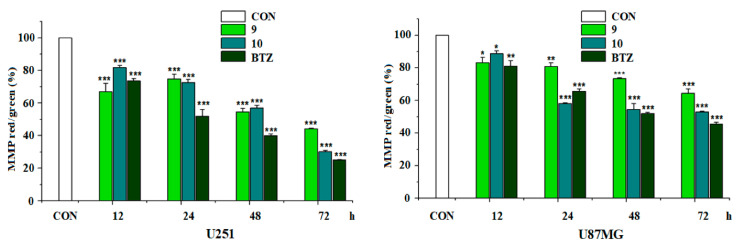
Effect of questiomycin A (**9**) and xanthocillin X (**10**) on the MMP in glioma U251 and U87MG cells. Glioma U251 and U87MG cells were treated with **9** (20 μM), **10** (20 μM), and BTZ (16 μM) at different time points. Data are presented as the mean ± SD, n = 4; * *p* < 0.05, ** *p* < 0.01 or *** *p* < 0.001 (vs. CON).

**Figure 9:**
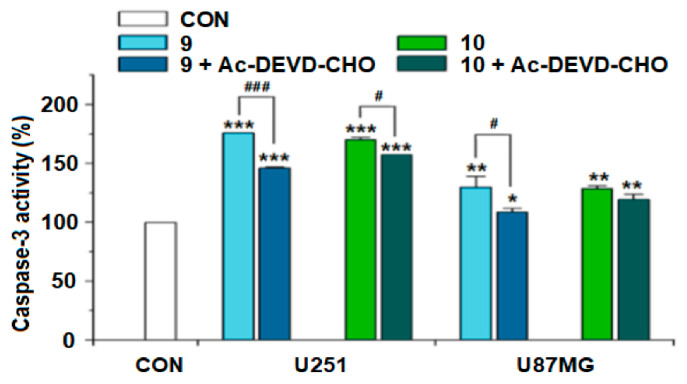
Effect of questiomycin A (**9**) and xanthocillin X (**10**) on the caspase-3 activity in glioma U251 and U87MG cells. Glioma U251 and U87MG cells were treated with **9** (20 μM), **10** (20 μM), **9** (20 μM) + Ac-DEVD-CHO (10 μM), and **10** (20 μM) + Ac-DEVD-CHO (10 μM) for 24 h. Data are presented as the mean ± SD, n = 4; * *p* < 0.05, ** *p* < 0.01 or *** *p* < 0.001 (vs. CON); ^#^ *p* < 0.05 or ^###^ *p* < 0.001 (**9** + Ac-DEVD-CHO or **10** + Ac-DEVD-CHO vs. **9** or **10**).

The effects of questiomycin A (**9**) and xanthocillin X (**10**) on the reactive oxygen species (ROS) production, the mitochondrial membrane potential (MMP), and the caspase-3 activity in glioma U251 and U87MG cells were further investigated. The results indicated that both questiomycin A (**9**, 20 μM) and xanthocillin X (**10**, 20 μM) significantly increased the ROS production (*p* < 0.001, Figure 7) and reduced the MMP (*p* < 0.001, Figure 8) in both U251 and U87MG cells after treatment for 48 h, when compared with the blank control (CON). Questiomycin A (**9**) and xanthocillin X (**10**) also significantly increased the caspase-3 activity in glioma U251 and U87MG cells (** *p* < 0.01 or *** *p* < 0.001, Figure 9) after treatment for 24 h. The enhancement of caspase-3 activity induced by questiomycin A (**9**) in both U251 and U87MG cells was significantly reduced by the caspase-3 inhibitor Ac-DEVD-CHO (^#^ *p* < 0.05 or ^###^ *p* < 0.001, Figure 9). However, Ac-DEVD-CHO only significantly reduced the enhancement of caspase-3 activity induced by xanthocillin X (**10**) in U251 cells (^#^ *p* < 0.05).

ROS is known as one of the main upstream effectors in the regulation of apoptosis and excess cellular levels of ROS cause damage to cellular components, which can result in cell death [37]. Mitochondria plays an important role in tumorigenesis and apoptosis. Mitochondrial damage, characterized by the loss of MMP, can cause cells to enter an irreversible process of apoptosis [38]. Caspase-3 is a cysteine-aspartic acid protease and has been identified as an important mediator of apoptosis in cancer; its deficiency may disturb the apoptosis, resulting in carcinogenesis. Thereupon, the enhancement of caspase-3 activity can induce apoptosis of cancer cells, with clinical significance [39]. The current study demonstrated that questiomycin A (**9**) and xanthocillin X (**10**) significantly enhanced the ROS production and the caspase-3 activity and decreased the MMP in glioma U251 and U87MG cells. All data, taken together, indicated that the antiglioma activities of both questiomycin A (**9**) and xanthocillin X (**10**) were related to the promotion of ROS production, the reduction of MMP, and the enhancement of caspase-3 activity.

## 3. Experimental Section

### 3.1. General Procedures

Ultraviolet-visible (UV), infrared (IR), OR, and ECD spectra were recorded on a METASH UV-8000 spectrometer (Shanghai METASH Instruments Co. Ltd., Shanghai, China), a Nicolet^TM^ IS^TM^ 10 FT-IR spectrometer (Thermo Fisher Scientific, Waltham, MA, USA), an Autopol I polarimeter (Rudolph Research Analytical, Hackettstown, NJ, USA) and a JASCO J-815 spectropolarimeter (JASCO Co., Tokyo, Japan), respectively. NMR spectra were acquired on a JEOL 600 spectrometer (JEOL Co. Ltd., Tokyo, Japan) using standard programs and acquisition parameters and chemical shift values were expressed in *δ* (ppm) relative to DMSO-*d*_6_ (*δ*_C_ 39.5, *δ*_H_ 2.50) or MeOH-*d*_4_ (*δ*_C_ 49.15, *δ*_H_ 3.31). HRESIMS data were obtained on an Agilent 6230 TOF LC/MS spectrometer (Agilent Technologies Co. Ltd., Santa Clara, CA, USA). Diaion HP-20 (Mitsubishi Chemical Group, Tokyo, Japan), silica gel (100–200 mesh, Qingdao Marine Chemical Co. Ltd., Qingdao, China) and octadecyl-functionalized silica gel (ODS, Cosmosil 75C_18_-Prep, Nacalai Tesque Inc., Kyoto, Japan) were used for column chromatography. HPLC separation was performed on a CXTH LC-3000 preparative HPLC system (Beijing Chuangxin Tongheng Science & Technology Co. Ltd., Beijing, China) with a CT-30 Fuji-C_18_ column (280 × 30 mm, 10 µm) and an Agilent 1260 infinity HPLC system using a Zorbax SB-C_18_ column (250 × 9.4 mm, 5 µm) and a DAD detector. All solvents used for this study were purchased from the Sinopharm Chemical Reagent Co. Ltd. (Shanghai, China) and Mosher reagents of (*R*)-α-methoxy-α-(trifluoromethyl)-phenylacetyl chloride (*R*-MTPA-Cl) and *S*-MTPA-Cl were bought from Shanghai Aladdin Biochemical Technology Co., Ltd. (Shanghai, China). Human glioma U87MG (JDS-2568) and U251 (XB-0439) cells were purchased from the Cell Bank of the Chinese Academy of Sciences (Shanghai, China). Doxorubicin (DOX) and bortezomib (BTZ) were purchased from Solarbio Science & Technology Co. Ltd. (Beijing, China). Ac-DEVD-CHO were ordered from Shanghai Aladdin Biochemical Technology Co., Ltd. (Shanghai, China). Artificial seawater (sea salt 35.0 g, tap water 1.0 L), rice medium (rice 40.0 g, 60 mL artificial seawater), and glycerol arginine (GA) liquid medium (glycerol 6.0 mL, arginine 1.0 g, K_2_HPO_4_ 1.0 g, MgSO_4_·7H_2_O 1.0 g, tap water 1.0 L) were made in the authors’ laboratory.

### 3.2. Isolation and Taxonomic Identity of Penicillium sp. ZZ1750

The marine-derived fungus *Penicillium* sp. ZZ1750 was isolated from a sample of marine mud, which was collected from the Arabian Sea close to Karachi, Sindh, Pakistan, in January 2019. The detailed isolation and taxonomic identity (Table S1 and Figure S1) of *Penicillium* sp. ZZ1750 were reported in a previous publication [17].

### 3.3. Large Culture of Strain ZZ1750

The large culture of the strain ZZ1750 in rice medium for 30 days was described in a previous publication [17]. A scale-up culture of the strain ZZ1750 was also conducted in GA liquid medium (Figure S2). Briefly, the pure colony of strain ZZ1750 from the PDA medium slant was inoculated into 500 mL Erlenmeyer flasks, each containing 200 mL of GA liquid medium, then incubated at 28 °C for 4 days on a rotary shaker (180 rpm) to produce seed broth. The seed broth (5 mL) was transferred into 200 mL GA liquid medium in a 500 mL Erlenmeyer flask, then incubated in static state at room temperature for 30 days. A total of 60 L culture (300 flasks) was prepared for this study.

### 3.4. Extraction and Isolation of Compounds ***1***–***12***

Compound **1** was isolated from the culture [17] of the strain ZZ1750 in rice medium. Briefly, the culture of the strain ZZ1750 in rice medium in each flask was extracted with EtOAc (250 mL) three times. The combined EtOAc extract was dried in vacuo to give an extract (100 g). This extract was fractionated on a column of silica gel (1600 g) eluting with a mixture of cyclohexane and EtOAc in different ratios (10:1, 5:1, 2:1, 1:1, and 1:2, each 1000 mL) to give four fractions of Frs.A–D based on the results of TLC analyses. Then, Fr. B was separated on a column of ODS (200 g) successively eluting with 60%, 70%, 80%, and 90% MeOH (each 1000 mL) to yield four subfractions of SFrs.B_1_–B_4_. SFr.B_4_ was further separated by HPLC using a Zorbax SB-C_18_ column (250 × 9.4 mm, 5 µm; mobile phase: MeOH/H_2_O, 87/13; flow rate: 1.0 mL/min; UV detection: 275 nm) to yield compounds **1e** (3.8 mg, t_R_ 41.0 min) and **1** (2.8 mg, t_R_ 48.0 min).

Compounds **2**–**12** were isolated from the culture of the strain ZZ1750 in GA liquid medium. The 60 L culture of the strain ZZ1750 in GA liquid medium was centrifuged to give filtrate and mycelia. The mycelia were extracted with EtOAc three times to yield an EtOAc extract after removing the organic solvent. The filtrate was applied to a HP-20 column eluting with water, then 100% MeOH. The collected MeOH fraction was dried in vacuo to obtain a MeOH extract. The EtOAc extract and MeOH extract were combined (13.2 g), then fractionated on a column of silica gel (130 g) successively eluting with a mixture of petroleum ether and EtOAc in different ratios (10:1, 8:1, 5:1, 3:1, 2:1, and 1:1, each 1000 mL) to give six fractions of Frs.A–F.

Fr.B was separated by a column of ODS (200 g) eluting with 50% and then 70% MeOH (each 400 mL) to produce three subfractions of SFr.B_1_ from 400 mL 50% MeOH elution, SFr.B_2_ from the first 200 mL 70% MeOH elution, and SFr.B_3_ from the second 200 mL 70% MeOH elution. SFr.B_1_, SFr.B_2_, and SFr.B_3_ were purified by HPLC using the Zorbax SB-C_18_ column (flow rate: 1.0 mL/min; UV detection: 210 nm) to afford **6** (3.2 mg; t_R_ 27.0 min; ACN/H_2_O, 50/50), **9** (15.3 mg; t_R_ 40.3 min; MeOH/H_2_O, 68/32), and **10** (4.2 mg; t_R_ 17.8 min; ACN/H_2_O, 63/37), respectively.

Similarly, Fr.C was separated by the column of ODS (200 g) successively eluting with 30%, 50%, and 70% MeOH (each 400 mL) to produce three subfractions of SFr.C_1_, SFr.C_2_, and SFr.C_3_. SFr.C_1_ was further separated by preparative HPLC using a Fuji-C_18_ CT-30 column (280 × 30 mm, 10 µm; mobile phase: MeOH/H_2_O, 20–50/80–50, in 50 min; flow rate: 10 mL/min; UV detection: 210 nm) to produce SFr.C_1a_ and SFr.C_1b_. SFr.C_1a_, SFr.C_1b_ and SFr.C_3_ were purified on the Zorbax SB-C_18_ column (flow rate: 1.0 mL/min; UV detection: 210 nm) to furnish **12** (2.1 mg; t_R_ 32.3 min; MeOH/0.1%TFA in H_2_O, 40/60), **8** (3.1 mg; t_R_ 22.1 min; MeOH/H_2_O, 45/55), and **5** (1.8 mg; t_R_ 32.3 min; MeOH/H_2_O, 70/30), respectively. Compound **7** (1.9 mg; t_R_ 47.6 min) was obtained from SFr.C_2_ via separation on the Fuji-C_18_ CT-30 column (mobile phase: MeOH/H_2_O, 40/60; flow rate: 10 mL/min; UV detection: 210 nm).

Finally, Fr.D was separated on the Fuji-C_18_ CT-30 column (mobile phase: MeOH/H_2_O, 20–50/80–50, in 50 min; flow rate: 10 mL/min; UV detection: 210 nm) to produce SFr.D_1_, SFr.D_2_, and SFr.D_3_. Further separation by HPLC using the same Zorbax SB-C_18_ column, the same flow rate of 1 mL/min, and the same UV detection of 210 nm, compound **2** (4.6 mg; t_R_ 32.0 min; MeOH/H_2_O, 40/60) was obtained from SFr.D_1_, **4** (3.5 mg; t_R_ 40.6 min; MeOH/H_2_O, 40/60) and **3** (5.2 mg; t_R_ 43.0 min; MeOH/H_2_O, 40/60), from SFr.D_2_ and **11** (2.4 mg; t_R_ 31.2 min; and MeOH/H_2_O, 45/55) from SFr.D_3_.

### 3.5. Compound Characterization Data

Penipyridinone B (**1**): White amorphous powder; molecular formula C_43_H_61_NO_8_; [*α*]^20^_D_ +80° (*c* 0.02, MeOH); UV (MeOH) λ_max_ (log ε) 271(3.89), 276(4.2) nm; ECD (4.5 mg/L, MeOH) λ_max_ (∆ε) 196 (−24.61), 218 (+22.35), 276 (+18.08), 333 (−12.17); IR (ATR) ν_max_ 3393, 3024, 2922, 2857, 1820, 1642, 1589, 1453, 1391, 1259, 1175, 1120, 1032, 987, 840, 706 cm^−1^; ^13^C NMR (150 MHz) and ^1^H NMR (600 MHz) data, see Table 1; HRESIMS data *m*/*z* 720.4473 [M + H]^+^ (calcd for C_43_H_62_NO_8_^+^, 720.4475) and 742.4292 [M + Na]^+^ (calcd for C_43_H_61_NNaO_8_^+^, 742.4295).

11*S*-(−)-Penilloid A (**2**): Yellow amorphous solid; molecular formula C_17_H_15_N_5_O_2_; [*α*]^20^_D_ −201.6 (*c* 0.16, MeOH); UV (MeOH) λ_max_ (log ε) 221 (3.80), 308 (3.75) nm; ECD (10.0 mg/L, MeOH) λ_max_ (∆ε) 197 (+10.87), 222 (−11.32), 309 (−12.28); IR (ATR) ν_max_ 3265, 1667, 1640, 1441, 1092 cm^−1^; ^13^C NMR (150 MHz) and ^1^H NMR (600 MHz) data, see Table 2; HRESIMS data *m*/*z* 322.1298 [M + H]^+^ (calcd for C_17_H_16_N_5_O_2_^+^, 322.1304) and 344.1118 [M + Na]^+^ (calcd for C_17_H_15_N_5_NaO_2_^+^, 344.1123).

11*R*,14*E*-(+)-Penilloid A (**3**): Yellow amorphous solid; molecular formula C_17_H_15_N_5_O_2_; [*α*]^20^_D_ +203.8 (*c* 0.18, MeOH); UV (MeOH) λ_max_ (log ε) 220 (3.87), 309 (3.68) nm; ECD (10.0 mg/L, MeOH) λ_max_ (∆ε) 220 (−61.65), 329 (+22.46); IR (ATR) ν_max_ 3139, 1674, 1631, 1439, 1202, 1131 cm^−1^; ^13^C NMR (150 MHz) and ^1^H NMR (600 MHz) data, see Table 3; HRESIMS data *m*/*z* 322.1302 [M + H]^+^ (calcd for C_17_H_16_N_5_O_2_^+^, 322.1304) and 344.1116 [M + Na]^+^ (calcd for C_17_H_15_N_5_NaO_2_^+^, 344.1123).

### 3.6. Esterification of Penipyridinone B (***1***)

Penipyridinone B (**1**, 1.0 mg) and dimethylaminopyridine (1.1 mg) were dissolved in 0.5 mL anhydrous pyridine; then, either *R*-MTPA-Cl or *S*-MTPA-Cl (50 μL) was added. The mixture was stirred at room temperature for 48 h until 1 mL MeOH was added to terminate the reaction. The reaction mixture was dried under reduced pressure to provide a residue. This residue was separated by the Zorbax SB-C_18_ column HPLC (mobile phase: MeOH/H_2_O, 92/8; flow rate: 1 mL/min; UV detection: 275 nm) to yield *S*-MTPA ester **1s** (0.5 mg, t_R_ 33.5 min,) or *R*-MTPA ester **1r** (0.4 mg, t_R_ 32.8 min, MeOH/H_2_O, 92/8).

*S*-MTPA ester **1s**: ^1^H NMR data (600 MHz, in MeOH-*d*_4_), see Table S15; HRESIMS data *m*/*z* 958.4699 [M + Na]^+^ (calcd for C_53_H_68_F_3_NNaO_10_^+^, 958.4688).

*R*-MTPA ester **1r**: ^1^H NMR data (600 MHz, in MeOH-*d*_4_), see Table S15; HRESIMS data *m*/*z* 936.4879 [M + H]^+^ (calcd for C_53_H_69_F_3_NO_10_^+^, 936.4868) and 958.4709 [M + Na]^+^ (calcd for C_53_H_68_F_3_NNaO_10_^+^, 958.4688).

### 3.7. Optical Rotation (OR) Calculations

Monte Carlo conformational searches were carried out by means of the Spartan’s 10 software using the Merck Molecular Force Field (MMFF). The chosen conformers for OR calculations were initially optimized at the B3LYP/6-31+g (d, p) level in MeOH using the Conductor-like Polarizable Continuum Model (CPCM). The theoretical calculations of OR were conducted in MeOH using the time-dependent density functional theory (TD-DFT) at the B3LYP/6-311+g (d, p) level for all conformers of the tested compound.

### 3.8. Electronic Circular Dichroism (ECD) Calculations

Monte Carlo conformational searches were carried out by means of the Spartan’s 10 software using the Merck Molecular Force Field (MMFF). The chosen conformers for ECD calculations were initially optimized at the B3LYP/6-31+g (d, p) level in MeOH using the CPCM polarizable conductor calculation model. The theoretical calculations of ECD were conducted in MeOH using the time-dependent density functional theory (TD-DFT) at the B3LYP/6-311+g (d, p) level for all conformers of the tested compound. Rotatory strengths for a total of 30 excited states were calculated. ECD spectra were generated using the program SpecDis 1.6 (University of Würzburg, Würzburg, Germany) and GraphPad Prism 5 (University of California San Diego, San Diego, CA, USA) from dipole-length rotational strengths by applying Gaussian band shapes with sigma = 0.3 eV.

### 3.9. Sulforhodamine B (SRB) Assay

Human glioma U87MG and U251 cells were cultured in Minimum Essential Medium, Gibco (MEM) and Dulbecco’s Modified Eagle Medium, Gibco (DMEM) containing 10% fetal bovine serum (FBS) (PAA Laboratories Inc., Toronto, ON, Canada) and 1% penicillin-streptomycin, respectively. All cells were incubated at 37 °C in a humidified incubator with 5% CO_2_ incubator and used for experiment after the third generation. The activity of all isolated compounds **1**–**12** against the proliferation of U251 and U87MG cells were evaluated using the SRB assay, as described in a previous publication [36]. Doxorubicin (DOX) was used as a positive control. Briefly, cells were seeded in 96-well plates for 24 h at a density of 2 × 10^3^ cells per well of 100 μL and incubated at 37 °C in 5% CO_2_. Then, cells were treated with compounds and a positive control drug for 72 h. After that, the cells were fixed with a 10% cold trichloroacetic acid solution and stained with SRB. Then, a microplate reader (Bio Tek, Winooski, VT, USA) was used to determine the optical density (OD) value at absorbance of 515 nm. The mean value of five wells was calculated to obtain cell viability. The cell viability (%) was calculated using the following formula: cell viability (%) = T_OD_/C_OD_ × 100%, where T_OD_ is the OD value of the tested compound and C_OD_ is the OD value of the blank control (0.3% DMSO in water). IC_50_ values were calculated by using GraphPad software and presented as the mean ± SD (n = 5).

### 3.10. Reactive Oxygen Species (ROS) Measurement

The ROS level was measured by using the DCFH-DA kit (Yeasen Biotechnology, Shanghai, China) according to the manufacturer’s instruction. Briefly, glioma U251 and U87MG cells in logarithmic growth phase were cultured in 96-well plates with a density of 3 × 10^3^ cells per well for 24 h, then treated with tested compounds, positive control H_2_O_2_, or blank control DMSO. After the treatment of the set time points, the treated cells were exposed to a 50 μL DCFH-DA solution (10 μM) for 30 min. Excess DCFH-DA was removed by washing the cells twice with phosphate buffered saline (PBS). Fluorescence was immediately measured in a microplate reader (Synergy 2, Bio Tek) with an excitation/emission wavelength of 485/525 nm.

### 3.11. Mitochondrial Membrane Potential (MMP) Measurement

The MMP was determined using the specific MMP fluorescent probe JC-1 kit (Beyotime Biotechnology, Shanghai, China). Briefly, glioma U251 and U87MG cells (3 × 10^3^ cells per well) in logarithmic growth phase were cultured in 96-well plates for 24 h, then treated with tested compounds, positive control bortezomib (BTZ), or blank control DMSO at the set time points. The treated cells were incubated for 20 min at 37 °C with a 50 μL JC-1 working solution, then washed twice with PBS. The stained cells were measured in a microplate reader (excitation/emission wavelength 485/528 nm for green and 500/590 nm for red). The MMP was indicated by the fluorescent ratio of red/green.

### 3.12. Caspase-3 Activity Determination

The Caspase-3 activity was determined by using the GreenNuc™ Caspase-3 Substrate kit (Beyotime Biotechnology). Briefly, glioma U251 and U87MG cells (3 × 10^3^ cells per well) in logarithmic growth phase were cultured in 96-well black plates for 24 h, then treated with tested compounds or blank control DMSO for 48 h. The treated cells were incubated for 30 min at room temperature in dark with a 100 μL GreenNuc™ Caspase-3 Substrate solution (5 μM), then washed twice with PBS. The stained cells were measured in a microplate reader (excitation/emission wavelength 485/515).

### 3.13. Statistical Analysis

All data are presented as the mean ± SD. GraphPad Prism 7.0 was used for statistical analysis. Comparisons between two groups were carried out with a two-tailed Student’s *t*-test. Variances among more than two groups were analyzed with one-way ANOVA. The *p* value < 0.05 was considered to indicate statistical significance.

## 4. Conclusions

Marine-derived *Penicillium* fungi are one of the most important sources for the discovery of new bioactive natural products. A large-scale culture of the marine-associated fungus *Penicillium* sp. ZZ1750 in GA liquid medium resulted in the isolation and identification of eleven compounds (**2**–**12**), including new alkaloids 11*S*-(−)-penilloid A (**2**) and 11*R*,14*E*-(+)-penilloid A (**3**).The structures of the new 11*S*-(−)-penilloid A (**2**) and 11*R*,14*E*-(+)-penilloid A (**3**), as well as that of the previously unidentified penipyridinone B (**1**), were determined by a combination of HRESIMS data, extensive NMR spectroscopic analyses, Mosher’s method, OR calculations, and ECD calculations. Penipyridinone B (**1**) is the first example of its structural type of compound, consisting of a unique meroterpenoid and a tetrahydropyran derivative. The new penipyridinone B (**1**) and the known questiomycin A (**9**) and xanthocillin X (**10**) showed antiproliferative activity against glioma cells. Questiomycin A (**9**) and xanthocillin X (**10**) significantly increased the ROS production and the caspase-3 activity and reduced the MMP level in glioma cells.

Compounds **2**–**7** were six indole alkaloids, as the major metabolites of strain ZZ1750 in GA liquid medium, and showed no antiglioma activity. It is well known that marine-derived indole alkaloids possess not only intriguing structures, but also diverse biological activities [40]. Therefore, other activities of these isolated indole alkaloids, especially the new 11*S*-(−)-penilloid A (**2**) and 11*R*,14*E*-(+)-penilloid A (**3**), need to be further evaluated.

The data from our previous publications and current study indicated that the fungal strain ZZ1750 produced different structural types of secondary metabolites in different culture conditions. The rare glycosylated alkylresorcinols of peniresorcinosides A–E were the only metabolites found in the rice solid medium for a 30 day culture and the polyhydroxanthones of ergochromes D–G were only found in the rice solid medium for a 90 day culture. In the GA liquid medium, the strain ZZ1750 produced the indole alkaloids, but no glycosylated alkylresorcinols or polyhydroxanthones.

## Figures and Tables

**Table 1:** ^13^C NMR (150 MHz) and ^1^H NMR (600 MHz) data of penipyridinone B (**1**).

No.	1 (in MeOH-*d*_4_)		1 (in DMSO-*d*_6_)	
	*δ*_C_, Type	*δ*_H_, Mult (*J* in Hz)	*δ*_C_, Type	*δ*_H_, Mult (*J* in Hz)
1	72.0, CH_2_	3.76, d (11.5); 3.61, dd (11.5, 1.6)	69.9, CH_2_	3.67 *^a^*, m; 3.53 *^b^*, m
2	33.9, CH	2.30, m	31.8, CH	2.25, m
3	82.9, CH	3.45, dd (9.7, 5.5)	80.5, CH	3.40, dd (9.9, 5.1)
4	71.0, CH	5.00, t (9.7)	69.1, CH	4.84, t (9.9)
5	86.9, CH	3.57, d (9.7)	84.1, CH	3.56, d (9.9)
6	134.0, C	–	132.8, C	–
7	131.6, CH	6.06, d (10.1)	129.4, CH	6.01, d (10.3)
8	127.3, CH	6.29, dd (15.0, 10.1)	126.1, CH	6.29, dd (14.9, 10.3)
9	136.0, CH	6.27, dd (15.0, 10.0)	133.9, CH	6.23, dd (14.9, 10.1)
10	132.5, CH	6.12, dd (15.1, 10.0)	130.9, CH	6.10, dd (15.0, 10.1)
11	136.6, CH	5.72, m	134.9, CH	5.70 *^c^*, m
12	36.2, CH_2_	2.06, m	34.4, CH_2_	2.03, m
13	23.7, CH_2_	1.41, m	21.5, CH_2_	1.34, m
14	14.3, CH_3_	0.91, t (7.5)	13.6, CH_3_	0.85, t (7.3)
15	11.2, CH_3_	1.07, d (7.0)	10.7, CH_3_	0.94, d (7.2)
16	12.0, CH_3_	1.72, s	11.6, CH_3_	1.63, s
17	56.9, CH_3_	3.34, s	55.9, CH_3_	3.24, s
18	171.2, C	–	168.9, C	–
19	46.4, CH_2_	3.75, d (17.6); 3.65, d (17.6)	44.8, CH_2_	3.67 *^a^*, m; 3.53 *^b^*, m
20	114.3, C	–	112.6, C	–
21	117.4, CH	5.54, s	114.7, CH	5.38, s
22	147.1, C	–	145.3, C	–
23	201.4, C	–	199.3, C	–
24	58.8, C	–	56.8, C	–
25	111.6, C	–	109.3, C	–
26	141.3, CH	6.15, d (1.8)	140.0, CH	6.28, s
27	8.8, CH_3_	1.62, d (1.8)	8.3, CH_3_	1.51, s
28	29.6, CH_2_	2.10, m; 1.18, t (13.8)	26.3, CH_2_	2.22, m; 0.97, m
29	32.6, CH	1.67, m	31.0, CH	1.56, m
30	82.1, C	–	79.8, C	–
31	50.4, CH_2_	2.54, dd (13.2, 8.6); 2.34, m	49.1, CH_2_	2.40, dd (13.5, 9.5); 2.24, m
32	123.3, CH	5.30, m	121.3, CH	5.20, m
33	145.3, CH	5.26, d (15.8)	143.1, CH	5.18, d (15.3)
34	37.4, C	–	35.7, C	–
35	43.1, CH_2_	2.19, m; 1.76, dd (11.9, 5.5)	41.6, CH_2_	2.00, m; 1.68, m
36	126.1, CH	5.42, dd (11.9, 5.5)	121.8, CH	5.28, dd (12.2, 5.0)
37	139.0, C	–	138.7, C	–
38	69.0, CH	4.55, m	67.5, CH	4.43, br s
39	39.7, CH_2_	1.89, m; 1.42, m	38.1, CH_2_	1.81, m; 1.25, m
40	23.7, CH_3_	1.15, s	22.2, CH_3_	1.01, s
41	29.0, CH_3_	1.10, s	27.4, CH_3_	1.06, s
42	27.1, CH_3_	1.05, s	26.9, CH_3_	1.01, s
43	20.2, CH_3_	1.74, s	20.5, CH_3_	1.70, s
NH-19	–	–	–	5.70 *^c^*, m

^*a*, *b*, *c*^ The data with the same label in each column were overlapped.

**Table 2:** ^13^C NMR (150 MHz) and ^1^H NMR (600 MHz) data of 11*S*-(−)-penilloid A (**2**) and penilloid A.

No.	2 (in MeOH-*d*_4_)		2 (in DMSO-*d*_6_)		Penilloid A *^a^* (in MeOH-*d*_4_)	
	*δ*_C_, Type	*δ*_H_, Mult (*J* in Hz)	*δ*_C_, Type	*δ*_H_, Mult (*J* in Hz)	*δ*_C_, Type	*δ*_H_, Mult (*J* in Hz)
NH-1	–	–	–	10.84, s	–	–
2	125.9, CH	7.04, s	124.3, CH	7.03, s	125.8, CH	7.06, s
3	108.9, C	–	108.1, C	–	108.8, C	–
4	120.1, CH	7.56, d (7.5)	118.8, CH	7.58, d (7.8)	120.0, CH	7.60, d (7.0)
5	119.7, CH	6.93, t (7.5)	118.3, CH	6.92, t (7.8)	119.5, CH	6.98, t (7.0)
6	122.4, CH	6.96, t (7.5)	120.7, CH	6.98, t (7.8)	122.3, CH	7.01, t (7.0)
7	112.1, CH	7.20, d (7.5)	111.1, CH	7.24, d (7.8)	112.0, CH	7.23, d (7.0)
8	129.2, C	–	127.7, C	–	129.1, C	–
9	138.0, C	–	135.7, C	–	137.9, C	–
10	31.9, CH_2_	3.43, dd (14.7, 4.5); 3.23, dd (14.7, 4.5)	29.2, CH_2_	3.32, dd (14.7, 4.7); 3.07, dd (14.7, 4.7)	31.8, CH_2_	3.49, dd (14.5, 4.5); 3.28, dd (14.5, 4.5)
11	58.2, CH	4.44, t (4.5)	55.9, CH	4.45, t (4.7)	58.1, CH	4.48, t (4.0)
NH-12	–	–	–	8.34, s	–	–
13	162.4, C	–	158.5, C	–	162.3, C	–
14	125.2, C	–	124.7, C	–	125.1, C	–
NH-15	–	–	–	11.37, s	–	–
16	168.3, C	–	165.0, C	–	168.2, C	–
17	106.7, CH	6.29, s	102.7, CH	6.25, s	106.6, CH	6.31, s
18	138.2, C	–	136.3, C	–	137.9, C	–
20	137.1, CH	7.64, s	136.1, CH	7.83, s	137.0, CH	7.66, s
NH-21	–	–	–	12.46, br s	–	–
22	119.5, CH	7.14, s	118.2, CH	7.34, s	119.4, CH	7.15, s

*^a^* From the reference data [33].

**Table 3:** ^13^C NMR (150 MHz) and ^1^H NMR (600 MHz) data of 11*R*,14*E*-(+)-penilloid A (**3**).

No.	3 (in MeOH-*d*_4_)		3 (in DMSO-*d*_6_)	
	*δ*_C_, type	*δ*_H_, mult (*J* in Hz)	*δ*_C_, type	*δ*_H_, mult (*J* in Hz)
NH-1	–	–	–	10.88, s
2	126.9, CH	6.96, s	124.9, CH	6.99, d (1.9)
3	108.4, C	–	107.3, C	–
4	120.9, CH	7.51, d (7.8)	118.6, CH	7.52, d (7.5)
5	120.4, CH	6.91, t (7.8)	118.5, CH	6.92, t (7.5)
6	122.4, CH	6.97, t (7.8)	120.7, CH	6.94, t (7.5)
7	111.8, CH	7.05, d (7.8)	110.9, CH	7.16, d (7.5)
8	129.2, C	–	127.5, C	–
9	138.0, C	–	135.9, C	–
10	31.8, CH_2_	3.44, dd (14.8, 3.2); 3.15, dd (14.8, 4.5)	29.5, CH_2_	3.32, dd (14.7, 3.5); 3.06, dd (14.7, 5.0)
11	58.6, CH	4.37, dd (4.5, 3.2)	56.1, CH	4.38, dd (5.0, 3.5)
NH-12	–	–	–	9.11, d (2.2)
13	163.1, C	–	160.0, C	–
14	129.5, C	–	128.1, C	–
NH-15	–	–	–	10.47, s
16	170.0, C	–	166.6, C	–
17	103.6, CH	5.35, s	101.8, CH	5.68, s
18	129.1, C	–	126.8, C	–
20	134.1, CH	8.62, s	133.7, CH	8.82, s
NH-21	–	–	–	14.32, brs
22	119.8, CH	7.36, s	120.3, CH	7.71, s

**Table 4:** Activity of compounds in inhibiting the proliferation of glioma cells (IC_50_: µM).

Compounds	U87MG	U251
Penipyridinone B (**1**)	2.45 ± 0.07	11.40 ± 0.49
Questiomycin A (**9**)	14.13 ± 0.09	22.56 ± 0.13
Xanthocillin X (**10**)	16.33 ± 0.23	13.65 ± 0.23
Doxorubicin (DOX)	3.76 ± 0.30	9.85 ± 0.25

## Data Availability

All the data in this research are presented in the manuscript and in the supplementary materials.

## Supplementary Materials

The following supporting information can be downloaded at: https://www.mdpi.com/article/10.3390/molecules27207099/s1, Table S1: Sequences producing significant alignments of strain ZZ1750; Tables S2–S5: ^13^C and ^1^H NMR data of compounds **4**–**12**; Table S6: ^1^H NMR data of the MTPA esters **1s** and **1r**; Tables S7–S14: Data of the ECD calculations of penipyridinone B (**1**); Tables S15–S18: Data of the OR calculations of 11*S*-(−)-penilloid A (**2**); Tables S19–S22: Data of the ECD calculations of 11*S*-(−)-penilloid A (**2**); Tables S23–S26: Data of the OR calculations of 11*R*,14*E*-(+)-penilloid A (**3**); Tables S27–S30: Data of the ECD calculations of 11*R*,14*E*-(+)-penilloid A (**3**); Figure S1: ITS rDNA sequence of *Penicillium* sp. ZZ1750; Figure S2. Static culture state of strain *Penicillium* sp. ZZ1750 in GA liquid medium; Figures S3–S39: NMR spectra of penipyridinone B (**1**); Figure S40: HRESIMS spectrum of penipyridinone B (**1**); Figures S41–S46: NMR spectra of the MTPA esters **1s** and **1r**; **Figures S47–S64**: NMR spectra of 11*S*-(−)-penilloid A (**2**); Figure S65: HRESIMS spectrum of 11*S*-(−)-penilloid A (**2**); Figures S66–S82: NMR spectra of 11*R*,14*E*-(+)-penilloid A (**3**); Figure S83: HRESIMS spectrum of 11*R*,14*E*-(+)-penilloid A (**3**).

## Sample Availability

Samples of the compounds **1**–**12** are available from the authors.

## References

[1] Ma H.G., Liu Q., Zhu G.L., Liu H.S., Zhu W.M. (2016). Marine natural products sourced from marine-derived *Penicillium* fungi. J. Asian Nat. Prod. Res..

[2] Zhang P., Wei Q., Yuan X., Xu K. (2020). Newly reported alkaloids produced by marine-derived *Penicillium* species (covering 2014–2018). Bioorg. Chem..

[3] Yang X., Liu J., Mei J., Jiang R., Tu S., Deng H., Liu J., Yang S., Li J. (2021). Origins, structures, and bioactivities of secondary metabolites from marine-derived *Penicillium* fungi. Mini-Rev. Med. Chem..

[4] Li Y., Li X., Li X., Yang S., Wang B., Li H. (2022). Verrucosidin derivatives from the deep sea cold-seep-derived fungus *Penicillium polonicum* CS-252. Int. J. Mol. Sci..

[5] Wang J.Q., Li T.W., Wang P.M., Ding W.J., Xu J.Z. (2022). Tanzawaic acids from a deep-sea derived *Penicillium* species. J. Nat. Prod..

[6] Hu X.Y., Li X.M., Wang B.G., Meng L.H. (2022). Tanzawaic acid derivatives: Fungal polyketides from the deep-sea coral-derived endozoic *Penicillium steckii* AS-324. J. Nat. Prod..

[7] Hu X.Y., Li X.M., Wang B.G., Meng H.L. (2022). Uncommon polyketides from *Penicillium steckii* AS-324, a marine endozoic fungus isolated from deep-sea coral in the Magellan seamount. Int. J. Mol. Sci..

[8] Fan H., Shi Z.M., Lei Y.H., Si-Tu M.X., Zhou F.G., Feng C., Wei X., Shao X.H., Chen Y., Zhang C.X. (2022). Rare carbon-bridged citrinin dimers from the starfish-derived symbiotic fungus *Penicillium* sp. GGF16-1-2. Mar. Drugs.

[9] Zhang Y.H., Li L., Li Y.Q., Luo J.H., Li W., Li L.F., Zheng C.J., Cao F. (2022). Oxalierpenes A and B, unusual indole-diterpenoid derivatives with antiviral activity from a marine-derived strain of the fungus *Penicillium oxalicum*. J. Nat. Prod..

[10] Yao F.H., Liang X., Lu X.H., Cheng X., Luo L.X., Qi S.H. (2022). Pyrrospirones K-Q, decahydrofluorene-class alkaloids from the marine-derived fungus *Penicillium* sp. SCSIO 41512. J. Nat. Prod..

[11] Chen M.X., Chai W.Y., Song T.F., Ma M.Z., Lian X.Y., Zhang Z.Z. (2018). Anti-glioma natural products downregulating tumor glycolytic enzymes from marine actinomycetes *Streptomyces* sp. ZZ406. Sci. Rep..

[12] Song T.F., Chen M.X., Chai W.Y., Zhang Z.Z., Lian X.Y. (2018). New bioactive pyrrospirones C-I from a marine-derived fungus *Penicillium* sp. ZZ380. Tetrahedron.

[13] Song T.F., Tang M.M., Ge H.G., Chen M.X., Lian X.Y., Zhang Z.Z. (2019). Novel bioactive penicipyrroether A and pyrrospirone J from the marine-derived *Penicillium* sp. ZZ380. Mar. Drugs.

[14] Zhang D., Yi W.W., Ge H.J., Zhang Z.Z., Wu B. (2019). Bioactive streptoglutarimides A–J from the marine-derived *Streptomyces* sp. ZZ741. J. Nat. Prod..

[15] Qin L., Yi W.W., Lian X.Y., Zhang Z.Z. (2020). Bioactive alkaloids from the actinomycete *Actinoalloteichus* sp. ZZ1866. J. Nat. Prod..

[16] Ge H.J., Zhang D., Shi M.R., Lian X.Y., Zhang Z.Z. (2021). Antiproliferative activity and potential mechanism of marine-sourced streptoglutarimide H against lung cancer cells. Mar. Drugs.

[17] Yong K., Kaleem S., Wu B., Zhang Z.Z. (2021). New antiproliferative compounds against glioma cells from the marine-sourced fungus *Penicillium* sp. ZZ1750. Mar. Drugs.

[18] Yi W.W., Lian X.Y., Zhang Z.Z. (2022). Cytotoxic metabolites from the marine-associated *Streptomyces* sp. ZZ1944. Phytochemistry.

[19] Yong K., Kaleem S., Yi W.W., Wu B., Zhang Z.Z. (2021). New polyhydroxanthones from the marine-associated fungus *Penicillium* sp. ZZ1750. Tetrahedron Lett..

[20] Wang J.F., He W.J., Qin X.C., Wei X.Y., Tian X.P., Liao L., Liao S.R., Yang B., Tu Z.C., Chen B. (2015). Three new indolyl diketopiperazine metabolites from the antarctic soil-derived fungus *Penicillium* sp. SCSIO 05705. RSC Adv..

[21] Tang H.Y., Zhang Q., Li H., Gao J.M. (2015). Antimicrobial and allelopathic metabolites produced by *Penicillium brasilianum*. Nat. Prod. Res..

[22] Wang F.Q., Jiang J., Ma H.R., Cheng L., Zhang G. (2017). Study on secondary metabolites of endophytic *Chaetomium* sp.. Chin. Tradit. Herb. Drugs.

[23] Cao Y.H., Zhang Z.H., Lin F.K., Lai D.W., Yao Y.R., Xie B.Y., Zhou L.G. (2016). Secondary metabolites of endophytic fungus *Acremonium implicatum* and their biological activities. Nat. Prod. Res. Dev..

[24] Teng X.C., Zhuang Y.B., Wang Y., Liu P.P., Xu Z.H., Zhu W.M. (2019). Secondary metabolites from *Penicillium* sp. gxwz406 symbiotic with the gorgonian *Echinogorgia flora*. Chin. J. Mar. Drugs.

[25] Graf E., Schneider K., Nicholson G., Ströbele M., Jones A.L., Goodfellow M., Beil W., Süssmuth R.D., Fiedler H.P. (2007). Elloxazinones A and B, new aminophenoxazinones from *Streptomyces griseus* Acta 2871. J. Antibiot..

[26] Shang Z., Li X.M., Li M., Li C.S., Wang B.G. (2012). Chemical profile of the secondary metabolites produced by a deep-sea sediment-derived fungus *Penicillium commune* SD-118. Chin. J. Oceanol. Limnol..

[27] Cao F., Yang Q., Shao C.L., Kong C.J., Zheng J.J., Liu Y.F., Wang C.Y. (2015). Bioactive 7-oxabicyclic[6.3.0]lactam and 12-membered macrolides from a gorgonian-derived *Cladosporium* sp. fungus. Mar. Drugs.

[28] Zheng J., Zhao D.S., Wu B., Wu L.J. (2002). A Study on chemical constituents in the herb of *Mentha spicata*. China J. Chin. Mater. Medica.

[29] Matsukuma S., Ohtsuka T., Kotaki H., Shirai H., Sano T., Watanabe K., Nakayama N., Itezono Y., Fujiu M., Shimma N. (1992). A new series of natural antifungals that inhibit P450 lanosterol C-14 demethylase I. Taxonomy, fermentation, isolation and structural elucidation. J. Antibiot..

[30] Barrett A.G.M., Bennett A.J., Menzer S., Smith M.L., White A.J.P., Williams D.J. (1999). Applications of crotonyl diisopino campheyl boranes in synthesis: Total synthesis of restrictinol. J. Org. Chem..

[31] El-Elimat T., Raja H.A., Ayers S., Kurina S.J., Burdette J.E., Mattes Z., Sabatelle R., Bacon J.W., Colby A.H., Grinstaff M.W. (2019). Meroterpenoids from *Neosetophoma* sp.: A dioxa[4.3.3]propellane ring system, potent cytotoxicity, and prolific expression. Org. Lett..

[32] Hensens O.D., Wichmann C.F., Liesch J.M., VanMiddlesworth F.L., Wilson K.E., Schwartz R.E. (1991). Structure elucidation of restricticin, a novel antifungal agent from *Penicillium restrictum*. Tetrahedron.

[33] He F., Han Z., Peng J., Qian P.Y., Qi S.H. (2013). Antifouling indole alkaloids from two marine derived fungi. Nat. Prod. Commun..

[34] Haghdani S., Gautun O.R., Koch H., Åstrand P.O. (2016). Optical rotation calculations for a set of pyrrole compounds. J. Phys. Chem. A.

[35] Ren Q., Zhao W.Y., Shi S.C., Han F.Y., Zhang Y.Y., Liu Q.B., Yao G.D., Lin B., Huang X.X., Song S.J. (2019). Guaiane-type sesquiterpenoids from the roots of *Daphne genkwa* and evaluation of their neuroprotective effects. J. Nat. Prod..

[36] Xin W.X., Ye X.W., Yu S.R., Lian X.Y., Zhang Z.Z. (2012). New apoamycin-type antibiotics and polyene acids from marine *Streptomyces fradiae* PTZ0025. Mar. Drugs.

[37] Qoorchi Moheb Seraj F., Heravi-Faz N., Soltani A., Ahmadi S.S., Shahbeiki F., Talebpour A., Afshari A.R., Ferns G.A., Bahrami A. (2022). Thymol has anticancer effects in U-87 human malignant glioblastoma cells. Mol. Biol. Rep..

[38] Yang Y., Ren Z.Z., Wei W.J., He Z.L., Deng Y.L., Wang Z., Fan Y.C., Zhou J., Jiang L.H. (2022). Study on the biological mechanism of urolithin a on nasopharyngeal carcinoma in vitro. Pharm. Biol..

[39] Asadi M., Taghizadeh S., Kaviani E., Vakili O., Taheri-Anganeh M., Tahamtan M., Savardashtaki A. (2021). Caspase-3: Structure, function, and biotechnological aspects. Biotechnol. Appl. Biochem..

[40] Wibowo J.T., Ahmadi P., Rahmawati S.I., Bayu A., Putra M.Y., Kijjoa A. (2022). Marine-derived indole alkaloids and their biological and pharmacological activities. Mar. Drugs.

