# Supplementary material for: Antiglioma Natural Products from the Marine-Associated Fungus Penicillium sp. ZZ1750
Source: Molecules. 2022 Oct 20;27(20):7099. doi: 10.3390/molecules27207099 (PMC9611354; doi:10.3390/molecules27207099)
Supplement: Supplementary file 1 [file molecules-27-07099-s001.zip › molecules-1962244-supplementary.pdf]

SUPPLEMENTARY MATERIALS FOR

Antiglioma natural products from the marine-associated fungus *Penicillium* sp. ZZ1750

Kuo Yong[1, †], Sidra Kaleem[1, †], Mingzhu Ma[1,3], Xiao yuan Lian[2,*], Zhizhen Zhang[1,*]

1 Ocean College, Zhoushan Campus, Zhejiang University, Zhoushan 316021, China

2 College of Pharmaceutical Sciences, Zhejiang University, Hangzhou 310058, China

3 Zhejiang Marine Development Research Institute, Zhoushan, 316000, China

† These authors contributed equally to this work.

* Correspondence: xylian@zju.edu.cn (X.L.); zzhang88@zju.edu.cn (Z.Z.); Tel: +86-13675859706 (Z.Z)

CONTENT

Table S1. Sequences producing significant alignments of strain ZZ1750……….………..……………5
Table S2. ^{13}C NMR (150 MHz) and ^{1}H NMR (600 MHz) data of compounds **4** and **5** (in DMSO-d_6)...6
Table S3. ^{13}C NMR (150 MHz) and ^{1}H NMR (600 MHz) data of compounds **6**–**8** (in DMSO-d_6)…....7
Table S4. ^{13}C NMR (150 MHz) and ^{1}H NMR (600 MHz) data of compounds **9** and **10** (in DMSO-d_6)..7
Table S5. ^{13}C NMR (150 MHz) and ^{1}H NMR (600 MHz) data of compounds **11** and **12** (in DMSO-d_6)…………………………………………………………………………………........................8
Table S6. ^{1}H NMR data of the MTPA esters **1s** and **1r** (600 MHz, in MeOH-d_4, J in Hz)………………9
Table S7. Gibbs free energies and equilibrium populations of low-energy conformers of **1f**……….....10
Table S8. Cartesian coordinates for the low-energy reoptimized MMFF conformers of **1f** at B3LYP/6-31+g (d, p) level of theory in MeOH…………………………………………………………..…...10
Table S9. Gibbs free energies and equilibrium populations of low-energy conformers of **1g**………..21
Table S10. Cartesian coordinates for the low-energy reoptimized MMFF conformers of **1g** at B3LYP/6-31+g (d, p) level of theory in MeOH……………………………………………………………..21
Table S11. Gibbs free energies and equilibrium populations of low-energy conformers of **1h**…..……32
Table S12. Cartesian coordinates for the low-energy reoptimized MMFF conformers of **1h** at B3LYP/6-31+g (d, p) level of theory in MeOH….………………………………………………………..…32
Table S13. Gibbs free energies and equilibrium populations of low-energy conformers of **1i**….……..41
Table S14. Cartesian coordinates for the low-energy reoptimized MMFF conformers of **1i** at B3LYP/6-31+g (d, p) level of theory in MeOH……………………………………………………………..41
Table S15. Boltzmann population and optical rotation (OR) of low-energy conformers of 11*R*-**2**…....54
Table S16. Cartesian coordinates for the low-energy reoptimized MMFF conformers of 11*R*-**2** at B3LYP/6-311+G (d, p) level of theory in MeOH………………………………………………......54
Table S17. Boltzmann population and optical rotation (OR) of low-energy conformers of 11*S*-**2**……59
Table S18. Cartesian coordinates for the low-energy reoptimized MMFF conformers of 11*S*-**2** at B3LYP/6-311+G (d, p) level of theory in MeOH………………………………………………......59
Table S19. Gibbs free energies and equilibrium populations of low-energy conformers of 11*R*-**2**…..64
Table S20. Cartesian coordinates for the low-energy reoptimized MMFF conformers of 11*R*-**2** at B3LYP/6-311+G (d, p) level of theory in MeOH………………………………………………...64
Table S21. Gibbs free energies and equilibrium populations of low-energy conformers of 11*S*-**2**…..69
Table S22. Cartesian coordinates for the low-energy reoptimized MMFF conformers of 11*S*-**2** at B3LYP/6-311+G (d, p) level of theory in MeOH………………………………………………..…..69
Table S23. Boltzmann population and optical rotation (OR) of low-energy conformers of 11*R*-**3**……74
Table S24. Cartesian coordinates for the low-energy reoptimized MMFF conformers of 11*R*-**3** at B3LYP/6-311+G (d, p) level of theory in MeOH…………………………………………………...74

Table S25. Boltzmann populations and optical rotation (OR) of low-energy conformers of 11*S*-**3**….79
Table S26. Cartesian coordinates for the low-energy reoptimized MMFF conformers of 11*S*-**3** at B3LYP/6-311+G (d, p) level of theory in MeOH………………………………………………….…79
Table S27. Gibbs free energies and equilibrium populations of low-energy conformers of 11*R*-**3**…..84
Table S28. Cartesian coordinates for the low-energy reoptimized MMFF conformers of 11*R*-**3** at B3LYP/6-31+g (d, p) level of theory in MeOH……………………………………………………....84
Table S29. Gibbs free energies and equilibrium populations of low-energy conformers of 11*S*-**3**……………………………………..……………………………………...……………...89
Table S30. Cartesian coordinates for the low-energy reoptimized MMFF conformers of 11*S*-**3** at B3LYP/6-31+g (d, p) level of theory in MeOH………………………………………..…………89
Figure S1. ITS rDNA sequence of *Penicillium* sp. ZZ1750…….……………………………..95
Figure S2. Static culture state of strain *Penicillium* sp. ZZ1750 in GA liquid medium……………...95
Figures S3–S6. ^{1}H NMR spectra of penipyridinone B (**1**, in MeOH-d_4)………………………………96
Figures S7–S10. ^{13}C NMR spectra of penipyridinone B (**1**, in MeOH-d_4) ……………………………98
Figures S11–S13. DEPT spectra of penipyridinone B (**1**, in MeOH-d_4) ………………….……….100
Figures S14–S17. HMQC spectra of penipyridinone B (**1**, in MeOH-d_4)……….…………………101
Figures S18–S20. COSY spectra of penipyridinone B (**1**, in MeOH-d_4) ……….…………….….103
Figures S21–S23. HMBC spectra of penipyridinone B (**1**, in MeOH-d_4) ……………………………105
Figure S24. NOE spectrum of penipyridinone B (**1**, in MeOH-d_4) …………………….…………106
Figures S25–S28. ^{1}H NMR spectra of penipyridinone B (**1**, in DMSO-d_6) ……………….….107
Figures S29–S32. ^{13}C NMR spectra of penipyridinone B (**1**, in DMSO-d_6) …….……………….109
Figures S33–S36. HMQC spectra of penipyridinone B (**1**, in DMSO-d_6) …….………….…………111
Figures S37–S39. HMBC spectra of penipyridinone B (**1**, in DMSO-d_6) ……………..…….……….113
Figure S40. HRESIMS spectrum of penipyridinone B (**1**) ………….…………………….…….114
Figure S41. ^{1}H NMR spectrum of **1s**……………………………………..……………….…….115
Figure S42. COSY NMR spectrum of **1s**………...……..…………………..…………….…….115
Figure S43. HRESIMS spectrum of **1s**………………..………………….…...…………….…….116
Figure S44. 1H NMR spectrum of **1r**……..…………………………………….…………….…….116

Figure S45. COSY spectrum of **1r**……..…………………………………......……..…………..…….117
Figure S46. HRESIMS spectrum of **1r**…………………………...……....………...……….…….117
Figures S47–S49. ^{1}H NMR spectra of penilloid B (**2**, in DMSO-d_6)……..………..……...……………..…..118
Figures S50–S51. ^{13}C NMR spectra of penilloid B (**2**, in DMSO-d_6) …...…….………..……….……..119
Figures S52–S53. HMQC spectra of penilloid B (**2**, in DMSO-d_6) ….………...……….……………..120
Figure S54. COSY spectrum of penilloid B (**2**, in DMSO-d_6) ….………...………………………….121
Figures S55–S58. HMBC spectra of penilloid B (**2**, in DMSO-d_6) ……….……….…...…….……..122
Figure S59. NOESY spectrum of penilloid B (**2**, in DMSO-d_6) ………...……….……………………..124
Figures S60–S62. ^{1}H NMR spectra of penilloid B (**2**, in MeOH-d_4)…………………………………124
Figures S63–S64. ^{13}C NMR spectra of penilloid B (**2**, in MeOH-d_4) ………...……………...……..126
Figure S65. HRESIMS spectrum of penilloid B (**2**) …...………………...……………………..…..127
Figures S66–S68. ^{1}H NMR spectra of penilloid C (**3**, in DMSO-d_6) ……………………………....127
Figures S69–S70. ^{13}C NMR spectra of penilloid C (**3**, in DMSO-d_6) …………………….……….....129
Figures S71–S72. HMQC spectra of penilloid C (**3**, in DMSO-d_6) ……………………….……….....130
Figure S73. COSY spectrum of penilloid C (**3**, in DMSO-d_6) ………………….……..…………..…..131
Figures S74–S76. HMBC spectra of penilloid C (**3**, in DMSO-d_6) …………..….………………..…..131
Figure S77. NOESY spectrum of penilloid C (**3**, in DMSO-d_6)…………………….………………..…..133
Figures S78–S80. ^{1}H NMR spectra of penilloid C (**3**, in MeOH-d_4) ……………..…………………..…..133
Figures S81–S82. ^{13}C NMR spectra of penilloid C (**3**, in MeOH-d_4) …………….……………………..135
Figure S83. HRESIMS spectrum of penilloid C (**3**) ……………………………………………….....136

Table S1. Sequences producing significant alignments of strain ZZ1750

Accession	Description	Max score	Total score	Query coverage	Evalue	Ident
MT364482.1	*Penicillium rubens* strain EF5 small subunit ribosomal RNA gene, partial sequence; internal transcribed spacer 1, 5.8S ribosomal RNA gene, and internal transcribed spacer 2, complete sequence; and large subunit ribosomal RNA gene, partial sequence	1040	1040	100%	0.0	100%
MK830090.1	*Penicillium chrysogenum* strain CD3 small subunit ribosomal *RNA* gene, partial sequence; internal transcribed spacer 1, 5.8S ribosomal *RNA* gene	1040	1040	100%	0.0	100%
MH863913.1	*Penicillium chrysogenum* strain CBS 126337 small subunit ribosomal *RNA* gene, partial sequence; internal transcribed spacer 1, 5.8S ribosomal *RNA* gene	1040	1040	100%	0.0	100%
MN341258.1	*Penicillium rubens* isolate KoRLI047057 small subunit ribosomal RNA gene, partial sequence; internal transcribed spacer 1, 5.8S ribosomal RNA gene, and internal transcribed spacer 2, complete sequence; and large subunit ribosomal RNA gene, partial sequence	1040	1040	100%	0.0	100%
MN413181.1	*Penicillium rubens* strain DTO269E3 small subunit ribosomal RNA gene, partial sequence; internal transcribed spacer 1, 5.8S ribosomal RNA gene, and internal transcribed spacer 2, complete sequence; and large subunit ribosomal RNA gene, partial sequence	1040	1040	100%	0.0	100%
MN413180.1	*Penicillium rubens* strain DTO269C2 small subunit ribosomal RNA gene, partial sequence; internal transcribed spacer 1, 5.8S ribosomal RNA gene, and internal transcribed spacer 2, complete sequence; and large subunit ribosomal RNA gene, partial sequence	1040	1040	100%	0.0	100%

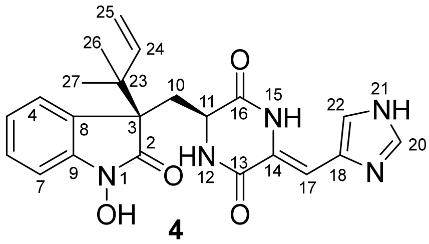

4

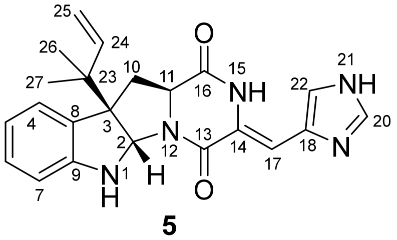

5

Compounds 4 and 5.

Table S2. ^{13}C NMR (150 MHz) and ^{1}H NMR (600 MHz) data of compounds **4** and **5** (in DMSO-d_6)

No.	**4**		**5**	
	δ_C, type	δ_H, J in Hz	δ_C, type	δ_H, J in Hz
1	–	6.86, br s	–	–
2	172.8, C	–	76.9, CH	5.56, s
3	55.0, C	–	60.8, C	–
4	125.3, CH	7.20, d (7.5)	125.6, CH	7.18, d (7.5)
5	121.9, CH	7.02, t (7.5)	118.9, CH	6.99, t (7.5)
6	128.5, CH	7.29, t (7.5)	128.7, CH	6.61, t (7.5)
7	106.9, CH	6.96, d (7.5)	108.2, CH	6.52, d (7.5)
8	124.2, C	–	128.4, C	–
9	143.1, C	–	151.4, C	–
10	36.3, CH_2	2.63, dd (14.6, 2.2);<br>2.33, dd (14.6,8.5)	37.6, CH_2	2.38, dd (12.0, 5.9);<br>2.33 dd (12.0, 11.5)
11	52.6, C	3.29 [a]	58.3, CH	4.00, dd (11.5, 5.9)
12	–	10.76, br s	–	–
13	158.5, C	–	156.5, C	–
14	125.0, C	–	124.8, C	–
15	–	12.53, br s	–	12.57, br s
16	164.3, C	–	164.4, C	–
17	103.9, CH	6.46, s	104.1, CH	6.59, s
18	136.3, C	–	136.3, C	–
20	136.4, CH	7.90, s	136.4, CH	7.92. s
21	–	11.36, s	–	12.55, s
22	118.8, CH	7.46, s	117.3, CH	7.52, s
23	42.1, C	–	40.7, C	–
24	142.6, CH	5.98, dd (17.5,10.5)	144.1, CH	6.01, dd (17.5,10.5)
25	114.2, CH	5.08, d (10.5);<br>4.96, d (17.5)	113.9, CH	5.07, d (10.5);<br>5.04, d (17.5)
26	21.9 [b], CH_3	1.03 [b] s	22.6 [b], CH_3	1.06 [b], s
27	21.5 [b], CH_3	0.94 [b], s	22.3 [b], CH_3	0.93 [b], s

[a] The signal was overlapped with that of H_2O; [b] The data with the same label in each column may be interchanged.

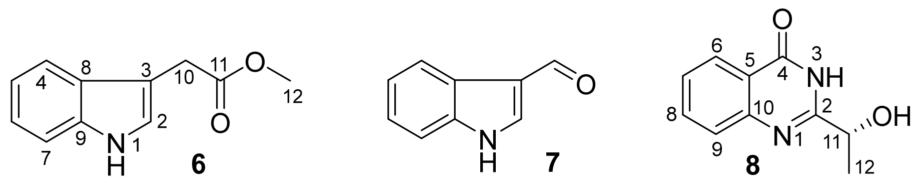

Compounds 6–8.

Table S3. ^{13}C NMR (150 MHz) and ^{1}H NMR (600 MHz) data of compounds **6–8** (in DMSO-d_6)

No.	**6**		**7**		**8**	
	δ_C, type	δ_H, J in Hz	δ_C, type	δ_H, J in Hz	δ_C, type	δ_H, J in Hz
1	–	10.95, br s	–	12.14, br s	–	–
2	124.1, CH	7.24, d (1.9)	137.0, CH	8.28, s	160.7, C	–
3	106.9, C	–	118.1, C	–	–	–
4	118.5, CH	7.47, d (8.2)	122.1, CH	8.08, d (8.0)	162.9, C	–
5	118.4, CH	6.97, t (8.2)	120.8, CH	7.20, t (8.0)	121.4, C	–
6	121.0 CH	7.06, t (8.2)	123.4, CH	7.24, t (8.0)	126.5 CH	8.01, d (8.0)
7	111.4, CH	7.34, d (8.2)	112.4, CH	7.50, d (8.0)	127.2, CH	7.38, t (8.0)
8	127.0, C	–	124.1, C	–	135.3, CH	7.68, t (8.0)
9	136.1, C	–	138.5, C	–	127.1, CH	7.55, d (8.0)
10	30.6, CH_2	3.74, s	184.9, CH	9.93, s	148.5, C	–
11	172.0, C	–			67.7, CH	4.61, q (6.9)
12	51.5, CH_3	3.60, s			22.3, CH_3	1.40, d (6.9)

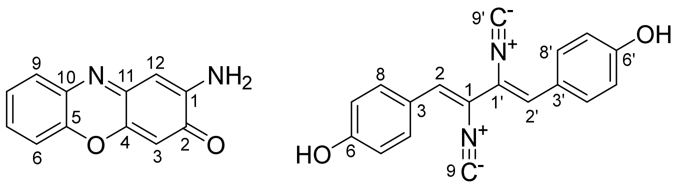

9 10

Compounds 9 and 10.

Table S4. ^{13}C NMR (150 MHz) and ^{1}H NMR (600 MHz) data of compounds **9** and **10** (in DMSO-d_6)

No.	**9**		**10**	
	δ_C, type	δ_H, J in Hz	δ_C, type	δ_H, J in Hz
1/1′	148.9, C	–	114.7, C	–
2/2′	180.2, C	–	127.6, CH	7.05, s
3/3′	103.4, CH	6.37, s	122.9, C	–
4/4′	147.4, C	–	131.9, CH	7.73, d (8.5)
5/5′	141.9, C	–	116.0, CH	6.89, d (8.5)
6/6′	115.9, CH	7.50, d (8.0)	159.7, C	–
OH-6	–	–	–	10.31, br s
7/7′	128.8 CH	7.45, td (8.0,1.5)	116.0, CH	6.89, d (8.5)
8/8′	125.3, CH	7.38, td (8.0,1.5)	131.9, CH	7.73, d (8.5)
9	128.0, CH	7.70, d (8.0)	172.6, C	–
10	133.7, C	–		
11	148.2, C	–		
12	98.3, CH	6.36, s		

NH-1	–	6.83, br s

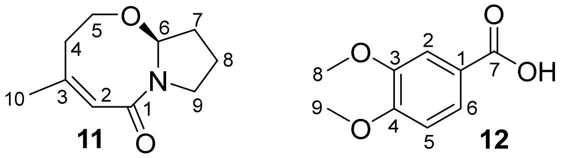

Compounds 11 and 12.

Table S5. ^{13}C NMR (150 MHz) and ^{1}H NMR (600 MHz) data of compounds **11** and **12** (in DMSO-d_6)

No.	**11**		**12**	
	δ_C, type	δ_H, J in Hz	δ_C, type	δ_H, J in Hz
1	166.2, C	–	123.0, C	–
2	121.4, CH	5.73, s	111.9, CH	7.43, d (1.5)
3	144.6, C	–	148.3, C	–
4	35.7, CH_2	2.35, dd (15.0, 9.5); 2.15, dd (15.0, 7.5);	152.6, C	–
5	62.5, CH_2	3.84, dd (12.0, 7.5); 3.41, dd (12.0, 9.5);	111.0, CH	7.04, d (8.5)
6	87.7, CH	5.14, d (4.5)	123.1, CH	7.55, dd (8.5, 1.5)
7	33.6, CH_2	1.97, m	167.1, C	–
8	21.3, CH_2	2.15, m; 1.86, m	55.6, CH_3	3.82, s
9	44.5, CH_2	3.41, m	55.4, CH_3	3.79, s
OH-9	–	–	–	12.67, br s
10	25.0, CH_3	1.83, s		

Table S6. ^{1}H NMR data of the MTPA esters **1s** and **1r** (600 MHz, in MeOH-d_4, J in Hz)

No.	*S*-MTPA-**1** (**1s**)	*R*-MTPA-**1** (**1r**)	$\Delta\delta_{S\text{-}R}$
1	3.77, 1H, dd (11.8, 1.6);	3.76, 1H, dd (12.0, 1.8);	+0.01;
	3.61, 1H, dd (11.8, 2.0)	3.61, 1H, dd (12.0, 2.3)	0
2	2.31, 1H, m	2.31, 1H, m	0
3	3.45, 1H, dd (9.7, 5.5)	3.45, 1H, dd (9.8, 5.4)	0
4	5.0, 1H, t (9.7)	5.0, 1H, t (9.8)	0
5	3.57, 1H, d (9.7)	3.57, 1H, d (9.8)	0
7	6.05, 1H, d (10.1)	6.05, 1H, d (10.1)	0
8	6.31, 1H, dd (14.9, 10.1)	6.31, 1H, dd (14.9, 10.1)	0
9	6.26, 1H, dd (14.9, 10.1)	6.26, 1H, dd (14.9, 9.9)	0
10	6.12, 1H, dd (14.9, 10.1)	6.13, 1H, dd (14.9, 9.9)	−0.01
11	5.72, 1H, m	5.72, 1H, m	0
12	2.08, 2H, m	2.08, 2H, m	0
13	1.42, 2H, m	1.42, 2H, m	0
14	0.91, 3H, t (7.5)	0.90, 3H, t (7.3)	+0.01
15	1.06, 3H, d (7.3)	1.05, 3H, d (7.2)	+0.01
16	1.72, 3H, s	1.72, 3H, s	0
17	3.34, 3H, s	3.34, 3H, s	0
19	3.75, 1H, d (17.8);	3.75, 1H, d (18.0);	0;
	3.64, 1H, d (17.8)	3.64, 1H, d (18.0)	0
21	5.52, 1H, s	5.51, 1H, s	+0.01
26	6.15, 1H, s	6.13, 1H, s	+0.02
27	1.57, 3H, s	1.49, 3H, s	+0.08
28	1.84, 1H, m; 1.07, 1H, m	2.01,1H, m; 1.17, 1H, m	−0.17; −0.10
29	1.68, 1H, s	1.74, 1H, s	−0.06
31	2.56, 1H, dd (13.7, 8.7);	2.55, 1H, dd (12.7, 9.2);	+0.01;
	2.36, 1H, dd (13.7, 5.3)	2.35, 1H, dd (12.7, 5.0)	+0.01;
32	5.40, 1H, m	5.37, 1H, m	+0.03
33	5.31, 1H, d (15.8)	5.30, 1H, d (15.9)	+0.01
35	2.30, 1H, m; 1.84, 1H, m	2.26, 1H, m; 1.82, 1H, m	+0.04; +0.02
36	5.62, 1H, dd (11.7, 5.6)	5.52, 1H, m	+0.10
38	5.95, 1H, dd (8.7, 4.5)	5.92, 1H, dd (7.0, 4.9)	+0.03
39	1.93, 1H, m; 1.49, 1H, m	2.10, 1H, m; 1.57, 1H, m	−0.17; −0.08
40	1.14, 3H, s	1.14, 3H, s	0
41	1.08, 3H, s	1.07, 3H, s	+0.01
42	1.17, 3H, s	1.16, 3H, s	+0.01
43	1.72, 3H, s	1.47, 3H, s	+0.25

Table S7. Gibbs free energies [a] and equilibrium populations [b] of low-energy conformers of **1f**

Conformers	In MeOH	
	G	P (%)
**$1f_1$**	−2331.8990733	4.68%
**$1f_2$**	−2331.9018169	85.83%
**$1f_3$**	−2331.8987215	3.23%
**$1f_4$**	−2331.8993469	6.26%

[a] B3LYP/6-31+g (d, p), in kcal/mol; [b] from G values at 298.15K.

Table S8. Cartesian coordinates for the low-energy reoptimized MMFF conformers of **1f** at B3LYP/6-31+g (d, p) level of theory in MeOH

**$1f_1$**			Standard Orientation (Ångstroms)		
No.	Atom	Type	X	Y	Z
1	6	0	−2.30701	−0.26853	−0.06643
2	6	0	−1.59497	−1.67937	0.011689
3	6	0	7.307088	−1.25419	−1.61492
4	6	0	6.577708	−1.09903	−0.26478
5	6	0	5.07293	−0.79104	−0.47072
6	6	0	4.887612	0.372162	−1.47615
7	8	0	5.598273	0.100404	−2.69658
8	6	0	7.00131	−0.0386	−2.49042
9	6	0	3.431728	0.637073	−1.8533
10	6	0	2.833765	1.773732	−1.43637
11	6	0	1.499428	2.226148	−1.76437
12	6	0	0.925906	3.286658	−1.17475
13	6	0	−0.4111	3.73047	−1.49997
14	6	0	−1.03526	4.707811	−0.82611
15	6	0	−2.43409	5.164415	−1.12649
16	6	0	−3.36255	4.910654	0.061624
17	6	0	6.943883	−2.55534	−2.3415
18	8	0	6.688968	−2.27866	0.550435
19	8	0	4.387595	−0.60201	0.796305
20	6	0	−4.78219	5.368092	−0.23375
21	8	0	−0.3523	−1.61973	0.757401
22	6	0	−1.38709	0.829915	0.509381
23	6	0	7.965575	−2.43358	1.156315
24	6	0	1.112518	−0.76112	2.363238
25	6	0	1.523993	0.493949	2.537243
26	6	0	−0.33183	−0.91416	1.990936
27	6	0	−0.89027	0.511283	1.907461
28	6	0	0.323834	1.342743	2.271844
29	8	0	−0.9868	−1.61095	3.073625
30	6	0	−1.88223	−0.69308	3.589118

31	6	0	−1.91065	0.500129	3.002162
32	8	0	0.355875	2.566304	2.354818
33	6	0	−2.77117	1.662146	3.33811
34	6	0	−1.10635	−2.18999	−1.35862
35	1	0	−3.19929	−0.29772	0.571272
36	6	0	−2.4831	−2.77691	0.67795
37	6	0	−3.71617	−3.20225	−0.06762
38	6	0	−5.3475	0.069279	−1.32085
39	6	0	−4.06831	−0.32042	−2.04365
40	6	0	−2.75479	0.234481	−1.46854
41	6	0	−4.95787	−2.936	0.370752
42	6	0	−6.25922	−3.25098	−0.35626
43	6	0	−6.48898	−2.22348	−1.50237
44	6	0	−6.37902	−0.7729	−1.09118
45	6	0	−7.41889	−3.18692	0.660861
46	6	0	−6.25104	−4.67123	−0.95906
47	8	0	−4.18031	0.19382	−3.37557
48	6	0	−5.46188	1.510216	−0.88644
49	6	0	4.00702	0.358688	2.930247
50	6	0	4.735675	0.437682	1.597393
51	7	0	2.722677	1.042006	2.902699
52	8	0	5.525767	1.32544	1.299705
53	6	0	2.776955	−0.41884	−2.70942
54	1	0	8.388681	−1.28313	−1.43522
55	1	0	7.040052	−0.26963	0.28295
56	1	0	4.618279	−1.69955	−0.88195
57	1	0	5.312171	1.296067	−1.06718
58	1	0	7.471786	−0.1481	−3.47335
59	1	0	7.39842	0.88132	−2.04415
60	1	0	3.402752	2.455307	−0.80217
61	1	0	0.947295	1.68349	−2.52567
62	1	0	1.454346	3.834807	−0.39683
63	1	0	−0.92462	3.223137	−2.31456
64	1	0	−0.52985	5.208964	−0.00225
65	1	0	−2.82628	4.655819	−2.01575
66	1	0	−2.40866	6.235854	−1.35778
67	1	0	−2.99321	5.439079	0.94894
68	1	0	−3.37496	3.842357	0.307343
69	1	0	5.879127	−2.60974	−2.58749
70	1	0	7.503453	−2.63545	−3.28008
71	1	0	7.197218	−3.4309	−1.73555
72	1	0	−4.81244	6.442187	−0.44247
73	1	0	−5.43049	5.17018	0.625702
74	1	0	−5.19388	4.838197	−1.09854

75	1	0	−1.90703	1.795666	0.518146
76	1	0	−0.53105	0.949611	−0.16438
77	1	0	8.207101	−1.56382	1.774712
78	1	0	7.931265	−3.31644	1.800495
79	1	0	8.741481	−2.59044	0.402782
80	1	0	1.740906	−1.63025	2.489374
81	1	0	−2.47051	−1.05601	4.416633
82	1	0	−3.40812	1.928907	2.489284
83	1	0	−3.42522	1.440316	4.187813
84	1	0	−2.16323	2.531867	3.603791
85	1	0	−0.36687	−1.50304	−1.78678
86	1	0	−0.58687	−3.15034	−1.24896
87	1	0	−1.91117	−2.33205	−2.08281
88	1	0	−1.87098	−3.6724	0.853423
89	1	0	−2.77931	−2.44995	1.678359
90	1	0	−3.56372	−3.74023	−0.99926
91	1	0	−3.97237	−1.40075	−2.14522
92	1	0	−2.84897	1.32963	−1.44692
93	1	0	−1.95019	0.084535	−2.19948
94	1	0	−5.06079	−2.37076	1.298019
95	1	0	−5.80654	−2.43551	−2.33387
96	1	0	−7.49234	−2.36988	−1.92582
97	1	0	−7.25592	−0.39159	−0.56811
98	1	0	−8.38217	−3.37715	0.173715
99	1	0	−7.2939	−3.93706	1.451082
100	1	0	−7.48469	−2.20775	1.148926
101	1	0	−7.22373	−4.9138	−1.40288
102	1	0	−6.03781	−5.42711	−0.1942
103	1	0	−5.50171	−4.77743	−1.75125
104	1	0	−5.06694	−0.0508	−3.692
105	1	0	−6.46654	1.756019	−0.52546
106	1	0	−4.7629	1.72312	−0.07213
107	1	0	−5.24656	2.18251	−1.72323
108	1	0	4.647993	0.833442	3.681943
109	1	0	3.911177	−0.68905	3.237154
110	1	0	2.757952	2.053134	2.754502
111	1	0	2.848579	−1.40131	−2.23339
112	1	0	3.262483	−0.47327	−3.68902
113	1	0	1.713504	−0.24268	−2.88396

	**1f$_2$**		Standard Orientation (Ångstroms)		
No.	Atom	Type	X	Y	Z
1	6	0	3.002836	−1.60985	−0.78375
2	6	0	2.834866	−0.08376	−1.16806

3	6	0	−7.85167	−0.34681	1.334772
4	6	0	−6.94911	−0.69333	0.129844
5	6	0	−5.46998	−0.36168	0.434768
6	6	0	−5.34666	1.088019	0.96649
7	8	0	−6.19415	1.271176	2.111042
8	6	0	−7.57224	1.088776	1.787797
9	6	0	−3.92065	1.457523	1.362822
10	6	0	−3.26916	2.417424	0.671346
11	6	0	−1.89401	2.829088	0.845619
12	6	0	−1.28985	3.724222	0.048704
13	6	0	0.112946	4.048031	0.179087
14	6	0	0.74804	4.90911	−0.62974
15	6	0	2.215853	5.212447	−0.52122
16	6	0	2.958832	4.79786	−1.7916
17	6	0	−7.68229	−1.32475	2.505087
18	8	0	−7.0379	−2.08306	−0.22585
19	8	0	−4.595	−0.65569	−0.68557
20	6	0	4.456238	5.027558	−1.66091
21	8	0	1.469765	0.228098	−1.53955
22	6	0	1.704323	−2.39483	−1.09448
23	6	0	−8.22184	−2.41188	−0.94033
24	6	0	−0.84496	−0.0378	−1.50609
25	6	0	−1.55481	−1.13672	−1.75677
26	6	0	0.413843	−0.25645	−0.72044
27	6	0	0.478202	−1.76456	−0.45042
28	6	0	−0.80591	−2.24678	−1.09241
29	8	0	0.285121	0.435271	0.538547
30	6	0	0.284491	−0.56229	1.492953
31	6	0	0.400433	−1.80825	1.044228
32	8	0	−1.21545	−3.40293	−1.0816
33	6	0	0.435554	−3.06139	1.83905
34	6	0	3.59897	0.308703	−2.44836
35	1	0	3.154818	−1.67327	0.301014
36	6	0	3.233103	0.890325	−0.01464
37	6	0	4.688027	0.959995	0.350422
38	6	0	5.837315	−2.7286	0.512369
39	6	0	5.582312	−2.20634	−0.89246
40	6	0	4.163614	−2.3957	−1.45684
41	6	0	5.161772	0.510956	1.524414
42	6	0	6.624134	0.450577	1.946311
43	6	0	7.315273	−0.7729	1.277126
44	6	0	6.584751	−2.08713	1.437951
45	6	0	6.687126	0.326716	3.483717

46	6	0	7.391901	1.729802	1.553146
47	8	0	6.466175	−2.9246	−1.7606
48	6	0	5.2475	−4.07737	0.846326
49	6	0	−3.67627	−0.3595	−2.88312
50	6	0	−4.68408	0.076357	−1.82338
51	7	0	−2.71766	−1.37219	−2.44204
52	8	0	−5.49381	0.979391	−2.00799
53	6	0	−3.33787	0.671002	2.509543
54	1	0	−8.90115	−0.40875	1.022104
55	1	0	−7.27567	−0.10178	−0.73354
56	1	0	−5.14086	−1.05869	1.214891
57	1	0	−5.69035	1.799116	0.206498
58	1	0	−8.15664	1.327972	2.68269
59	1	0	−7.86266	1.809083	1.013237
60	1	0	−3.79809	2.922414	−0.13805
61	1	0	−1.31499	2.351133	1.6295
62	1	0	−1.84168	4.209254	−0.7529
63	1	0	0.670772	3.533567	0.959992
64	1	0	0.203354	5.424082	−1.41811
65	1	0	2.653468	4.701563	0.345423
66	1	0	2.339918	6.288498	−0.35158
67	1	0	2.581162	5.367351	−2.64954
68	1	0	2.777713	3.738221	−2.00666
69	1	0	−6.66539	−1.31559	2.908944
70	1	0	−8.36338	−1.06135	3.321896
71	1	0	−7.91874	−2.34968	2.202432
72	1	0	4.67647	6.083456	−1.47468
73	1	0	4.966847	4.730494	−2.58224
74	1	0	4.87279	4.438102	−0.83768
75	1	0	1.800865	−3.43485	−0.75599
76	1	0	1.570108	−2.44859	−2.18431
77	1	0	−9.10973	−2.25371	−0.32297
78	1	0	−8.29093	−1.8256	−1.86148
79	1	0	−8.17479	−3.47106	−1.20778
80	1	0	−1.11396	0.952943	−1.84082
81	1	0	0.196023	−0.21296	2.509091
82	1	0	1.380271	−3.59083	1.682995
83	1	0	0.343198	−2.85526	2.910503
84	1	0	−0.38679	−3.72462	1.55595
85	1	0	3.233106	−0.26386	−3.30896
86	1	0	3.41818	1.361732	−2.69758
87	1	0	4.679287	0.170118	−2.37152
88	1	0	2.9047	1.905089	−0.27557

89	1	0	2.66315	0.644078	0.885218
90	1	0	5.359175	1.387343	−0.38948
91	1	0	5.854636	−1.15601	−0.98851
92	1	0	3.94047	−3.4715	−1.41926
93	1	0	4.177594	−2.1971	−2.53591
94	1	0	4.452491	0.06535	2.223221
95	1	0	7.49156	−0.56478	0.215217
96	1	0	8.320141	−0.90063	1.702865
97	1	0	6.713087	−2.55015	2.416233
98	1	0	7.723735	0.249139	3.831338
99	1	0	6.239731	1.202113	3.969722
100	1	0	6.151296	−0.55743	3.847536
101	1	0	8.417542	1.705249	1.939894
102	1	0	6.906251	2.625714	1.957488
103	1	0	7.462004	1.84993	0.466398
104	1	0	7.339376	−2.91981	−1.33207
105	1	0	5.596435	−4.45343	1.814284
106	1	0	4.155723	−4.02094	0.894244
107	1	0	5.530935	−4.81775	0.091301
108	1	0	−3.14837	0.53036	−3.24189
109	1	0	−4.24937	−0.78013	−3.71597
110	1	0	−3.14064	−2.2623	−2.16396
111	1	0	−3.08758	−0.34407	2.185931
112	1	0	−4.05219	0.602283	3.336379
113	1	0	−2.43429	1.119923	2.927994

	**1f$_3$**		Standard Orientation (Ångstroms)		
No.	Atom	Type	X	Y	Z
1	6	0	−3.17301	−0.67833	−0.85887
2	6	0	−3.35083	−2.22301	−1.1568
3	6	0	7.675261	−0.9892	1.656543
4	6	0	6.832572	−1.27717	0.399147
5	6	0	6.067083	−0.01478	−0.07085
6	6	0	5.346686	0.68975	1.106877
7	8	0	6.232094	0.882105	2.222277
8	6	0	6.778844	−0.33865	2.709193
9	6	0	4.801571	2.067262	0.726293
10	6	0	3.481803	2.219504	0.490323
11	6	0	2.778438	3.446457	0.176692
12	6	0	1.478968	3.458614	−0.15963
13	6	0	0.740201	4.665313	−0.45233
14	6	0	−0.55458	4.650111	−0.80409
15	6	0	−1.35213	5.883972	−1.11615
16	6	0	−2.5256	6.041235	−0.14878

17	6	0	8.903564	−0.11871	1.364963
18	8	0	7.639061	−1.74619	−0.69644
19	8	0	5.1889	−0.29783	−1.19673
20	6	0	−3.34959	7.278133	−0.47012
21	8	0	−2.08887	−2.86025	−1.48494
22	6	0	−1.72782	−0.22815	−1.16768
23	6	0	8.042539	−3.10294	−0.56451
24	6	0	0.237165	−3.10883	−1.41069
25	6	0	1.178836	−2.20767	−1.69277
26	6	0	−0.95644	−2.58384	−0.67084
27	6	0	−0.68792	−1.08979	−0.46812
28	6	0	0.671021	−0.92536	−1.11554
29	8	0	−0.98845	−3.2371	0.615956
30	6	0	−0.77913	−2.22006	1.52718
31	6	0	−0.61721	−0.99912	1.024864
32	8	0	1.289787	0.131682	−1.1633
33	6	0	−0.38745	0.262651	1.771987
34	6	0	−4.17944	−2.50573	−2.42588
35	1	0	−3.32531	−0.5184	0.215819
36	6	0	−3.97471	−3.00873	0.040139
37	6	0	−5.3965	−2.68456	0.402516
38	6	0	−5.64575	1.175262	0.303417
39	6	0	−5.53063	0.508466	−1.05774
40	6	0	−4.10878	0.311303	−1.60983
41	6	0	−5.73016	−2.06834	1.548845
42	6	0	−7.12784	−1.62598	1.963639
43	6	0	−7.52359	−0.3294	1.199368
44	6	0	−6.50931	0.789583	1.268622
45	6	0	−7.13206	−1.37216	3.48647
46	6	0	−8.18331	−2.71339	1.673261
47	8	0	−6.21687	1.355179	−1.98628
48	6	0	−4.75992	2.374502	0.542275
49	6	0	3.457877	−1.37419	−2.38912
50	6	0	4.166521	−1.18646	−1.04704
51	7	0	2.377244	−2.37176	−2.34468
52	8	0	3.893331	−1.76887	−0.00436
53	6	0	5.818459	3.178918	0.655538
54	1	0	8.046957	−1.93807	2.062029
55	1	0	6.116419	−2.06733	0.647136
56	1	0	6.815921	0.667301	−0.49098
57	1	0	4.514178	0.073411	1.465989
58	1	0	7.35696	−0.10671	3.610084

59	1	0	5.966004	−1.01026	3.011185
60	1	0	2.846686	1.334517	0.548665
61	1	0	3.323913	4.382751	0.235109
62	1	0	0.931687	2.521009	−0.22935
63	1	0	1.272392	5.611	−0.37654
64	1	0	−1.08326	3.702638	−0.89014
65	1	0	−1.72566	5.804521	−2.14409
66	1	0	−0.71781	6.777896	−1.07594
67	1	0	−2.15619	6.113925	0.881371
68	1	0	−3.17392	5.158057	−0.19604
69	1	0	8.631334	0.862018	0.963122
70	1	0	9.478032	0.048114	2.282756
71	1	0	9.570632	−0.60555	0.646511
72	1	0	−2.74101	8.185044	−0.39682
73	1	0	−4.18353	7.370945	0.232626
74	1	0	−3.76294	7.221862	−1.48227
75	1	0	−1.58798	0.821491	−0.87667
76	1	0	−1.56921	−0.25741	−2.25538
77	1	0	8.699771	−3.23517	0.298625
78	1	0	7.170209	−3.75813	−0.48148
79	1	0	8.598814	−3.38112	−1.4638
80	1	0	0.299111	−4.15176	−1.68506
81	1	0	−0.7766	−2.53467	2.558602
82	1	0	−1.16702	0.995765	1.544328
83	1	0	−0.39804	0.092179	2.853532
84	1	0	0.583574	0.692704	1.514004
85	1	0	−3.68772	−2.08644	−3.31173
86	1	0	−4.24582	−3.58556	−2.60967
87	1	0	−5.1989	−2.11739	−2.37536
88	1	0	−3.91829	−4.08538	−0.17208
89	1	0	−3.35416	−2.87437	0.929667
90	1	0	−6.16196	−2.96762	−0.31483
91	1	0	−6.04736	−0.45018	−1.08574
92	1	0	−3.63471	1.302721	−1.64494
93	1	0	−4.16932	0.04475	−2.67239
94	1	0	−4.92493	−1.7803	2.226031
95	1	0	−7.75369	−0.5696	0.154771
96	1	0	−8.46783	0.05796	1.606542
97	1	0	−6.51766	1.339228	2.209821
98	1	0	−8.11356	−1.02138	3.825428
99	1	0	−6.8989	−2.28929	4.040927
100	1	0	−6.39342	−0.61757	3.779909

101	1	0	−9.16713	−2.41583	2.054713
102	1	0	−7.91663	−3.66425	2.149316
103	1	0	−8.29818	−2.89855	0.599513
104	1	0	−7.06786	1.584183	−1.57427
105	1	0	−3.70925	2.074576	0.602784
106	1	0	−4.87388	3.102223	−0.26767
107	1	0	−5.00491	2.890857	1.476838
108	1	0	4.204476	−1.73126	−3.10717
109	1	0	3.081016	−0.41146	−2.74704
110	1	0	2.707113	−3.33379	−2.33225
111	1	0	6.696532	2.866472	0.082188
112	1	0	6.147019	3.458243	1.661804
113	1	0	5.440935	4.080013	0.166548

	**1f$_4$**		Standard Orientation (Ångstroms)		
No.	Atom	Type	X	Y	Z
1	6	0	−3.45874	−0.75062	0.67773
2	6	0	−3.43494	0.630085	−0.09532
3	6	0	8.994198	−1.0538	1.077606
4	6	0	7.633688	−1.67753	0.713967
5	6	0	6.679907	−0.6396	0.084817
6	6	0	6.631587	0.630763	0.972302
7	8	0	7.942254	1.133322	1.258025
8	6	0	8.758183	0.186053	1.938852
9	6	0	5.824425	1.762488	0.34072
10	6	0	4.543856	1.961282	0.720306
11	6	0	3.6189	2.939171	0.189233
12	6	0	2.303508	2.897733	0.452196
13	6	0	1.359644	3.842485	−0.09957
14	6	0	0.038083	3.750068	0.110297
15	6	0	−0.97092	4.698067	−0.47093
16	6	0	−1.71139	5.460287	0.627852
17	6	0	9.836153	−0.70912	−0.15727
18	8	0	7.777014	−2.7717	−0.20768
19	8	0	5.360805	−1.24352	0.039675
20	6	0	−2.77448	6.378809	0.046291
21	8	0	−2.14369	0.882822	−0.70537
22	6	0	−2.04533	−1.37751	0.715579
23	6	0	8.236479	−3.97235	0.397989
24	6	0	−0.1073	0.266504	−1.67731
25	6	0	0.830439	−0.55719	−1.20934
26	6	0	−1.52118	−0.16544	−1.43797
27	6	0	−1.42046	−1.48659	−0.66737
28	6	0	0.080116	−1.68783	−0.57891

29	8	0	−2.12203	−0.41709	−2.72642
30	6	0	−2.39097	−1.77254	−2.72714
31	6	0	−2.05493	−2.44323	−1.62885
32	8	0	0.621813	−2.65607	−0.0554
33	6	0	−2.23715	−3.89098	−1.35656
34	6	0	−3.5829	1.850835	0.834507
35	1	0	−4.09281	−1.4462	0.113831
36	6	0	−4.51214	0.720845	−1.22236
37	6	0	−5.95063	0.749723	−0.78937
38	6	0	−6.2579	−1.97853	1.936864
39	6	0	−5.4978	−0.73414	2.366738
40	6	0	−3.97541	−0.743	2.144356
41	6	0	−6.80154	−0.26131	−1.03113
42	6	0	−8.24621	−0.35924	−0.55734
43	6	0	−8.28449	−0.74886	0.948941
44	6	0	−7.45873	−1.96059	1.31719
45	6	0	−8.97078	−1.42671	−1.40527
46	6	0	−9.00381	0.971849	−0.74378
47	8	0	−5.70422	−0.59666	3.776907
48	6	0	−5.62761	−3.30554	2.283953
49	6	0	3.163317	−1.44825	−0.83407
50	6	0	4.581647	−0.92126	−1.03094
51	7	0	2.189709	−0.44576	−1.26449
52	8	0	4.941393	−0.26909	−2.00493
53	6	0	6.539032	2.54503	−0.733
54	1	0	9.573781	−1.77486	1.666349
55	1	0	7.154567	−2.06325	1.625169
56	1	0	7.035872	−0.41733	−0.92818
57	1	0	6.16228	0.370016	1.932028
58	1	0	9.709496	0.674974	2.174689
59	1	0	8.287579	−0.0794	2.893342
60	1	0	4.124082	1.306237	1.484598
61	1	0	4.010121	3.718046	−0.45802
62	1	0	1.898496	2.109504	1.084015
63	1	0	1.755029	4.647379	−0.71566
64	1	0	−0.36369	2.93727	0.712599
65	1	0	−1.68367	4.11863	−1.07006
66	1	0	−0.48932	5.409527	−1.15283
67	1	0	−1.00137	6.057597	1.212664
68	1	0	−2.18729	4.75787	1.32275
69	1	0	9.342631	0.023045	−0.80372
70	1	0	10.80024	−0.28599	0.145463
71	1	0	10.04519	−1.60238	−0.75462
72	1	0	−2.32904	7.117781	−0.62727

73	1	0	−3.29017	6.916868	0.84781
74	1	0	−3.52142	5.807683	−0.51448
75	1	0	−2.08575	−2.37275	1.177635
76	1	0	−1.40254	−0.7719	1.370573
77	1	0	9.250172	−3.85194	0.788296
78	1	0	7.55743	−4.28586	1.196507
79	1	0	8.254905	−4.75376	−0.36654
80	1	0	0.108961	1.191172	−2.19329
81	1	0	−2.85756	−2.13657	−3.62864
82	1	0	−2.8827	−4.04142	−0.48599
83	1	0	−2.70224	−4.40026	−2.20709
84	1	0	−1.27506	−4.37459	−1.16477
85	1	0	−2.74868	1.902796	1.544028
86	1	0	−3.53791	2.781194	0.255239
87	1	0	−4.51936	1.861142	1.395783
88	1	0	−4.32357	1.620798	−1.82387
89	1	0	−4.38035	−0.10751	−1.92312
90	1	0	−6.28395	1.635323	−0.25527
91	1	0	−5.90786	0.167376	1.913391
92	1	0	−3.575609	−1.619809	2.673529
93	1	0	−3.526092	0.089693	2.699841
94	1	0	−6.414907	−1.140681	−1.548058
95	1	0	−7.991602	0.111179	1.562595
96	1	0	−9.322341	−0.956528	1.243992
97	1	0	−7.924255	−2.910931	1.056159
98	1	0	−10.008936	−1.55357	−1.077297
99	1	0	−8.990802	−1.143527	−2.464692
100	1	0	−8.482858	−2.405744	−1.339168
101	1	0	−10.062011	0.860432	−0.479465
102	1	0	−8.956211	1.314595	−1.784054
103	1	0	−8.597447	1.768478	−0.110976
104	1	0	−6.656354	−0.722913	3.930457
105	1	0	−4.718263	−3.467038	1.696861
106	1	0	−5.373111	−3.343152	3.348094
107	1	0	−6.298498	−4.147811	2.082542
108	1	0	3.013238	−1.66617	0.228768
109	1	0	3.061197	−2.370636	−1.414397
110	1	0	2.563886	0.301088	−1.851121
111	1	0	7.043158	1.871101	−1.432825
112	1	0	7.293104	3.199932	−0.284498
113	1	0	5.872904	3.168157	−1.333711

Table S9. Gibbs free energies [a] and equilibrium populations [b] of low-energy conformers of **1g**

Conformers	In MeOH	
	G	*P* (%)
**1g$_1$**	−2331.8921728	73.57%
**1g$_2$**	−2331.8911824	25.75%
**1g$_3$**	−2331.8877616	0.69%
**1g$_4$**	−2331.8783378	0.00%

[a] B3LYP/6-31+g (d, p), in kcal/mol; [b] from G values at 298.15K.

Table S10. Cartesian coordinates for the low-energy reoptimized MMFF conformers of **1g** at B3LYP/6-31+g (d, p) level of theory in MeOH

**1g$_1$**			Standard Orientation (Ångstroms)		
No.	Atom	Type	X	Y	Z
1	6	0	−2.2436	−1.71664	−0.61274
2	6	0	−2.75805	−3.002	0.111662
3	6	0	5.941541	0.57901	0.144066
4	6	0	4.88769	0.630224	1.265164
5	6	0	3.593545	1.343351	0.797203
6	6	0	3.960754	2.719341	0.183659
7	8	0	4.937108	2.55188	−0.85793
8	6	0	6.158886	1.991262	−0.39273
9	6	0	2.842244	3.56911	−0.43048
10	6	0	1.565217	3.151215	−0.5566
11	6	0	0.45979	3.869703	−1.16029
12	6	0	−0.78132	3.360954	−1.20903
13	6	0	−1.89967	4.049358	−1.81142
14	6	0	−3.12511	3.508656	−1.88846
15	6	0	−4.3105	4.203822	−2.49509
16	6	0	−5.48686	4.328503	−1.52303
17	6	0	5.55602	−0.38518	−0.98503
18	8	0	4.554088	−0.67919	1.748723
19	8	0	2.75232	1.551511	1.959474
20	6	0	−5.18421	5.212585	−0.32123
21	8	0	−2.45541	−2.91368	1.510435
22	6	0	−2.91068	−0.46163	−0.02988
23	6	0	5.560939	−1.27718	2.552109
24	6	0	−1.7334	−1.60209	3.254233
25	6	0	−1.01332	−0.48418	3.161571
26	6	0	−2.82325	−1.75342	2.2321
27	6	0	−2.8713	−0.40372	1.493086
28	6	0	−1.63859	0.290293	2.043179

29	8	0	−4.07651	−1.93157	2.933121
30	6	0	−4.71453	−0.71126	2.831289
31	6	0	−4.11869	0.205463	2.073647
32	8	0	−1.21731	1.374046	1.652268
33	6	0	−4.574	1.590775	1.788712
34	6	0	−4.26778	−3.23941	−0.06156
35	1	0	−1.17658	−1.619	−0.38198
36	6	0	−2.02134	−4.3136	−0.3461
37	6	0	−0.51881	−4.16757	−0.42021
38	6	0	−0.02508	−1.19572	−2.83725
39	6	0	−1.48733	−0.78363	−2.92848
40	6	0	−2.43723	−1.72867	−2.15038
41	6	0	0.207926	−4.50649	−1.49955
42	6	0	1.644804	−4.06973	−1.81106
43	6	0	1.661602	−3.139	−3.0846
44	6	0	0.367366	−2.39471	−3.31242
45	6	0	2.482385	−5.32978	−2.09903
46	6	0	2.320236	−3.32117	−0.6414
47	8	0	−1.88441	−0.80229	−4.30063
48	6	0	0.866612	−0.23368	−2.10741
49	6	0	1.03241	0.948898	3.466483
50	6	0	1.781386	0.611695	2.176089
51	7	0	0.091442	−0.10169	3.888055
52	8	0	1.548968	−0.34319	1.445467
53	6	0	3.326085	4.932478	−0.87439
54	1	0	6.892148	0.220978	0.557135
55	1	0	5.289108	1.202478	2.113893
56	1	0	3.086482	0.723469	0.048198
57	1	0	4.402135	3.333439	0.982812
58	1	0	6.861153	1.978153	−1.23301
59	1	0	6.589133	2.644907	0.375796
60	1	0	1.292927	2.160714	−0.19913
61	1	0	0.649701	4.842708	−1.60045
62	1	0	−0.98218	2.386003	−0.77037
63	1	0	−1.72164	5.047141	−2.20657
64	1	0	−3.30357	2.508281	−1.50167
65	1	0	−4.03537	5.19855	−2.86625
66	1	0	−4.63543	3.62224	−3.36629
67	1	0	−5.79368	3.335285	−1.17327
68	1	0	−6.34259	4.751422	−2.06276
69	1	0	4.623373	−0.09533	−1.47867
70	1	0	6.340269	−0.40853	−1.74959
71	1	0	5.436885	−1.40603	−0.60882
72	1	0	−4.39529	4.784008	0.304333

73	1	0	−6.07913	5.320959	0.299941
74	1	0	−4.86949	6.2117	−0.63897
75	1	0	−2.43019	0.430075	−0.4473
76	1	0	−3.94957	−0.41917	−0.38358
77	1	0	6.454661	−1.49648	1.962599
78	1	0	5.813636	−0.63318	3.399572
79	1	0	5.169393	−2.2215	2.940441
80	1	0	−1.53671	−2.38968	3.96693
81	1	0	−5.64003	−0.64084	3.379819
82	1	0	−3.83503	2.320497	2.13242
83	1	0	−5.51955	1.810682	2.295312
84	1	0	−4.73047	1.734544	0.715767
85	1	0	−4.58769	−4.11231	0.51979
86	1	0	−4.53146	−3.41194	−1.10974
87	1	0	−4.86771	−2.40166	0.302771
88	1	0	−2.23392	−5.1144	0.374759
89	1	0	−2.41432	−4.64422	−1.31517
90	1	0	−0.06018	−3.64486	0.416446
91	1	0	−1.61853	0.24689	−2.58297
92	1	0	−3.47342	−1.46092	−2.39829
93	1	0	−2.32461	−2.74343	−2.5471
94	1	0	−0.29729	−5.02521	−2.31501
95	1	0	2.513167	−2.44966	−3.05295
96	1	0	1.828202	−3.74959	−3.98288
97	1	0	−0.3786	−2.99112	−3.84151
98	1	0	3.512514	−5.06656	−2.36545
99	1	0	2.5218	−5.99034	−1.22478
100	1	0	2.063563	−5.90889	−2.93053
101	1	0	2.36885	−3.94465	0.259212
102	1	0	1.786056	−2.40196	−0.38394
103	1	0	3.348925	−3.04023	−0.89533
104	1	0	−1.14772	−0.41517	−4.80446
105	1	0	0.881745	0.726805	−2.63281
106	1	0	0.496585	−0.06574	−1.09229
107	1	0	1.901207	−0.57621	−2.03375
108	1	0	1.775765	1.061864	4.263396
109	1	0	0.513398	1.904912	3.349297
110	1	0	0.555627	−0.89992	4.316566
111	1	0	3.941703	4.843119	−1.77532
112	1	0	2.520894	5.63837	−1.08774
113	1	0	3.933238	5.392829	−0.08696

	**$1g_2$**		Standard Orientation (Ångstroms)		
No.	Atom	Type	X	Y	Z

1	6	0	4.363988	0.658727	−0.216087
2	6	0	4.621481	1.450688	−1.538549
3	6	0	−1.192356	−3.54313	2.242792
4	6	0	−1.132571	−2.004219	2.270624
5	6	0	−2.332877	−1.373258	1.529777
6	6	0	−2.518147	−2.012226	0.127883
7	8	0	−2.601525	−3.44366	0.258752
8	6	0	−1.412579	−4.010744	0.804395
9	6	0	−3.735916	−1.582528	−0.699477
10	6	0	−4.725652	−0.79791	−0.221232
11	6	0	−5.907308	−0.343699	−0.92692
12	6	0	−6.820023	0.463606	−0.36308
13	6	0	−8.003143	0.917644	−1.058858
14	6	0	−8.914439	1.724963	−0.495306
15	6	0	−10.14814	2.211319	−1.202125
16	6	0	−11.416702	1.719383	−0.504735
17	6	0	−2.273447	−4.112594	3.170338
18	8	0	−1.119458	−1.506454	3.618656
19	8	0	−2.229652	0.077382	1.518275
20	6	0	−12.667952	2.220518	−1.208861
21	8	0	3.534829	2.356818	−1.769421
22	6	0	4.225057	1.613814	0.979778
23	6	0	0.183386	−1.462918	4.18623
24	6	0	1.662968	3.416911	−0.960426
25	6	0	0.879385	2.973581	0.022845
26	6	0	3.137351	3.240004	−0.73875
27	6	0	3.268542	2.773364	0.722135
28	6	0	1.821409	2.474358	1.076439
29	8	0	3.768451	4.536042	−0.85916
30	6	0	3.99474	4.93685	0.443011
31	6	0	3.728014	4.041678	1.390231
32	8	0	1.457057	1.92769	2.112828
33	6	0	3.874708	4.197336	2.860062
34	6	0	5.943166	2.236518	−1.533464
35	1	0	3.387329	0.172734	−0.317077
36	6	0	4.620111	0.537544	−2.819129
37	6	0	3.464507	−0.436935	−2.858179
38	6	0	3.861893	−2.394428	0.462366
39	6	0	4.970527	−1.536307	1.05544
40	6	0	5.440044	−0.409884	0.102712
41	6	0	3.614067	−1.759645	−3.046771
42	6	0	2.582515	−2.852567	−2.742984
43	6	0	3.109172	−3.785713	−1.584393

44	6	0	4.091898	−3.111977	−0.655676
45	6	0	2.38172	−3.696444	−4.015472
46	6	0	1.202658	−2.289713	−2.336152
47	8	0	6.098144	−2.366118	1.337204
48	6	0	2.530142	−2.275684	1.141505
49	6	0	−1.286896	2.186643	1.049744
50	6	0	−1.22495	0.676643	0.819109
51	7	0	−0.49238	2.954354	0.084429
52	8	0	−0.412008	0.085662	0.119567
53	6	0	−3.68801	−2.124016	−2.111536
54	1	0	−0.233158	−3.94169	2.595635
55	1	0	−0.210116	−1.683539	1.776609
56	1	0	−3.224275	−1.580042	2.135293
57	1	0	−1.640386	−1.793144	−0.490336
58	1	0	−1.51822	−5.100301	0.768697
59	1	0	−0.559013	−3.75071	0.167481
60	1	0	−4.67816	−0.456452	0.811086
61	1	0	−6.060346	−0.677777	−1.947868
62	1	0	−6.689048	0.805284	0.661701
63	1	0	−8.144255	0.576819	−2.082781
64	1	0	−8.779061	2.072133	0.527082
65	1	0	−10.131195	3.307739	−1.21372
66	1	0	−10.151587	1.882982	−2.248621
67	1	0	−11.431214	2.061704	0.537228
68	1	0	−11.432219	0.622871	−0.483236
69	1	0	−3.276527	−3.773974	2.893784
70	1	0	−2.271342	−5.207436	3.130526
71	1	0	−2.092635	−3.824897	4.210977
72	1	0	−12.700961	3.314727	−1.218657
73	1	0	−13.563336	1.858233	−0.69412
74	1	0	−12.704331	1.865901	−2.243897
75	1	0	3.895964	1.050319	1.861773
76	1	0	5.221432	1.99627	1.239172
77	1	0	0.630617	−2.460164	4.215316
78	1	0	0.822584	−0.777613	3.621272
79	1	0	0.097859	−1.090758	5.210621
80	1	0	1.293977	3.843793	−1.881735
81	1	0	4.374815	5.940719	0.544953
82	1	0	2.909277	4.073798	3.359568
83	1	0	4.258952	5.18968	3.118351
84	1	0	4.571162	3.455361	3.26173
85	1	0	6.036164	2.836524	−2.446327
86	1	0	6.809076	1.569684	−1.473289

87	1	0	6.005799	2.943025	−0.701899
88	1	0	4.540756	1.171252	−3.712365
89	1	0	5.571049	−0.003319	−2.895022
90	1	0	2.495794	−0.018306	−2.594553
91	1	0	4.654464	−1.110587	2.013263
92	1	0	6.325117	0.070491	0.541688
93	1	0	5.816477	−0.867034	−0.818919
94	1	0	4.605647	−2.129684	−3.310582
95	1	0	2.273624	−4.221765	−1.024869
96	1	0	3.636052	−4.646939	−2.018193
97	1	0	5.114216	−3.123016	−1.040608
98	1	0	1.678342	−4.517986	−3.837345
99	1	0	1.984832	−3.089338	−4.837646
100	1	0	3.325344	−4.137533	−4.357688
101	1	0	0.775501	−1.663501	−3.128182
102	1	0	1.260996	−1.685119	−1.425634
103	1	0	0.488098	−3.09885	−2.145522
104	1	0	5.749873	−3.21185	1.667387
105	1	0	2.616398	−2.586861	2.187931
106	1	0	2.175339	−1.24112	1.119599
107	1	0	1.762673	−2.902727	0.683194
108	1	0	−2.33026	2.500524	0.932437
109	1	0	−0.982069	2.411278	2.076146
110	1	0	−0.930733	3.040945	−0.829625
111	1	0	−3.787339	−3.214353	−2.104018
112	1	0	−4.467064	−1.730144	−2.766425
113	1	0	−2.730842	−1.868948	−2.579882

	**1g$_3$**		Standard Orientation (Ångstroms)		
No.	Atom	Type	X	Y	Z
1	6	0	−3.176596	−0.507139	1.119609
2	6	0	−3.592654	−1.806553	1.882677
3	6	0	7.58026	−0.382678	−0.891548
4	6	0	6.217439	−0.951601	−1.328831
5	6	0	5.057025	0.018967	−0.988227
6	6	0	5.14076	0.490802	0.489765
7	8	0	6.458948	1.021225	0.73979
8	6	0	7.486697	0.050907	0.569228
9	6	0	4.161745	1.568354	0.972009
10	6	0	3.147044	2.055336	0.226787
11	6	0	2.180978	3.061258	0.623661
12	6	0	1.047748	3.2766	−0.06271
13	6	0	0.059593	4.254461	0.331958
14	6	0	−1.08925	4.433663	−0.337072

15	6	0	−2.145373	5.423556	0.064225
16	6	0	−2.39661	6.445848	−1.044204
17	6	0	8.053671	0.776248	−1.777867
18	8	0	6.175571	−1.22877	−2.740195
19	8	0	3.782478	−0.557272	−1.383496
20	6	0	−3.480745	7.436754	−0.650026
21	8	0	−2.533135	−2.771688	1.791733
22	6	0	−1.897273	0.089515	1.723261
23	6	0	6.882456	−2.405352	−3.110116
24	6	0	−0.337909	−3.221857	1.24092
25	6	0	0.365444	−2.53818	0.337557
26	6	0	−1.214258	−2.389202	2.136805
27	6	0	−0.781262	−0.936317	1.872606
28	6	0	0.059328	−1.099893	0.619562
29	8	0	−0.893596	−2.716347	3.508483
30	6	0	−0.073768	−1.686707	3.927129
31	6	0	0.07165	−0.669814	3.082073
32	8	0	0.47957	−0.175021	−0.066608
33	6	0	0.886982	0.556066	3.263398
34	6	0	−3.930477	−1.565654	3.36335
35	1	0	−2.910749	−0.807358	0.099904
36	6	0	−4.822286	−2.540341	1.233597
37	6	0	−4.707618	−2.68774	−0.266746
38	6	0	−4.229084	0.966052	−1.475764
39	6	0	−4.093174	1.602758	−0.098879
40	6	0	−4.269658	0.587992	1.059367
41	6	0	−5.660299	−2.290431	−1.128183
42	6	0	−5.499151	−2.063967	−2.636238
43	6	0	−5.700477	−0.537443	−2.981945
44	6	0	−5.396103	0.392599	−1.83242
45	6	0	−6.573617	−2.890549	−3.367193
46	6	0	−4.121847	−2.513367	−3.171682
47	8	0	−5.096381	2.60947	0.04547
48	6	0	−2.974539	0.943058	−2.302147
49	6	0	2.063299	−2.16866	−1.490511
50	6	0	3.307938	−1.668542	−0.761483
51	7	0	1.200137	−3.004185	−0.648377
52	8	0	3.845378	−2.229099	0.18606
53	6	0	4.416928	1.989713	2.404082
54	1	0	8.33916	−1.170743	−0.968107
55	1	0	6.053213	−1.896939	−0.798266
56	1	0	5.167507	0.896333	−1.638324

57	1	0	4.996838	−0.36656	1.158241
58	1	0	8.429107	0.504383	0.894199
59	1	0	7.297051	−0.805164	1.228081
60	1	0	2.989542	1.674816	−0.780099
61	1	0	2.38761	3.650301	1.51134
62	1	0	0.827265	2.675577	−0.943473
63	1	0	0.270804	4.852986	1.215592
64	1	0	−1.303884	3.830963	−1.216904
65	1	0	−3.070125	4.875063	0.279015
66	1	0	−1.861518	5.942469	0.987885
67	1	0	−2.697646	5.935136	−1.96702
68	1	0	−1.472844	6.993847	−1.266253
69	1	0	7.363268	1.624821	−1.75483
70	1	0	9.031768	1.137308	−1.4415
71	1	0	8.164418	0.457872	−2.819234
72	1	0	−4.427898	6.924145	−0.453897
73	1	0	−3.645749	8.158489	−1.456127
74	1	0	−3.19689	7.99156	0.249865
75	1	0	−1.550973	0.926135	1.103945
76	1	0	−2.139129	0.535768	2.697232
77	1	0	7.95286	−2.297615	−2.917508
78	1	0	6.486742	−3.275936	−2.578567
79	1	0	6.742312	−2.562818	−4.183051
80	1	0	−0.347171	−4.299932	1.310479
81	1	0	0.356123	−1.822422	4.906577
82	1	0	1.673926	0.596499	2.50765
83	1	0	1.367711	0.574838	4.246838
84	1	0	0.269232	1.454593	3.175726
85	1	0	−4.15376	−2.514786	3.864747
86	1	0	−4.797206	−0.907027	3.476925
87	1	0	−3.097392	−1.124774	3.916519
88	1	0	−4.899735	−3.552045	1.653423
89	1	0	−5.750474	−2.016028	1.491015
90	1	0	−3.739576	−3.029582	−0.625015
91	1	0	−3.129496	2.112078	−0.00714
92	1	0	−4.297614	1.142284	2.007435
93	1	0	−5.265793	0.138727	0.984062
94	1	0	−6.614777	−1.958457	−0.717464
95	1	0	−5.128288	−0.260595	−3.874792
96	1	0	−6.749777	−0.358189	−3.254466
97	1	0	−6.236064	0.500044	−1.142364
98	1	0	−6.522591	−2.734038	−4.450911

99	1	0	−6.448061	−3.963277	−3.178389
100	1	0	−7.58321	−2.614635	−3.040492
101	1	0	−3.952671	−3.582123	−2.994498
102	1	0	−3.300856	−1.961485	−2.702423
103	1	0	−4.048164	−2.35048	−4.253347
104	1	0	−5.163175	3.060726	−0.81332
105	1	0	−2.63763	1.967236	−2.493401
106	1	0	−2.174379	0.411099	−1.778909
107	1	0	−3.112896	0.463574	−3.274052
108	1	0	1.499705	−1.329624	−1.909036
109	1	0	2.410272	−2.775609	−2.334271
110	1	0	1.590552	−3.921258	−0.441675
111	1	0	5.292367	2.644619	2.458455
112	1	0	3.57579	2.513655	2.863783
113	1	0	4.603663	1.109752	3.029925

	**1g4**		Standard Orientation (Ångstroms)		
No.	Atom	Type	X	Y	Z
1	6	0	−3.164863	0.03168	0.820235
2	6	0	−3.687365	−0.738943	2.076477
3	6	0	7.554611	−0.595	−0.57177
4	6	0	6.225179	−1.366592	−0.668575
5	6	0	5.024122	−0.411505	−0.893167
6	6	0	5.044318	0.758358	0.129264
7	8	0	6.336772	1.39844	0.08399
8	6	0	7.399282	0.525756	0.452863
9	6	0	4.017336	1.887133	−0.023707
10	6	0	3.00215	1.870381	−0.913176
11	6	0	1.984711	2.888637	−1.089415
12	6	0	0.85055	2.658914	−1.770311
13	6	0	−0.213976	3.61817	−1.963421
14	6	0	−0.67774	4.442955	−1.011635
15	6	0	−1.823301	5.393365	−1.213047
16	6	0	−2.932248	5.142284	−0.190615
17	6	0	8.02085	−0.040704	−1.923862
18	8	0	6.238991	−2.33436	−1.733323
19	8	0	3.784091	−1.167305	−0.950961
20	6	0	−4.111744	6.076758	−0.406824
21	8	0	−2.657395	−1.611361	2.568274
22	6	0	−1.92173	0.859768	1.171788
23	6	0	6.993709	−3.5008	−1.432311
24	6	0	−0.440074	−2.25352	2.508931
25	6	0	0.342064	−2.106179	1.43893
26	6	0	−1.358449	−1.095107	2.794595

27	6	0	−0.855969	0.038835	1.884458
28	6	0	0.05001	−0.726236	0.937872
29	8	0	−1.160417	−0.693174	4.169453
30	6	0	−0.324479	0.403809	4.090323
31	6	0	−0.074133	0.864918	2.867803
32	8	0	0.525679	−0.273264	−0.096818
33	6	0	0.783163	2.012897	2.4849
34	6	0	−4.146392	0.187111	3.214806
35	1	0	−2.824527	−0.722841	0.102567
36	6	0	−4.877816	−1.713535	1.75177
37	6	0	−4.630338	−2.570563	0.5308
38	6	0	−3.921802	0.053447	−2.247591
39	6	0	−3.898161	1.279461	−1.344533
40	6	0	−4.210382	0.946721	0.137295
41	6	0	−5.481631	−2.652009	−0.506634
42	6	0	−5.170192	−3.182734	−1.911446
43	6	0	−5.288942	−2.020204	−2.972212
44	6	0	−5.066669	−0.643517	−2.394386
45	6	0	−6.195508	−4.28025	−2.252307
46	6	0	−3.762654	−3.808984	−2.023345
47	8	0	−4.885047	2.207167	−1.79969
48	6	0	−2.594823	−0.341409	−2.831395
49	6	0	2.128788	−2.690041	−0.242656
50	6	0	3.32189	−1.820723	0.147853
51	7	0	1.233383	−2.987942	0.880313
52	8	0	3.837335	−1.783379	1.258505
53	6	0	4.222386	3.002899	0.979828
54	1	0	8.339597	−1.275553	−0.220398
55	1	0	6.072448	−1.908231	0.272449
56	1	0	5.128316	0.009129	−1.901468
57	1	0	4.906244	0.362786	1.142835
58	1	0	8.31586	1.122733	0.50793
59	1	0	7.214679	0.125494	1.457227
60	1	0	2.880532	1.014296	−1.573085
61	1	0	2.150854	3.864086	−0.643955
62	1	0	0.681368	1.68725	−2.232387
63	1	0	−0.686647	3.596629	−2.943435
64	1	0	−0.221364	4.444611	−0.024164
65	1	0	−1.448332	6.418333	−1.106848
66	1	0	−2.228641	5.303208	−2.228126
67	1	0	−2.545238	5.28292	0.826101
68	1	0	−3.280184	4.105874	−0.2587
69	1	0	7.304062	0.666674	−2.351557
70	1	0	8.975637	0.484543	−1.810668

71	1	0	8.176057	−0.845834	−2.648971
72	1	0	−3.805373	7.123462	−0.313111
73	1	0	−4.890393	5.882252	0.33733
74	1	0	−4.548565	5.93326	−1.400336
75	1	0	−1.500569	1.300901	0.260517
76	1	0	−2.225478	1.715273	1.789985
77	1	0	8.053309	−3.262439	−1.310042
78	1	0	6.607831	−3.987924	−0.531797
79	1	0	6.89417	−4.195848	−2.270518
80	1	0	−0.482207	−3.150925	3.108851
81	1	0	0.029429	0.77057	5.040404
82	1	0	1.616015	1.668976	1.868075
83	1	0	1.203286	2.50966	3.365524
84	1	0	0.211665	2.756647	1.922645
85	1	0	−4.447027	−0.401248	4.089798
86	1	0	−4.995655	0.807742	2.9123
87	1	0	−3.349627	0.850506	3.560859
88	1	0	−5.027709	−2.393872	2.600536
89	1	0	−5.806951	−1.143798	1.631013
90	1	0	−3.646945	−3.031918	0.48202
91	1	0	−2.934279	1.791787	−1.421666
92	1	0	−4.305018	1.888485	0.694542
93	1	0	−5.210023	0.502507	0.193772
94	1	0	−6.457097	−2.174196	−0.406238
95	1	0	−4.625347	−2.201093	−3.825434
96	1	0	−6.301098	−2.015742	−3.399989
97	1	0	−5.964613	−0.231022	−1.928657
98	1	0	−6.036456	−4.668354	−3.265019
99	1	0	−6.122434	−5.124073	−1.555948
100	1	0	−7.222543	−3.899554	−2.201628
101	1	0	−3.644501	−4.654309	−1.335163
102	1	0	−2.973697	−3.082827	−1.801755
103	1	0	−3.580878	−4.189684	−3.035299
104	1	0	−4.848469	2.196145	−2.771407
105	1	0	−2.201048	0.475726	−3.445182
106	1	0	−1.870292	−0.554422	−2.039857
107	1	0	−2.657392	−1.224158	−3.472332
108	1	0	1.571215	−2.229423	−1.064074
109	1	0	2.53326	−3.635694	−0.620512
110	1	0	1.60472	−3.664812	1.543085
111	1	0	5.068665	3.630959	0.683591
112	1	0	3.350149	3.649183	1.101718
113	1	0	4.431058	2.586458	1.971727

Table S11. Gibbs free energies [a] and equilibrium populations [b] of low-energy conformers of **1h**

Conformers	In MeOH	
	G	*P* (%)
**1h1**	−2331.8887971	0.02%
**1h2**	−2331.8881096	0.01%
**1h3**	−2331.893428	3.33%
**1h4**	−2331.8966049	96.63%

[a] B3LYP/6-31+g (d, p), in kcal/mol; [b] from G values at 298.15K.

Table S12. Cartesian coordinates for the low-energy reoptimized MMFF conformers of **1h** at B3LYP/6-31+g (d, p) level of theory in MeOH

**1h1**			Standard Orientation (Ångstroms)		
No.	Atom	Type	X	Y	Z
1	6	0	3.030459	0.608821	1.079357
2	6	0	2.999188	2.175176	1.259691
3	6	0	−1.289117	−5.093611	0.116503
4	6	0	−0.657983	−4.113001	−0.88687
5	6	0	−1.507279	−2.831131	−1.049852
6	6	0	−2.974242	−3.235244	−1.353451
7	8	0	−3.476848	−4.144697	−0.364344
8	6	0	−2.734431	−5.359113	−0.297785
9	6	0	−4.020234	−2.122817	−1.474406
10	6	0	−3.858566	−0.893773	−0.942223
11	6	0	−4.757825	0.238184	−1.03099
12	6	0	−4.432014	1.445826	−0.543432
13	6	0	−5.309606	2.591473	−0.614543
14	6	0	−4.975086	3.791514	−0.116192
15	6	0	−5.873033	4.9959	−0.166813
16	6	0	−5.339066	6.110525	−1.070502
17	6	0	−1.22104	−4.600952	1.567217
18	8	0	0.672867	−3.79384	−0.447359
19	8	0	−0.981774	−2.088669	−2.179975
20	6	0	−4.054285	6.746851	−0.558916
21	8	0	3.581267	2.893973	0.146984
22	6	0	4.161359	0.214265	0.106094
23	6	0	1.633439	−3.890879	−1.498628
24	6	0	2.238409	2.461336	−1.81272
25	6	0	1.77329	1.329644	−2.344087
26	6	0	3.576066	2.342959	−1.159333
27	6	0	3.982606	0.867526	−1.256107
28	6	0	2.821574	0.291867	−2.055473

29	8	0	4.50391	3.109306	−1.979291
30	6	0	5.436661	2.196978	−2.422995
31	6	0	5.229605	0.929632	−2.081812
32	8	0	2.737921	−0.871856	−2.434042
33	6	0	6.050724	−0.2565	−2.430917
34	6	0	3.88169	2.644395	2.437223
35	1	0	2.080571	0.322137	0.613987
36	6	0	1.552046	2.75182	1.444305
37	6	0	0.716712	2.167545	2.551041
38	6	0	1.22805	−1.658753	3.079493
39	6	0	2.600926	−1.646212	2.42177
40	6	0	3.190365	−0.207928	2.402286
41	6	0	−0.465509	1.559392	2.339058
42	6	0	−1.268192	0.726909	3.34566
43	6	0	−1.198934	−0.790485	2.942559
44	6	0	0.156841	−1.187336	2.415571
45	6	0	−2.738051	1.185457	3.302094
46	6	0	−0.778125	0.897477	4.799747
47	8	0	2.561979	−2.199827	1.119641
48	6	0	1.220005	−2.081423	4.521312
49	6	0	−0.013503	−0.203333	−3.235742
50	6	0	−0.535176	−0.821331	−1.940728
51	7	0	0.593947	1.118502	−3.021963
52	8	0	−0.595427	−0.240815	−0.86326
53	6	0	−5.240927	−2.559171	−2.25135
54	1	0	−0.728298	−6.03644	0.07843
55	1	0	−0.602773	−4.619354	−1.861824
56	1	0	−1.43863	−2.258796	−0.119795
57	1	0	−2.967122	−3.751141	−2.325594
58	1	0	−3.228908	−6.012455	0.429028
59	1	0	−2.781231	−5.865521	−1.269447
60	1	0	−2.965003	−0.689751	−0.356576
61	1	0	−5.722676	0.10161	−1.507531
62	1	0	−3.46319	1.597336	−0.070513
63	1	0	−6.276125	2.455193	−1.095258
64	1	0	−4.013497	3.92559	0.37547
65	1	0	−6.86975	4.708444	−0.523895
66	1	0	−6.008344	5.375904	0.852945
67	1	0	−6.105164	6.891642	−1.147074
68	1	0	−5.175388	5.727257	−2.084931
69	1	0	−1.772255	−3.667544	1.712225
70	1	0	−1.654605	−5.346848	2.24246
71	1	0	−0.185486	−4.440581	1.882797
72	1	0	−3.771246	7.588039	−1.199893

73	1	0	−3.223792	6.034602	−0.560904
74	1	0	−4.182801	7.126264	0.459778
75	1	0	5.124685	0.513495	0.541868
76	1	0	4.212466	−0.872546	−0.016967
77	1	0	2.611169	−3.594055	−1.112532
78	1	0	1.370419	−3.236042	−2.332533
79	1	0	1.702658	−4.926723	−1.845138
80	1	0	1.728678	3.411947	−1.866866
81	1	0	6.229686	2.617507	−3.019683
82	1	0	5.461015	−0.983905	−2.996531
83	1	0	6.429778	−0.743594	−1.527504
84	1	0	6.912922	0.023277	−3.045268
85	1	0	3.511949	2.329176	3.415448
86	1	0	3.953951	3.739248	2.453653
87	1	0	4.90807	2.277622	2.3206
88	1	0	1.62242	3.835999	1.607509
89	1	0	1.007297	2.654283	0.499114
90	1	0	1.148358	2.201719	3.546213
91	1	0	3.271294	−2.286489	3.007628
92	1	0	2.770712	0.337152	3.253462
93	1	0	4.264027	−0.291091	2.621431
94	1	0	−0.851711	1.527798	1.318182
95	1	0	−1.508988	−1.42809	3.777765
96	1	0	−1.921968	−0.993116	2.140722
97	1	0	0.297918	−0.913758	1.369397
98	1	0	−3.354449	0.60146	3.995274
99	1	0	−2.834899	2.242389	3.576598
100	1	0	−3.16657	1.063947	2.301765
101	1	0	0.262517	0.585202	4.92757
102	1	0	−1.383626	0.299192	5.49067
103	1	0	−0.852228	1.942539	5.123575
104	1	0	1.776117	−2.775803	1.041465
105	1	0	0.227032	−2.041625	4.975293
106	1	0	1.573215	−3.114314	4.608599
107	1	0	1.882387	−1.439081	5.110577
108	1	0	0.692654	−0.883192	−3.720175
109	1	0	−0.870342	−0.074753	−3.906023
110	1	0	−0.09555	1.842031	−2.826555
111	1	0	−4.9589	−2.816719	−3.277893
112	1	0	−6.024629	−1.802222	−2.312526
113	1	0	−5.689361	−3.443444	−1.78585

	**1h$_2$**		Standard Orientation (Ångstroms)		
No.	Atom	Type	X	Y	Z

1	6	0	3.613208	−1.251613	0.102983
2	6	0	3.534226	−1.362016	−1.47211
3	6	0	−8.770408	−0.058371	−0.341015
4	6	0	−7.459068	−0.819285	−0.047036
5	6	0	−6.401274	0.12082	0.575265
6	6	0	−6.256782	1.40985	−0.271123
7	8	0	−7.532876	2.044314	−0.440627
8	6	0	−8.456173	1.209673	−1.138929
9	6	0	−5.293316	2.420451	0.350255
10	6	0	−4.102639	2.647642	−0.245383
11	6	0	−3.042362	3.521947	0.209189
12	6	0	−1.879735	3.647938	−0.450349
13	6	0	−0.808212	4.505403	0.003509
14	6	0	0.354896	4.628204	−0.65397
15	6	0	1.478798	5.515899	−0.198525
16	6	0	2.779099	4.750303	0.057949
17	6	0	−9.557422	0.284218	0.930865
18	8	0	−7.670521	−1.919386	0.853346
19	8	0	−5.137487	−0.54949	0.827344
20	6	0	2.680949	3.769974	1.2184
21	8	0	2.17294	−1.529856	−1.923241
22	6	0	2.234166	−1.458299	0.773578
23	6	0	−8.233495	−3.063962	0.225813
24	6	0	0.000119	−2.349486	−1.777572
25	6	0	−0.879182	−2.202262	−0.787631
26	6	0	1.415438	−2.585522	−1.347885
27	6	0	1.392293	−2.584683	0.185924
28	6	0	−0.083846	−2.361862	0.471176
29	8	0	1.798849	−3.899026	−1.811209
30	6	0	1.982097	−4.635819	−0.656722
31	6	0	1.789767	−3.994474	0.492649
32	8	0	−0.579466	−2.31591	1.592442
33	6	0	1.895013	−4.542863	1.869258
34	6	0	4.39454	−2.468779	−2.112264
35	1	0	3.843737	−0.206992	0.328832
36	6	0	3.939241	0.010837	−2.129737
37	6	0	5.311985	0.534734	−1.787809
38	6	0	6.412521	−0.413611	1.751391
39	6	0	5.376871	−1.489349	2.065287
40	6	0	4.745377	−2.085056	0.779298
41	6	0	5.523077	1.66584	−1.090306
42	6	0	6.854714	2.165326	−0.523295
43	6	0	6.871587	2.028668	1.043954

44	6	0	6.056805	0.875557	1.58038
45	6	0	7.001067	3.65652	−0.890288
46	6	0	8.075835	1.430739	−1.117133
47	8	0	4.379477	−0.98486	2.940532
48	6	0	7.814776	−0.931515	1.579398
49	6	0	−3.06002	−1.541298	0.282028
50	6	0	−4.388498	−0.983301	−0.218474
51	7	0	−2.223453	−1.961424	−0.84612
52	8	0	−4.709487	−0.906716	−1.39979
53	6	0	−5.770035	3.08098	1.619289
54	1	0	−9.421305	−0.695522	−0.952076
55	1	0	−7.070801	−1.221332	−0.989886
56	1	0	−6.761802	0.387194	1.576038
57	1	0	−5.895626	1.16253	−1.276147
58	1	0	−9.368537	1.792394	−1.305251
59	1	0	−8.048715	0.962602	−2.126839
60	1	0	−3.879856	2.10993	−1.168519
61	1	0	−3.195148	4.085129	1.124254
62	1	0	−1.708013	3.087751	−1.36739
63	1	0	−0.969935	5.065005	0.92276
64	1	0	0.522389	4.075446	−1.576275
65	1	0	1.659212	6.261787	−0.982171
66	1	0	1.198553	6.073107	0.703734
67	1	0	3.572923	5.474256	0.277945
68	1	0	3.083945	4.212739	−0.847685
69	1	0	−9.825139	−0.620164	1.486346
70	1	0	−8.995593	0.941156	1.601546
71	1	0	−10.491165	0.796386	0.673714
72	1	0	2.349446	4.274156	2.13169
73	1	0	3.660212	3.32391	1.416451
74	1	0	1.982602	2.956563	0.998534
75	1	0	2.328204	−1.592008	1.855563
76	1	0	1.679054	−0.514875	0.658467
77	1	0	−8.321459	−3.854732	0.975848
78	1	0	−7.582768	−3.420736	−0.578084
79	1	0	−9.231802	−2.847239	−0.162932
80	1	0	−0.257437	−2.2997	−2.82543
81	1	0	2.262693	−5.663984	−0.820319
82	1	0	0.927359	−4.501399	2.377847
83	1	0	2.620739	−3.97838	2.461451
84	1	0	2.219128	−5.588753	1.85594
85	1	0	5.464263	−2.244071	−2.088595
86	1	0	4.111306	−2.618842	−3.16078

87	1	0	4.26258	−3.431577	−1.613666
88	1	0	3.869646	−0.079855	−3.222554
89	1	0	3.176353	0.757684	−1.866301
90	1	0	6.148063	−0.08916	−2.087069
91	1	0	5.874144	−2.301995	2.609084
92	1	0	5.569263	−2.260414	0.082077
93	1	0	4.35013	−3.074864	1.026927
94	1	0	4.652653	2.251476	−0.794727
95	1	0	7.901155	1.988498	1.418281
96	1	0	6.447182	2.937578	1.493303
97	1	0	5.008322	1.136478	1.73485
98	1	0	7.928266	4.074288	−0.481463
99	1	0	7.020375	3.799945	−1.977153
100	1	0	6.168957	4.251421	−0.495009
101	1	0	8.059827	0.358691	−0.902004
102	1	0	9.011904	1.830607	−0.709914
103	1	0	8.119628	1.548269	−2.206498
104	1	0	4.844387	−0.497802	3.642401
105	1	0	8.547908	−0.138284	1.415872
106	1	0	8.128483	−1.469962	2.480211
107	1	0	7.867658	−1.620252	0.730456
108	1	0	−2.560749	−0.742947	0.841572
109	1	0	−3.263224	−2.398857	0.93065
110	1	0	−2.602861	−1.735444	−1.765567
111	1	0	−5.862151	2.344317	2.423116
112	1	0	−6.747601	3.549617	1.466423
113	1	0	−5.104221	3.868066	1.978885

	**1h$_3$**		Standard Orientation (Ångstroms)		
No.	Atom	Type	X	Y	Z
1	6	0	−4.110897	−0.398052	0.220524
2	6	0	−3.835255	−0.635584	−1.31846
3	6	0	8.532623	−2.440773	0.728539
4	6	0	7.097718	−2.666968	0.203336
5	6	0	6.540371	−1.382714	−0.452092
6	6	0	6.724491	−0.171015	0.495723
7	8	0	8.103302	−0.040691	0.873449
8	6	0	8.566828	−1.180502	1.597289
9	6	0	6.264217	1.147015	−0.126616
10	6	0	5.12113	1.71753	0.312436
11	6	0	4.512949	2.946715	−0.1501
12	6	0	3.335095	3.389156	0.319096
13	6	0	2.72819	4.623763	−0.125394
14	6	0	1.555583	5.072238	0.347789

15	6	0	0.925743	6.366947	−0.082435
16	6	0	−0.419755	6.132922	−0.768241
17	6	0	9.570456	−2.348422	−0.398048
18	8	0	7.042643	−3.727744	−0.765341
19	8	0	5.178642	−1.547665	−0.929546
20	6	0	−1.062935	7.443522	−1.193788
21	8	0	−2.451964	−0.97114	−1.563016
22	6	0	−2.816914	−0.476765	1.065675
23	6	0	7.079803	−5.02509	−0.184652
24	6	0	−0.154384	−0.806038	−1.20155
25	6	0	0.521425	−0.995074	−0.069998
26	6	0	−1.462956	−0.091311	−1.047203
27	6	0	−1.603681	0.209944	0.451832
28	6	0	−0.312657	−0.370682	0.999201
29	8	0	−1.358747	1.156525	−1.768109
30	6	0	−1.413925	2.130444	−0.789726
31	6	0	−1.558212	1.706106	0.462626
32	8	0	0.051292	−0.327588	2.169798
33	6	0	−1.625517	2.525437	1.699778
34	6	0	−4.223125	0.523765	−2.257022
35	1	0	−4.686938	−1.259999	0.568175
36	6	0	−4.588266	−1.93061	−1.806972
37	6	0	−6.085223	−1.940641	−1.619611
38	6	0	−7.192241	−0.014928	1.500181
39	6	0	−5.901962	0.739531	1.806938
40	6	0	−4.980697	0.850912	0.563158
41	6	0	−6.730522	−2.793036	−0.802206
42	6	0	−8.20642	−2.735245	−0.395585
43	6	0	−8.344088	−2.294518	1.108416
44	6	0	−7.258755	−1.358884	1.58678
45	6	0	−8.803426	−4.148774	−0.553224
46	6	0	−9.041983	−1.790415	−1.286448
47	8	0	−5.219611	0.142918	2.899437
48	6	0	−8.324998	0.856955	1.029775
49	6	0	2.82825	−1.704248	−0.750859
50	6	0	4.172797	−1.70725	−0.030984
51	7	0	1.726994	−1.580177	0.204558
52	8	0	4.317099	−1.804073	1.181892
53	6	0	7.153129	1.700721	−1.210995
54	1	0	8.821032	−3.295276	1.3529
55	1	0	6.456199	−2.944571	1.047785
56	1	0	7.120131	−1.21329	−1.367068

57	1	0	6.161699	−0.326099	1.423553
58	1	0	9.590347	−0.97008	1.925566
59	1	0	7.959938	−1.309211	2.501884
60	1	0	4.569413	1.214686	1.109172
61	1	0	5.037968	3.53123	−0.898794
62	1	0	2.79556	2.816245	1.071435
63	1	0	3.266419	5.205177	−0.871437
64	1	0	1.0167	4.499583	1.10003
65	1	0	0.785927	6.995072	0.805661
66	1	0	1.592375	6.917427	−0.757458
67	1	0	−1.101349	5.604822	−0.090987
68	1	0	−0.284888	5.497095	−1.651702
69	1	0	9.584548	−3.26278	−0.999558
70	1	0	9.380945	−1.50399	−1.067623
71	1	0	10.574764	−2.218575	0.020033
72	1	0	−1.242577	8.090969	−0.329492
73	1	0	−2.024279	7.253038	−1.681187
74	1	0	−0.424441	7.982565	−1.900886
75	1	0	−2.976667	−0.106916	2.083094
76	1	0	−2.582884	−1.544649	1.19263
77	1	0	6.992345	−5.76058	−0.989027
78	1	0	6.239645	−5.162954	0.502619
79	1	0	8.026211	−5.195724	0.334723
80	1	0	0.183947	−1.135617	−2.17247
81	1	0	−1.331238	3.139645	−1.158765
82	1	0	−0.773392	2.31279	2.351837
83	1	0	−2.545258	2.321733	2.255257
84	1	0	−1.608533	3.595448	1.468822
85	1	0	−5.304615	0.63545	−2.37258
86	1	0	−3.798137	0.369352	−3.255885
87	1	0	−3.843073	1.484322	−1.902315
88	1	0	−4.375512	−2.088726	−2.873229
89	1	0	−4.141074	−2.800129	−1.304418
90	1	0	−6.633898	−1.16578	−2.14532
91	1	0	−6.162131	1.754801	2.130511
92	1	0	−5.626112	1.118871	−0.277895
93	1	0	−4.317316	1.708232	0.712915
94	1	0	−6.14064	−3.547137	−0.279158
95	1	0	−9.337749	−1.872714	1.299256
96	1	0	−8.289059	−3.182094	1.754419
97	1	0	−6.3742	−1.888923	1.94485
98	1	0	−9.855027	−4.169706	−0.244751

99	1	0	−8.753673	−4.48904	−1.594379
100	1	0	−8.265713	−4.882911	0.058564
101	1	0	−8.692541	−0.755528	−1.235712
102	1	0	−10.095596	−1.793473	−0.983342
103	1	0	−9.006069	−2.099973	−2.337788
104	1	0	−5.888871	−0.038084	3.581633
105	1	0	−9.25423	0.304754	0.871173
106	1	0	−8.539754	1.62783	1.777573
107	1	0	−8.062545	1.352533	0.089918
108	1	0	2.821164	−0.852427	−1.440466
109	1	0	2.726892	−2.638369	−1.311324
110	1	0	2.028	−1.423363	1.174288
111	1	0	7.261766	0.978627	−2.025784
112	1	0	8.148135	1.924341	−0.812824
113	1	0	6.775699	2.622458	−1.658389

	**1h$_4$**		Standard Orientation (Ångstroms)		
No.	Atom	Type	X	Y	Z
1	6	0	3.231596	−0.897444	−0.072014
2	6	0	3.011864	0.62745	0.285448
3	6	0	−5.75099	−3.171597	−1.397704
4	6	0	−4.908462	−2.881409	−0.142592
5	6	0	−4.96403	−1.390533	0.25078
6	6	0	−4.643094	−0.520642	−0.986241
7	8	0	−5.481266	−0.85487	−2.099807
8	6	0	−5.331246	−2.211179	−2.509766
9	6	0	−4.79064	0.976368	−0.72451
10	6	0	−3.68453	1.736759	−0.582113
11	6	0	−3.617241	3.1574	−0.316239
12	6	0	−2.451127	3.805196	−0.166186
13	6	0	−2.366306	5.221766	0.106454
14	6	0	−1.197363	5.861399	0.263636
15	6	0	−1.083126	7.331928	0.551297
16	6	0	−0.301209	8.090793	−0.523774
17	6	0	−7.258725	−3.075561	−1.133345
18	8	0	−5.351093	−3.662244	0.980806
19	8	0	−3.942627	−1.192065	1.256194
20	6	0	−1.008584	8.120401	−1.871517
21	8	0	1.79948	0.823487	1.045013
22	6	0	2.046798	−1.782772	0.383862
23	6	0	−4.831983	−4.985848	0.982581
24	6	0	−0.4211	0.393274	1.607611
25	6	0	−0.968052	−0.764403	1.972261
26	6	0	0.59526	0.296077	0.50887

27	6	0	0.666473	−1.190719	0.131436
28	6	0	−0.364869	−1.789503	1.068953
29	8	0	0.086047	1.039343	−0.620998
30	6	0	−0.138895	0.087065	−1.59651
31	6	0	0.153717	−1.169685	−1.275166
32	8	0	−0.718782	−2.964297	1.089567
33	6	0	−0.012131	−2.38432	−2.114012
34	6	0	2.994365	1.602104	−0.908389
35	1	0	4.05669	−1.246244	0.554947
36	6	0	4.123348	1.110372	1.292318
37	6	0	5.550935	0.964586	0.827116
38	6	0	6.061633	−2.080322	−1.439032
39	6	0	4.606319	−2.405645	−1.761741
40	6	0	3.672273	−1.186485	−1.54046
41	6	0	6.447431	0.147989	1.410717
42	6	0	7.83861	−0.215791	0.882066
43	6	0	7.860947	−1.705684	0.377172
44	6	0	6.547031	−2.194111	−0.186153
45	6	0	8.845562	−0.071564	2.041559
46	6	0	8.31766	0.717938	−0.250686
47	8	0	4.166069	−3.531582	−1.017469
48	6	0	6.855501	−1.559856	−2.606701
49	6	0	−3.054212	−0.249504	3.266932
50	6	0	−4.232711	−0.366222	2.29809
51	7	0	−1.910396	−1.106365	2.911614
52	8	0	−5.297627	0.217326	2.460943
53	6	0	−6.20894	1.481698	−0.634133
54	1	0	−5.552978	−4.196828	−1.733095
55	1	0	−3.857103	−3.134498	−0.343785
56	1	0	−5.958243	−1.177183	0.659837
57	1	0	−3.603363	−0.715634	−1.287356
58	1	0	−5.952365	−2.360186	−3.39954
59	1	0	−4.291	−2.385994	−2.810601
60	1	0	−2.714675	1.248214	−0.664572
61	1	0	−4.547216	3.711056	−0.23443
62	1	0	−1.510712	3.263709	−0.248668
63	1	0	−3.300216	5.775213	0.182012
64	1	0	−0.260781	5.311347	0.19388
65	1	0	−2.073689	7.786928	0.674098
66	1	0	−0.56523	7.448677	1.51106
67	1	0	0.695963	7.651345	−0.647166
68	1	0	−0.152467	9.12291	−0.184392
69	1	0	−7.55863	−2.075616	−0.805116
70	1	0	−7.820569	−3.306253	−2.045135

71	1	0	−7.571547	−3.792383	−0.367374
72	1	0	−1.103654	7.116899	−2.297265
73	1	0	−0.440258	8.730789	−2.580542
74	1	0	−2.009869	8.552996	−1.77924
75	1	0	2.103678	−2.7867	−0.04784
76	1	0	2.163453	−1.939936	1.466829
77	1	0	−5.165377	−5.539104	0.100707
78	1	0	−5.206881	−5.49781	1.872964
79	1	0	−3.738786	−4.968494	1.028263
80	1	0	−0.654672	1.34478	2.062523
81	1	0	−0.536294	0.476927	−2.52044
82	1	0	−0.736852	−3.069298	−1.664251
83	1	0	0.937727	−2.913478	−2.230375
84	1	0	−0.37476	−2.128275	−3.11509
85	1	0	3.983128	1.753171	−1.350017
86	1	0	2.617003	2.583932	−0.598521
87	1	0	2.33956	1.256677	−1.71133
88	1	0	3.949479	2.166735	1.53934
89	1	0	3.982285	0.57772	2.243706
90	1	0	5.818049	1.521547	−0.065318
91	1	0	4.542008	−2.697039	−2.817308
92	1	0	4.184422	−0.321281	−1.970599
93	1	0	2.776519	−1.334899	−2.151139
94	1	0	6.135054	−0.406506	2.29671
95	1	0	8.676333	−1.859928	−0.33918
96	1	0	8.099252	−2.37173	1.218483
97	1	0	5.880268	−2.587952	0.583106
98	1	0	9.856628	−0.350726	1.723241
99	1	0	8.88429	0.960363	2.410322
100	1	0	8.577699	−0.713566	2.889127
101	1	0	7.662181	0.679783	−1.125084
102	1	0	9.324751	0.444466	−0.58653
103	1	0	8.359893	1.761092	0.085062
104	1	0	4.876141	−4.194576	−1.068707
105	1	0	7.907489	−1.387507	−2.36776
106	1	0	6.833173	−2.284616	−3.427628
107	1	0	6.432801	−0.617193	−2.967944
108	1	0	−2.748768	0.800258	3.321385
109	1	0	−3.412291	−0.56511	4.252163
110	1	0	−2.203177	−2.079004	2.763844
111	1	0	−6.283199	2.514977	−0.289169
112	1	0	−6.692651	1.429347	−1.614927
113	1	0	−6.793032	0.878851	0.067514

Table S13. Gibbs free energies [a] and equilibrium populations [b] of low-energy conformers of **1i**

Conformers	In MeOH	
	G	*P* (%)
**1i$_1$**	−2331.9030703	67.64%
**1i$_2$**	−2331.8963299	0.05%
**1i$_3$**	−2331.8960539	0.04%
**1i$_4$**	−2331.9023723	32.27%

[a] B3LYP/6-31+g (d, p), in kcal/mol; [b] from G values at 298.15K

Table S14. Cartesian coordinates for the low-energy reoptimized MMFF conformers of **1i** at B3LYP/6-31+g (d, p) level of theory in MeOH

**1i$_1$**			Standard Orientation (Ångstroms)		
No.	Atom	Type	X	Y	Z
1	6	0	−3.271735	−1.365477	−0.453684
2	6	0	−2.669781	−0.125518	−1.173855
3	6	0	7.864742	−0.57983	1.370437
4	6	0	6.956263	−1.036002	0.209788
5	6	0	6.079396	0.131519	−0.303445
6	6	0	5.374785	0.858217	0.869507
7	8	0	6.327423	1.242765	1.872617
8	6	0	7.01116	0.121934	2.428488
9	6	0	4.638306	2.122076	0.423658
10	6	0	3.293775	2.190726	0.535575
11	6	0	2.443883	3.295886	0.142112
12	6	0	1.112376	3.288692	0.311962
13	6	0	0.267958	4.399272	−0.066969
14	6	0	−1.064483	4.389675	0.091401
15	6	0	−1.951808	5.548732	−0.267196
16	6	0	−3.011352	5.200334	−1.3146
17	6	0	9.013401	0.32561	0.908082
18	8	0	7.714673	−1.547742	−0.899877
19	8	0	5.161836	−0.275678	−1.354419
20	6	0	−2.421195	4.837143	−2.669992
21	8	0	−1.514973	0.333038	−0.458474
22	6	0	−2.253884	−2.510225	−0.420313
23	6	0	8.204872	−2.86582	−0.689751
24	6	0	0.616965	−0.589252	−0.961586
25	6	0	1.086013	−1.799452	−1.262036
26	6	0	−0.537587	−0.59137	−0.010959
27	6	0	−0.927274	−2.076205	0.19803
28	6	0	0.227396	−2.766606	−0.508485

29	8	0	−0.024319	−0.094345	1.257895
30	6	0	−0.321691	−1.076138	2.174641
31	6	0	−0.822218	−2.206656	1.687718
32	8	0	0.451434	−3.972411	−0.505863
33	6	0	−1.172993	−3.432587	2.449337
34	6	0	−2.277864	−0.395401	−2.637921
35	1	0	−3.426452	−1.074104	0.594121
36	6	0	−3.637053	1.108612	−1.182398
37	6	0	−4.375011	1.326479	0.119407
38	6	0	−6.958843	−2.092089	−0.04436
39	6	0	−5.500793	−2.507223	0.058634
40	6	0	−4.621108	−1.862398	−1.034652
41	6	0	−5.707257	1.180274	0.219724
42	6	0	−6.564868	1.155814	1.478698
43	6	0	−6.907049	−0.328637	1.838858
44	6	0	−7.541939	−1.15265	0.735393
45	6	0	−7.852157	1.958585	1.204104
46	6	0	−5.864321	1.79425	2.69622
47	8	0	−5.402904	−3.925455	−0.046258
48	6	0	−7.754771	−2.755884	−1.142515
49	6	0	3.324793	−1.407428	−2.310917
50	6	0	4.186172	−1.179819	−1.069471
51	7	0	2.139831	−2.227755	−2.032674
52	8	0	4.033064	−1.726263	0.015284
53	6	0	5.513849	3.227647	−0.114417
54	1	0	8.323989	−1.46299	1.830982
55	1	0	6.311881	−1.841626	0.577156
56	1	0	6.756951	0.833256	−0.804713
57	1	0	4.655932	0.186829	1.353307
58	1	0	7.642149	0.490442	3.244368
59	1	0	6.282603	−0.568023	2.871392
60	1	0	2.76394	1.335982	0.960249
61	1	0	2.910138	4.166041	−0.309055
62	1	0	0.621017	2.429442	0.763698
63	1	0	0.753975	5.27688	−0.488001
64	1	0	−1.556556	3.515437	0.514638
65	1	0	−2.458914	5.880057	0.647155
66	1	0	−1.358485	6.401411	−0.619658
67	1	0	−3.673223	6.065361	−1.442375
68	1	0	−3.638782	4.376148	−0.956327
69	1	0	8.652973	1.250516	0.447743
70	1	0	9.643685	0.604906	1.759581
71	1	0	9.653747	−0.186943	0.183306
72	1	0	−3.223479	4.655231	−3.392173

73	1	0	−1.811459	3.930172	−2.614132
74	1	0	−1.79656	5.649349	−3.055114
75	1	0	−2.670733	−3.362216	0.129649
76	1	0	−2.097943	−2.893445	−1.437152
77	1	0	7.3788	−3.55851	−0.503112
78	1	0	8.72419	−3.184287	−1.597752
79	1	0	8.916747	−2.892378	0.139213
80	1	0	1.035286	0.337886	−1.324157
81	1	0	−0.077366	−0.828762	3.195205
82	1	0	−2.248925	−3.624706	2.401598
83	1	0	−0.901367	−3.335809	3.505849
84	1	0	−0.644283	−4.302951	2.049982
85	1	0	−1.829757	0.499961	−3.085179
86	1	0	−3.146019	−0.680694	−3.240878
87	1	0	−1.528381	−1.184001	−2.736646
88	1	0	−3.059827	2.018365	−1.387234
89	1	0	−4.35795	1.01277	−2.004265
90	1	0	−3.755705	1.518721	0.991875
91	1	0	−5.113045	−2.268584	1.051154
92	1	0	−4.434907	−2.575883	−1.84778
93	1	0	−5.160217	−1.027873	−1.488802
94	1	0	−6.258728	0.966466	−0.697798
95	1	0	−6.002918	−0.821964	2.211684
96	1	0	−7.602026	−0.339678	2.690043
97	1	0	−8.592859	−0.919204	0.563089
98	1	0	−8.528431	1.926142	2.065971
99	1	0	−7.624673	3.012015	1.000693
100	1	0	−8.40252	1.572155	0.338818
101	1	0	−6.531737	1.81489	3.565804
102	1	0	−5.562419	2.82733	2.487608
103	1	0	−4.969077	1.234559	2.990253
104	1	0	−6.002336	−4.292748	0.626065
105	1	0	−8.760929	−2.333406	−1.236857
106	1	0	−7.25608	−2.627943	−2.108578
107	1	0	−7.866534	−3.826666	−0.94492
108	1	0	3.93709	−1.924529	−3.056704
109	1	0	3.01778	−0.438086	−2.718177
110	1	0	2.375969	−3.194177	−1.783381
111	1	0	5.864757	2.978411	−1.120515
112	1	0	5.008699	4.194353	−0.169339
113	1	0	6.388174	3.380832	0.526481

	**1i2**		Standard Orientation (Ångstroms)		
No.	Atom	Type	X	Y	Z

1	6	0	3.07273	−0.730019	0.256886
2	6	0	2.616505	−0.466821	−1.206067
3	6	0	−8.68077	0.174683	−0.396242
4	6	0	−7.457774	−0.711845	−0.073677
5	6	0	−6.316706	0.125747	0.54768
6	6	0	−6.028455	1.373932	−0.322761
7	8	0	−7.229904	2.136281	−0.512519
8	6	0	−8.229908	1.392061	−1.20774
9	6	0	−4.968569	2.294256	0.280755
10	6	0	−3.796872	2.470873	−0.367498
11	6	0	−2.663217	3.260378	0.066912
12	6	0	−1.556986	3.407635	−0.679457
13	6	0	−0.416035	4.185633	−0.250325
14	6	0	0.678165	4.351611	−1.008712
15	6	0	1.877218	5.152681	−0.58515
16	6	0	3.116285	4.265683	−0.461481
17	6	0	−9.443769	0.615513	0.859936
18	8	0	−7.790182	−1.769518	0.840738
19	8	0	−5.134882	−0.667152	0.834034
20	6	0	4.345883	5.070217	−0.072043
21	8	0	2.247816	−1.707559	−1.822793
22	6	0	1.92943	−1.339972	1.073123
23	6	0	−8.465894	−2.858441	0.225457
24	6	0	0.001749	−2.453829	−1.621389
25	6	0	−0.920223	−2.42199	−0.659586
26	6	0	1.406532	−2.62383	−1.138756
27	6	0	1.33904	−2.582332	0.407716
28	6	0	−0.166854	−2.606571	0.625019
29	8	0	1.819313	−3.964461	−1.523609
30	6	0	2.203778	−4.570311	−0.34911
31	6	0	1.978067	−3.889013	0.769475
32	8	0	−0.713979	−2.722549	1.716566
33	6	0	2.246289	−4.336424	2.160156
34	6	0	1.438422	0.518821	−1.310203
35	1	0	3.850955	−1.503891	0.208902
36	6	0	3.762991	0.100827	−2.116593
37	6	0	5.120885	−0.520668	−1.876795
38	6	0	5.902712	1.062789	2.02631
39	6	0	4.714877	0.115505	2.026506
40	6	0	3.645879	0.512584	0.98684
41	6	0	6.148883	0.179349	−1.365952
42	6	0	7.50214	−0.333694	−0.889372
43	6	0	7.49132	−0.451491	0.67103

44	6	0	7.089037	0.796241	1.433203
45	6	0	8.5864	0.661399	−1.349526
46	6	0	7.858386	−1.717718	−1.4709
47	8	0	4.119708	0.101321	3.321433
48	6	0	5.677087	2.399162	2.691425
49	6	0	−3.156246	−1.866439	0.346625
50	6	0	−4.412566	−1.188183	−0.190124
51	7	0	−2.273612	−2.255993	−0.758695
52	8	0	−4.7009	−1.099778	−1.37891
53	6	0	−5.337764	2.943211	1.590759
54	1	0	−9.386125	−0.402123	−1.006725
55	1	0	−7.100687	−1.167003	−1.00476
56	1	0	−6.661959	0.450669	1.536593
57	1	0	−5.692076	1.066923	−1.319803
58	1	0	−9.075306	2.064136	−1.390562
59	1	0	−7.842679	1.089802	−2.188502
60	1	0	−3.655716	1.960944	−1.321869
61	1	0	−2.715009	3.74131	1.038899
62	1	0	−1.491482	2.933576	−1.656726
63	1	0	−0.46552	4.646708	0.734437
64	1	0	0.729869	3.899811	−1.997379
65	1	0	2.051865	5.936297	−1.332107
66	1	0	1.689619	5.659687	0.369195
67	1	0	3.311056	3.753053	−1.411386
68	1	0	2.943754	3.490476	0.294025
69	1	0	−8.826168	1.223453	1.527922
70	1	0	−10.317945	1.215131	0.582818
71	1	0	−9.808037	−0.247921	1.425545
72	1	0	5.218741	4.414023	0.004212
73	1	0	4.203425	5.559987	0.89651
74	1	0	4.564026	5.840154	−0.818848
75	1	0	2.282706	−1.579125	2.08313
76	1	0	1.150222	−0.582622	1.231463
77	1	0	−7.851498	−3.292846	−0.56886
78	1	0	−8.639983	−3.624546	0.985924
79	1	0	−9.43408	−2.544559	−0.173281
80	1	0	−0.218557	−2.373169	−2.675732
81	1	0	2.618001	−5.558643	−0.46734
82	1	0	3.011752	−3.710884	2.628772
83	1	0	2.606133	−5.37064	2.182688
84	1	0	1.336879	−4.289414	2.766279
85	1	0	1.131443	0.642613	−2.355483
86	1	0	1.707018	1.501492	−0.913415

87	1	0	0.549185	0.17875	−0.775142
88	1	0	3.508755	−0.067952	−3.171637
89	1	0	3.831981	1.188865	−1.990532
90	1	0	5.195722	−1.589069	−2.063104
91	1	0	5.050982	−0.90908	1.855255
92	1	0	2.831647	1.069251	1.468355
93	1	0	4.085818	1.198665	0.26005
94	1	0	5.989933	1.244383	−1.186237
95	1	0	6.856956	−1.296426	0.960025
96	1	0	8.497147	−0.728758	1.015946
97	1	0	7.869586	1.556197	1.475382
98	1	0	9.576331	0.361409	−0.986968
99	1	0	8.636081	0.71328	−2.443797
100	1	0	8.395837	1.676852	−0.984167
101	1	0	8.862542	−2.02729	−1.158283
102	1	0	7.843044	−1.707032	−2.566968
103	1	0	7.161955	−2.493072	−1.131698
104	1	0	4.832779	−0.108166	3.949031
105	1	0	6.518849	3.083527	2.539994
106	1	0	4.785159	2.885559	2.284137
107	1	0	5.544694	2.274466	3.770695
108	1	0	−3.449666	−2.752673	0.91681
109	1	0	−2.64982	−1.142259	0.994207
110	1	0	−2.63108	−2.021345	−1.685297
111	1	0	−5.327783	2.204188	2.397765
112	1	0	−4.664982	3.754689	1.87717
113	1	0	−6.339406	3.382229	1.537692

	**1i$_3$**		Standard Orientation (Ångstroms)		
No.	Atom	Type	X	Y	Z
1	6	0	3.165378	−0.50796	0.078859
2	6	0	2.234777	−1.163191	−0.980725
3	6	0	−0.953023	4.562777	−0.301182
4	6	0	−0.971072	3.300721	0.588081
5	6	0	−1.88324	2.208603	−0.015868
6	6	0	−3.26424	2.793046	−0.398308
7	8	0	−3.110118	3.937206	−1.250696
8	6	0	−2.389643	4.988613	−0.609235
9	6	0	−4.152071	1.779899	−1.121295
10	6	0	−5.275363	1.334909	−0.518344
11	6	0	−6.212325	0.354134	−1.023877
12	6	0	−7.303453	−0.022578	−0.338521
13	6	0	−8.24131	−1.006588	−0.833799
14	6	0	−9.328891	−1.383591	−0.143445

15	6	0	−10.368464	−2.392262	−0.559255
16	6	0	−10.16885	−3.067591	−1.919023
17	6	0	−0.156263	4.363946	−1.597923
18	8	0	0.35001	2.75594	0.740297
19	8	0	−1.977	1.024573	0.819624
20	6	0	−11.284361	−4.059382	−2.21449
21	8	0	1.734432	−2.410737	−0.480089
22	6	0	2.393253	−0.230963	1.370311
23	6	0	1.112498	3.413666	1.746878
24	6	0	−0.258061	−2.443247	0.814599
25	6	0	−0.809195	−1.651932	1.735851
26	6	0	1.23521	−2.503506	0.84493
27	6	0	1.684024	−1.470572	1.905067
28	6	0	0.342033	−1.070612	2.50298
29	8	0	1.58408	−3.824777	1.346655
30	6	0	2.366605	−3.592606	2.454616
31	6	0	2.480377	−2.32693	2.842949
32	8	0	0.206191	−0.329883	3.471237
33	6	0	3.215751	−1.815538	4.027745
34	6	0	1.044687	−0.273856	−1.384676
35	1	0	3.924276	−1.257623	0.340757
36	6	0	2.989598	−1.545864	−2.303237
37	6	0	4.366389	−2.132864	−2.081652
38	6	0	6.336721	1.410166	−0.644749
39	6	0	5.238467	0.97095	0.309306
40	6	0	3.880665	0.782763	−0.400248
41	6	0	5.487824	−1.490135	−2.450924
42	6	0	6.932741	−1.83549	−2.112066
43	6	0	7.422705	−0.910786	−0.947604
44	6	0	7.26749	0.581165	−1.170648
45	6	0	7.791615	−1.621079	−3.374259
46	6	0	7.112398	−3.301367	−1.665512
47	8	0	5.093085	1.942344	1.343699
48	6	0	6.309149	2.863538	−1.0533
49	6	0	−2.659903	−0.256469	2.738531
50	6	0	−2.560597	1.102766	2.044598
51	7	0	−2.13975	−1.388949	1.958091
52	8	0	−3.013173	2.135111	2.529065
53	6	0	−3.674037	1.350767	−2.486132
54	1	0	−0.462841	5.376313	0.247456
55	1	0	−1.347226	3.577402	1.579355
56	1	0	−1.384161	1.850335	−0.92403

57	1	0	−3.790177	3.137892	0.499421
58	1	0	−2.395949	5.852635	−1.282407
59	1	0	−2.917461	5.288973	0.304173
60	1	0	−5.521923	1.734172	0.466848
61	1	0	−6.015663	−0.094402	−1.992545
62	1	0	−7.51649	0.417301	0.633799
63	1	0	−8.029592	−1.439207	−1.808413
64	1	0	−9.516896	−0.932028	0.830253
65	1	0	−10.411079	−3.164773	0.218768
66	1	0	−11.34247	−1.886876	−0.552641
67	1	0	−9.209634	−3.598509	−1.939963
68	1	0	−10.146122	−2.31426	−2.715331
69	1	0	−0.57799	3.573604	−2.226428
70	1	0	−0.152116	5.28752	−2.187234
71	1	0	0.887049	4.108676	−1.386428
72	1	0	−11.123837	−4.53076	−3.189135
73	1	0	−12.258274	−3.559868	−2.235102
74	1	0	−11.318442	−4.848831	−1.456879
75	1	0	3.068419	0.183362	2.128029
76	1	0	1.665279	0.565703	1.188287
77	1	0	0.635536	3.293249	2.724088
78	1	0	2.102359	2.951962	1.786054
79	1	0	1.23795	4.474342	1.514336
80	1	0	−0.821689	−3.030438	0.104621
81	1	0	2.776758	−4.479621	2.909719
82	1	0	4.053487	−1.18218	3.721643
83	1	0	3.624415	−2.636457	4.62658
84	1	0	2.553928	−1.232048	4.674212
85	1	0	0.405626	−0.793448	−2.108595
86	1	0	1.376948	0.666943	−1.83539
87	1	0	0.398315	−0.029613	−0.539509
88	1	0	2.402487	−2.289865	−2.858224
89	1	0	3.064937	−0.666862	−2.956005
90	1	0	4.401481	−3.063131	−1.520023
91	1	0	5.532561	0.052467	0.821808
92	1	0	3.233921	1.652449	−0.227822
93	1	0	4.037793	0.740422	−1.480352
94	1	0	5.37104	−0.549706	−2.992734
95	1	0	6.923597	−1.212226	−0.020369
96	1	0	8.489693	−1.099837	−0.764902
97	1	0	7.999645	0.999767	−1.861461
98	1	0	8.851818	−1.804886	−3.165572

99	1	0	7.489017	−2.302684	−4.178269
100	1	0	7.704826	−0.600479	−3.763909
101	1	0	8.170005	−3.53015	−1.488696
102	1	0	6.74722	−3.999484	−2.427643
103	1	0	6.577169	−3.510961	−0.732301
104	1	0	5.975438	2.054011	1.737624
105	1	0	7.049084	3.088989	−1.8288
106	1	0	5.326022	3.130646	−1.453888
107	1	0	6.525671	3.509401	−0.196563
108	1	0	−3.726619	−0.444077	2.904914
109	1	0	−2.178324	−0.197833	3.717878
110	1	0	−2.732525	−1.641927	1.170486
111	1	0	−2.784988	0.71871	−2.397395
112	1	0	−4.420343	0.788677	−3.051471
113	1	0	−3.4187	2.220727	−3.099669

	**1i4**		Standard Orientation (Ångstroms)		
No.	Atom	Type	X	Y	Z
1	6	0	−3.956604	−1.137547	0.028442
2	6	0	−3.62593	−1.301458	−1.48242
3	6	0	8.637733	−2.396765	0.465917
4	6	0	7.111044	−2.530323	0.636332
5	6	0	6.364547	−1.370587	−0.055075
6	6	0	6.97172	−0.024993	0.404854
7	8	0	8.386324	0.013803	0.187507
8	6	0	9.074971	−1.001081	0.913536
9	6	0	6.34176	1.17689	−0.295507
10	6	0	5.427181	1.920227	0.363808
11	6	0	4.690229	3.059601	−0.138662
12	6	0	3.738687	3.672956	0.582865
13	6	0	2.977047	4.797751	0.089237
14	6	0	2.022905	5.401515	0.814151
15	6	0	1.2179	6.572308	0.323662
16	6	0	−0.267198	6.248761	0.138382
17	6	0	9.099816	−2.675628	−0.970105
18	8	0	6.617992	−3.767774	0.097134
19	8	0	4.976065	−1.42325	0.357784
20	6	0	−0.991857	5.946702	1.442744
21	8	0	−2.895765	−0.15849	−1.946128
22	6	0	−2.667799	−1.032124	0.849179
23	6	0	6.873717	−4.894002	0.925041
24	6	0	−0.528048	−0.090453	−1.753046
25	6	0	0.368058	−0.49999	−0.855128
26	6	0	−1.827702	0.373549	−1.179187

27	6	0	−1.754581	0.080327	0.339517
28	6	0	−0.299528	−0.343221	0.479369
29	8	0	−1.858213	1.819282	−1.344283
30	6	0	−2.039569	2.321048	−0.07626
31	6	0	−1.994815	1.442505	0.919565
32	8	0	0.264281	−0.559621	1.547357
33	6	0	−2.106233	1.73046	2.372309
34	6	0	−2.812941	−2.570465	−1.797152
35	1	0	−4.458778	−0.166527	0.136168
36	6	0	−4.906769	−1.3422	−2.38931
37	6	0	−5.982885	−0.357854	−1.987179
38	6	0	−7.180035	−2.188151	1.703476
39	6	0	−5.744355	−1.694899	1.768974
40	6	0	−4.876194	−2.24072	0.614583
41	6	0	−7.172643	−0.754216	−1.503033
42	6	0	−8.268554	0.094134	−0.869479
43	6	0	−8.190642	−0.042527	0.68787
44	6	0	−8.217103	−1.45654	1.235148
45	6	0	−9.630755	−0.406066	−1.390305
46	6	0	−8.151446	1.591089	−1.22405
47	8	0	−5.164097	−2.087902	3.010439
48	6	0	−7.405525	−3.61462	2.144045
49	6	0	2.658446	−1.104084	−0.004808
50	6	0	4.048685	−1.220021	−0.621283
51	7	0	1.640921	−0.959737	−1.050815
52	8	0	4.284358	−1.097454	−1.818439
53	6	0	6.79045	1.407282	−1.716664
54	1	0	9.135545	−3.133779	1.107441
55	1	0	6.858251	−2.502233	1.705734
56	1	0	6.458874	−1.505163	−1.138625
57	1	0	6.801575	0.083164	1.48604
58	1	0	10.147829	−0.862132	0.742663
59	1	0	8.900003	−0.859833	1.987178
60	1	0	5.181716	1.64086	1.389278
61	1	0	4.91349	3.418714	−1.138453
62	1	0	3.50108	3.321059	1.584773
63	1	0	3.196306	5.149485	−0.916969
64	1	0	1.807629	5.054435	1.822399
65	1	0	1.332608	7.399617	1.034291
66	1	0	1.616415	6.929758	−0.634033
67	1	0	−0.385331	5.401768	−0.548126
68	1	0	−0.753187	7.110235	−0.335429
69	1	0	8.671668	−1.968715	−1.687424
70	1	0	10.190309	−2.597321	−1.040673

71	1	0	8.824777	−3.687261	−1.284828
72	1	0	−2.061677	5.809878	1.254789
73	1	0	−0.876566	6.769411	2.155555
74	1	0	−0.617374	5.030238	1.908159
75	1	0	−2.912018	−0.871243	1.905812
76	1	0	−2.144101	−1.996837	0.83572
77	1	0	6.430382	−4.753379	1.915277
78	1	0	6.411994	−5.769705	0.46071
79	1	0	7.94712	−5.080112	1.013656
80	1	0	−0.345553	−0.056529	−2.817225
81	1	0	−2.16458	3.390526	−0.030665
82	1	0	−3.005307	1.270016	2.792063
83	1	0	−2.168914	2.807629	2.560284
84	1	0	−1.233912	1.350791	2.911961
85	1	0	−2.583981	−2.622278	−2.868374
86	1	0	−3.359128	−3.47799	−1.520411
87	1	0	−1.847584	−2.590765	−1.285971
88	1	0	−4.628215	−1.114995	−3.427095
89	1	0	−5.322484	−2.357884	−2.399978
90	1	0	−5.705808	0.692625	−2.028119
91	1	0	−5.723596	−0.603191	1.779033
92	1	0	−4.267737	−3.086789	0.959379
93	1	0	−5.52372	−2.640099	−0.169371
94	1	0	−7.369585	−1.82751	−1.474613
95	1	0	−7.303613	0.489736	1.048056
96	1	0	−9.038508	0.492909	1.13733
97	1	0	−9.205421	−1.916235	1.215025
98	1	0	−10.456977	0.140577	−0.921537
99	1	0	−9.714181	−0.266654	−2.474882
100	1	0	−9.783905	−1.472039	−1.187018
101	1	0	−8.98821	2.160237	−0.802304
102	1	0	−8.160456	1.747093	−2.309189
103	1	0	−7.229641	2.032122	−0.827982
104	1	0	−5.7634	−1.767941	3.706676
105	1	0	−8.435046	−3.944071	1.9667
106	1	0	−6.747002	−4.294612	1.594179
107	1	0	−7.206808	−3.723914	3.214784
108	1	0	2.666144	−0.219057	0.640309
109	1	0	2.455609	−2.006203	0.580276
110	1	0	2.029948	−0.803774	−1.9829
111	1	0	6.710527	0.486829	−2.302836
112	1	0	6.202505	2.161733	−2.243261
113	1	0	7.834869	1.735302	−1.734805

Table S15. Boltzmann population [a] and optical rotation (OR) of low-energy conformers of 11*R*-**2**

Conformers	In MeOH	
	Boltzmann population (%)	OR
11*R*-**2a**	9.70	737.8
11*R*-**2b**	62.47	596.17
11*R*-**2c**	16.36	389.8
11*R*-**2d**	10.18	1258.46
11*R*-**2e**	1.29	870.52
Average		697.42

[a] From Δ*G* values at 298.15K.

Table S16. Cartesian coordinates for the low-energy reoptimized MMFF conformers of 11*R*-**2** at B3LYP/6-311+G (d, p) level of theory in MeOH

11*R*-**2a**		Standard Orientation a(Ångstroms)			
No.	Atom	Type	X	Y	Z
1	6	0	−1.0253	2.763	−3.1885
2	6	0	−1.4965	4.0728	−3.1721
3	6	0	−1.4929	4.823	−1.9931
4	6	0	−1.003	4.2091	−0.8339
5	6	0	−0.5248	2.8983	−0.816
6	6	0	−0.5356	2.1659	−2.0211
7	7	0	−0.8831	4.7022	0.4438
8	6	0	−0.3567	3.7483	1.2716
9	6	0	−0.1206	2.6081	0.5275
10	6	0	0.4485	1.3207	1.0469
11	6	0	−0.6355	0.371	1.5838
12	6	0	0.0467	−0.7372	2.395
13	7	0	−1.4714	−0.1861	0.5198
14	7	0	0.3587	−1.873	1.6924
15	8	0	0.4406	−0.567	3.5472
16	6	0	−0.0584	−2.1483	0.4089
17	6	0	−1.0288	−1.2187	−0.2652
18	8	0	−1.4435	−1.3798	−1.4064
19	6	0	0.3324	−3.249	−0.2593
20	6	0	1.2152	−4.2563	0.2197
21	7	0	1.7605	−4.2104	1.4789
22	6	0	2.5063	−5.2891	1.5662
23	7	0	2.461	−6.0254	0.4149
24	6	0	1.6341	−5.374	−0.4605
25	1	0	−1.0375	2.1939	−4.115
26	1	0	−1.8725	4.5193	−4.0897

27	1	0	−1.8593	5.8443	−1.9838
28	1	0	−0.1726	1.1425	−2.0546
29	1	0	−1.1482	5.6362	0.7238
30	1	0	−0.1921	3.9616	2.3199
31	1	0	1.164	1.5573	1.846
32	1	0	1.0346	0.834	0.2568
33	1	0	−1.3216	0.8966	2.2587
34	1	0	−2.0712	0.4634	0.0234
35	1	0	0.9324	−2.5786	2.1575
36	1	0	−0.0569	−3.4022	−1.2643
37	1	0	3.0941	−5.5881	2.4243
38	1	0	2.9511	−6.8937	0.2476
39	1	0	1.4279	−5.7546	−1.4509

11*R*-**2b**	Standard Orientation (Ångstroms)				
No.	Atom	Type	X	Y	Z
1	6	0	−1.0003	5.0294	2.0606
2	6	0	−1.5539	5.8741	1.1017
3	6	0	−1.6139	5.4956	−0.2426
4	6	0	−1.0994	4.2398	−0.5867
5	6	0	−0.5396	3.3706	0.3499
6	6	0	−0.4901	3.7771	1.6993
7	7	0	−1.0253	3.6185	−1.8112
8	6	0	−0.4467	2.385	−1.6818
9	6	0	−0.1296	2.1916	−0.3512
10	6	0	0.5115	0.9727	0.2415
11	6	0	−0.5099	0.0264	0.8941
12	6	0	0.2563	−1.0881	1.6154
13	7	0	−1.4602	−0.5247	−0.0727
14	7	0	0.49	−2.2164	0.8703
15	8	0	0.7742	−0.9272	2.7188
16	6	0	−0.061	−2.4757	−0.366
17	6	0	−1.0989	−1.539	−0.9205
18	8	0	−1.6338	−1.6823	−2.013
19	6	0	0.2572	−3.5665	−1.0868
20	6	0	1.1865	−4.5783	−0.7179
21	7	0	1.8635	−4.5473	0.4761
22	6	0	2.6144	−5.626	0.4696
23	7	0	2.4463	−6.3479	−0.6794
24	6	0	1.5304	−5.6868	−1.4531
25	1	0	−0.9615	5.3441	3.1006
26	1	0	−1.9444	6.8438	1.402
27	1	0	−2.0443	6.1569	−0.9873
28	1	0	−0.0586	3.1297	2.4574
29	1	0	−1.3545	4.0201	−2.6782

30	1	0	−0.3114	1.7477	−2.5465
31	1	0	1.2543	1.2897	0.9852
32	1	0	1.077	0.4473	−0.5395
33	1	0	−1.1151	0.5521	1.6417
34	1	0	−2.1097	0.1303	−0.4915
35	1	0	1.1115	−2.9263	1.2617
36	1	0	−0.2367	−3.7067	−2.0467
37	1	0	3.2907	−5.9348	1.2561
38	1	0	2.9157	−7.2134	−0.909
39	1	0	1.2194	−6.0553	−2.4204

11*R*-**2c**			Standard Orientation (Ångstroms)		
No.	Atom	Type	X	Y	Z
1	6	0	3.0781	0.7617	0.6792
2	6	0	3.5224	0.518	−0.6174
3	6	0	2.7485	0.8819	−1.7226
4	6	0	1.5144	1.496	−1.4774
5	6	0	1.039	1.7529	−0.1909
6	6	0	1.8426	1.3789	0.9061
7	7	0	0.5622	1.9488	−2.3589
8	6	0	−0.4958	2.4862	−1.6781
9	6	0	−0.2497	2.3661	−0.3234
10	6	0	−1.1631	2.8033	0.7834
11	6	0	−2.1494	1.7374	1.2945
12	6	0	−1.4173	0.6416	2.0797
13	7	0	−3.0273	1.1949	0.2605
14	7	0	−1.1946	−0.5265	1.4
15	8	0	−0.9406	0.8412	3.1963
16	6	0	−1.6673	−0.8098	0.1375
17	6	0	−2.6495	0.1261	−0.5115
18	8	0	−3.147	−0.062	−1.6162
19	6	0	−1.31	−1.919	−0.5348
20	6	0	−0.4174	−2.929	−0.0803
21	7	0	0.1164	−2.9186	1.1851
22	6	0	0.882	−3.985	1.2404
23	7	0	0.8558	−4.6831	0.0653
24	6	0	0.0212	−4.0189	−0.7927
25	1	0	3.6914	0.4679	1.5272
26	1	0	4.4848	0.0365	−0.7729
27	1	0	3.0967	0.6905	−2.732
28	1	0	1.5088	1.5559	1.925
29	1	0	0.6371	1.8877	−3.3646
30	1	0	−1.3439	2.8891	−2.2161
31	1	0	−1.741	3.6665	0.4259
32	1	0	−0.5558	3.1729	1.6197

33	1	0	−2.8289	2.2175	2.0102
34	1	0	−3.6476	1.8319	−0.2207
35	1	0	−0.5886	−1.2233	1.8351
36	1	0	−1.7299	−2.0715	−1.5269
37	1	0	1.4712	−4.3008	2.0913
38	1	0	1.3637	−5.5353	−0.1283
39	1	0	−0.1721	−4.3688	−1.7967

11*R*-**2d**			Standard Orientation (Ångstroms)		
No.	Atom	Type	X	Y	Z
1	6	0	−0.7903	2.1852	−3.2642
2	6	0	0.3737	1.6488	−3.8076
3	6	0	1.4532	1.3002	−2.9917
4	6	0	1.3197	1.5091	−1.6137
5	6	0	0.1675	2.0451	−1.037
6	6	0	−0.9056	2.3896	−1.8843
7	7	0	2.2044	1.2572	−0.5926
8	6	0	1.6583	1.6225	0.6064
9	6	0	0.3812	2.0995	0.3786
10	6	0	−0.5742	2.5825	1.4289
11	6	0	−1.4694	1.504	2.064
12	6	0	−0.6276	0.4194	2.7519
13	7	0	−2.4582	0.943	1.1485
14	7	0	−0.4591	−0.7397	2.0384
15	8	0	−0.0261	0.6216	3.8058
16	6	0	−1.0842	−1.0317	0.8455
17	6	0	−2.183	−0.1341	0.3482
18	8	0	−2.8581	−0.3649	−0.6486
19	6	0	−0.7817	−2.1214	0.1169
20	6	0	0.2007	−3.1011	0.43
21	7	0	0.9603	−3.0395	1.5728
22	6	0	1.7344	−4.1004	1.5245
23	7	0	1.504	−4.8393	0.3974
24	6	0	0.5212	−4.2089	−0.3169
25	1	0	−1.6226	2.4423	−3.9146
26	1	0	0.4431	1.4953	−4.8819
27	1	0	2.3584	0.8803	−3.4171
28	1	0	−1.824	2.7992	−1.4761
29	1	0	3.1241	0.8576	−0.7156
30	1	0	2.2159	1.4941	1.5258
31	1	0	−1.2091	3.3675	0.9982
32	1	0	0.0099	3.0681	2.2225
33	1	0	−2.0669	1.9771	2.8533
34	1	0	−3.1716	1.5518	0.7711
35	1	0	0.2161	−1.42	2.3879

36	1	0	−1.3306	−2.2821	−0.8092
37	1	0	2.4701	−4.3848	2.2653
38	1	0	1.9749	−5.6975	0.145
39	1	0	0.1511	−4.5955	−1.2559

11*R*-**2e**			Standard Orientation (Ångstroms)		
No.	Atom	Type	X	Y	Z
1	6	0	−1.819	4.4537	−1.7868
2	6	0	−0.6257	5.1122	−2.0715
3	6	0	0.5361	4.8417	−1.3434
4	6	0	0.4558	3.8864	−0.323
5	6	0	−0.7227	3.2056	−0.0143
6	6	0	−1.8815	3.5012	−0.7628
7	7	0	1.4242	3.4312	0.54
8	6	0	0.9015	2.4911	1.3843
9	6	0	−0.4304	2.3097	1.0661
10	6	0	−1.3791	1.3598	1.7374
11	6	0	−1.2949	−0.0864	1.2263
12	6	0	−0.0224	−0.8016	1.7161
13	7	0	−1.4685	−0.1903	−0.2197
14	7	0	0.3406	−1.898	0.9722
15	8	0	0.5329	−0.5538	2.7848
16	6	0	−0.2286	−2.2702	−0.2258
17	6	0	−1.2469	−1.3729	−0.8728
18	8	0	−1.811	−1.6319	−1.9291
19	6	0	0.1243	−3.3945	−0.8757
20	6	0	1.0853	−4.3514	−0.4463
21	7	0	1.7933	−4.2034	0.7207
22	6	0	2.5727	−5.2598	0.7824
23	7	0	2.3942	−6.0791	−0.2976
24	6	0	1.4409	−5.508	−1.0971
25	1	0	−2.7117	4.6804	−2.3643
26	1	0	−0.596	5.8485	−2.8713
27	1	0	1.4644	5.3571	−1.5665
28	1	0	−2.8205	3.0003	−0.5506
29	1	0	2.3828	3.7503	0.5468
30	1	0	1.5219	2.0212	2.1377
31	1	0	−2.4035	1.7249	1.5877
32	1	0	−1.2063	1.3899	2.8208
33	1	0	−2.1285	−0.6495	1.6688
34	1	0	−2.1864	0.3719	−0.6571
35	1	0	1.0959	−2.4842	1.3306
36	1	0	−0.36	−3.6078	−1.827
37	1	0	3.2779	−5.4845	1.5719
38	1	0	2.8823	−6.9477	−0.4692

39	1	0	1.1167	−5.9621	−2.0228

Table S17. Boltzmann population [a] and optical rotation (OR) of low-energy conformers of 11*S*-**2**

Conformers	In MeOH	
	Boltzmann population (%)	OR
11*S*-**2a**	9.78	−737.78
11*S*-**2b**	62.33	−595.63
11*S*-**2c**	16.36	−86.51
11*S*-**2d**	10.24	−1258.35
11*S*-**2e**	1.30	−870.53
Average		−697.60

[a] From Δ*G* values at 298.15K.

Table S18. Cartesian coordinates for the low-energy reoptimized MMFF conformers of 11*S*-**2** at B3LYP/6-311+G (d, p) level of theory in MeOH

11*S*-**2a**		Standard Orientation a(Ångstroms)			
No.	Atom	Type	X	Y	Z
1	6	0	1.4524	0.2577	4.0836
2	6	0	1.596	1.3326	4.9565
3	6	0	1.4796	2.6486	4.501
4	6	0	1.211	2.8439	3.1407
5	6	0	1.0604	1.7888	2.2399
6	6	0	1.1876	0.4711	2.7259
7	7	0	1.0492	4.0098	2.4306
8	6	0	0.7911	3.7313	1.1161
9	6	0	0.7871	2.359	0.9542
10	6	0	0.5391	1.6252	−0.3307
11	6	0	−0.954	1.3465	−0.5716
12	6	0	−1.14	0.9209	−2.0332
13	7	0	−1.4937	0.3437	0.3472
14	7	0	−1.0939	−0.4304	−2.2625
15	8	0	−1.1614	1.7287	−2.9599
16	6	0	−1.0876	−1.3988	−1.2831
17	6	0	−1.2659	−0.9939	0.1538
18	8	0	−1.2622	−1.7854	1.0885
19	6	0	−0.9738	−2.7103	−1.5619
20	6	0	−0.8296	−3.2875	−2.854
21	7	0	−0.8151	−2.5258	−3.9962
22	6	0	−0.6719	−3.3877	−4.9781
23	7	0	−0.5946	−4.671	−4.5125
24	6	0	−0.695	−4.6227	−3.1479

25	1	0	1.5445	−0.7596	4.4565
26	1	0	1.8021	1.1461	6.0079
27	1	0	1.5937	3.4843	5.1834
28	1	0	1.0788	−0.3811	2.061
29	1	0	1.1127	4.9362	2.8294
30	1	0	0.6343	4.5328	0.4056
31	1	0	1.1145	0.6906	−0.3359
32	1	0	0.9422	2.2281	−1.1556
33	1	0	−1.5547	2.2503	−0.4141
34	1	0	−1.5438	0.6021	1.3263
35	1	0	−1.0774	−0.7489	−3.2329
36	1	0	−0.9953	−3.4134	−0.7311
37	1	0	−0.6185	−3.1478	−6.0321
38	1	0	−0.4844	−5.501	−5.0792
39	1	0	−0.6633	−5.5128	−2.5355

11*S*-**2b**	Standard Orientation (Ångstroms)				
No.	Atom	Type	X	Y	Z
1	6	0	0.5017	5.2954	1.4994
2	6	0	0.7844	5.4178	2.8576
3	6	0	1.104	4.2975	3.6301
4	6	0	1.1277	3.0529	2.9895
5	6	0	0.8484	2.897	1.6316
6	6	0	0.5302	4.0433	0.8745
7	7	0	1.4069	1.8036	3.4918
8	6	0	1.3062	0.8627	2.5032
9	6	0	0.9598	1.5022	1.3287
10	6	0	0.7341	0.8527	−0.0037
11	6	0	−0.7566	0.6276	−0.306
12	6	0	−0.8866	0.1258	−1.7485
13	7	0	−1.3853	−0.2995	0.6356
14	7	0	−0.8804	−1.2382	−1.8978
15	8	0	−0.8298	0.8794	−2.7183
16	6	0	−0.9536	−2.1471	−0.8645
17	6	0	−1.1863	−1.6518	0.5365
18	8	0	−1.2538	−2.3831	1.5166
19	6	0	−0.872	−3.4757	−1.061
20	6	0	−0.6868	−4.133	−2.3089
21	7	0	−0.5908	−3.4412	−3.4909
22	6	0	−0.4318	−4.3639	−4.4133
23	7	0	−0.4226	−5.6193	−3.8713
24	6	0	−0.5861	−5.487	−2.5184
25	1	0	0.2574	6.1799	0.9162
26	1	0	0.758	6.3996	3.3248
27	1	0	1.3254	4.3968	4.6876

28	1	0	0.3102	3.9632	−0.1863
29	1	0	1.6491	1.6143	4.4546
30	1	0	1.487	−0.1829	2.7187
31	1	0	1.2806	−0.0992	−0.0376
32	1	0	1.1821	1.4815	−0.7843
33	1	0	−1.3222	1.5633	−0.2268
34	1	0	−1.4735	0.0195	1.593
35	1	0	−0.8275	−1.614	−2.8461
36	1	0	−0.957	−4.1269	−0.1928
37	1	0	−0.3198	−4.1888	−5.4753
38	1	0	−0.3155	−6.4845	−4.3831
39	1	0	−0.6155	−6.3397	−1.855

11*S*-**2c**			Standard Orientation (Ångstroms)		
No.	Atom	Type	X	Y	Z
1	6	0	2.7317	0.5108	−1.6714
2	6	0	3.4036	−0.5549	−1.0791
3	6	0	3.2428	−0.8401	0.2794
4	6	0	2.3827	−0.0186	1.0182
5	6	0	1.6932	1.0559	0.4551
6	6	0	1.8774	1.3235	−0.9173
7	7	0	2.0397	−0.0677	2.348
8	6	0	1.1622	0.9381	2.6481
9	6	0	0.9026	1.6503	1.4925
10	6	0	−0.0263	2.8214	1.3653
11	6	0	−1.4785	2.4824	0.9828
12	6	0	−1.5687	2.0147	−0.4754
13	7	0	−2.1384	1.5644	1.9079
14	7	0	−1.6714	0.6611	−0.6585
15	8	0	−1.422	2.7862	−1.4226
16	6	0	−1.8056	−0.266	0.3514
17	6	0	−2.0384	0.2044	1.7608
18	8	0	−2.2015	−0.5476	2.7154
19	6	0	−1.771	−1.5915	0.1235
20	6	0	−1.5863	−2.2253	−1.1365
21	7	0	−1.518	−1.5151	−2.31
22	6	0	−1.3424	−2.4203	−3.246
23	7	0	−1.3023	−3.6826	−2.7223
24	6	0	−1.462	−3.5741	−1.3671
25	1	0	2.8654	0.7153	−2.7306
26	1	0	4.0622	−1.1753	−1.6821
27	1	0	3.7671	−1.6714	0.7385
28	1	0	1.3561	2.1474	−1.3974
29	1	0	2.3867	−0.7529	3.0045
30	1	0	0.7805	1.048	3.6547

31	1	0	0.3904	3.5275	0.6356
32	1	0	−0.0367	3.3526	2.3268
33	1	0	−2.0699	3.4051	1.0399
34	1	0	−2.2438	1.8521	2.8713
35	1	0	−1.5896	0.3011	−1.6102
36	1	0	−1.8876	−2.2585	0.9752
37	1	0	−1.2395	−2.227	−4.3057
38	1	0	−1.177	−4.5373	−3.2471
39	1	0	−1.4672	−4.4362	−0.7155

11*S*-**2d**			Standard Orientation (Ångstroms)		
No.	Atom	Type	X	Y	Z
1	6	0	1.1614	−0.4017	3.8137
2	6	0	2.0629	−1.3582	3.355
3	6	0	2.6149	−1.2694	2.0744
4	6	0	2.2282	−0.1877	1.2741
5	6	0	1.3278	0.7883	1.7033
6	6	0	0.7877	0.6748	3.0009
7	7	0	2.6021	0.1412	−0.0068
8	6	0	1.9794	1.2933	−0.3996
9	6	0	1.1594	1.7199	0.628
10	6	0	0.2782	2.9332	0.5999
11	6	0	−1.1356	2.7185	0.0319
12	6	0	−1.0803	2.2259	−1.4216
13	7	0	−1.9883	1.8815	0.8704
14	7	0	−1.2623	0.8779	−1.5965
15	8	0	−0.7553	2.9596	−2.3541
16	6	0	−1.6106	−0.0086	−0.6006
17	6	0	−2.0195	0.5177	0.7471
18	8	0	−2.4354	−0.1863	1.6604
19	6	0	−1.6432	−1.3395	−0.7943
20	6	0	−1.3214	−2.0262	−1.9975
21	7	0	−0.9473	−1.3654	−3.1421
22	6	0	−0.7441	−2.3121	−4.0307
23	7	0	−0.9708	−3.553	−3.5032
24	6	0	−1.3418	−3.3856	−2.1964
25	1	0	0.7363	−0.4927	4.8101
26	1	0	2.3389	−2.1885	4.0006
27	1	0	3.3155	−2.0172	1.7182
28	1	0	0.0759	1.406	3.3703
29	1	0	3.2439	−0.3956	−0.5731
30	1	0	2.1555	1.704	−1.386
31	1	0	0.7834	3.7066	0.0055
32	1	0	0.1984	3.3383	1.6169
33	1	0	−1.6458	3.6897	0.0088

34	1	0	−2.2277	2.2065	1.7972
35	1	0	−1.08	0.489	−2.5218
36	1	0	−1.9386	−1.9681	0.0439
37	1	0	−0.4377	−2.1646	−5.058
38	1	0	−0.8827	−4.4313	−3.9959
39	1	0	−1.5813	−4.22	−1.5524

11*S*-**2e**	Standard Orientation (Ångstroms)				
No.	Atom	Type	X	Y	Z
1	6	0	1.198	2.4214	4.3633
2	6	0	2.5823	2.4947	4.2338
3	6	0	3.1842	2.5593	2.9742
4	6	0	2.3465	2.5454	1.8524
5	6	0	0.9563	2.4688	1.9466
6	6	0	0.3752	2.4086	3.2307
7	7	0	2.6593	2.5993	0.5147
8	6	0	1.5193	2.563	−0.2395
9	6	0	0.4349	2.4686	0.6111
10	6	0	−1.0069	2.3907	0.2012
11	6	0	−1.4791	0.982	−0.1894
12	6	0	−0.9282	0.5444	−1.5589
13	7	0	−1.2309	−0.0109	0.8519
14	7	0	−0.9317	−0.813	−1.7704
15	8	0	−0.6785	1.3241	−2.4763
16	6	0	−1.2134	−1.7659	−0.8164
17	6	0	−1.4198	−1.3455	0.6124
18	8	0	−1.6859	−2.1236	1.5206
19	6	0	−1.2674	−3.0802	−1.1007
20	6	0	−1.0704	−3.6763	−2.3772
21	7	0	−0.7733	−2.9334	−3.493
22	6	0	−0.6613	−3.81	−4.4658
23	7	0	−0.8735	−5.0849	−4.0196
24	6	0	−1.1374	−5.0152	−2.6781
25	1	0	0.7502	2.3737	5.3527
26	1	0	3.2056	2.5026	5.125
27	1	0	4.2631	2.6175	2.876
28	1	0	−0.7022	2.3559	3.3492
29	1	0	3.5983	2.6593	0.147
30	1	0	1.5726	2.5988	−1.3208
31	1	0	−1.1808	3.0917	−0.6251
32	1	0	−1.6266	2.7494	1.0333
33	1	0	−2.5702	1.0154	−0.3168
34	1	0	−1.4161	0.2463	1.8123
35	1	0	−0.7033	−1.1465	−2.708
36	1	0	−1.4779	−3.7706	−0.2859

37	1	0	−0.4312	−3.5869	−5.4994
38	1	0	−0.8392	−5.9232	−4.5836
39	1	0	−1.3403	−5.8947	−2.0835

Table S19. Gibbs free energies [a] and equilibrium populations [b] of low-energy conformers of 11*R*-**2**

Conformers	In MeOH	
	G	*P* (%)
11*R*-**2a**	−1081.1733003	4.06%
11*R*-**2b**	−1081.1746994	17.88%
11*R*-**2c**	−1081.1740062	8.57%
11*R*-**2d**	−1081.1757619	55.14%
11*R*-**2e**	−1081.1744918	14.35%

[a] B3LYP/6-31+g (d, p), in kcal/mol; [b] from G values at 298.15K.

Table S20. Cartesian coordinates for the low-energy reoptimized MMFF conformers of 11*R*-**2** at B3LYP/6-311+G (d, p) level of theory in MeOH

11*R*-**2a**			Standard Orientation (Ångstroms)		
No.	Atom	Type	X	Y	Z
1	6	0	4.641379	−1.783357	1.441109
2	6	0	5.699567	−1.776745	0.536005
3	6	0	5.761189	−0.831963	−0.492233
4	6	0	4.721794	0.101975	−0.578829
5	6	0	3.647572	0.12161	0.31084
6	6	0	3.611305	−0.841157	1.340824
7	7	0	4.526937	1.128953	−1.471834
8	6	0	3.363101	1.79048	−1.186508
9	6	0	2.782483	1.192862	−0.084507
10	6	0	1.490605	1.596	0.565106
11	6	0	0.261202	0.966246	−0.105801
12	6	0	−1.026332	1.572473	0.479715
13	7	0	0.251792	−0.492672	0.003854
14	7	0	−2.153186	0.807882	0.303744
15	8	0	−1.100874	2.71763	0.919763
16	6	0	−2.163654	−0.490727	−0.155166
17	6	0	−0.858628	−1.206138	−0.368597
18	8	0	−0.771746	−2.355255	−0.783525
19	6	0	−3.302683	−1.172657	−0.374864
20	6	0	−4.62624	−0.683209	−0.19515
21	7	0	−4.879558	0.583678	0.269457
22	6	0	−6.190004	0.675961	0.308942
23	7	0	−6.78761	−0.480131	−0.110332
24	6	0	−5.794606	−1.364115	−0.437319
25	1	0	4.612935	−2.526376	2.234308

26	1	0	6.491105	−2.516746	0.630506
27	1	0	6.588269	−0.828904	−1.194553
28	1	0	2.792407	−0.857725	2.054485
29	1	0	5.157714	1.356922	−2.227783
30	1	0	3.048699	2.632454	−1.78931
31	1	0	1.419056	2.691125	0.532072
32	1	0	1.513764	1.327552	1.629261
33	1	0	0.25574	1.199638	−1.179213
34	1	0	1.108011	−0.972709	−0.252663
35	1	0	−3.054125	1.224549	0.543821
36	1	0	−3.223367	−2.201848	−0.720527
37	1	0	−6.757302	1.540749	0.627566
38	1	0	−7.783402	−0.646064	−0.165179
39	1	0	−6.005467	−2.360839	−0.798304

11*R*-**2b**			Standard Orientation (Ångstroms)		
No.	Atom	Type	X	Y	Z
1	6	0	4.653588	−1.747757	1.755678
2	6	0	5.704153	−0.839956	1.863012
3	6	0	5.766531	0.289052	1.041367
4	6	0	4.735806	0.473076	0.111904
5	6	0	3.66817	−0.415309	−0.017821
6	6	0	3.63113	−1.548496	0.82086
7	7	0	4.545765	1.472914	−0.812546
8	6	0	3.392542	1.253925	−1.516462
9	6	0	2.812471	0.09034	−1.048924
10	6	0	1.529634	−0.522358	−1.530931
11	6	0	0.34816	−0.25389	−0.587288
12	6	0	−0.883948	−1.054641	−1.041964
13	7	0	0.039743	1.172148	−0.481719
14	7	0	−2.084244	−0.572431	−0.580973
15	8	0	−0.816294	−2.135154	−1.623775
16	6	0	−2.258402	0.636264	0.056714
17	6	0	−1.098475	1.587416	0.160133
18	8	0	−1.15763	2.678226	0.714072
19	6	0	−3.445971	1.029819	0.552065
20	6	0	−4.660581	0.290217	0.517504
21	7	0	−4.748321	−0.942138	−0.081461
22	6	0	−6.000131	−1.309548	0.077854
23	7	0	−6.719196	−0.362799	0.753244
24	6	0	−5.871398	0.672684	1.042056
25	1	0	4.626494	−2.621844	2.401743
26	1	0	6.489975	−1.012645	2.594902
27	1	0	6.588474	0.992308	1.125285

28	1	0	2.82102	−2.267786	0.744222
29	1	0	5.173875	2.252859	−0.948953
30	1	0	3.082939	1.942717	−2.29192
31	1	0	1.686265	−1.602275	−1.648689
32	1	0	1.297158	−0.137984	−2.53323
33	1	0	0.585105	−0.598549	0.428192
34	1	0	0.822119	1.789461	−0.297139
35	1	0	−2.917085	−1.144119	−0.731532
36	1	0	−3.502757	2.008078	1.026196
37	1	0	−6.440053	−2.235512	−0.268735
38	1	0	−7.699798	−0.422443	0.991936
39	1	0	−6.198239	1.554275	1.574987

	11*R*-**2c**		Standard Orientation (Ångstroms)		
No.	Atom	Type	X	Y	Z
1	6	0	−3.722661	−2.555393	−0.381782
2	6	0	−5.076942	−2.259365	−0.253954
3	6	0	−5.523379	−0.935468	−0.224484
4	6	0	−4.561303	0.076933	−0.324635
5	6	0	−3.196773	−0.185617	−0.452634
6	6	0	−2.774652	−1.530797	−0.484446
7	7	0	−4.713236	1.443339	−0.324234
8	6	0	−3.494473	2.054865	−0.436395
9	6	0	−2.521956	1.076564	−0.517158
10	6	0	−1.04561	1.311775	−0.644808
11	6	0	−0.351	1.452757	0.720108
12	6	0	1.052763	2.027462	0.49461
13	7	0	−0.296732	0.18931	1.455986
14	7	0	2.051524	1.099074	0.348236
15	8	0	1.253336	3.22333	0.290998
16	6	0	1.914428	−0.25239	0.575657
17	6	0	0.625012	−0.77599	1.14475
18	8	0	0.412467	−1.961412	1.367951
19	6	0	2.910206	−1.129364	0.35211
20	6	0	4.205212	−0.823252	−0.150492
21	7	0	4.588077	0.461278	−0.447729
22	6	0	5.826694	0.35704	−0.87483
23	7	0	6.254296	−0.941738	−0.8629
24	6	0	5.222403	−1.71269	−0.398887
25	1	0	−3.393641	−3.591626	−0.401015
26	1	0	−5.798154	−3.069614	−0.176063
27	1	0	−6.579759	−0.707967	−0.126334
28	1	0	−1.722464	−1.782607	−0.583453
29	1	0	−5.601108	1.920236	−0.250726

30	1	0	−3.421562	3.134781	−0.451799
31	1	0	−0.891563	2.224535	−1.23598
32	1	0	−0.594268	0.499154	−1.228258
33	1	0	−0.89196	2.152993	1.367751
34	1	0	−1.180761	−0.191596	1.774503
35	1	0	2.97395	1.432414	0.062615
36	1	0	2.72665	−2.179002	0.574364
37	1	0	6.457553	1.173411	−1.201521
38	1	0	7.16886	−1.266761	−1.145691
39	1	0	5.306484	−2.784961	−0.291902

	11*R*-**2d**		Standard Orientation (Ångstroms)		
No.	Atom	Type	X	Y	Z
1	6	0	−5.436163	1.719814	0.164566
2	6	0	−6.328447	0.65232	0.104241
3	6	0	−5.887029	−0.638508	−0.200459
4	6	0	−4.519285	−0.816779	−0.4405
5	6	0	−3.600146	0.231434	−0.386639
6	6	0	−4.071701	1.524157	−0.078386
7	7	0	−3.817175	−1.955525	−0.758847
8	6	0	−2.486769	−1.667143	−0.89997
9	6	0	−2.309272	−0.315581	−0.676127
10	6	0	−1.008541	0.42888	−0.71847
11	6	0	−0.412669	0.657067	0.680598
12	6	0	0.811748	1.568603	0.541499
13	7	0	−0.066255	−0.595692	1.352899
14	7	0	2.010508	0.92036	0.381746
15	8	0	0.717172	2.787434	0.410824
16	6	0	2.200255	−0.437342	0.523364
17	6	0	1.063674	−1.292442	1.012258
18	8	0	1.136125	−2.506443	1.157226
19	6	0	3.383573	−1.032279	0.285697
20	6	0	4.576615	−0.391021	−0.148791
21	7	0	4.642677	0.964502	−0.356185
22	6	0	5.879043	1.190054	−0.741043
23	7	0	6.608762	0.034639	−0.788376
24	6	0	5.784733	−0.990981	−0.409642
25	1	0	−5.800707	2.716525	0.401289
26	1	0	−7.385156	0.82476	0.29532
27	1	0	−6.585229	−1.467557	−0.248376
28	1	0	−3.387549	2.366605	−0.030672
29	1	0	−4.230357	−2.871118	−0.869229
30	1	0	−1.777955	−2.44797	−1.145536
31	1	0	−1.16901	1.393715	−1.217279

32	1	0	−0.299665	−0.120234	−1.352588
33	1	0	−1.129883	1.160974	1.33885
34	1	0	−0.838709	−1.196178	1.615558
35	1	0	2.830326	1.483795	0.149808
36	1	0	3.454653	−2.107708	0.438515
37	1	0	6.300066	2.153937	−0.995559
38	1	0	7.581217	−0.041058	−1.054409
39	1	0	6.12425	−2.015934	−0.36192

	11*R*-**2e**		Standard Orientation (Ångstroms)		
No.	Atom	Type	X	Y	Z
1	6	0	−0.228983	3.071676	1.456008
2	6	0	−0.024305	3.789316	0.280815
3	6	0	−0.595618	3.372481	−0.924483
4	6	0	−1.376113	2.2103	−0.905645
5	6	0	−1.599423	1.467171	0.253977
6	6	0	−1.014156	1.913055	1.457182
7	7	0	−2.04489	1.578465	−1.926564
8	6	0	−2.686362	0.46595	−1.455089
9	6	0	−2.418544	0.346838	−0.104472
10	6	0	−2.903562	−0.747134	0.800195
11	6	0	−1.969972	−1.963772	0.935578
12	6	0	−0.71142	−1.605289	1.735823
13	7	0	−1.660183	−2.619726	−0.332487
14	7	0	0.417928	−1.378458	0.994668
15	8	0	−0.740767	−1.400603	2.948787
16	6	0	0.518504	−1.553906	−0.367929
17	6	0	−0.611477	−2.207762	−1.114606
18	8	0	−0.595693	−2.43376	−2.319619
19	6	0	1.611736	−1.191992	−1.063469
20	6	0	2.784497	−0.585448	−0.534208
21	7	0	2.962437	−0.388736	0.813331
22	6	0	4.137902	0.188377	0.920862
23	7	0	4.724774	0.363566	−0.30145
24	6	0	3.868489	−0.132968	−1.247127
25	1	0	0.226856	3.408235	2.383724
26	1	0	0.589769	4.68637	0.300227
27	1	0	−0.434282	3.933176	−1.839059
28	1	0	−1.15826	1.361902	2.382661
29	1	0	−2.054934	1.89366	−2.886485
30	1	0	−3.261502	−0.158652	−2.126
31	1	0	−3.873942	−1.095928	0.421275
32	1	0	−3.102377	−0.324427	1.793433
33	1	0	−2.491921	−2.729868	1.522832
34	1	0	−2.424849	−3.008624	−0.867485

35	1	0	1.235407	−0.999735	1.474648
36	1	0	1.614028	−1.367039	−2.137388
37	1	0	4.611774	0.498219	1.843044
38	1	0	5.62818	0.784639	−0.469294
39	1	0	4.100121	−0.114181	−2.302434

Table S21. Gibbs free energies [a] and equilibrium populations [b] of low-energy conformers of 11*S*-**2**

Conformers	In MeOH	
	G	*P* (%)
11*S*-**2a**	−1081.1732837	4.01%
11*S*-**2b**	−1081.1746967	17.93%
11*S*-**2c**	−1081.1740092	8.65%
11*S*-**2d**	−1081.1757548	55.05%
11*S*-**2e**	−1081.1744874	14.36%

[a] B3LYP/6-31+g (d, p), in kcal/mol; [b] from G values at 298.15K.

Table S22. Cartesian coordinates for the low-energy reoptimized MMFF conformers of 11*S*-**2** at B3LYP/6-311+G (d, p) level of theory in MeOH

11*S*-**2a**			Standard Orientation (Ångstroms)		
No.	Atom	Type	X	Y	Z
1	6	0	−4.64141	−1.783354	1.441029
2	6	0	−5.699599	−1.776654	0.536088
3	6	0	−5.761167	−0.831994	−0.492203
4	6	0	−4.721711	0.101962	−0.578822
5	6	0	−3.647586	0.121589	0.310799
6	6	0	−3.611219	−0.84117	1.340805
7	7	0	−4.527013	1.129031	−1.471852
8	6	0	−3.363099	1.790491	−1.186496
9	6	0	−2.782422	1.192837	−0.084445
10	6	0	−1.490562	1.596021	0.565156
11	6	0	−0.261226	0.966177	−0.105815
12	6	0	1.026284	1.572486	0.479677
13	7	0	−0.251818	−0.492735	0.00384
14	7	0	2.153236	0.807826	0.303694
15	8	0	1.100906	2.717588	0.919795
16	6	0	2.163675	−0.490751	−0.155157
17	6	0	0.858585	−1.20617	−0.368675
18	8	0	0.771669	−2.355221	−0.783511
19	6	0	3.302769	−1.172634	−0.37485
20	6	0	4.626171	−0.683164	−0.195161
21	7	0	4.879522	0.583745	0.269512

22	6	0	6.190002	0.676021	0.308919
23	7	0	6.787629	−0.480186	−0.110425
24	6	0	5.79457	−1.364147	−0.437221
25	1	0	−4.612959	−2.526336	2.234355
26	1	0	−6.491101	−2.516693	0.63051
27	1	0	−6.588225	−0.828817	−1.194617
28	1	0	−2.792367	−0.857745	2.054387
29	1	0	−5.157687	1.356915	−2.227771
30	1	0	−3.048704	2.632487	−1.78931
31	1	0	−1.51379	1.327544	1.629271
32	1	0	−1.419056	2.691114	0.532125
33	1	0	−0.255687	1.199597	−1.179239
34	1	0	−1.107955	−0.972721	−0.252659
35	1	0	3.054194	1.224597	0.543841
36	1	0	3.223371	−2.201859	−0.720532
37	1	0	6.757326	1.540759	0.627597
38	1	0	7.783367	−0.645986	−0.165171
39	1	0	6.005453	−2.360846	−0.79822

	11*S*-**2b**		Standard Orientation (Ångstroms)		
No.	Atom	Type	X	Y	Z
1	6	0	−3.1904	3.909	0.4887
2	6	0	−3.4365	4.397	1.7696
3	6	0	−2.4831	4.2697	2.7838
4	6	0	−1.2774	3.6335	2.4649
5	6	0	−1.0018	3.1307	1.1933
6	6	0	−1.9793	3.2754	0.1871
7	7	0	−0.1767	3.3788	3.2487
8	6	0	0.7815	2.7258	2.5215
9	6	0	0.3053	2.547	1.2369
10	6	0	1.0231	1.8686	0.1065
11	6	0	0.5253	0.4375	−0.1436
12	6	0	1.1697	−0.1308	−1.4195
13	7	0	0.7663	−0.4384	1.0029
14	7	0	1.169	−1.5016	−1.5015
15	8	0	1.5073	0.5613	−2.3774
16	6	0	0.8322	−2.355	−0.4738
17	6	0	0.5657	−1.7904	0.8941
18	8	0	0.2536	−2.4701	1.8641
19	6	0	0.78	−3.6907	−0.6285
20	6	0	1.0347	−4.4107	−1.8286
21	7	0	1.4069	−3.7833	−2.9917
22	6	0	1.5556	−4.7514	−3.8681

23	7	0	1.2953	−5.9743	−3.3144
24	6	0	0.9598	−5.7715	−2.0026
25	1	0	−3.944	4.0223	−0.2868
26	1	0	−4.3832	4.8874	1.9844
27	1	0	−2.6768	4.6543	3.7797
28	1	0	−1.7987	2.9049	−0.8177
29	1	0	−0.0934	3.6421	4.2209
30	1	0	1.7219	2.4455	2.9783
31	1	0	2.1009	1.8538	0.317
32	1	0	0.8985	2.4793	−0.7969
33	1	0	−0.5592	0.4355	−0.3161
34	1	0	0.4743	−0.0865	1.9075
35	1	0	1.4517	−1.9264	−2.3862
36	1	0	0.5225	−4.2957	0.239
37	1	0	1.8464	−4.6326	−4.9037
38	1	0	1.3448	−6.8635	−3.7926
39	1	0	0.7083	−6.5866	−1.3389

	11*S*-**2c**		Standard Orientation (Ångstroms)		
No.	Atom	Type	X	Y	Z
1	6	0	3.722696	−2.555438	−0.38186
2	6	0	5.076931	−2.259346	−0.253948
3	6	0	5.523341	−0.935397	−0.224444
4	6	0	4.56132	0.076951	−0.324634
5	6	0	3.196731	−0.185584	−0.452692
6	6	0	2.77461	−1.530814	−0.484464
7	7	0	4.713283	1.44329	−0.324179
8	6	0	3.494477	2.054879	−0.436446
9	6	0	2.521963	1.07656	−0.517148
10	6	0	1.045664	1.311773	−0.644746
11	6	0	0.35101	1.452776	0.720082
12	6	0	−1.052787	2.027459	0.494651
13	7	0	0.296798	0.189242	1.455997
14	7	0	−2.051526	1.099118	0.348215
15	8	0	−1.253318	3.223314	0.291015
16	6	0	−1.914413	−0.252445	0.575699
17	6	0	−0.625054	−0.776027	1.144705
18	8	0	−0.412407	−1.961465	1.36794
19	6	0	−2.910267	−1.129326	0.352101
20	6	0	−4.205302	−0.823304	−0.150548
21	7	0	−4.588087	0.461329	−0.447741
22	6	0	−5.826723	0.357007	−0.874834
23	7	0	−6.254303	−0.941664	−0.862869

24	6	0	−5.222389	−1.712659	−0.398844
25	1	0	3.393587	−3.591599	−0.401064
26	1	0	5.79817	−3.069608	−0.176022
27	1	0	6.579685	−0.707892	−0.126329
28	1	0	1.722476	−1.782526	−0.583398
29	1	0	5.601178	1.920227	−0.250694
30	1	0	3.421536	3.134745	−0.451808
31	1	0	0.594192	0.499153	−1.228278
32	1	0	0.891587	2.22462	−1.235948
33	1	0	0.892001	2.15297	1.367719
34	1	0	1.180801	−0.191586	1.774502
35	1	0	−2.973893	1.43239	0.062657
36	1	0	−2.726604	−2.179002	0.574389
37	1	0	−6.457554	1.173371	−1.201512
38	1	0	−7.168827	−1.266672	−1.145678
39	1	0	−5.306542	−2.784977	−0.29192

	11*S*-**2d**		Standard Orientation (Ångstroms)		
No.	Atom	Type	X	Y	Z
1	6	0	5.436161	1.719876	0.164569
2	6	0	6.328437	0.652316	0.104281
3	6	0	5.886915	−0.638476	−0.20049
4	6	0	4.519258	−0.816711	−0.440541
5	6	0	3.600156	0.231387	−0.386656
6	6	0	4.0717	1.524144	−0.078361
7	7	0	3.817145	−1.955481	−0.758838
8	6	0	2.486743	−1.66715	−0.900055
9	6	0	2.309262	−0.315577	−0.676111
10	6	0	1.008457	0.42889	−0.718501
11	6	0	0.412717	0.65703	0.68059
12	6	0	−0.811777	1.56858	0.541541
13	7	0	0.066256	−0.595773	1.352881
14	7	0	−2.010527	0.92032	0.381835
15	8	0	−0.717122	2.787445	0.410818
16	6	0	−2.200242	−0.437336	0.523338
17	6	0	−1.063623	−1.292457	1.01229
18	8	0	−1.136058	−2.506531	1.157205
19	6	0	−3.383513	−1.032242	0.285725
20	6	0	−4.576637	−0.391094	−0.148855
21	7	0	−4.64272	0.964574	−0.356209
22	6	0	−5.878993	1.190056	−0.741053
23	7	0	−6.60881	0.034687	−0.78836
24	6	0	−5.784675	−0.990925	−0.409605

25	1	0	5.800698	2.716513	0.401322
26	1	0	7.385177	0.824716	0.295377
27	1	0	6.585263	−1.467541	−0.248379
28	1	0	3.387587	2.366576	−0.030565
29	1	0	4.230362	−2.871111	−0.869316
30	1	0	1.778011	−2.447927	−1.145559
31	1	0	0.299729	−0.120178	−1.35257
32	1	0	1.16891	1.393725	−1.217195
33	1	0	1.129917	1.160999	1.338819
34	1	0	0.838728	−1.196194	1.615528
35	1	0	−2.830317	1.483745	0.149837
36	1	0	−3.454575	−2.10773	0.438513
37	1	0	−6.300008	2.153966	−0.995514
38	1	0	−7.581221	−0.041048	−1.054359
39	1	0	−6.124304	−2.015973	−0.361921

	11*S*-**2e**		Standard Orientation (Ångstroms)		
No.	Atom	Type	X	Y	Z
1	6	0	0.228901	3.071693	1.455991
2	6	0	0.024193	3.789325	0.280795
3	6	0	0.595555	3.37256	−0.924476
4	6	0	1.376044	2.210309	−0.905655
5	6	0	1.599441	1.467197	0.254053
6	6	0	1.014101	1.913104	1.457244
7	7	0	2.044838	1.578495	−1.926598
8	6	0	2.686341	0.465918	−1.455064
9	6	0	2.418495	0.346899	−0.104509
10	6	0	2.903523	−0.747058	0.800173
11	6	0	1.970004	−1.963702	0.935595
12	6	0	0.711431	−1.605316	1.735886
13	7	0	1.660236	−2.619671	−0.332472
14	7	0	−0.417915	−1.378482	0.99457
15	8	0	0.740833	−1.400668	2.948773
16	6	0	−0.518456	−1.553957	−0.367956
17	6	0	0.611568	−2.207728	−1.114678
18	8	0	0.595693	−2.433781	−2.319586
19	6	0	−1.611716	−1.192111	−1.06347
20	6	0	−2.784535	−0.585445	−0.534215
21	7	0	−2.962354	−0.388766	0.813352
22	6	0	−4.137953	0.18832	0.92088
23	7	0	−4.724736	0.363489	−0.301461
24	6	0	−3.868444	−0.132984	−1.247089
25	1	0	−0.226941	3.408183	2.383642

26	1	0	−0.589791	4.686404	0.300249
27	1	0	0.434207	3.93317	−1.839066
28	1	0	1.158303	1.36196	2.382645
29	1	0	2.055001	1.89369	−2.886455
30	1	0	3.261575	−0.158621	−2.126
31	1	0	3.102422	−0.324315	1.793398
32	1	0	3.873896	−1.095836	0.42122
33	1	0	2.492022	−2.729834	1.522887
34	1	0	2.42484	−3.008575	−0.867483
35	1	0	−1.235332	−0.999722	1.474699
36	1	0	−1.613989	−1.367011	−2.137363
37	1	0	−4.611691	0.498138	1.842996
38	1	0	−5.628148	0.784541	−0.469236
39	1	0	−4.100027	−0.114184	−2.30242

Table S23. Boltzmann population [a] and optical rotation (OR) of low-energy conformers of 11*R*-**3**

Conformers	In MeOH	
	Boltzmann population (%)	OR
11*R*-**3a**	11.18	164.34
11*R*-**3b**	10.43	62.44
11*R*-**3c**	44.18	808.04
11*R*-**3d**	4.73	358.45
11*R*-**3e**	29.49	521.39
Average		552.62

[a] From Δ*G* values at 298.15K.

Table S24. Cartesian coordinates for the low-energy reoptimized MMFF conformers of 11*R*-**3** at B3LYP/6-311+G (d, p) level of theory in MeOH

11*R*-**3a**	Standard Orientation a(Ångstroms)				
No.	Atom	Type	X	Y	Z
1	6	0	3.1916	3.045	2.4749
2	6	0	2.4892	3.7842	3.4232
3	6	0	1.1547	4.1425	3.2123
4	6	0	0.5529	3.7297	2.0175
5	6	0	1.2276	2.9855	1.0496
6	6	0	2.5745	2.6404	1.2855
7	7	0	−0.723	3.9404	1.5504
8	6	0	−0.8824	3.3499	0.3259
9	6	0	0.3078	2.7397	−0.0204

10	6	0	0.5803	1.9664	−1.2772
11	6	0	0.5399	0.4468	−1.0624
12	6	0	0.9903	−0.2786	−2.3331
13	7	0	−0.7823	−0.0207	−0.6599
14	7	0	0.5854	−1.5875	−2.4378
15	8	0	1.7942	0.1825	−3.1403
16	6	0	−0.3331	−2.2148	−1.6092
17	6	0	−1.031	−1.3685	−0.5638
18	8	0	−1.7945	−1.777	0.2993
19	6	0	−0.5566	−3.5293	−1.7984
20	6	0	−1.4526	−4.4349	−1.1742
21	7	0	−1.0757	−5.7478	−1.042
22	6	0	−2.0988	−6.3439	−0.4725
23	7	0	−3.1322	−5.4746	−0.267
24	6	0	−2.7454	−4.2545	−0.7499
25	1	0	4.2302	2.7802	2.6574
26	1	0	2.9861	4.0901	4.3411
27	1	0	0.6116	4.7213	3.9521
28	1	0	3.1367	2.0691	0.5528
29	1	0	−1.4344	4.4606	2.0452
30	1	0	−1.8282	3.4156	−0.1963
31	1	0	1.5614	2.2705	−1.6639
32	1	0	−0.1508	2.2519	−2.0456
33	1	0	1.2377	0.1521	−0.2674
34	1	0	−1.2015	0.4745	0.1194
35	1	0	0.9883	−2.0957	−3.2156
36	1	0	0.0597	−4.0292	−2.5439
37	1	0	−2.1512	−7.3874	−0.1925
38	1	0	−4.0232	−5.6973	0.1544
39	1	0	−3.4034	−3.3975	−0.7277
11*R*-**3b**	Standard Orientation (Ångstroms)				
No.	Atom	Type	X	Y	Z
1	6	0	−2.2396	3.8335	1.0979
2	6	0	−1.9285	4.3668	2.3459
3	6	0	−0.6278	4.3002	2.853
4	6	0	0.3459	3.6774	2.063
5	6	0	0.0668	3.1324	0.8097
6	6	0	−1.2534	3.2158	0.3202
7	7	0	1.6841	3.4748	2.3041
8	6	0	2.2594	2.8109	1.2543
9	6	0	1.2857	2.5759	0.3027
10	6	0	1.4785	1.8709	−1.008
11	6	0	1.3634	0.345	−0.8877
12	6	0	1.7468	−0.3192	−2.2138

13	7	0	0.0294	−0.0877	−0.4909
14	7	0	1.2986	−1.6088	−2.3731
15	8	0	2.5402	0.1599	−3.0208
16	6	0	0.3984	−2.2547	−1.5385
17	6	0	−0.3077	−1.4188	−0.4888
18	8	0	−1.1712	−1.8151	0.2807
19	6	0	0.1598	−3.5614	−1.7615
20	6	0	−0.6749	−4.4985	−1.0998
21	7	0	−1.2365	−5.5121	−1.8353
22	6	0	−1.9167	−6.2295	−0.9699
23	7	0	−1.7841	−5.7399	0.2983
24	6	0	−0.9553	−4.6532	0.2349
25	1	0	−3.2577	3.8967	0.7213
26	1	0	−2.7086	4.8436	2.9352
27	1	0	−0.3888	4.7189	3.8251
28	1	0	−1.5109	2.8057	−0.6522
29	1	0	2.1668	3.777	3.139
30	1	0	3.3135	2.5665	1.2718
31	1	0	2.4668	2.1439	−1.401
32	1	0	0.7498	2.2453	−1.7386
33	1	0	2.0616	−0.032	−0.1285
34	1	0	−0.3959	0.3993	0.2912
35	1	0	1.6671	−2.0906	−3.1841
36	1	0	0.6711	−4.015	−2.6091
37	1	0	−2.5127	−7.1031	−1.1976
38	1	0	−2.212	−6.1195	1.1313
39	1	0	−0.6715	−4.1011	1.1193

11*R*-**3c**			Standard Orientation (Ångstroms)		
No.	Atom	Type	X	Y	Z
1	6	0	3.3267	2.8519	2.3451
2	6	0	2.7108	3.5362	3.3899
3	6	0	1.3725	3.9298	3.3024
4	6	0	0.6769	3.6093	2.1304
5	6	0	1.2636	2.9216	1.0679
6	6	0	2.6164	2.5392	1.1802
7	7	0	−0.6244	3.8733	1.7734
8	6	0	−0.8848	3.3699	0.5274
9	6	0	0.2636	2.7644	0.0547
10	6	0	0.4266	2.0733	−1.2673
11	6	0	0.3701	0.5439	−1.1499
12	6	0	0.7019	−0.1041	−2.4971
13	7	0	−0.9271	0.0708	−0.6843
14	7	0	0.2843	−1.4064	−2.6375
15	8	0	1.4375	0.4012	−3.3419

16	6	0	−0.5492	−2.0814	−1.7575
17	6	0	−1.2239	−1.2696	−0.6687
18	8	0	−2.0348	−1.6916	0.1433
19	6	0	−0.7586	−3.3947	−1.9704
20	6	0	−1.5256	−4.3573	−1.2649
21	7	0	−2.0994	−5.3856	−1.9695
22	6	0	−2.7047	−6.1253	−1.0679
23	7	0	−2.5113	−5.6345	0.1921
24	6	0	−1.7214	−4.5227	0.0835
25	1	0	4.3702	2.5594	2.432
26	1	0	3.2796	3.7715	4.2866
27	1	0	0.897	4.4665	4.1168
28	1	0	3.1118	2.011	0.371
29	1	0	−1.2867	4.3713	2.352
30	1	0	−1.8642	3.4872	0.0816
31	1	0	1.3831	2.3862	−1.7054
32	1	0	−0.3525	2.4217	−1.9586
33	1	0	1.1185	0.1845	−0.4316
34	1	0	−1.3216	0.5456	0.1199
35	1	0	0.6239	−1.8756	−3.4682
36	1	0	−0.2806	−3.8318	−2.8456
37	1	0	−3.2863	−7.0162	−1.2629
38	1	0	−2.8763	−6.0292	1.0477
39	1	0	−1.4017	−3.9639	0.9514

11*R*-**3d**			Standard Orientation (Ångstroms)		
No.	Atom	Type	X	Y	Z
1	6	0	−3.1807	1.7433	0.7715
2	6	0	−3.5247	3.0486	1.1115
3	6	0	−2.6659	4.1144	0.83
4	6	0	−1.4509	3.8205	0.1991
5	6	0	−1.0753	2.5236	−0.1534
6	6	0	−1.9633	1.4677	0.1381
7	7	0	−0.4334	4.6581	−0.1921
8	6	0	0.577	3.9378	−0.7689
9	6	0	0.2186	2.6033	−0.7632
10	6	0	1.0418	1.4686	−1.2968
11	6	0	2.0185	0.9082	−0.25
12	6	0	3.0381	0.0179	−0.9558
13	7	0	1.3477	0.1692	0.8147
14	7	0	2.7259	−1.3163	−1.0274
15	8	0	4.0032	0.4648	−1.5729
16	6	0	1.6782	−1.9345	−0.3625
17	6	0	0.8621	−1.0973	0.6013
18	8	0	−0.1399	−1.4657	1.1972

19	6	0	1.4608	−3.241	−0.6066
20	6	0	0.5258	−4.159	−0.063
21	7	0	0.0362	−5.1558	−0.8685
22	6	0	−0.7627	−5.8592	−0.0979
23	7	0	−0.7778	−5.3757	1.1797
24	6	0	0.0761	−4.308	1.2253
25	1	0	−3.8609	0.9259	0.9987
26	1	0	−4.4758	3.2423	1.602
27	1	0	−2.938	5.1311	1.0939
28	1	0	−1.7145	0.4417	−0.1193
29	1	0	−0.4387	5.6611	−0.0685
30	1	0	1.4644	4.436	−1.1377
31	1	0	1.6014	1.8299	−2.1702
32	1	0	0.3793	0.6789	−1.6735
33	1	0	2.572	1.7143	0.2461
34	1	0	0.7388	0.7126	1.4172
35	1	0	3.3545	−1.8694	−1.5971
36	1	0	2.0801	−3.708	−1.3706
37	1	0	−1.3451	−6.7184	−0.4021
38	1	0	−1.3135	−5.7482	1.9514
39	1	0	0.2606	−3.763	2.1402
11*R*-**3e**	Standard Orientation (Ångstroms)				
No.	Atom	Type	X	Y	Z
1	6	0	0.9822	5.4493	−0.3797
2	6	0	−0.1256	6.023	0.2391
3	6	0	−1.2565	5.2595	0.5424
4	6	0	−1.2328	3.9015	0.203
5	6	0	−0.1385	3.2969	−0.4162
6	6	0	0.9893	4.0898	−0.7126
7	7	0	−2.1889	2.9286	0.3771
8	6	0	−1.7354	1.7291	−0.1006
9	6	0	−0.4626	1.9152	−0.6039
10	6	0	0.4108	0.863	−1.2172
11	6	0	1.4452	0.3043	−0.2266
12	6	0	2.3772	−0.6424	−0.9776
13	7	0	0.832	−0.3832	0.9056
14	7	0	2.0072	−1.9645	−0.9911
15	8	0	3.3116	−0.249	−1.6729
16	6	0	0.9804	−2.5206	−0.2421
17	6	0	0.2657	−1.6243	0.7498
18	8	0	−0.7158	−1.9257	1.4135
19	6	0	0.6931	−3.8221	−0.4346
20	6	0	−0.2392	−4.6852	0.1965
21	7	0	−0.8166	−5.6859	−0.5435

22	6	0	−1.5894	−6.3342	0.2987
23	7	0	−1.5041	−5.8122	1.5582
24	6	0	−0.6105	−4.7773	1.5147
25	1	0	1.8507	6.062	−0.6091
26	1	0	−0.1126	7.0816	0.4889
27	1	0	−2.1188	5.7104	1.0225
28	1	0	1.8592	3.6568	−1.1981
29	1	0	−3.0938	3.0832	0.7997
30	1	0	−2.3553	0.8431	−0.0403
31	1	0	0.9238	1.291	−2.0884
32	1	0	−0.2204	0.0552	−1.6109
33	1	0	2.0561	1.1065	0.2031
34	1	0	0.2906	0.2014	1.5322
35	1	0	2.5715	−2.5578	−1.5872
36	1	0	1.2402	−4.3346	−1.2242
37	1	0	−2.2214	−7.179	0.0601
38	1	0	−2.0017	−6.1406	2.374
39	1	0	−0.3471	−4.2128	2.3979

Table S25. Boltzmann populations [a] and optical rotation (OR) of low-energy conformers of 11*S*-**3**

Conformers	In MeOH	
	Boltzmann population (%)	*OR*
11*S*-**3a**	11.18	−164.58
11*S*-**3b**	10.39	−62.2
11*S*-**3c**	44.25	−808.04
11*S*-**3d**	4.77	−358.28
11*S*-**3e**	29.41	-521.52
Average		-552.95

[a] From Δ*G* values at 298.15K.

Table S26. Cartesian coordinates for the low-energy reoptimized MMFF conformers of 11*S*-**3** at B3LYP/6-311+G (d, p) level of theory in MeOH

11*S*-**3a**			Standard Orientation a(Ångstroms)		
No.	Atom	Type	X	Y	Z
1	6	0	−3.0868	3.8654	−1.0553
2	6	0	−3.5729	4.4104	0.1304
3	6	0	−2.8087	4.3855	1.3004
4	6	0	−1.5423	3.7922	1.2359
5	6	0	−1.0281	3.2343	0.0653

6	6	0	−1.8181	3.2759	−1.1022
7	7	0	−0.5882	3.6343	2.2134
8	6	0	0.5075	2.9899	1.7057
9	6	0	0.2723	2.7183	0.3717
10	6	0	1.2031	2.0149	−0.5722
11	6	0	0.8158	0.5483	−0.8085
12	6	0	1.6959	−0.0609	−1.9031
13	7	0	0.9043	−0.251	0.4089
14	7	0	1.7711	−1.4329	−1.9015
15	8	0	2.178	0.58	−2.8344
16	6	0	1.2918	−2.2642	−0.9002
17	6	0	0.7301	−1.6124	0.3473
18	8	0	0.1885	−2.1902	1.2787
19	6	0	1.3718	−3.5945	−1.0944
20	6	0	1.0315	−4.693	−0.2635
21	7	0	0.584	−5.8492	−0.8516
22	6	0	0.3857	−6.6861	0.1418
23	7	0	0.7228	−6.1263	1.3413
24	6	0	1.1773	−4.8604	1.091
25	1	0	−3.6963	3.8989	−1.955
26	1	0	−4.561	4.8648	0.1468
27	1	0	−3.1896	4.814	2.2216
28	1	0	−1.4501	2.8601	−2.0354
29	1	0	−0.6882	3.954	3.1669
30	1	0	1.3625	2.7812	2.3358
31	1	0	2.2278	2.0694	−0.1806
32	1	0	1.2117	2.5645	−1.5222
33	1	0	−0.2213	0.4766	−1.1621
34	1	0	0.4274	0.132	1.2178
35	1	0	2.2511	−1.8328	−2.6985
36	1	0	1.7399	−3.9274	−2.0635
37	1	0	0.0089	−7.6964	0.0561
38	1	0	0.6585	−6.5721	2.2458
39	1	0	1.5322	−4.215	1.8818

11*S*-**3b**			Standard Orientation (Ångstroms)		
No.	Atom	Type	X	Y	Z
1	6	0	0.5464	3.1427	3.2775
2	6	0	−0.6231	3.7674	3.7026
3	6	0	−1.654	4.0537	2.8032
4	6	0	−1.4679	3.6876	1.4648
5	6	0	−0.3095	3.0597	1.0071
6	6	0	0.7166	2.7852	1.9351
7	7	0	−2.2983	3.8461	0.3806
8	6	0	−1.7125	3.3306	−0.7441

9	6	0	−0.4731	2.8281	−0.3972
10	6	0	0.5065	2.1674	−1.3223
11	6	0	0.2368	0.6673	−1.5048
12	6	0	1.1521	0.0952	−2.5918
13	7	0	0.4104	−0.0884	−0.2707
14	7	0	1.31	−1.27	−2.5646
15	8	0	1.5865	0.7454	−3.5399
16	6	0	0.8746	−2.1084	−1.549
17	6	0	0.3703	−1.461	−0.2739
18	8	0	0.0116	−2.0504	0.7354
19	6	0	0.9944	−3.4369	−1.7351
20	6	0	0.6429	−4.5434	−0.9196
21	7	0	1.4005	−5.6851	−0.9993
22	6	0	0.8312	−6.5346	−0.1743
23	7	0	−0.2839	−5.9979	0.4039
24	6	0	−0.4397	−4.7347	−0.0979
25	1	0	1.3367	2.9299	3.9933
26	1	0	−0.7365	4.0383	4.7499
27	1	0	−2.5629	4.5426	3.1379
28	1	0	1.6352	2.2996	1.6181
29	1	0	−3.2089	4.2829	0.4169
30	1	0	−2.2254	3.3696	−1.6963
31	1	0	1.5274	2.322	−0.9497
32	1	0	0.4592	2.679	−2.2927
33	1	0	−0.7964	0.5028	−1.8386
34	1	0	−0.0286	0.2892	0.5626
35	1	0	1.8002	−1.658	−3.3613
36	1	0	1.4583	−3.7614	−2.6651
37	1	0	1.1728	−7.5382	0.0403
38	1	0	−0.8881	−6.4563	1.0716
39	1	0	−1.272	−4.1073	0.1873

11*S*-**3c**	Standard Orientation (Ångstroms)				
No.	Atom	Type	X	Y	Z
1	6	0	−3.0937	3.7089	−1.1715
2	6	0	−3.6326	4.26	−0.0118
3	6	0	−2.8983	4.295	1.177
4	6	0	−1.6068	3.7551	1.1582
5	6	0	−1.0397	3.1924	0.0146
6	6	0	−1.8006	3.1731	−1.1727
7	7	0	−0.6718	3.6621	2.1621
8	6	0	0.4626	3.0531	1.6975
9	6	0	0.273	2.7398	0.3654
10	6	0	1.2566	2.0541	−0.5371
11	6	0	0.9357	0.5678	−0.7466

12	6	0	1.8733	−0.0344	−1.7969
13	7	0	1.0266	−0.199	0.4893
14	7	0	1.9761	−1.4052	−1.7709
15	8	0	2.3714	0.603	−2.7219
16	6	0	1.4635	−2.2299	−0.78
17	6	0	0.9321	−1.5686	0.477
18	8	0	0.5096	−2.1463	1.4684
19	6	0	1.5361	−3.5614	−0.969
20	6	0	1.1021	−4.6549	−0.1761
21	7	0	1.8154	−5.8263	−0.2248
22	6	0	1.1721	−6.6543	0.5671
23	7	0	0.053	−6.074	1.0932
24	6	0	−0.0259	−4.8036	0.5916
25	1	0	−3.6804	3.6964	−2.0868
26	1	0	−4.6387	4.6729	−0.031
27	1	0	−3.32	4.7289	2.0777
28	1	0	−1.391	2.7527	−2.0864
29	1	0	−0.8087	4.0004	3.1046
30	1	0	1.3095	2.8966	2.3532
31	1	0	2.2676	2.1607	−0.1213
32	1	0	1.2674	2.5804	−1.5002
33	1	0	−0.0873	0.444	−1.1255
34	1	0	0.5681	0.1954	1.303
35	1	0	2.4832	−1.8093	−2.5487
36	1	0	2.0259	−3.9012	−1.8801
37	1	0	1.4612	−7.6719	0.7925
38	1	0	−0.6017	−6.5096	1.7277
39	1	0	−0.8446	−4.143	0.839

11*S*-**3d**			Standard Orientation (Ångstroms)		
No.	Atom	Type	X	Y	Z
1	6	0	0.7974	0.6992	3.5533
2	6	0	0.7777	1.8363	4.3562
3	6	0	0.8025	3.1145	3.7921
4	6	0	0.8445	3.2077	2.3956
5	6	0	0.8637	2.0883	1.5626
6	6	0	0.8428	0.8105	2.1588
7	7	0	0.878	4.3174	1.5849
8	6	0	0.9063	3.9416	0.2694
9	6	0	0.8965	2.5611	0.2106
10	6	0	0.9152	1.7333	−1.0401
11	6	0	−0.4952	1.4744	−1.5946
12	6	0	−0.373	0.9413	−3.0198
13	7	0	−1.2692	0.5519	−0.7702
14	7	0	−0.3316	−0.4239	−3.151

15	8	0	−0.1577	1.6661	−3.9893
16	6	0	−0.5759	−1.3378	−2.1375
17	6	0	−1.0209	−0.7984	−0.7933
18	8	0	−1.193	−1.4591	0.2209
19	6	0	−0.4138	−2.6454	−2.4159
20	6	0	−0.6282	−3.8095	−1.6341
21	7	0	0.1886	−4.8944	−1.8296
22	6	0	−0.2627	−5.8166	−1.0095
23	7	0	−1.3591	−5.3801	−0.3213
24	6	0	−1.6283	−4.1066	−0.7421
25	1	0	0.777	−0.2877	4.0094
26	1	0	0.7437	1.7287	5.4379
27	1	0	0.7895	3.9989	4.4206
28	1	0	0.8582	−0.0897	1.5505
29	1	0	0.8817	5.2707	1.9202
30	1	0	0.9343	4.6883	−0.5137
31	1	0	1.4377	0.7876	−0.8474
32	1	0	1.5187	2.2592	−1.7923
33	1	0	−1.0794	2.4013	−1.6381
34	1	0	−1.5048	0.8786	0.1608
35	1	0	−0.1349	−0.7545	−4.0879
36	1	0	−0.0276	−2.8936	−3.4031
37	1	0	0.1535	−6.8059	−0.8745
38	1	0	−1.8813	−5.9078	0.3641
39	1	0	−2.4706	−3.5458	−0.3625

11*S*-**3e**			Standard Orientation (Ångstroms)		
No.	Atom	Type	X	Y	Z
1	6	0	5.712965	0.698866	0.303301
2	6	0	6.219716	−0.594742	0.399344
3	6	0	5.410167	−1.705643	0.14591
4	6	0	4.075674	−1.469559	−0.205335
5	6	0	3.537665	−0.186442	−0.308795
6	6	0	4.376099	0.917262	−0.049413
7	7	0	3.069344	−2.356778	−0.507156
8	6	0	1.912525	−1.681969	−0.788179
9	6	0	2.161492	−0.327837	−0.676678
10	6	0	1.170694	0.776143	−0.889964
11	6	0	0.581871	1.303156	0.428716
12	6	0	−0.293916	2.514473	0.121702
13	7	0	−0.187179	0.293346	1.149538
14	7	0	−1.622242	2.253059	−0.107236
15	8	0	0.164309	3.637539	−0.078428
16	6	0	−2.248487	1.031597	0.093969
17	6	0	−1.431459	−0.086635	0.710361

18	8	0	−1.797225	−1.242883	0.865486
19	6	0	−3.543448	0.921485	−0.258678
20	6	0	−4.466527	−0.148582	−0.134959
21	7	0	−5.436586	−0.296401	−1.093855
22	6	0	−6.153611	−1.32545	−0.701744
23	7	0	−5.704957	−1.82226	0.489073
24	6	0	−4.646131	−1.051063	0.883499
25	1	0	6.360813	1.549155	0.502111
26	1	0	7.261658	−0.743984	0.673477
27	1	0	5.808972	−2.712031	0.219442
28	1	0	3.995674	1.93191	−0.123622
29	1	0	3.174742	−3.361932	−0.51628
30	1	0	1.008431	−2.221768	−1.041844
31	1	0	0.371233	0.419817	−1.553136
32	1	0	1.662764	1.595066	−1.430838
33	1	0	1.373051	1.625779	1.115239
34	1	0	0.344827	−0.494785	1.500418
35	1	0	−2.164162	3.048974	−0.421086
36	1	0	−3.993759	1.782075	−0.750458
37	1	0	−6.999291	−1.748089	−1.227399
38	1	0	−6.093933	−2.608273	0.991005
39	1	0	−4.128079	−1.226285	1.81561

Table S27. Gibbs free energies [a] and equilibrium populations [b] of low-energy conformers of 11*R*-**3**

Conformers	In MeOH	
	G	P (%)
11*R*-**3a**	−1081.1530967	3.29%
11*R*-**3b**	−1081.154545	15.27%
11*R*-**3c**	−1081.1544924	14.44%
11*R*-**3d**	−1081.1558426	60.44%
11*R*-**3e**	−1081.1537464	6.55%

[a] B3LYP/6-31+g (d, p), in kcal/mol; [b] from G values at 298.15K.

Table S28. Cartesian coordinates for the low-energy reoptimized MMFF conformers of 11*R*-**3** at B3LYP/6-31+g (d, p) level of theory in MeOH

11*R*-**3a**			Standard Orientation (Ångstroms)		
No.	Atom	Type	X	Y	Z
1	6	0	−3.917693	−2.220124	−1.551121
2	6	0	−4.833637	−2.644676	−0.592059
3	6	0	−5.075062	−1.887179	0.557455
4	6	0	−4.362637	−0.691405	0.708444
5	6	0	−3.438055	−0.238758	−0.232794

6	6	0	−3.214903	−1.021315	−1.384823
7	7	0	−4.399281	0.238564	1.720399
8	6	0	−3.524055	1.25796	1.458591
9	6	0	−2.902181	0.99804	0.252553
10	6	0	−1.863154	1.852984	−0.411729
11	6	0	−0.442501	1.568106	0.095593
12	6	0	0.539115	2.589699	−0.486163
13	7	0	0.002684	0.213759	−0.215213
14	7	0	1.858533	2.206428	−0.48196
15	8	0	0.235504	3.741588	−0.788531
16	6	0	2.325893	0.934322	−0.188437
17	6	0	1.29986	−0.154979	0.049538
18	8	0	1.537513	−1.297688	0.412868
19	6	0	3.658928	0.7462	−0.150533
20	6	0	4.442991	−0.417631	0.058348
21	7	0	5.656686	−0.289955	0.685802
22	6	0	6.162299	−1.502594	0.706579
23	7	0	5.338815	−2.397929	0.085249
24	6	0	4.250009	−1.706834	−0.371242
25	1	0	−3.745598	−2.823977	−2.438706
26	1	0	−5.37066	−3.579013	−0.739135
27	1	0	−5.790608	−2.220403	1.302002
28	1	0	−2.504091	−0.702253	−2.141569
29	1	0	−4.990486	0.171914	2.537248
30	1	0	−3.420606	2.082951	2.151359
31	1	0	−2.122054	2.905385	−0.235366
32	1	0	−1.908072	1.709882	−1.499098
33	1	0	−0.399866	1.675282	1.187856
34	1	0	−0.636369	−0.530012	0.046731
35	1	0	2.512151	2.930978	−0.752929
36	1	0	4.282915	1.629211	−0.278594
37	1	0	7.107658	−1.789566	1.1471
38	1	0	5.51006	−3.387544	−0.025914
39	1	0	3.458127	−2.190998	−0.924824

	11*R*-**3b**		Standard Orientation (Ångstroms)		
No.	Atom	Type	X	Y	Z
1	6	0	−4.766105	−0.203346	2.25035
2	6	0	−5.482035	−1.3047	1.788368
3	6	0	−5.293391	−1.793747	0.492622
4	6	0	−4.359556	−1.138454	−0.318883
5	6	0	−3.623968	−0.035458	0.114976
6	6	0	−3.836116	0.440185	1.425583
7	7	0	−3.980471	−1.394001	−1.615518

8	6	0	−3.026028	−0.49785	−2.015101
9	6	0	−2.771547	0.362936	−0.964723
10	6	0	−1.787807	1.496121	−0.959405
11	6	0	−0.484472	1.15023	−0.225478
12	6	0	0.401593	2.393229	−0.107955
13	7	0	0.249087	0.071353	−0.878389
14	7	0	1.735941	2.145919	0.107453
15	8	0	−0.03024	3.541537	−0.035317
16	6	0	2.347489	0.903647	0.027798
17	6	0	1.50489	−0.267521	−0.435526
18	8	0	1.859118	−1.436956	−0.475374
19	6	0	3.652435	0.822495	0.350916
20	6	0	4.567331	−0.261328	0.322419
21	7	0	5.572757	−0.298573	1.255566
22	6	0	6.270608	−1.373855	0.966755
23	7	0	5.77587	−2.008801	−0.136774
24	6	0	4.706516	−1.282354	−0.584222
25	1	0	−4.931093	0.163047	3.260598
26	1	0	−6.200946	−1.79105	2.443974
27	1	0	−5.855446	−2.650266	0.135018
28	1	0	−3.289372	1.3014	1.797868
29	1	0	−4.359169	−2.137123	−2.186126
30	1	0	−2.610426	−0.546925	−3.013329
31	1	0	−2.267064	2.367287	−0.494572
32	1	0	−1.564323	1.788718	−1.994175
33	1	0	−0.696852	0.816918	0.798973
34	1	0	−0.291641	−0.761446	−1.084682
35	1	0	2.293274	2.96745	0.307345
36	1	0	4.121479	1.729631	0.728397
37	1	0	7.133336	−1.736414	1.50939
38	1	0	6.141953	−2.854282	−0.551891
39	1	0	4.153946	−1.566237	−1.468586

	11*R*-**3c**		Standard Orientation (Ångstroms)		
No.	Atom	Type	X	Y	Z
1	6	0	−3.956944	−2.09458	−1.700437
2	6	0	−4.85896	−2.585242	−0.759963
3	6	0	−5.077943	−1.91339	0.4459
4	6	0	−4.357093	−0.734834	0.672596
5	6	0	−3.44571	−0.217586	−0.24802
6	6	0	−3.245885	−0.913622	−1.458349
7	7	0	−4.37327	0.118872	1.750147
8	6	0	−3.49663	1.150933	1.550052
9	6	0	−2.895581	0.977983	0.318098

10	6	0	−1.864406	1.876366	−0.29941
11	6	0	−0.435907	1.546027	0.154734
12	6	0	0.541295	2.60497	−0.365236
13	7	0	−0.000982	0.223931	−0.277979
14	7	0	1.860818	2.22088	−0.390965
15	8	0	0.237606	3.775823	−0.582636
16	6	0	2.326347	0.932526	−0.172994
17	6	0	1.299592	−0.181233	−0.105323
18	8	0	1.542749	−1.37324	0.016951
19	6	0	3.658876	0.747781	−0.107076
20	6	0	4.444802	−0.412896	0.111957
21	7	0	5.680765	−0.490412	−0.479529
22	6	0	6.182547	−1.637143	−0.079914
23	7	0	5.334744	−2.282247	0.774919
24	6	0	4.233372	−1.487406	0.939413
25	1	0	−3.801981	−2.632084	−2.632864
26	1	0	−5.402697	−3.504314	−0.966671
27	1	0	−5.783367	−2.297752	1.175328
28	1	0	−2.546642	−0.542517	−2.20226
29	1	0	−4.953389	−0.004387	2.568431
30	1	0	−3.378674	1.92293	2.299311
31	1	0	−2.1125	2.913239	−0.036477
32	1	0	−1.932729	1.818548	−1.393479
33	1	0	−0.370172	1.562068	1.250823
34	1	0	−0.647274	−0.543253	−0.124603
35	1	0	2.516459	2.963281	−0.602341
36	1	0	4.28338	1.625952	−0.262805
37	1	0	7.141524	−2.046238	−0.368111
38	1	0	5.498241	−3.177864	1.213242
39	1	0	3.421822	−1.767368	1.595631

	11*R*-**3d**		Standard Orientation (Ångstroms)		
No.	Atom	Type	X	Y	Z
1	6	0	−4.688651	−0.537292	2.237837
2	6	0	−5.412051	−1.562548	1.634014
3	6	0	−5.25446	−1.851295	0.27563
4	6	0	−4.343634	−1.075444	−0.451409
5	6	0	−3.601078	−0.044451	0.124387
6	6	0	−3.781757	0.228693	1.496083
7	7	0	−3.997256	−1.131003	−1.780922
8	6	0	−3.056138	−0.178617	−2.065063
9	6	0	−2.77753	0.516831	−0.904254
10	6	0	−1.797033	1.642911	−0.754052
11	6	0	−0.475646	1.199248	−0.111229

12	6	0	0.415609	2.414113	0.161757
13	7	0	0.245684	0.23663	−0.933932
14	7	0	1.75041	2.132576	0.333484
15	8	0	−0.010871	3.542459	0.396299
16	6	0	2.348604	0.90377	0.09526
17	6	0	1.519855	−0.15528	−0.605598
18	8	0	1.910815	−1.261643	−0.949408
19	6	0	3.642999	0.756715	0.437232
20	6	0	4.526843	−0.349029	0.343649
21	7	0	5.86158	−0.108029	0.134627
22	6	0	6.428639	−1.293334	0.128505
23	7	0	5.5177	−2.283832	0.36275
24	6	0	4.299648	−1.687778	0.543655
25	1	0	−4.830316	−0.326228	3.294967
26	1	0	−6.113223	−2.145986	2.226593
27	1	0	−5.822912	−2.648219	−0.192453
28	1	0	−3.229617	1.02946	1.978978
29	1	0	−4.388499	−1.782348	−2.447405
30	1	0	−2.666157	−0.074598	−3.069469
31	1	0	−2.266389	2.432252	−0.152837
32	1	0	−1.60002	2.087615	−1.738754
33	1	0	−0.661054	0.716983	0.857438
34	1	0	−0.293925	−0.53653	−1.306644
35	1	0	2.31314	2.9128	0.649995
36	1	0	4.143118	1.630982	0.850676
37	1	0	7.478919	−1.494612	−0.034177
38	1	0	5.712569	−3.274335	0.405856
39	1	0	3.412673	−2.261445	0.771631

	11*R*-**3e**		Standard Orientation (Ångstroms)		
No.	Atom	Type	X	Y	Z
1	6	0	−2.521089	−3.000259	−0.281262
2	6	0	−3.868583	−3.191178	0.011562
3	6	0	−4.739849	−2.104575	0.126027
4	6	0	−4.207916	−0.822848	−0.060955
5	6	0	−2.862289	−0.597094	−0.35386
6	6	0	−2.006595	−1.712104	−0.46837
7	7	0	−4.823729	0.405195	−0.010212
8	6	0	−3.915267	1.40024	−0.249125
9	6	0	−2.680304	0.819459	−0.46625
10	6	0	−1.403742	1.549887	−0.760367
11	6	0	−0.656629	1.965451	0.517563
12	6	0	0.419892	2.982986	0.148895
13	7	0	−0.06673	0.833139	1.224312

14	7	0	1.663757	2.472104	−0.12423
15	8	0	0.175985	4.170147	−0.058232
16	6	0	2.05902	1.160081	0.086334
17	6	0	1.075682	0.226832	0.763083
18	8	0	1.235346	−0.971789	0.943274
19	6	0	3.294535	0.802628	−0.312994
20	6	0	4.005932	−0.419315	−0.198258
21	7	0	4.882637	−0.76752	−1.194452
22	6	0	5.41152	−1.905501	−0.804508
23	7	0	4.936602	−2.283936	0.419163
24	6	0	4.062826	−1.318009	0.837539
25	1	0	−1.85731	−3.857373	−0.365946
26	1	0	−4.249297	−4.200085	0.1522
27	1	0	−5.790085	−2.256676	0.351924
28	1	0	−0.951888	−1.585343	−0.696921
29	1	0	−5.807015	0.544622	0.176615
30	1	0	−4.224864	2.437373	−0.24782
31	1	0	−1.645908	2.441305	−1.354748
32	1	0	−0.764294	0.929522	−1.401121
33	1	0	−1.334033	2.446632	1.233017
34	1	0	−0.720999	0.163142	1.614496
35	1	0	2.331511	3.146678	−0.477528
36	1	0	3.875821	1.554113	−0.844639
37	1	0	6.13544	−2.491451	−1.354683
38	1	0	5.194325	−3.119227	0.925952
39	1	0	3.567335	−1.373052	1.79646

Table S29. Gibbs free energies [a] and equilibrium populations [b] of low-energy conformers of 11*S*-**3**

Conformers	In MeOH	
	G	*P* (%)
11*S*-**3a**	−1081.1530917	3.27%
11*S*-**3b**	−1081.1545526	15.37%
11*S*-**3c**	−1081.1544858	14.35%
11*S*-**3d**	−1081.1558427	60.49%
11*S*-**3e**	−1081.1537412	6.52%

[a] B3LYP/6-31+g (d, p), in kcal/mol; b from G values at 298.15K

Table S30. Cartesian coordinates for the low-energy reoptimized MMFF conformers of 11*S*-**3** at B3LYP/6-31+g (d, p) level of theory in MeOH

11*S*-**3a**			Standard Orientation (Ångstroms)		
No.	Atom	Type	X	Y	Z
1	6	0	3.917726	−2.220143	−1.551103

2	6	0	4.833627	−2.644657	−0.592069
3	6	0	5.075047	−1.887153	0.557448
4	6	0	4.362635	−0.691502	0.708449
5	6	0	3.438008	−0.238785	−0.232753
6	6	0	3.214909	−1.021268	−1.384791
7	7	0	4.399255	0.238557	1.720392
8	6	0	3.523957	1.257978	1.45855
9	6	0	2.902219	0.99799	0.252536
10	6	0	1.863189	1.853009	−0.411707
11	6	0	0.442502	1.568145	0.095662
12	6	0	−0.539098	2.589746	−0.486163
13	7	0	−0.002765	0.2138	−0.215156
14	7	0	−1.858594	2.206456	−0.481969
15	8	0	−0.235504	3.741634	−0.788547
16	6	0	−2.325852	0.934322	−0.188452
17	6	0	−1.29988	−0.154942	0.049564
18	8	0	−1.537512	−1.297713	0.412787
19	6	0	−3.658898	0.746147	−0.150598
20	6	0	−4.442911	−0.417649	0.058324
21	7	0	−5.656704	−0.289961	0.685867
22	6	0	−6.162274	−1.50259	0.706621
23	7	0	−5.338781	−2.397996	0.0852
24	6	0	−4.249975	−1.706801	−0.371264
25	1	0	3.745621	−2.823987	−2.438726
26	1	0	5.370628	−3.579041	−0.739223
27	1	0	5.790608	−2.220383	1.301972
28	1	0	2.504087	−0.702187	−2.141619
29	1	0	4.990558	0.171915	2.537264
30	1	0	3.420595	2.08296	2.151398
31	1	0	1.908032	1.709853	−1.499135
32	1	0	2.122042	2.905367	−0.23537
33	1	0	0.399816	1.6752	1.187946
34	1	0	0.636422	−0.530027	0.046794
35	1	0	−2.512135	2.930934	−0.752881
36	1	0	−4.282841	1.629157	−0.278561
37	1	0	−7.107631	−1.789597	1.147059
38	1	0	−5.510071	−3.387604	−0.025902
39	1	0	−3.458052	−2.191008	−0.924782

	11*S*-**3b**		Standard Orientation (Ångstroms)		
No.	Atom	Type	X	Y	Z
1	6	0	4.766074	−0.203404	2.250384
2	6	0	5.482	−1.304833	1.788316

3	6	0	5.293396	−1.793779	0.492599
4	6	0	4.359592	−1.138419	−0.318849
5	6	0	3.623966	−0.035447	0.115048
6	6	0	3.836064	0.440184	1.42565
7	7	0	3.980337	−1.394	−1.61561
8	6	0	3.025999	−0.497764	−2.015071
9	6	0	2.771603	0.362906	−0.964752
10	6	0	1.787833	1.496165	−0.95937
11	6	0	0.484524	1.150301	−0.225532
12	6	0	−0.401567	2.393229	−0.107962
13	7	0	−0.24917	0.071313	−0.878412
14	7	0	−1.73591	2.145894	0.107435
15	8	0	0.030244	3.541594	−0.035239
16	6	0	−2.347467	0.903702	0.027763
17	6	0	−1.5048	−0.267556	−0.435566
18	8	0	−1.859168	−1.436968	−0.475405
19	6	0	−3.652374	0.822551	0.350938
20	6	0	−4.567376	−0.261359	0.322459
21	7	0	−5.57274	−0.298616	1.25561
22	6	0	−6.270587	−1.373868	0.966798
23	7	0	−5.775874	−2.00883	−0.136785
24	6	0	−4.706473	−1.282319	−0.584318
25	1	0	4.931105	0.163022	3.260616
26	1	0	6.200868	−1.791179	2.443897
27	1	0	5.855402	−2.6503	0.135071
28	1	0	3.289352	1.301429	1.797858
29	1	0	4.359197	−2.137153	−2.186188
30	1	0	2.610417	−0.546811	−3.013316
31	1	0	1.564378	1.788741	−1.994164
32	1	0	2.267168	2.367259	−0.494538
33	1	0	0.69684	0.816945	0.798941
34	1	0	0.291607	−0.761428	−1.084674
35	1	0	−2.293195	2.967459	0.307419
36	1	0	−4.121455	1.72969	0.728478
37	1	0	−7.133283	−1.736364	1.509364
38	1	0	−6.142026	−2.854186	−0.551873
39	1	0	−4.15393	−1.566222	−1.4686

	11*S*-**3c**		Standard Orientation (Ångstroms)		
No.	Atom	Type	X	Y	Z
1	6	0	4.894565	−0.094998	2.209917
2	6	0	5.614294	−1.230159	1.775817
3	6	0	5.39816	−1.787357	0.51633

4	6	0	4.435593	−1.176294	−0.298035
5	6	0	3.693108	−0.033843	0.116921
6	6	0	3.938164	0.508147	1.395664
7	7	0	4.017575	−1.498692	−1.57247
8	6	0	3.043134	−0.603166	−1.965063
9	6	0	2.804813	0.318915	−0.963756
10	6	0	1.815299	1.456768	−0.994309
11	6	0	0.529462	1.171391	−0.204764
12	6	0	−0.378715	2.393601	−0.126773
13	7	0	−0.207117	0.050306	−0.798196
14	7	0	−1.70952	2.120278	0.034296
15	8	0	0.045915	3.548698	−0.173525
16	6	0	−2.34532	0.862326	0.001199
17	6	0	−1.47923	−0.296752	−0.390419
18	8	0	−1.873019	−1.461885	−0.433891
19	6	0	−3.672055	0.80327	0.303151
20	6	0	−4.658825	−0.268733	0.317738
21	7	0	−5.719633	−0.19496	1.21456
22	6	0	−6.498939	−1.229658	0.9647
23	7	0	−6.008907	−1.965703	−0.074836
24	6	0	−4.83653	−1.380645	−0.489588
25	1	0	5.08768	0.318934	3.195398
26	1	0	6.353941	−1.680795	2.431609
27	1	0	5.954629	−2.658214	0.183039
28	1	0	3.397699	1.383981	1.744561
29	1	0	4.371959	−2.262681	−2.128942
30	1	0	2.594349	−0.688294	−2.944536
31	1	0	1.545593	1.687207	−2.031042
32	1	0	2.267628	2.362507	−0.580352
33	1	0	0.790964	0.930911	0.837692
34	1	0	0.363411	−0.771378	−0.981532
35	1	0	−2.290665	2.941936	0.159748
36	1	0	−4.104817	1.751945	0.623856
37	1	0	−7.413091	−1.485974	1.481022
38	1	0	−6.429152	−2.800623	−0.46019
39	1	0	−4.261947	−1.779428	−1.306853

	11*S*-3**d**		Standard Orientation (Ångstroms)		
No.	Atom	Type	X	Y	Z
1	6	0	4.688689	−0.537331	2.237794
2	6	0	5.41206	−1.562572	1.634049
3	6	0	5.254424	−1.851293	0.275547
4	6	0	4.343646	−1.075414	−0.451405

5	6	0	3.600991	−0.044423	0.124424
6	6	0	3.781756	0.228698	1.496088
7	7	0	3.997224	−1.131009	−1.780899
8	6	0	3.056151	−0.178588	−2.065109
9	6	0	2.77757	0.51683	−0.904256
10	6	0	1.797032	1.642934	−0.754003
11	6	0	0.475656	1.199264	−0.11128
12	6	0	−0.415647	2.414081	0.161766
13	7	0	−0.245673	0.236696	−0.933904
14	7	0	−1.750365	2.13261	0.333473
15	8	0	0.01085	3.542439	0.396332
16	6	0	−2.34871	0.90375	0.095301
17	6	0	−1.519808	−0.155312	−0.605658
18	8	0	−1.910869	−1.261666	−0.949339
19	6	0	−3.642995	0.756717	0.437223
20	6	0	−4.526831	−0.349038	0.343716
21	7	0	−5.861555	−0.108092	0.13462
22	6	0	−6.428605	−1.293284	0.128433
23	7	0	−5.51767	−2.283825	0.362735
24	6	0	−4.299565	−1.687771	0.543636
25	1	0	4.830353	−0.326314	3.294996
26	1	0	6.113297	−2.145949	2.226524
27	1	0	5.822868	−2.648226	−0.192501
28	1	0	3.229632	1.029401	1.979
29	1	0	4.388518	−1.782303	−2.447451
30	1	0	2.666164	−0.074562	−3.069497
31	1	0	1.600035	2.087705	−1.738803
32	1	0	2.266413	2.432277	−0.152835
33	1	0	0.660953	0.716945	0.857409
34	1	0	0.293905	−0.536579	−1.306595
35	1	0	−2.313132	2.912728	0.649976
36	1	0	−4.143225	1.631023	0.850702
37	1	0	−7.478952	−1.494566	−0.034194
38	1	0	−5.712592	−3.274403	0.405872
39	1	0	−3.412691	−2.261513	0.77168

	11*S*-**3e**		Standard Orientation (Ångstroms)		
No.	Atom	Type	X	Y	Z
1	6	0	2.521116	−3.000318	−0.2813
2	6	0	3.868612	−3.191166	0.011564
3	6	0	4.73982	−2.104531	0.126027
4	6	0	4.207903	−0.822775	−0.06092
5	6	0	2.862339	−0.597059	−0.353883

6	6	0	2.006627	−1.712053	−0.468366
7	7	0	4.823667	0.405115	−0.010215
8	6	0	3.915228	1.400256	−0.249193
9	6	0	2.680314	0.819431	−0.466306
10	6	0	1.40369	1.549918	−0.760317
11	6	0	0.656682	1.965387	0.517613
12	6	0	−0.419969	2.982988	0.148932
13	7	0	0.06679	0.833102	1.224321
14	7	0	−1.66373	2.4721	−0.124179
15	8	0	−0.175959	4.170154	−0.058292
16	6	0	−2.058961	1.160073	0.086285
17	6	0	−1.075645	0.226805	0.763077
18	8	0	−1.235346	−0.971821	0.943362
19	6	0	−3.294563	0.802692	−0.312966
20	6	0	−4.005972	−0.419297	−0.19825
21	7	0	−4.882644	−0.767558	−1.194416
22	6	0	−5.411576	−1.905474	−0.804558
23	7	0	−4.936609	−2.283956	0.419084
24	6	0	−4.062843	−1.317981	0.837592
25	1	0	1.857336	−3.857379	−0.365994
26	1	0	4.249284	−4.200095	0.152211
27	1	0	5.790052	−2.256689	0.351895
28	1	0	0.951907	−1.585241	−0.696916
29	1	0	5.807003	0.544601	0.176614
30	1	0	4.224866	2.437366	−0.247737
31	1	0	0.764269	0.92955	−1.401145
32	1	0	1.645923	2.441294	−1.354744
33	1	0	1.334084	2.446615	1.233006
34	1	0	0.720959	0.163127	1.614453
35	1	0	−2.331481	3.146664	−0.477537
36	1	0	−3.875813	1.554135	−0.844706
37	1	0	−6.135412	−2.491445	−1.354733
38	1	0	−5.194364	−3.119196	0.925979
39	1	0	−3.567298	−1.372973	1.796424

Figure S1. ITS rDNA sequence of *Penicillium* sp. ZZ1750

GAACCTGCGGAAGGATCATTACCGAGTGAGGGCCCTCTGGGTCCAACCTCCCACCCGTGT
TTATTTTACCTTGTTGCTTCGGCGGGCCCGCCTTAACTGGCCGCCGGGGGGCTTACGCCCC
CGGGCCCGCGCCCGCCGAAGACACCCTCGAACTCTGTCTGAAGATTGTAGTCTGAGTGA
AAATATAAATTATTTAAAACTTTCAACAACGGATCTCTTGGTTCCGGCATCGATGAAGAAC
GCAGCGAAATGCGATACGTAATGTGAATTGCAAATTCAGTGAATCATCGAGTCTTTGAAC
GCACATTGCGCCCCCTGGTATTCCGGGGGGCATGCCTGTCCGAGCGTCATTTCTGCCCTCA
AGCACGGCTTGTGTGTTGGGCCCCGTCCTCCGATCCCGGGGGACGGGCCCGAAAGGCAG
CGGCGGCACCGCGTCCGGTCCTCGAGCGTATGGGGCTTTGTCACCCGCTCTGTAGGCCCG
GCCGGCGCTTGCCGATCAACCCAAATTTTTATCCAGGTTGACCTCGGATCAGGTAGGGATA
CCCGCTGAACTTAAGCATATC (563 bp).

Figure S2. Static culture state of strain *Penicillium* sp. ZZ1750 in GA liquid medium

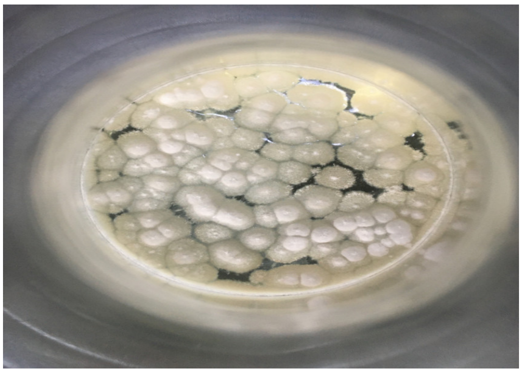

Figure S3. ^{1}H NMR spectrum of penipyridinone B (**1**, in MeOH-d_4)

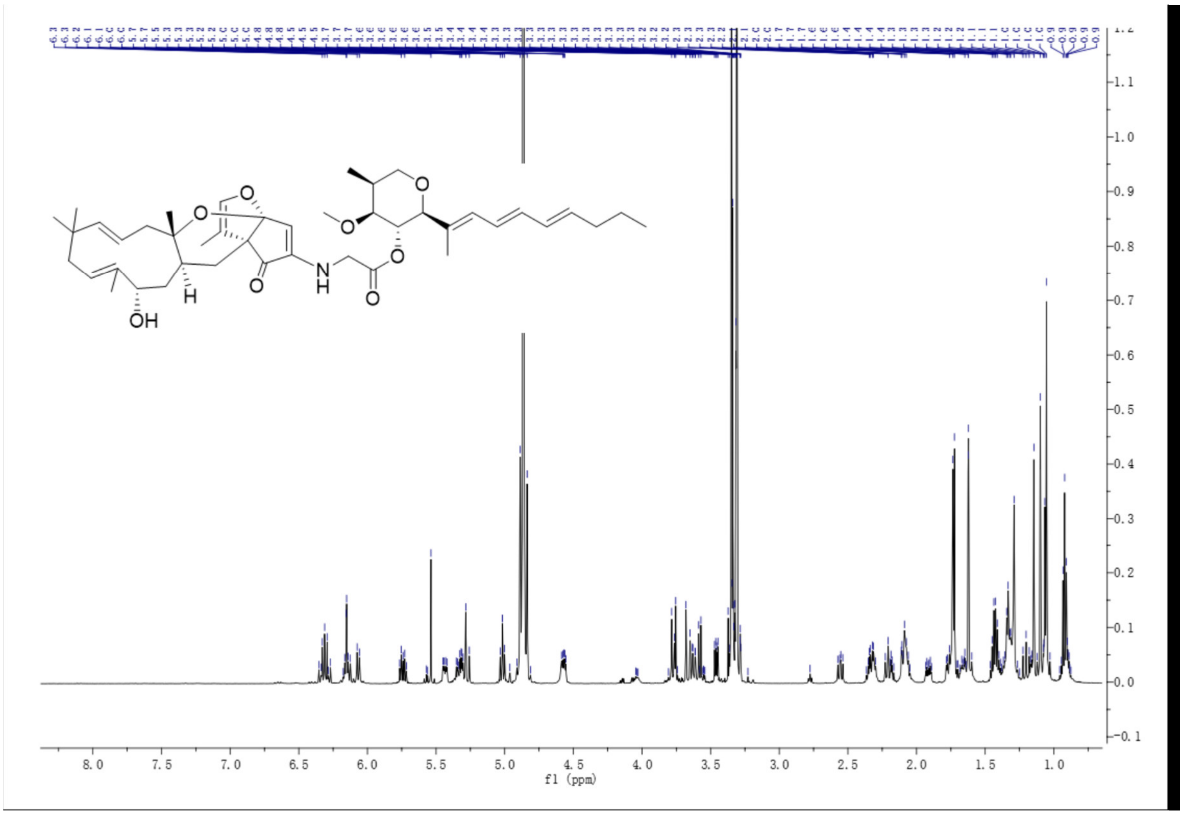

Figure S4. ^{1}H NMR spectrum of penipyridinone B (**1**, in MeOH-d_4)

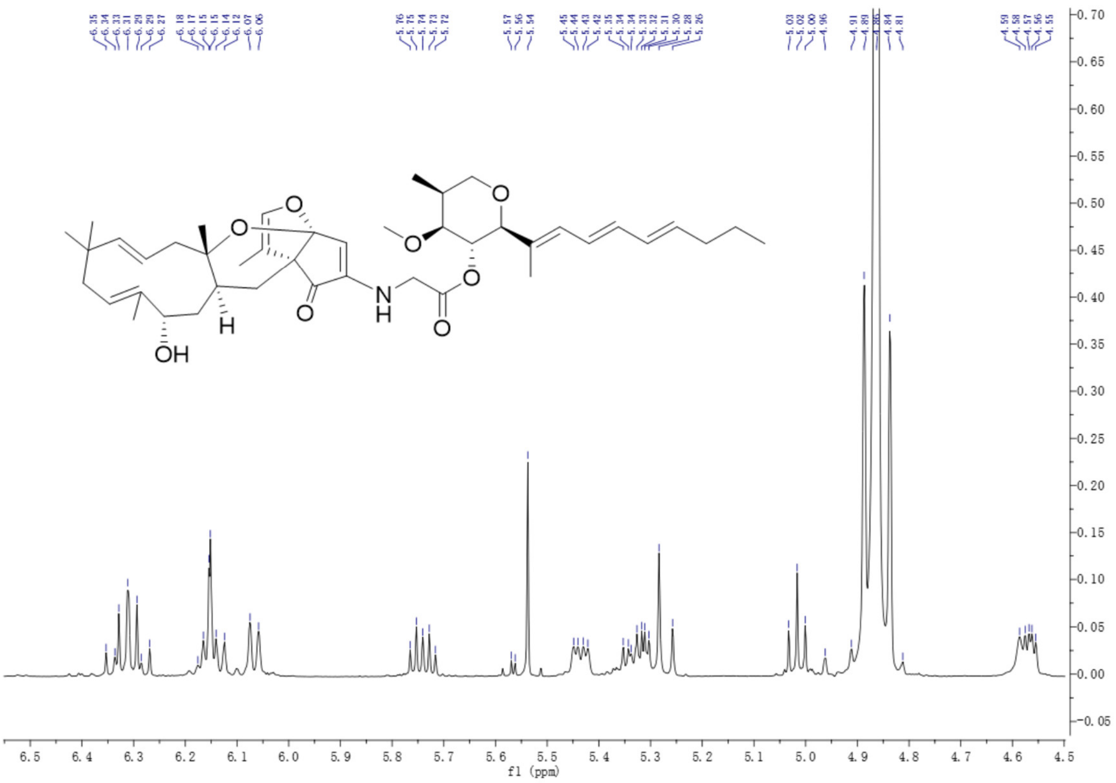

Figure S5. ^{1}H NMR spectrum of penipyridinone B (**1**, in MeOH-d_4)

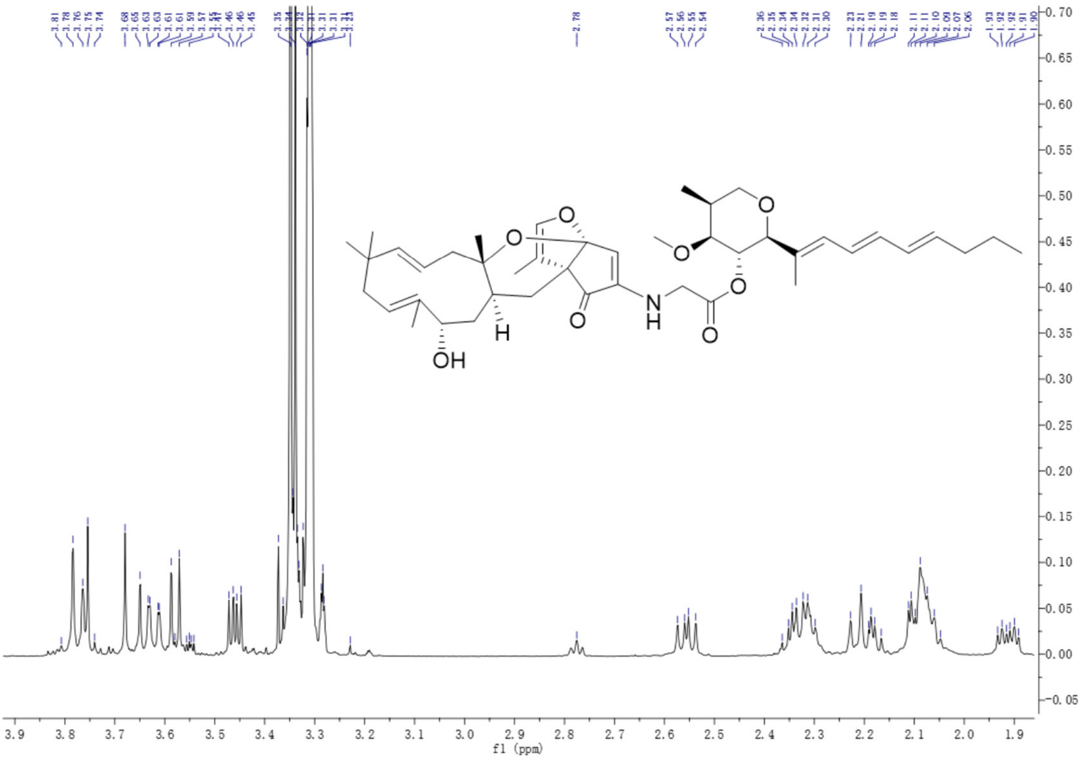

Figure S6. ^{1}H NMR spectrum of penipyridinone B (**1**, in MeOH-d_4)

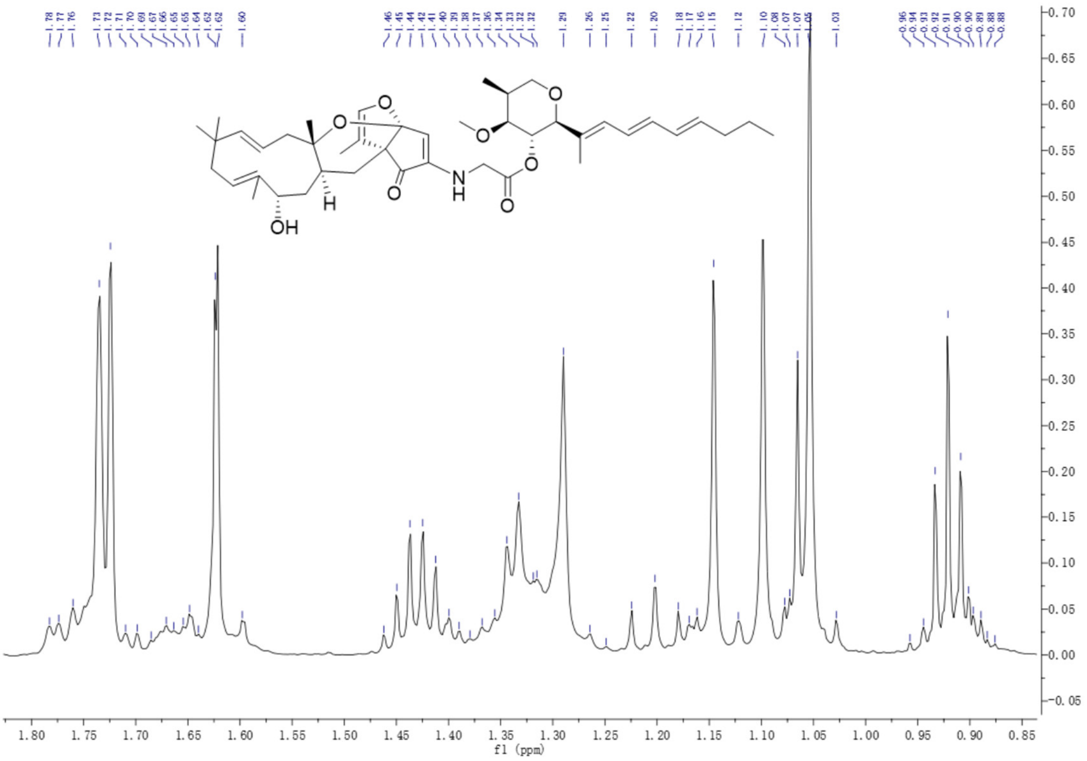

Figure S7. ^{13}C NMR spectrum of penipyridinone B (**1**, in MeOH-d_4)

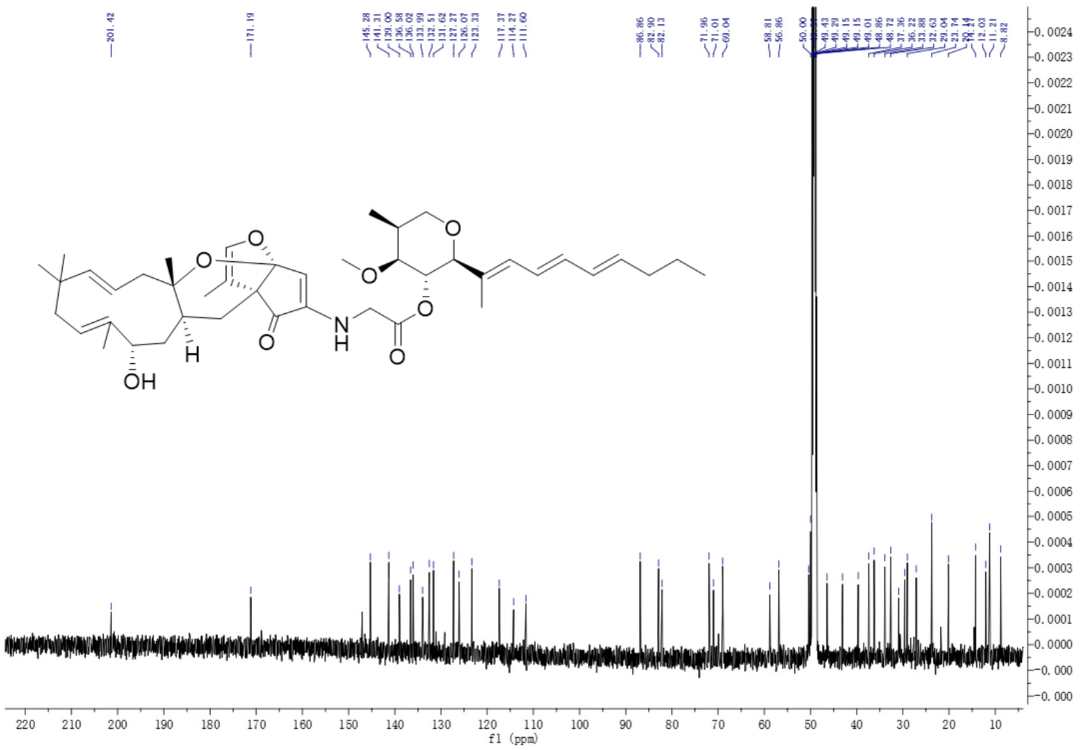

Figure S8. ^{13}C NMR spectrum of penipyridinone B (**1**, in MeOH-d_4)

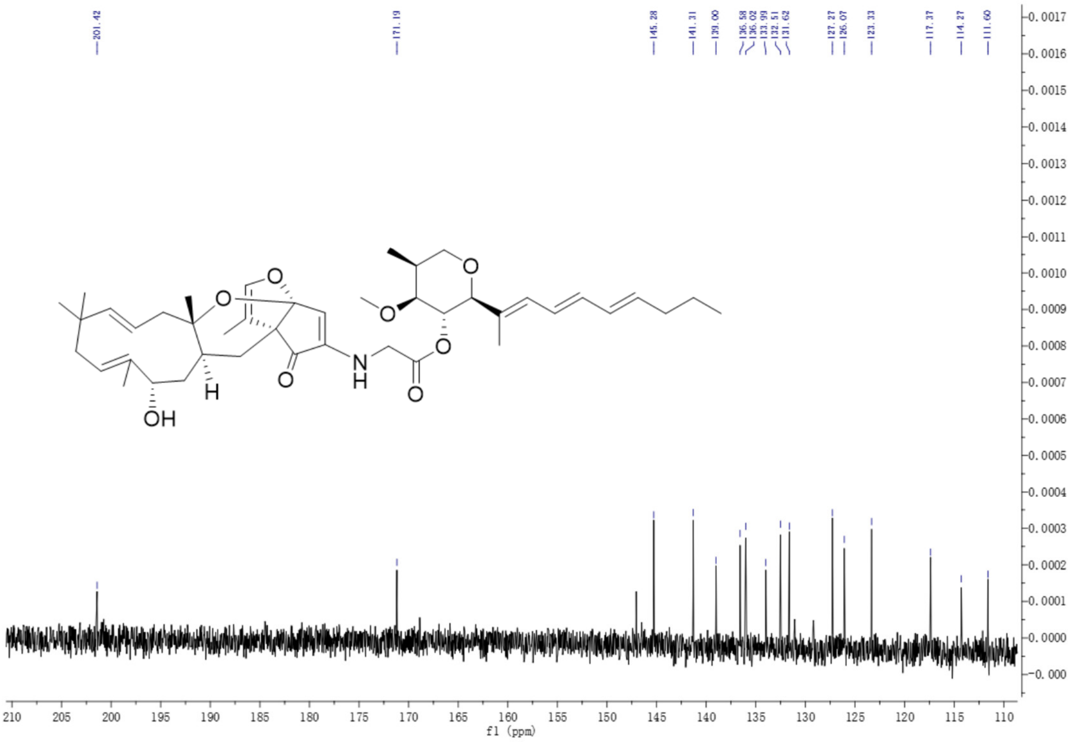

Figure S9. ^{13}C NMR spectrum of penipyridinone B (**1**, in MeOH-d_4)

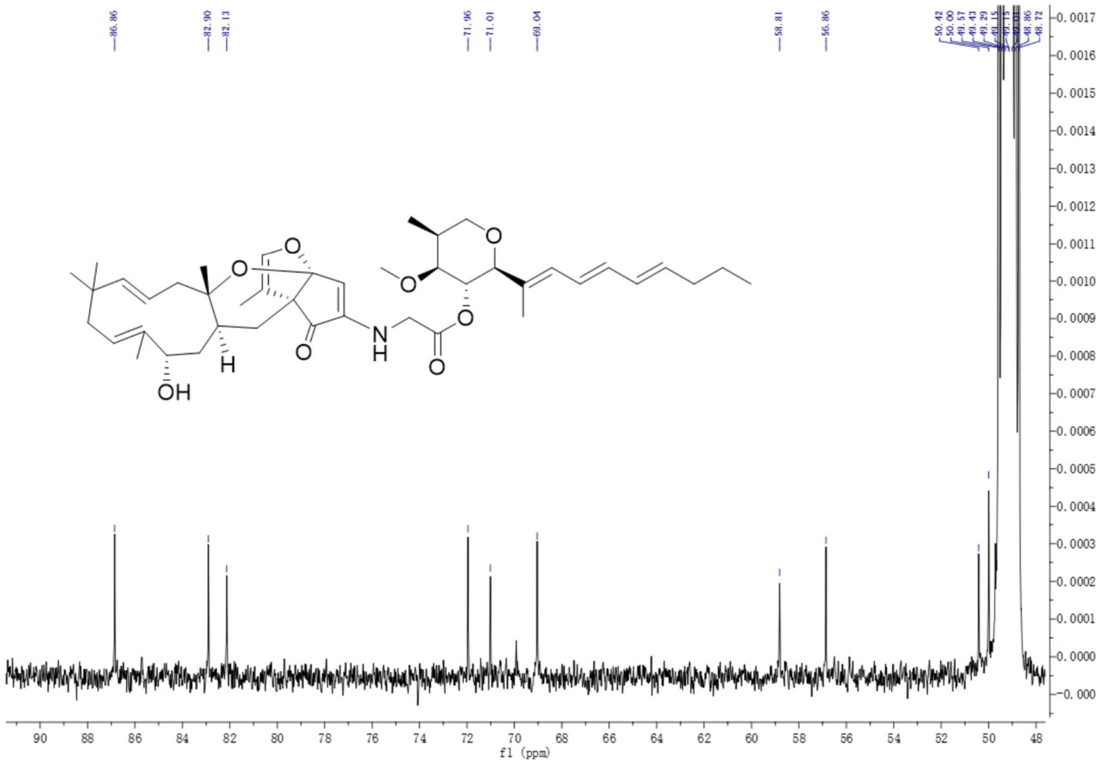

Figure S10. ^{13}C NMR spectrum of penipyridinone B (**1**, in MeOH-d_4)

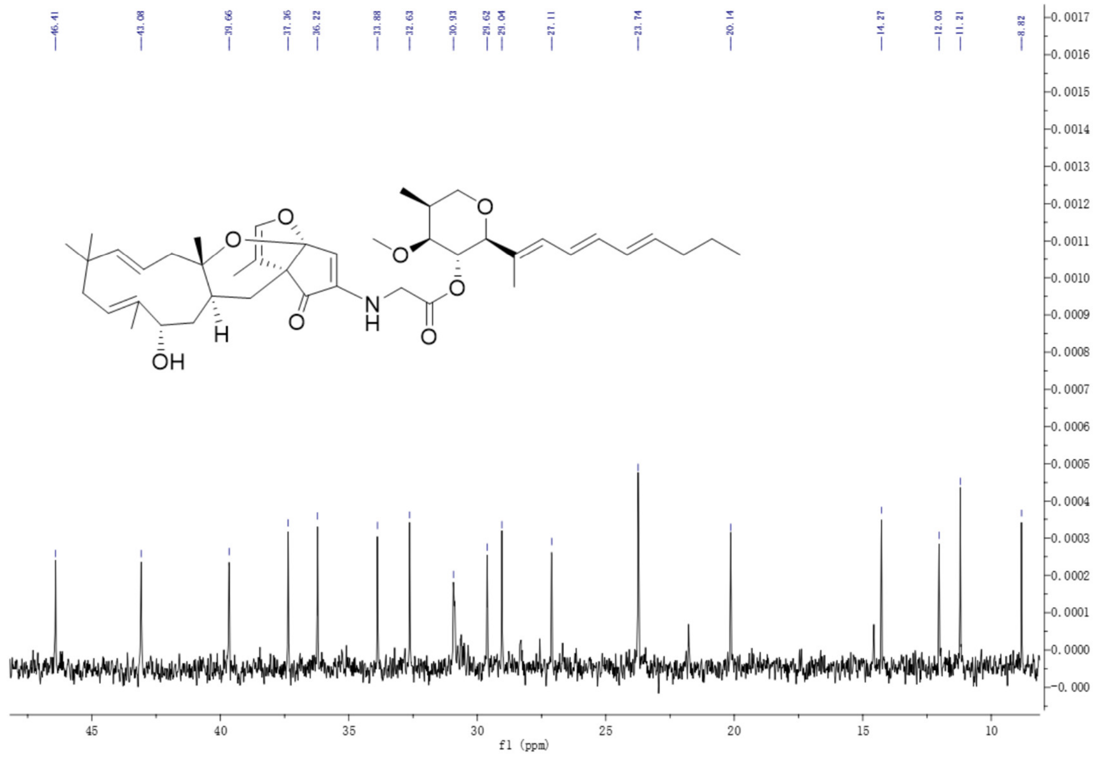

Figure S11. DEPT spectrum of penipyridinone B (**1**, in MeOH-d_4)

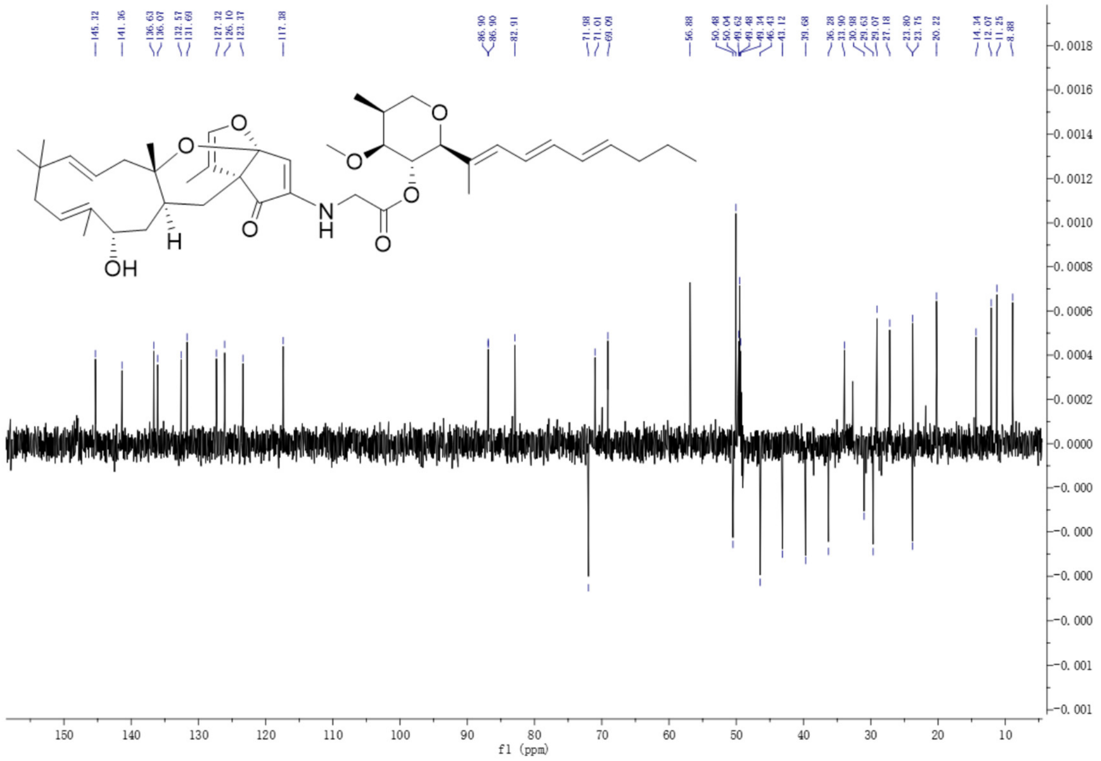

Figure S12. DEPT spectrum of penipyridinone B (**1**, in MeOH-d_4)

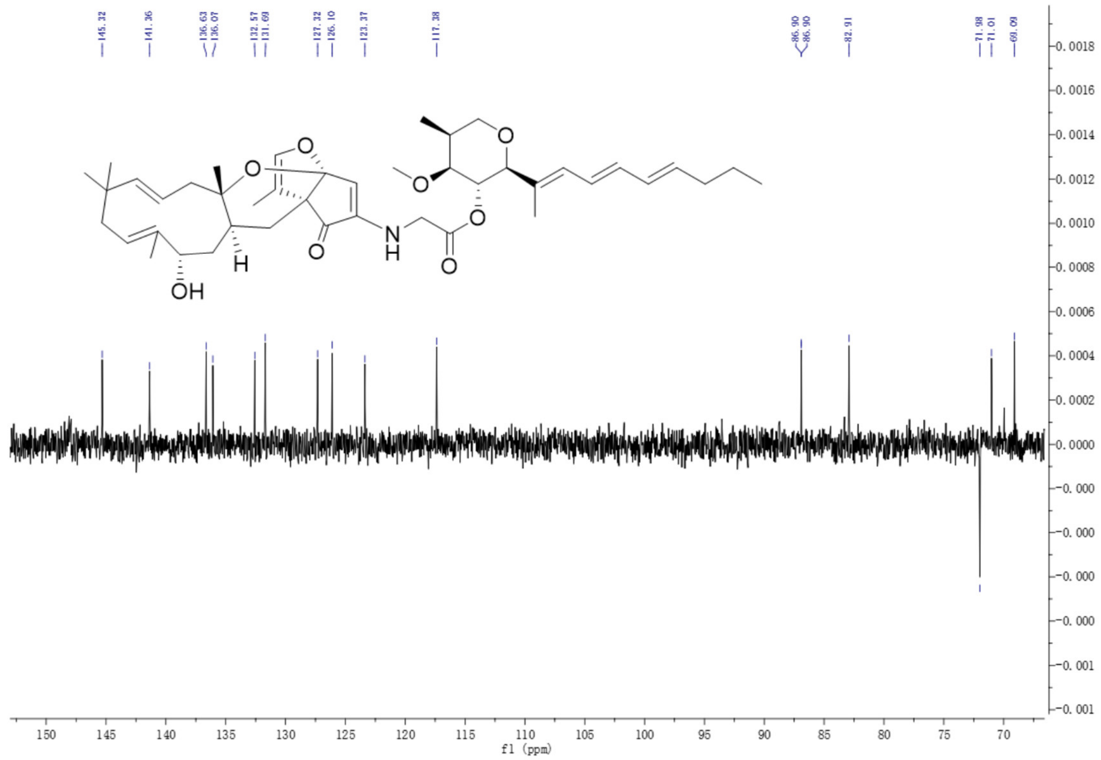

Figure S13. DEPT spectrum of penipyridinone B (**1**, in MeOH-d_4)

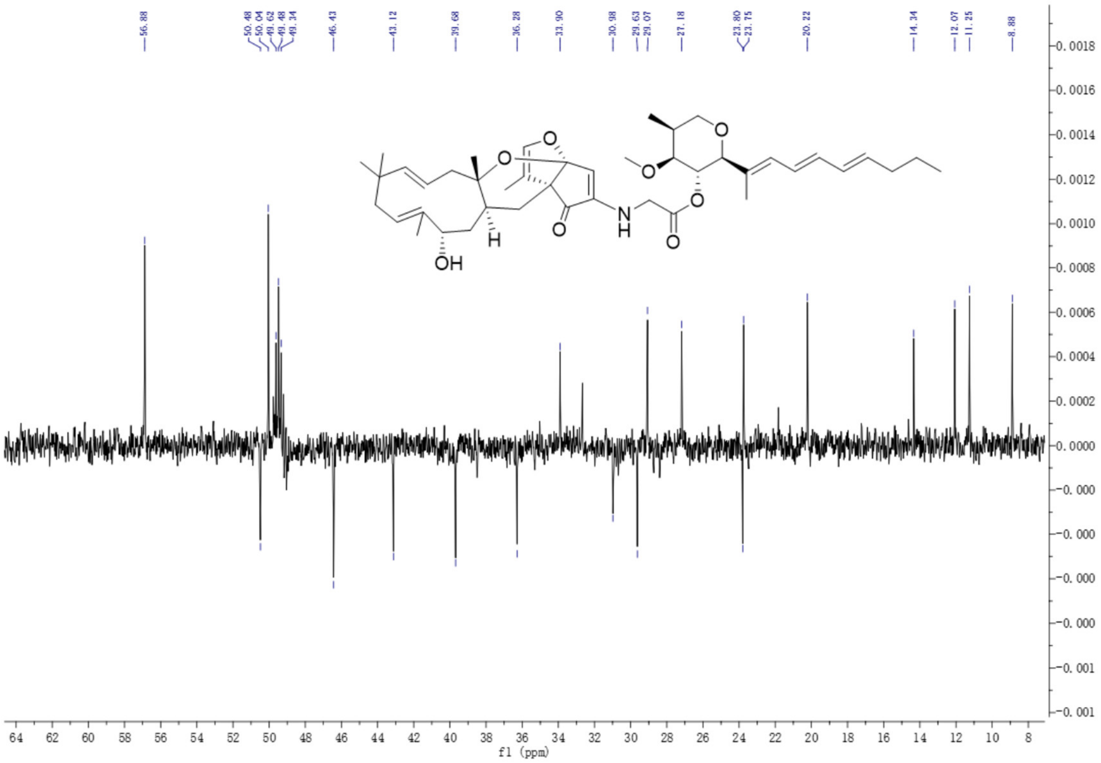

Figure S14. HMQC spectrum of penipyridinone B (**1**, in MeOH-d_4)

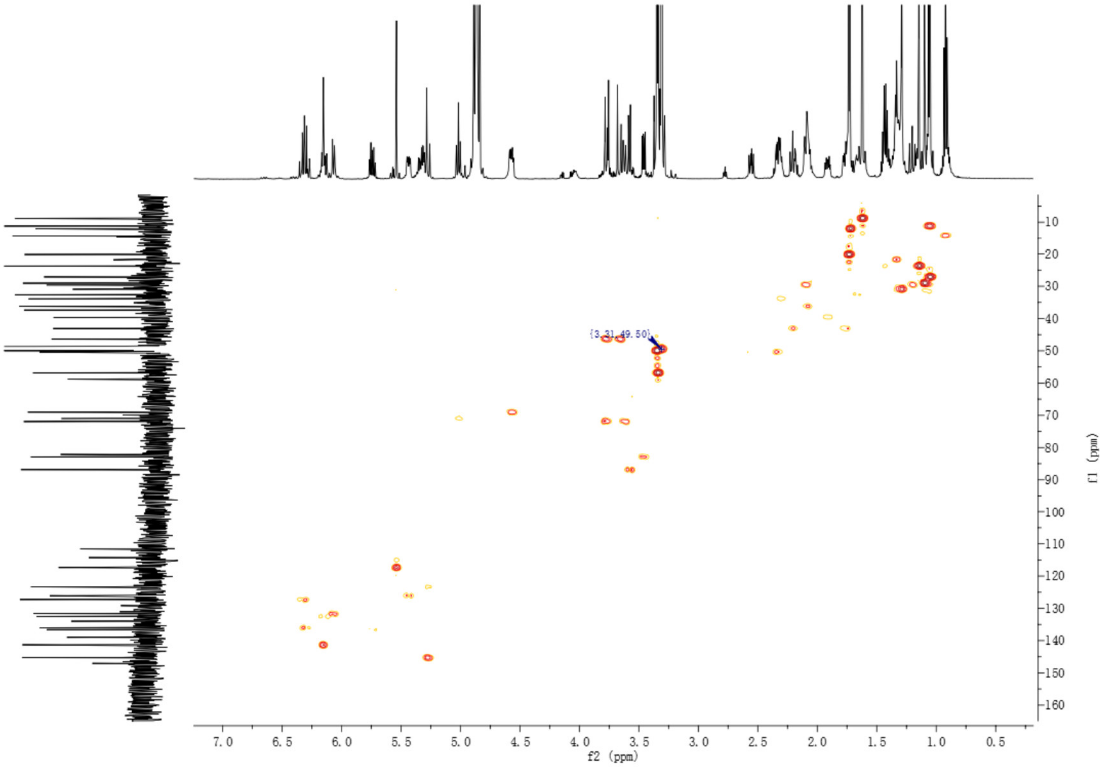

Figure S15. HMQC spectrum of penipyridinone B (**1**, in MeOH-d_4)

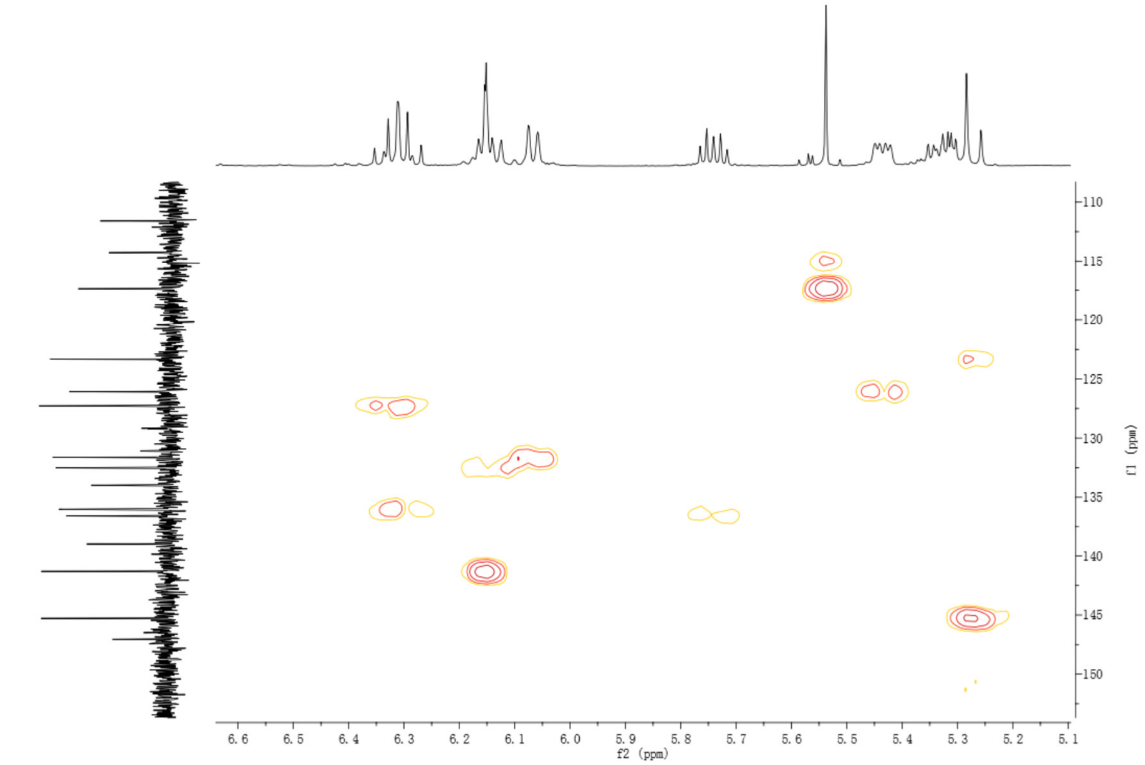

Figure S16. HMQC spectrum of penipyridinone B (**1**, in MeOH-d_4)

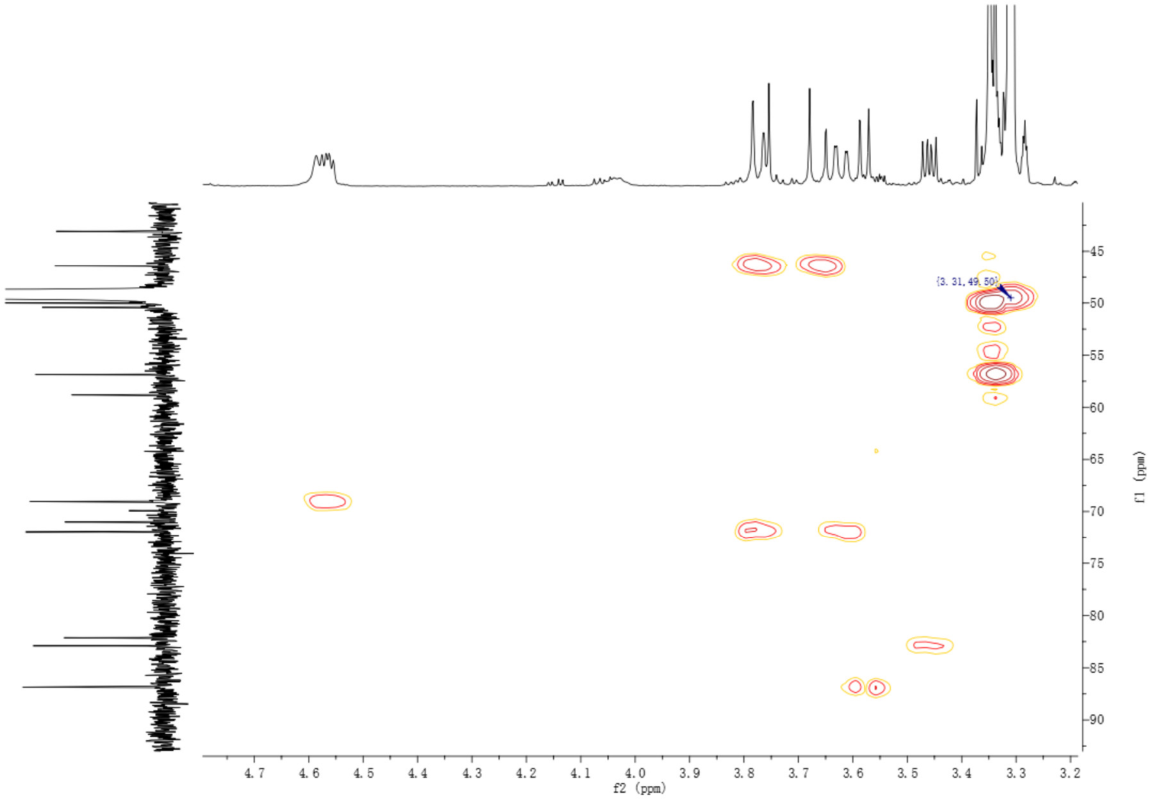

Figure S17. HMQC spectrum of penipyridinone B (**1**, in MeOH-d_4)

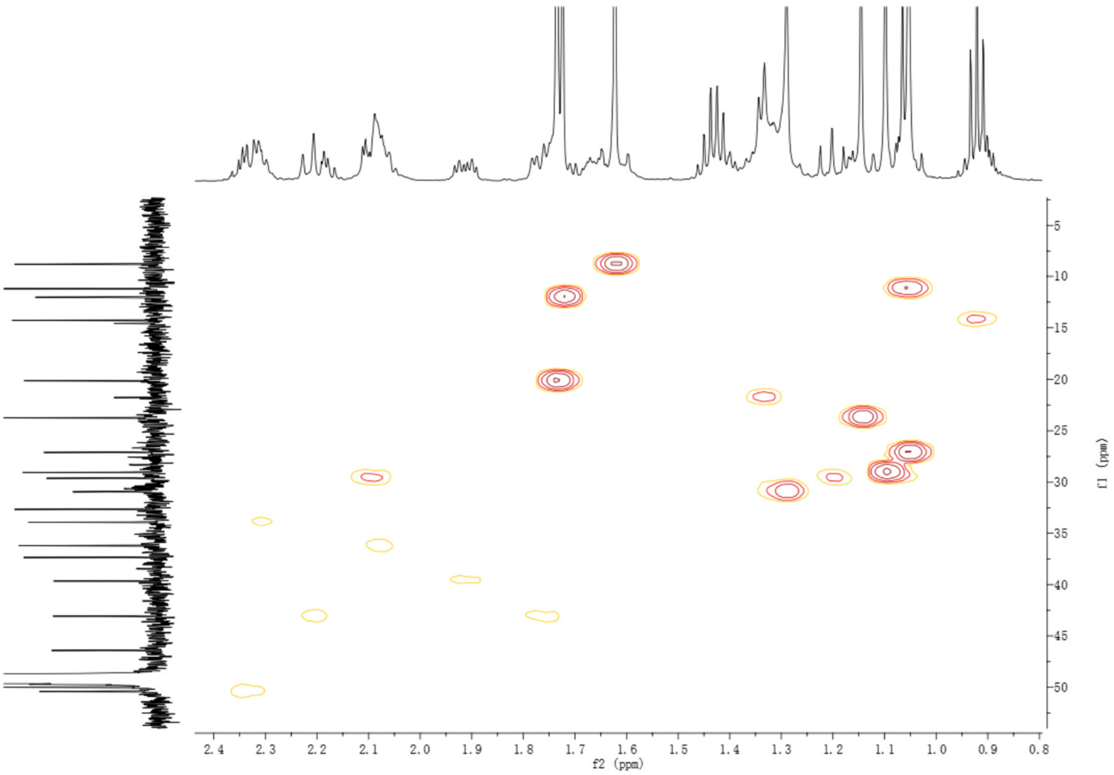

Figure S18. COSY spectrum of penipyridinone B (**1**, in MeOH-d_4)

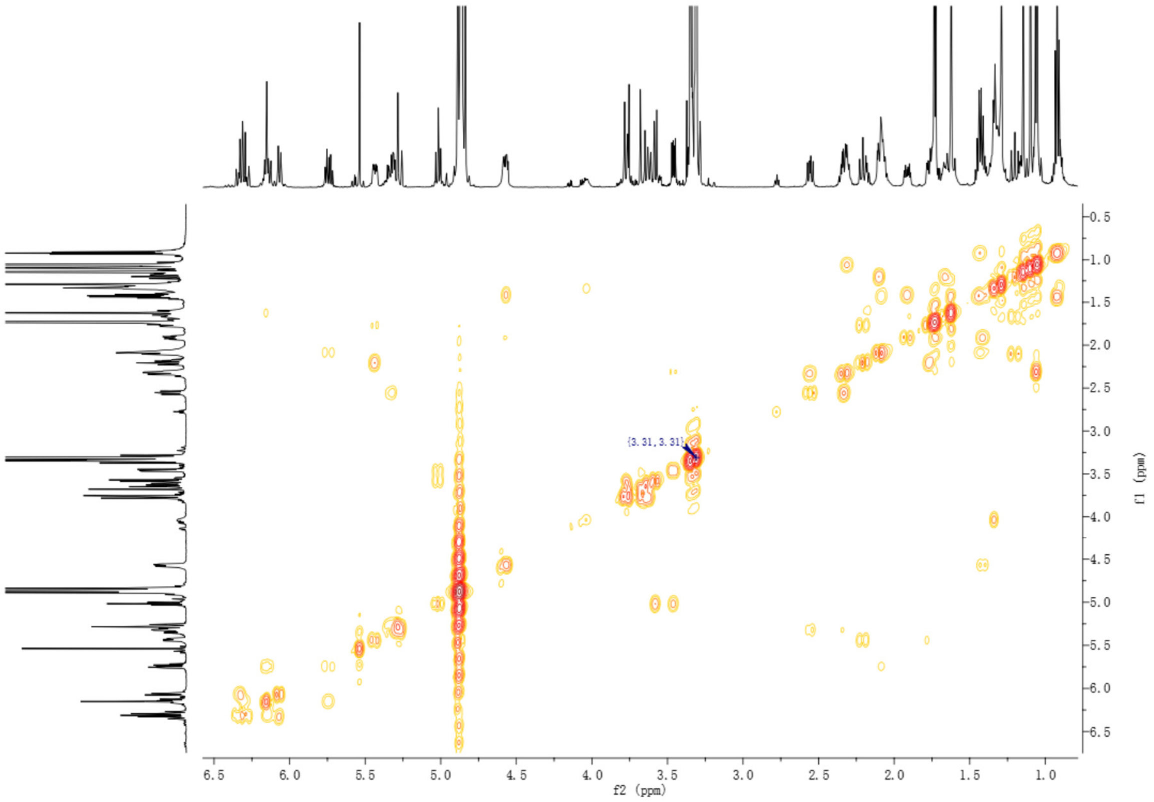

Figure S19. COSY spectrum of penipyridinone B (**1**, in MeOH-d_4)

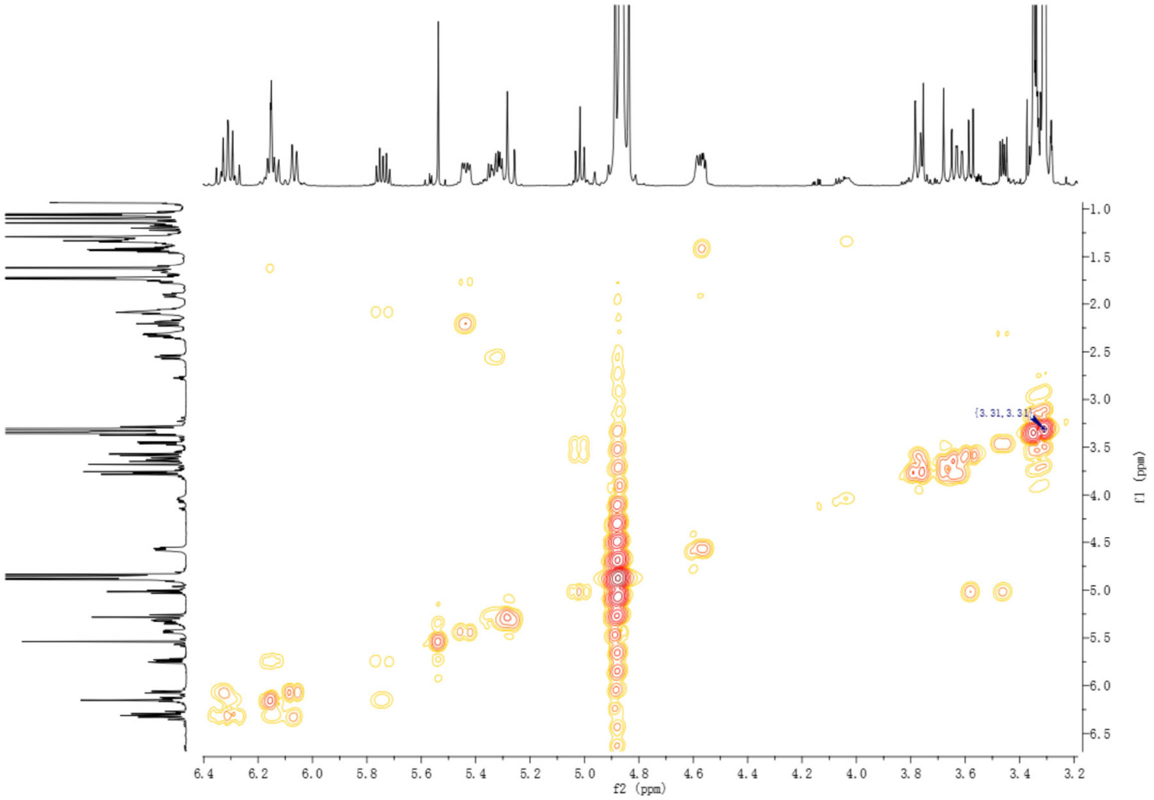

Figure S20. COSY spectrum of penipyridinone B (**1**, in MeOH-d_4)

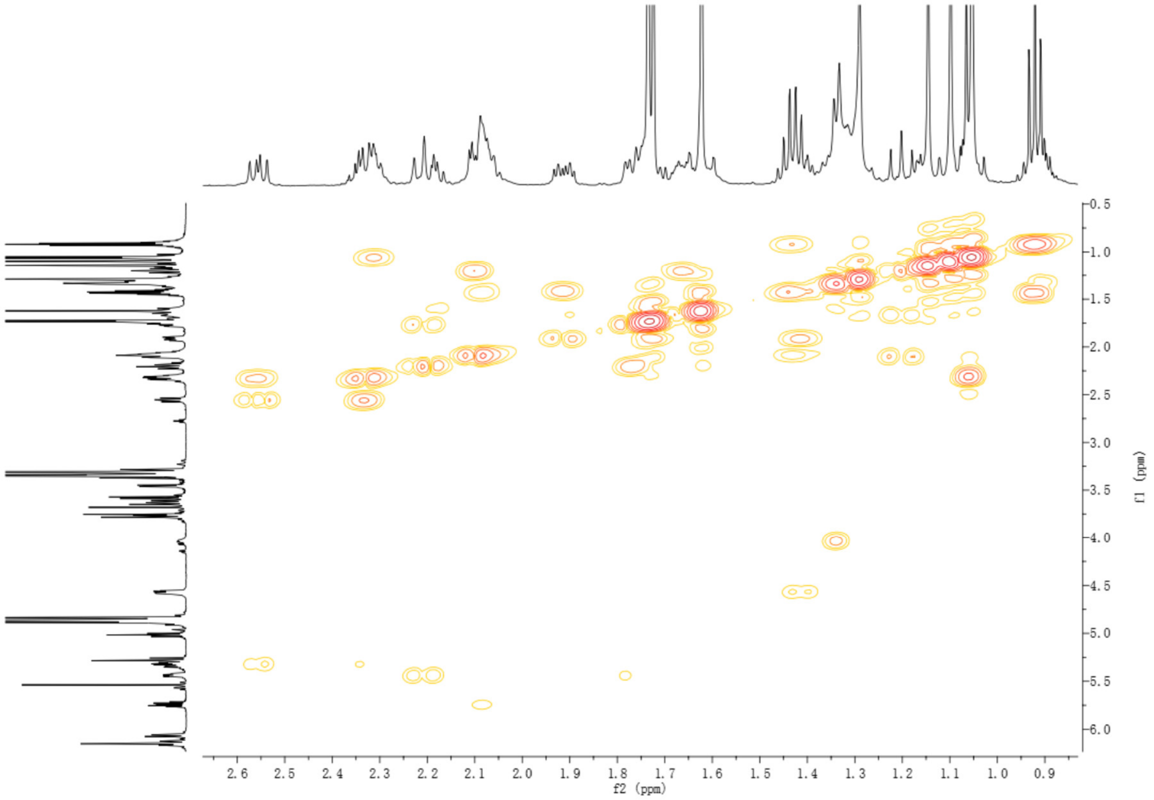

Figure S21. HMBC spectrum of penipyridinone B (**1**, in MeOH-d_4)

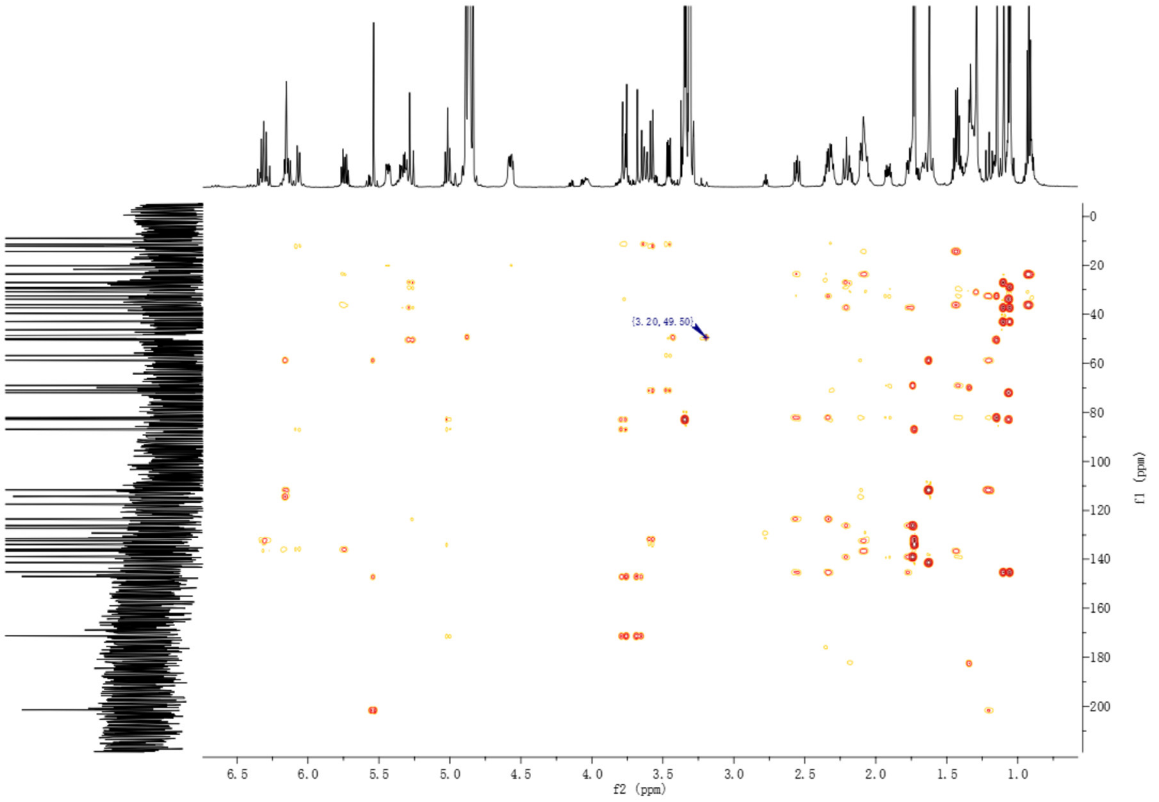

Figure S22. HMBC spectrum of penipyridinone B (**1**, in MeOH-d_4)

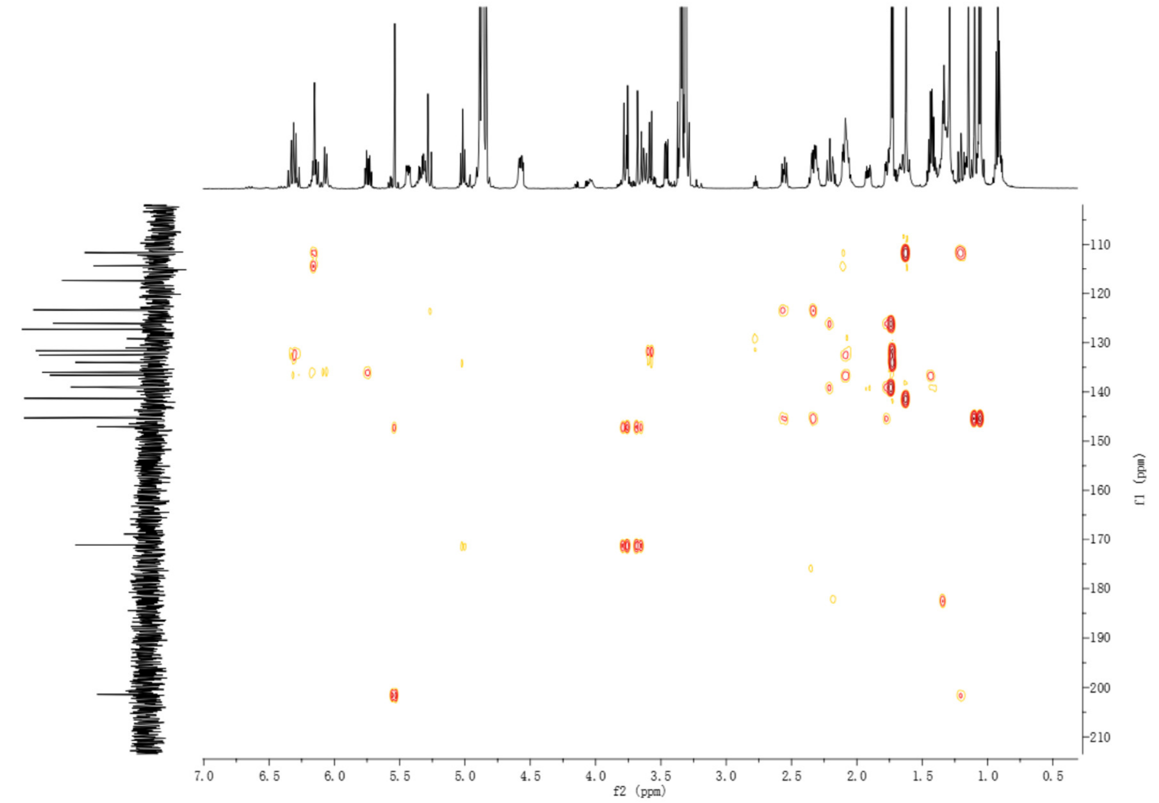

Figure S23. HMBC spectrum of penipyridinone B (**1**, in MeOH-d_4)

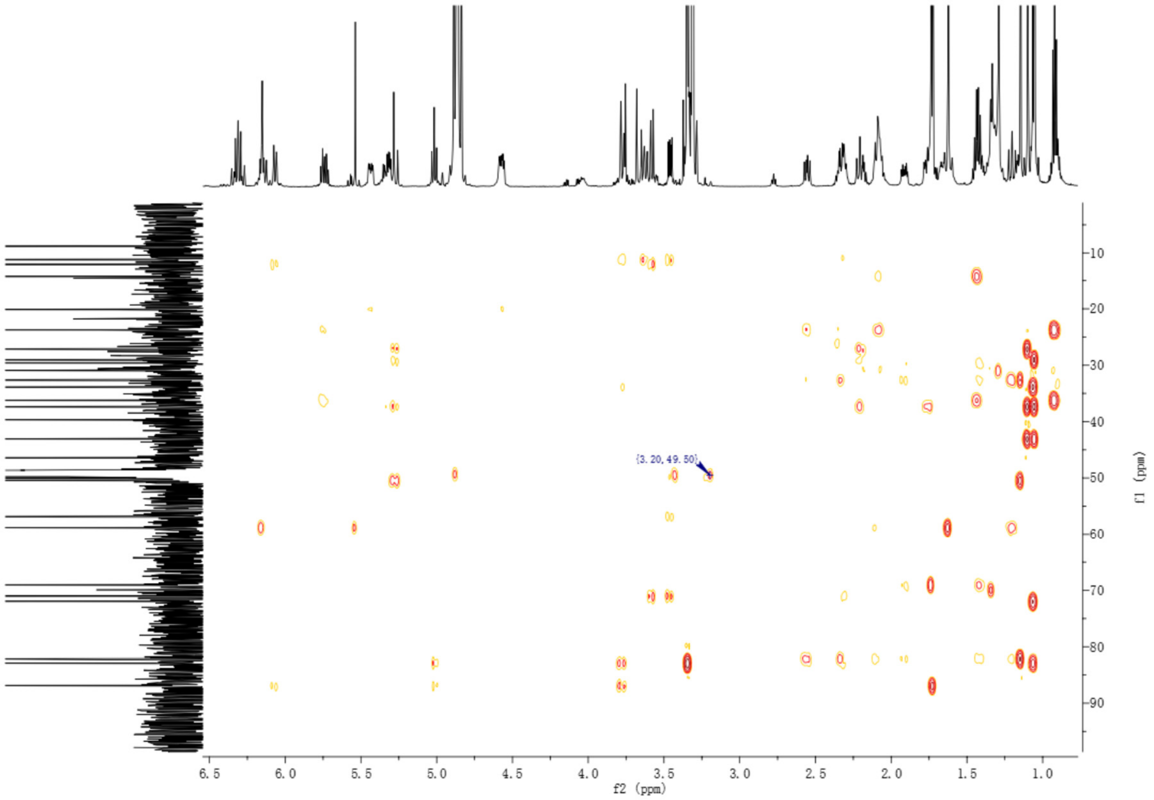

Figure S24. NOE spectrum of penipyridinone B (**1**, in MeOH-d_4)

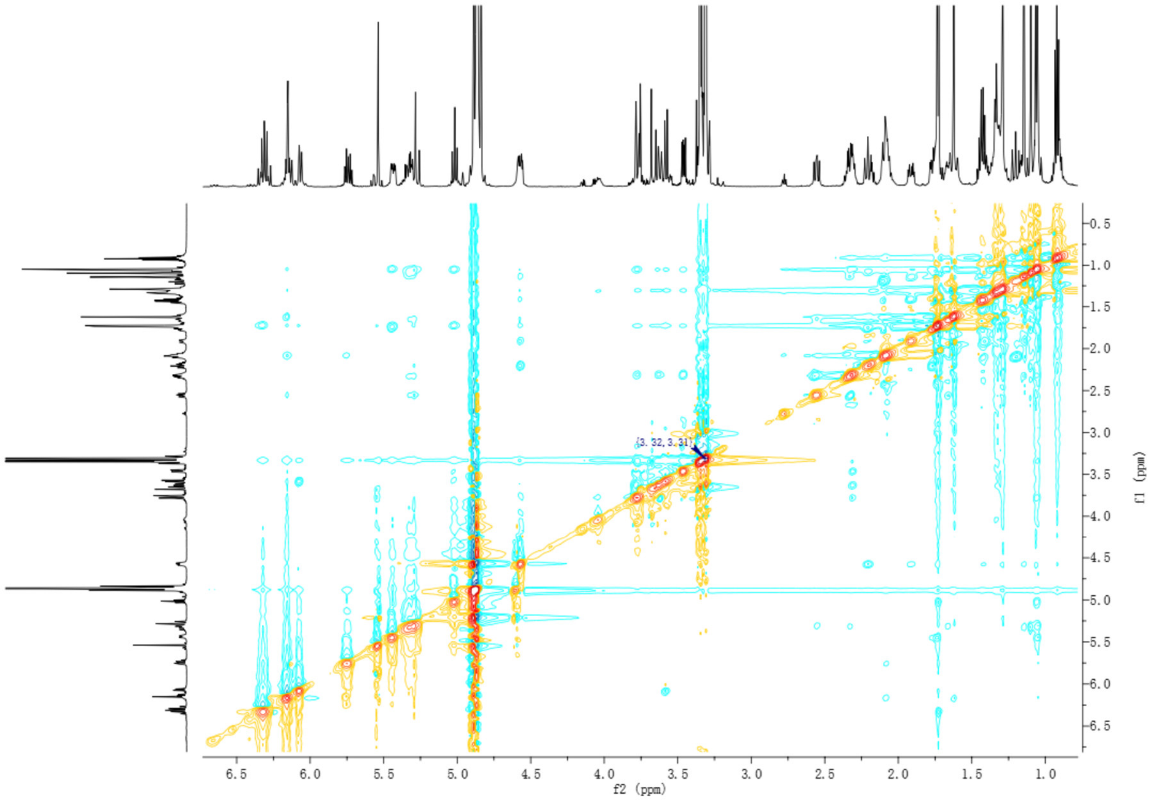

Figure S25. ^{1}H NMR spectrum of penipyridinone B (**1**, in DMSO-d_6)

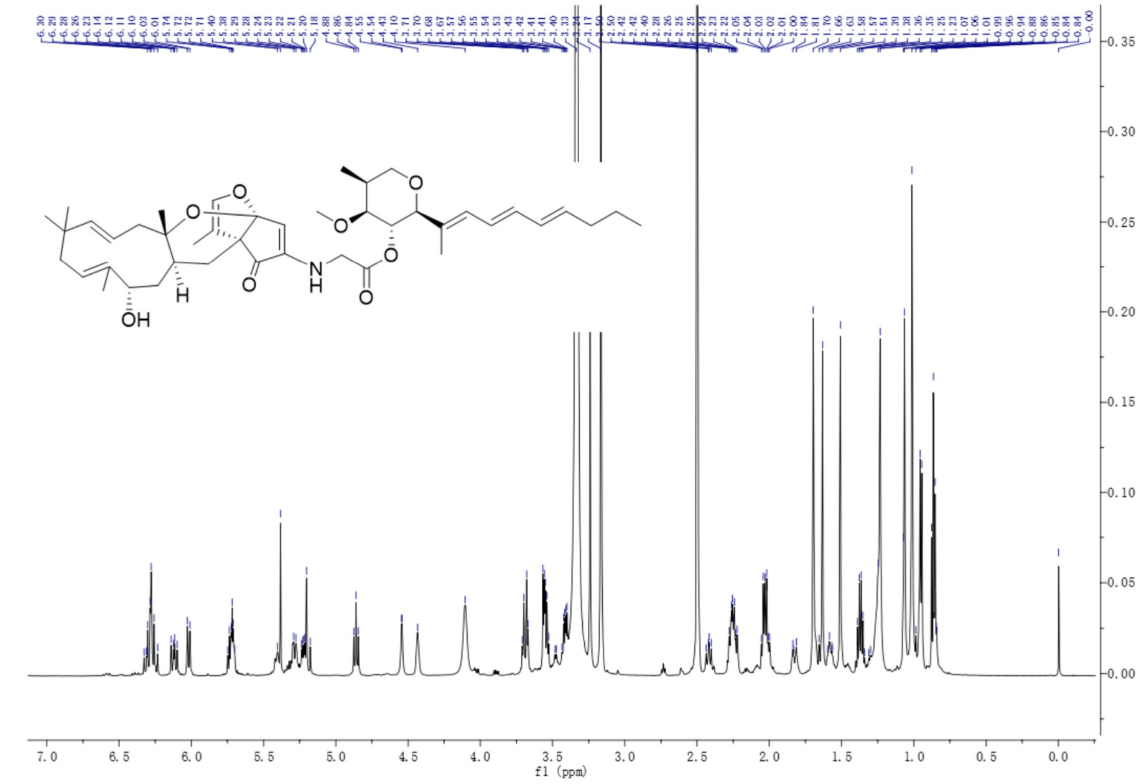

Figure S26. ^{1}H NMR spectrum of penipyridinone B (**1**, in DMSO-d_6)

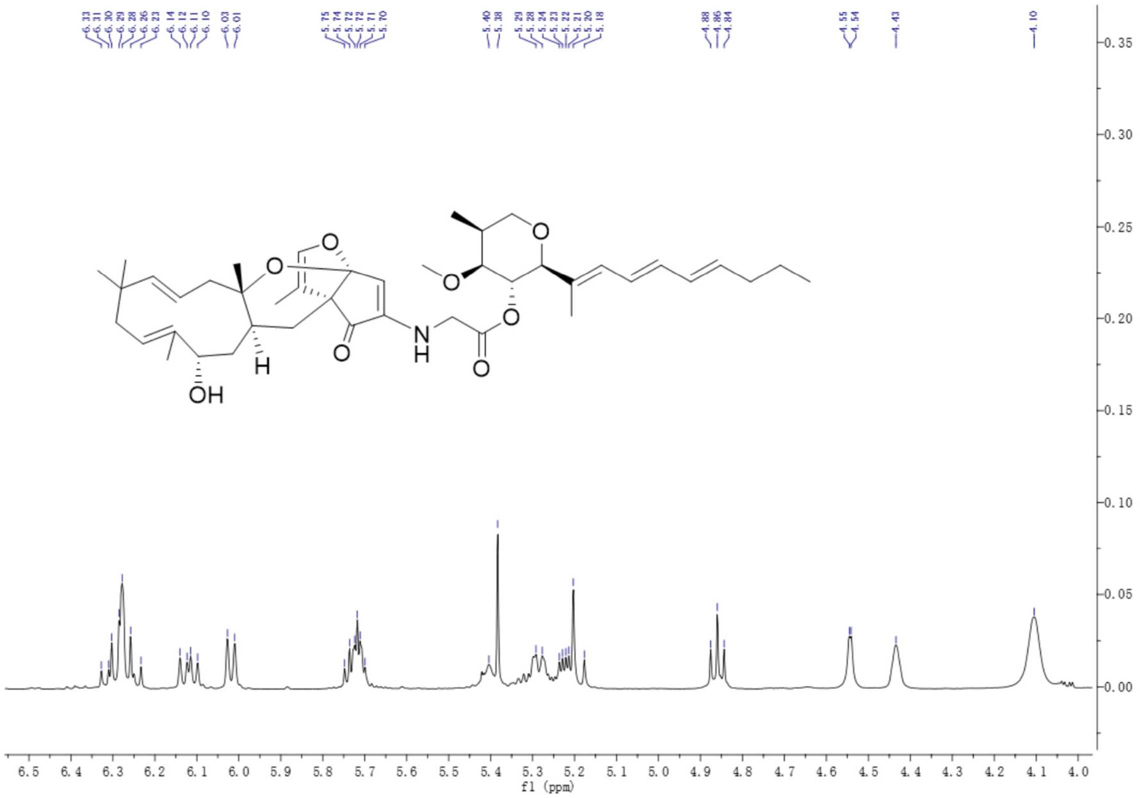

Figure S27. ^{1}H NMR spectrum of penipyridinone B (**1**, in DMSO-d_6)

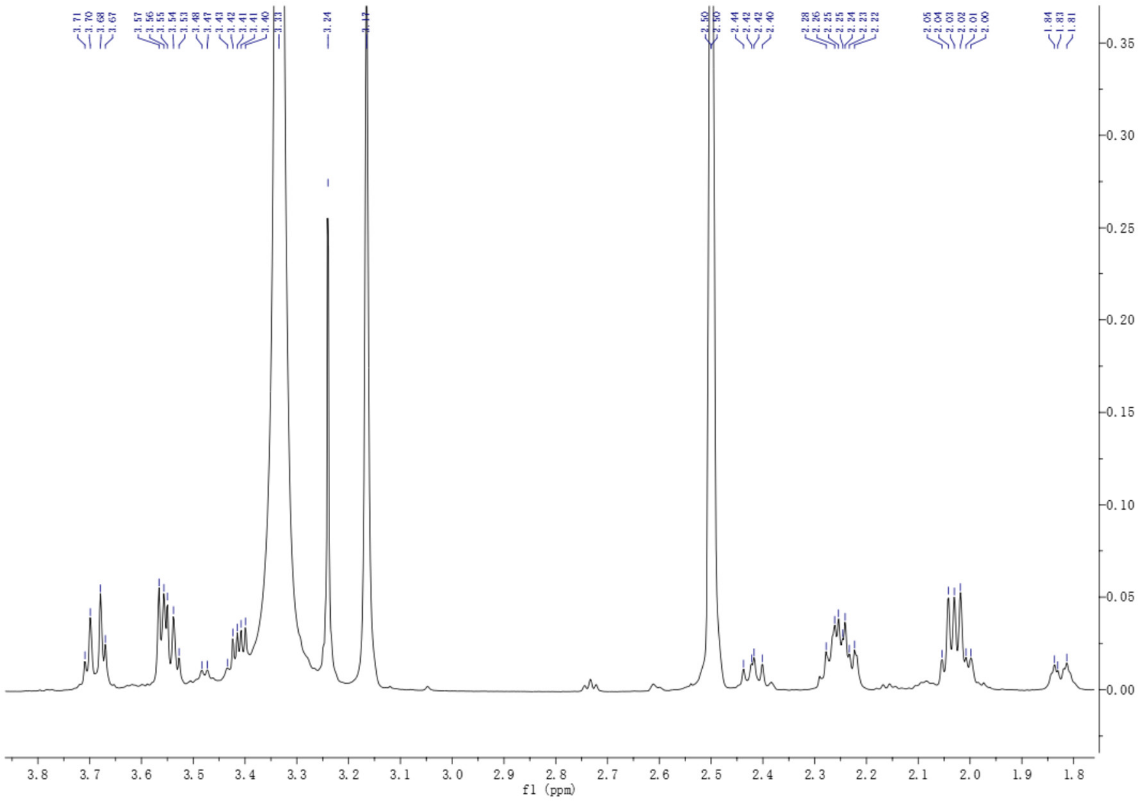

Figure S28. ^{1}H NMR spectrum of penipyridinone B (**1**, in DMSO-d_6)

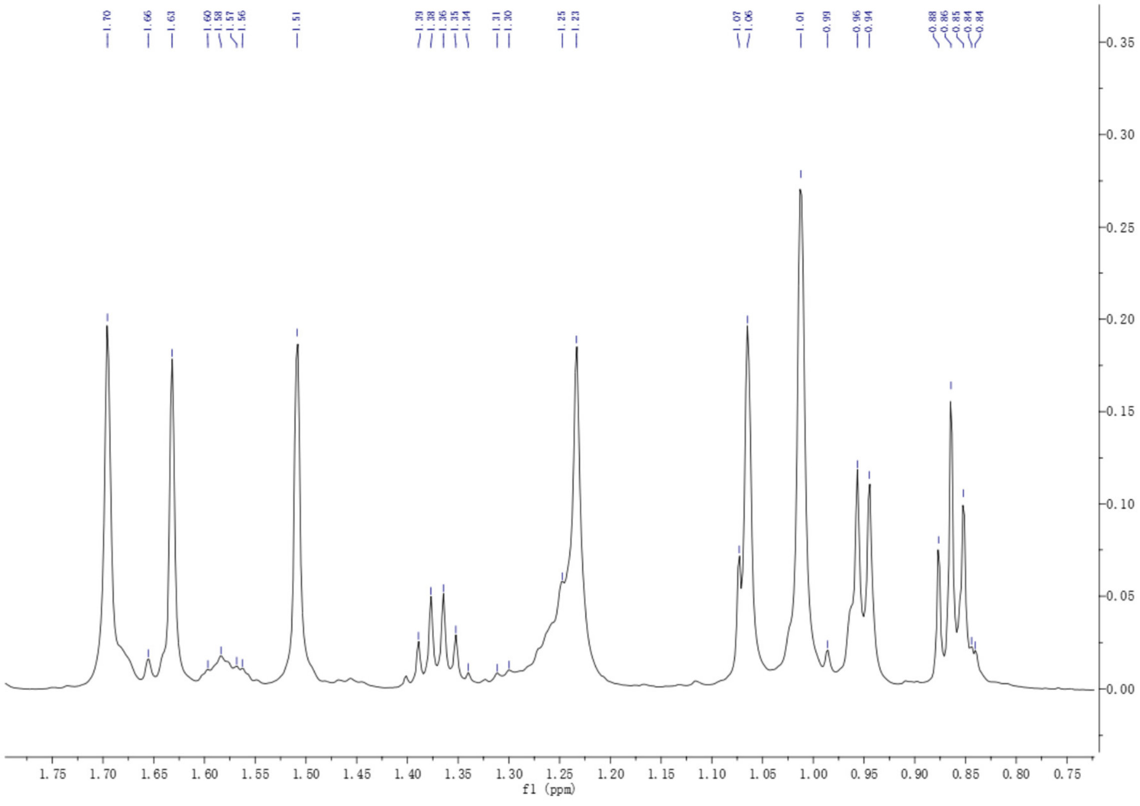

Figure S29. ^{13}C NMR spectrum of penipyridinone B (**1**, in DMSO-d_6)

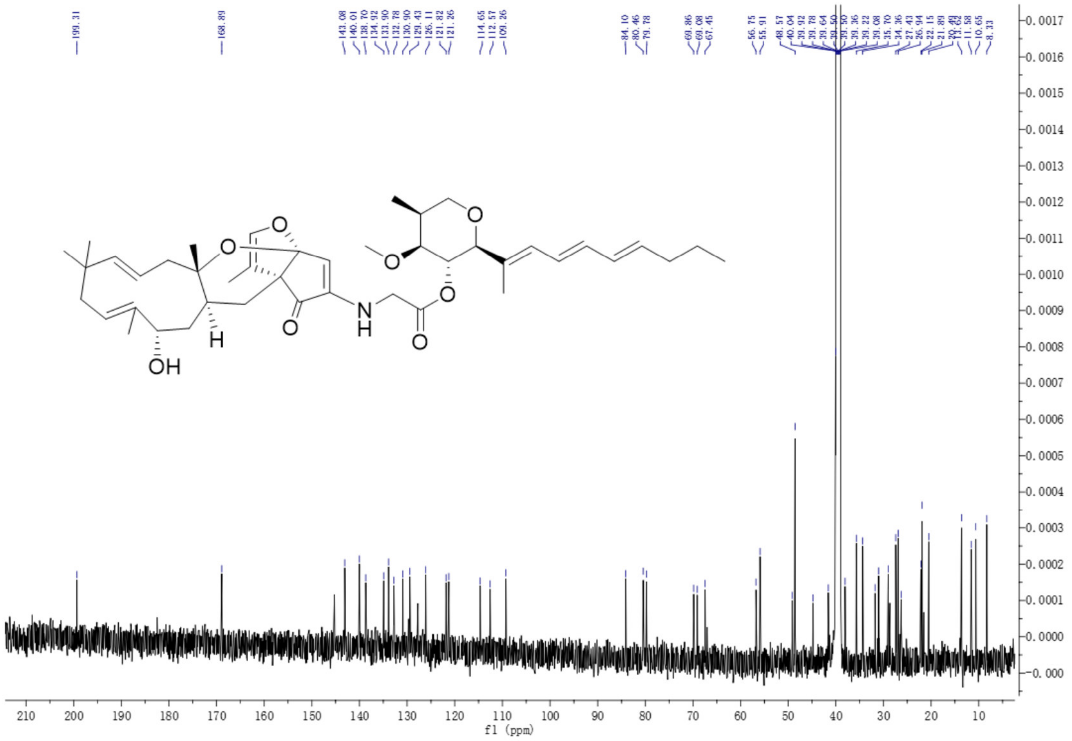

Figure S30. ^{13}C NMR spectrum of penipyridinone B (**1**, in DMSO-d_6)

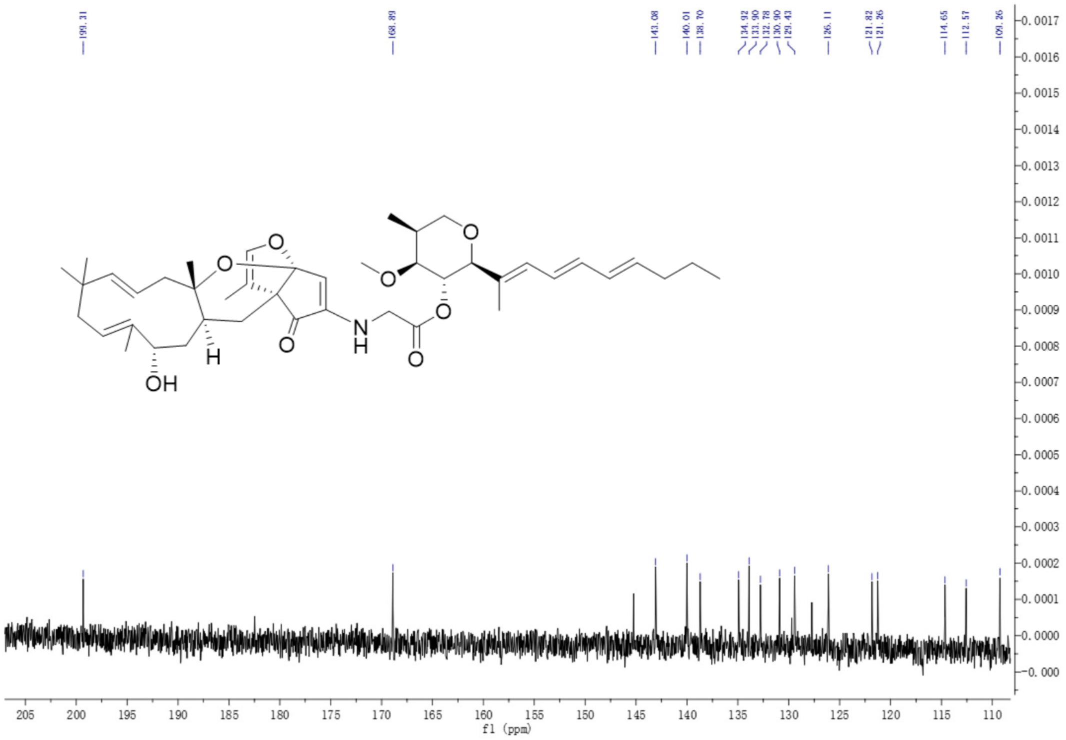

Figure S31. ^{13}C NMR spectrum of penipyridinone B (**1**, in DMSO-d_6)

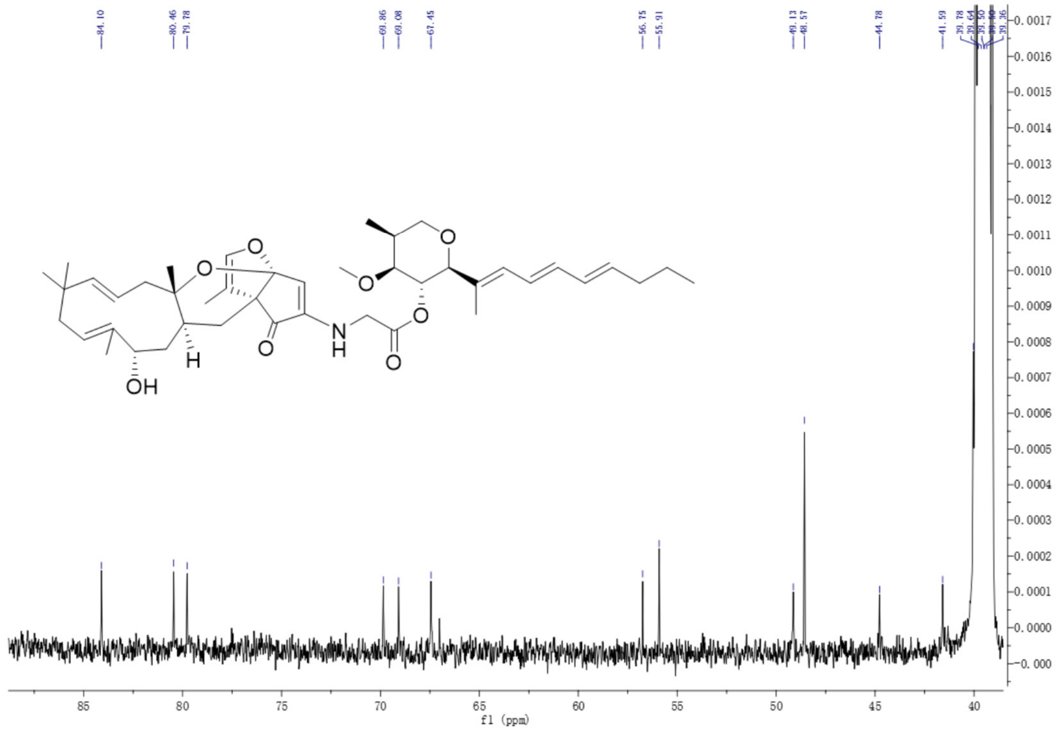

Figure S32. ^{13}C NMR spectrum of penipyridinone B (**1**, in DMSO-d_6)

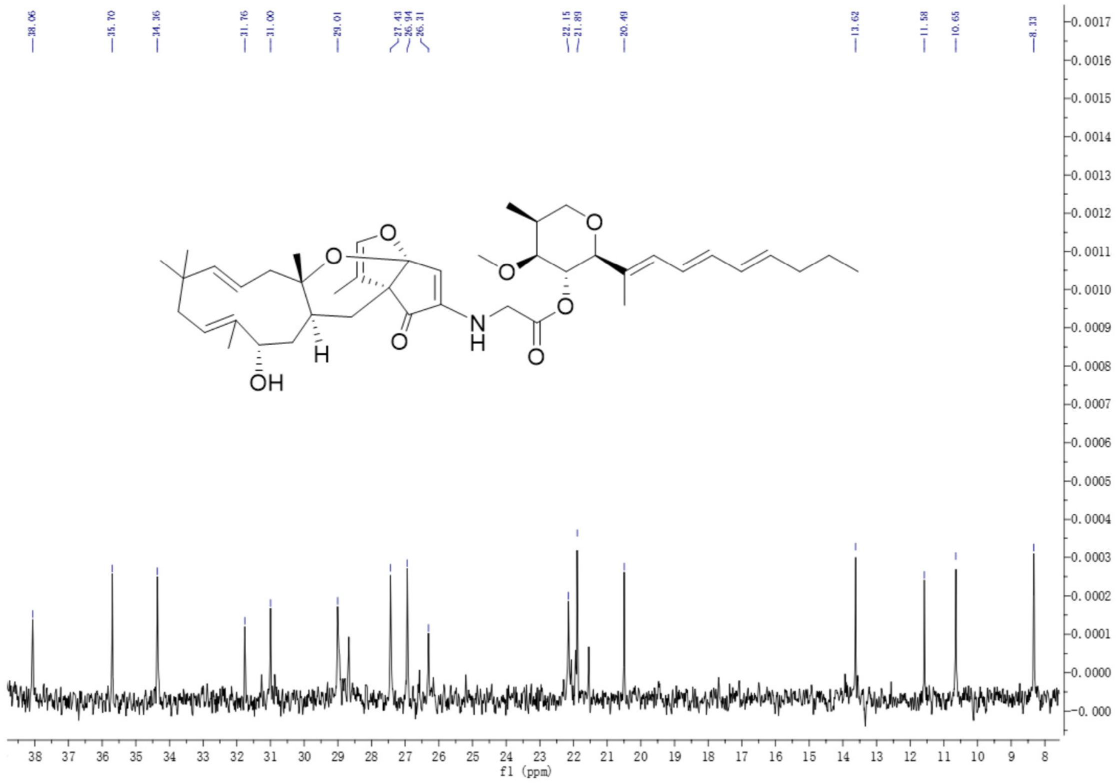

Figure S33. HMQC spectrum of penipyridinone B (**1**, in DMSO-d_6)

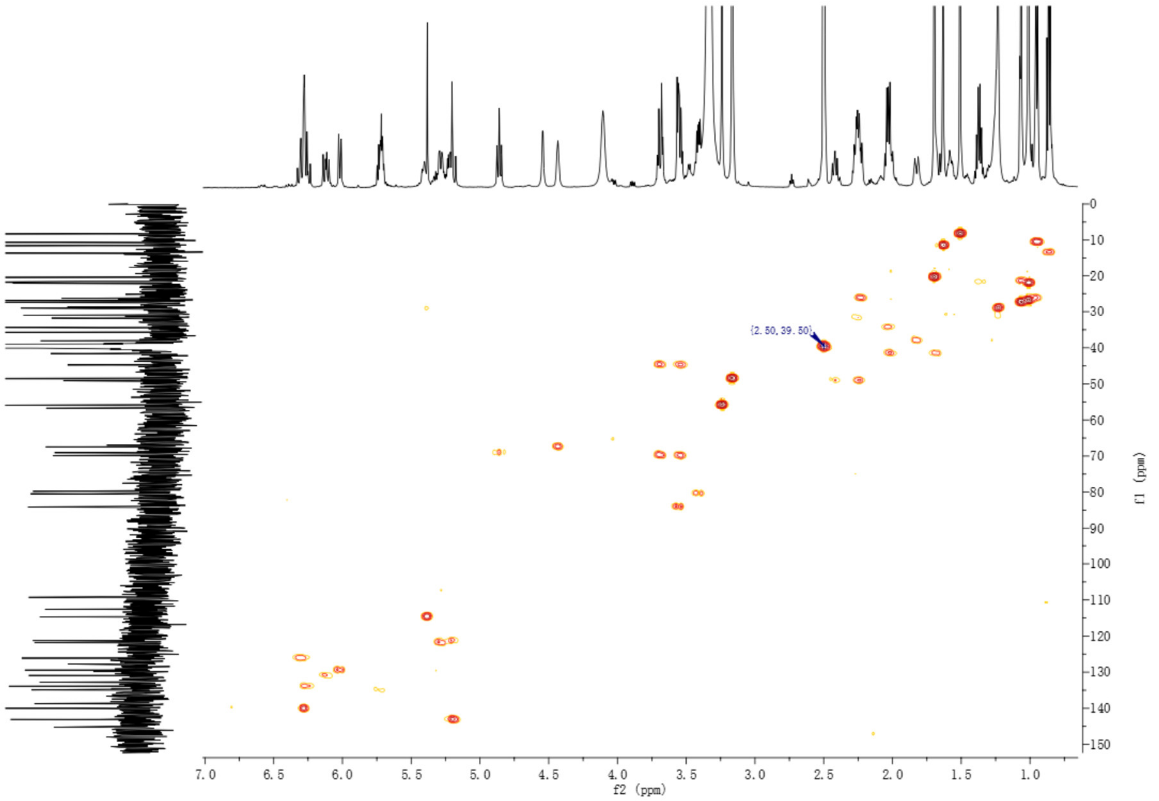

Figure S34. HMQC spectrum of penipyridinone B (**1**, in DMSO-d_6)

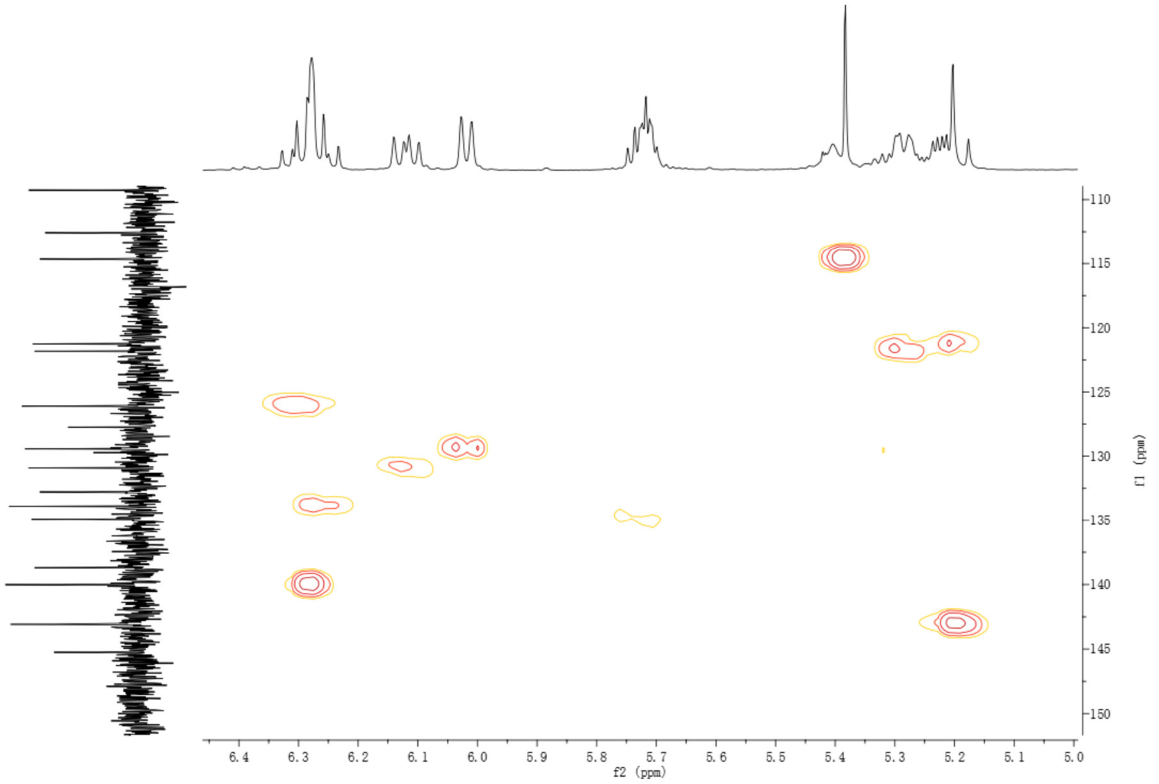

Figure S35. HMQC spectrum of penipyridinone B (**1**, in DMSO-d_6)

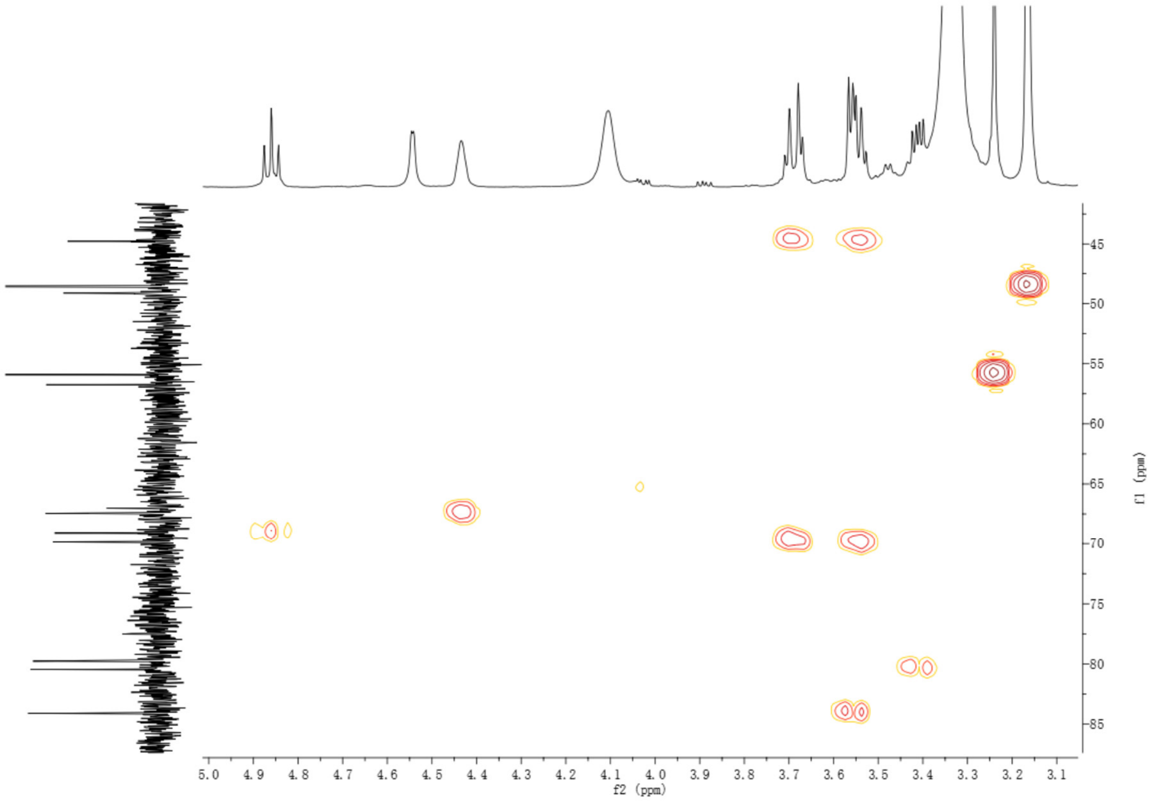

Figure S36. HMQC spectrum of penipyridinone B (**1**, in DMSO-d_6)

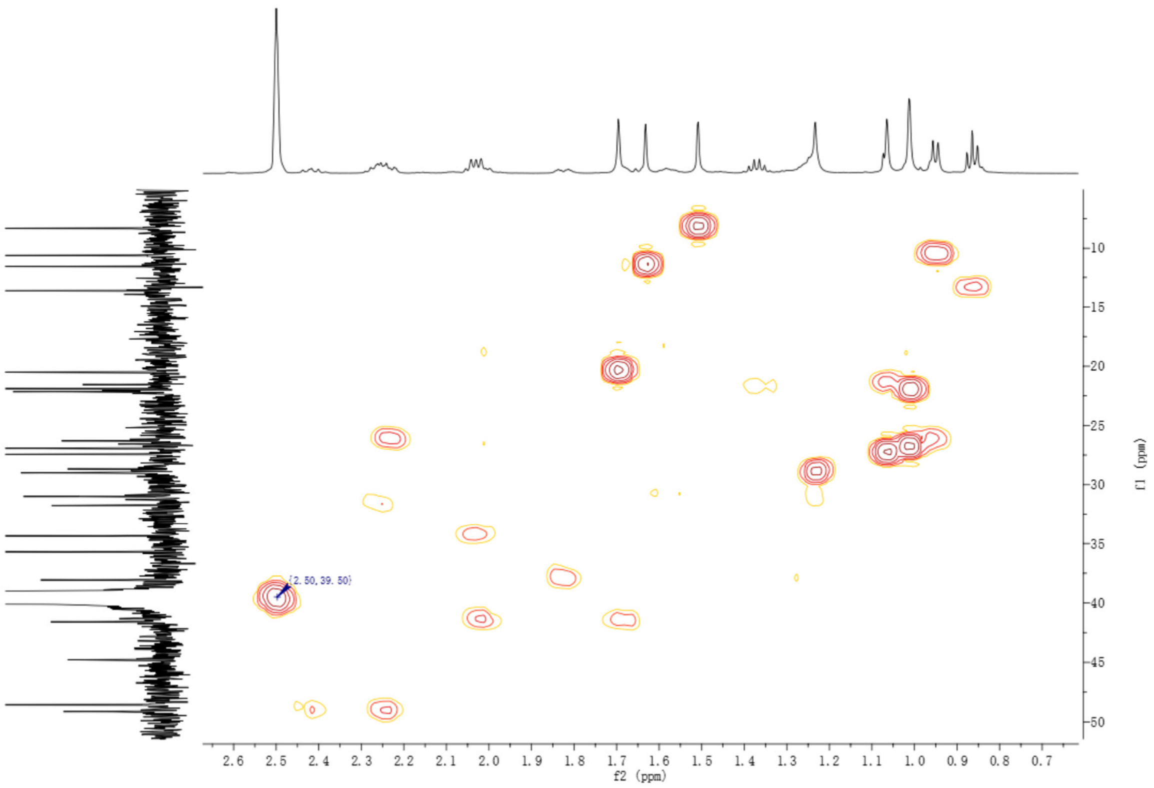

Figure S37. HMBC spectrum of penipyridinone B (**1**, in DMSO-d_6)

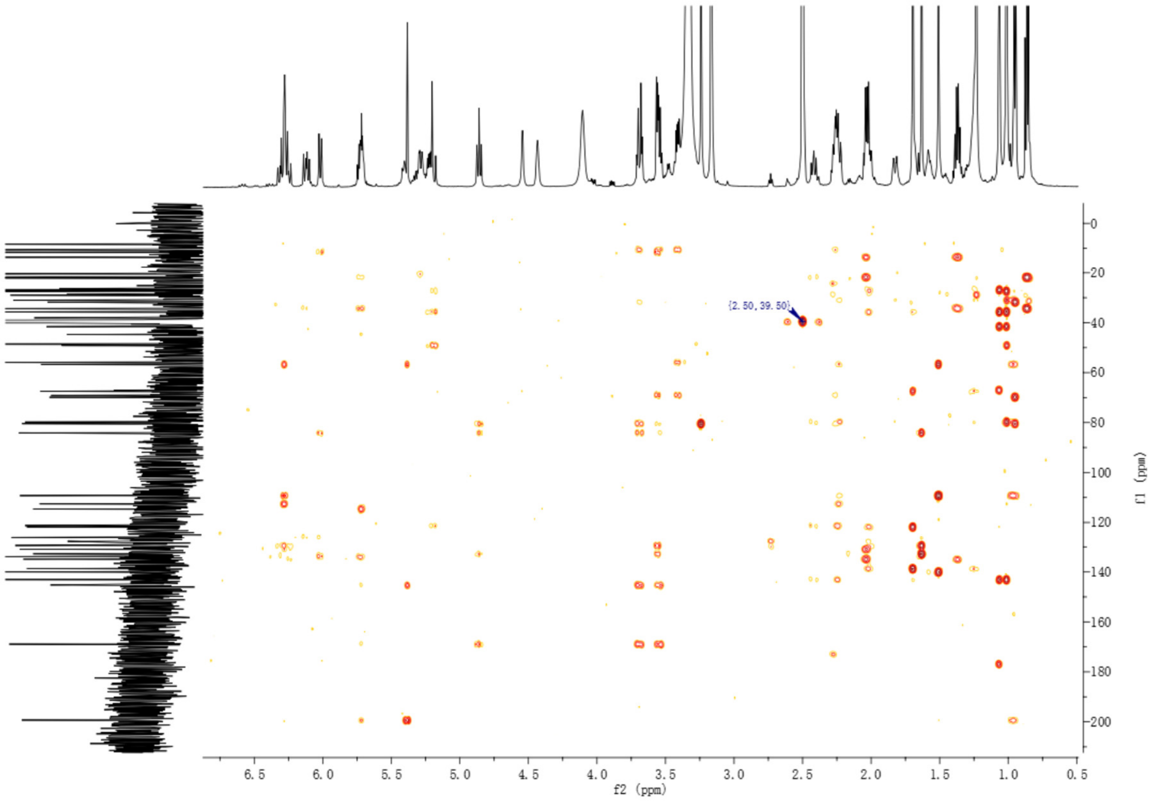

Figure S38. HMBC spectrum of penipyridinone B (**1**, in DMSO-d_6)

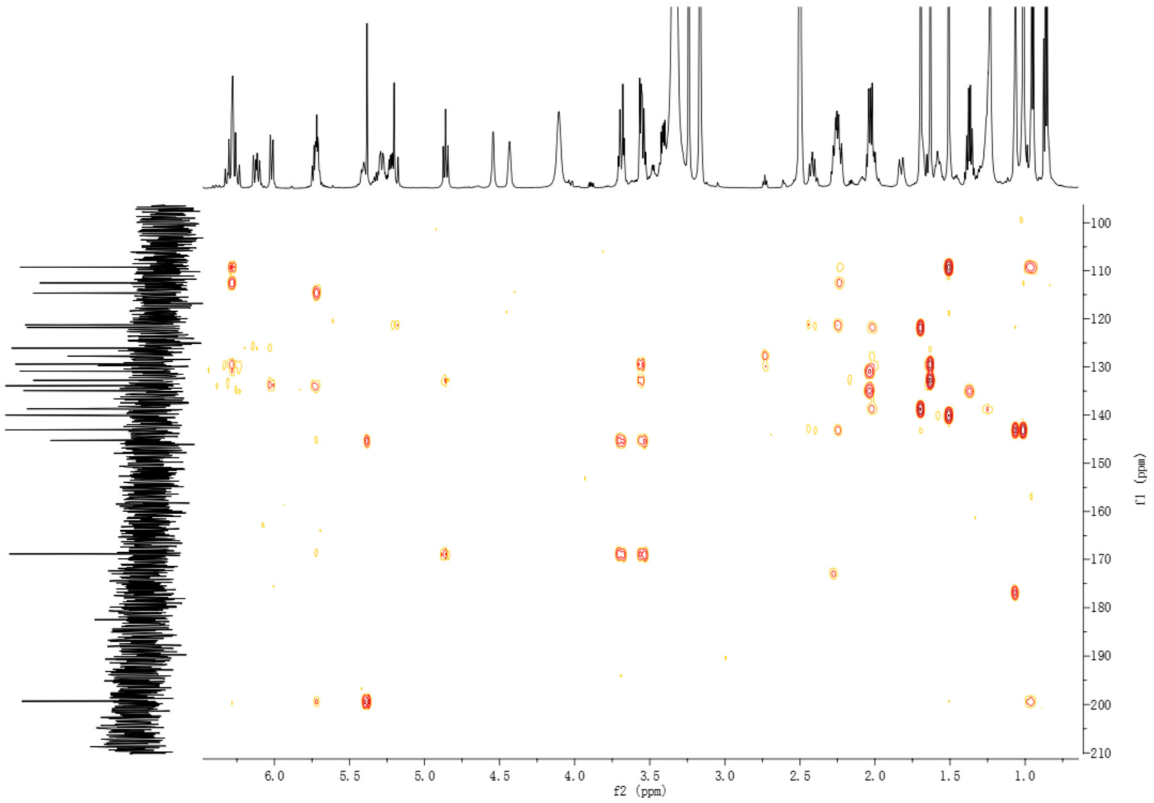

Figure S39. HMBC spectrum of penipyridinone B (**1**, in DMSO-d_6)

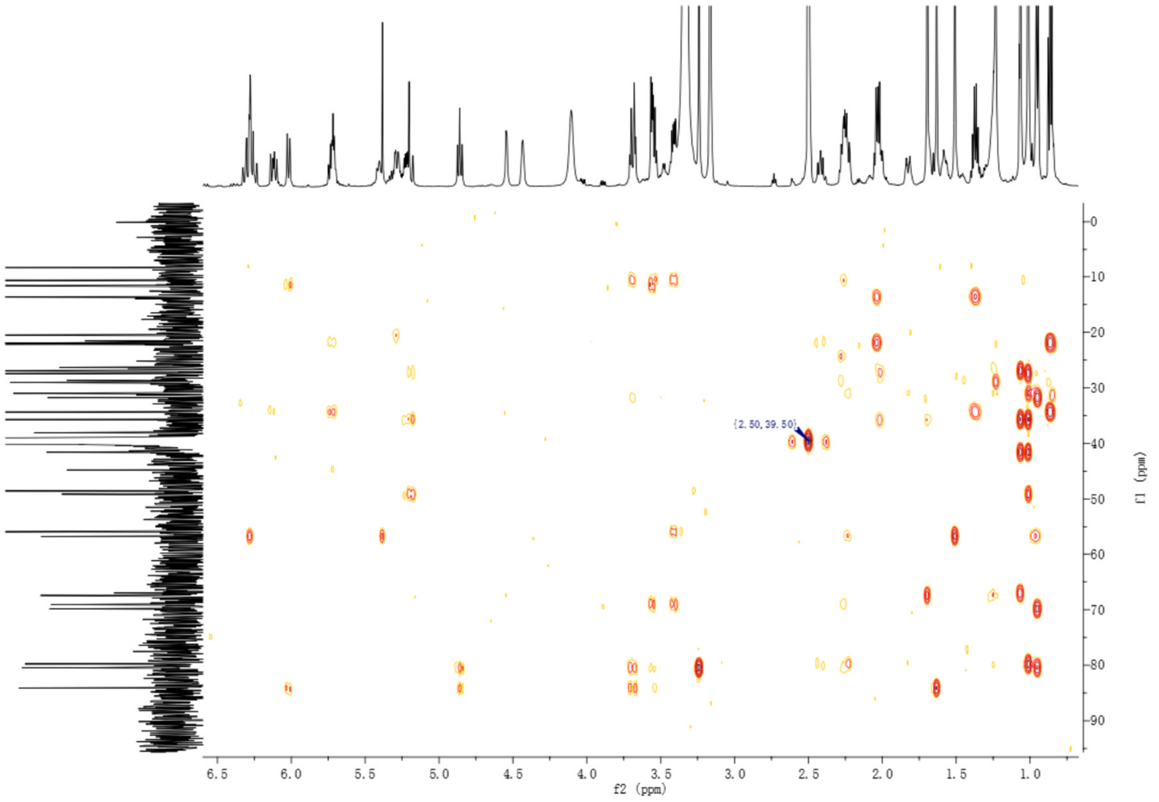

Figure S40. HRESIMS spectrum of penipyridinone B (**1**)

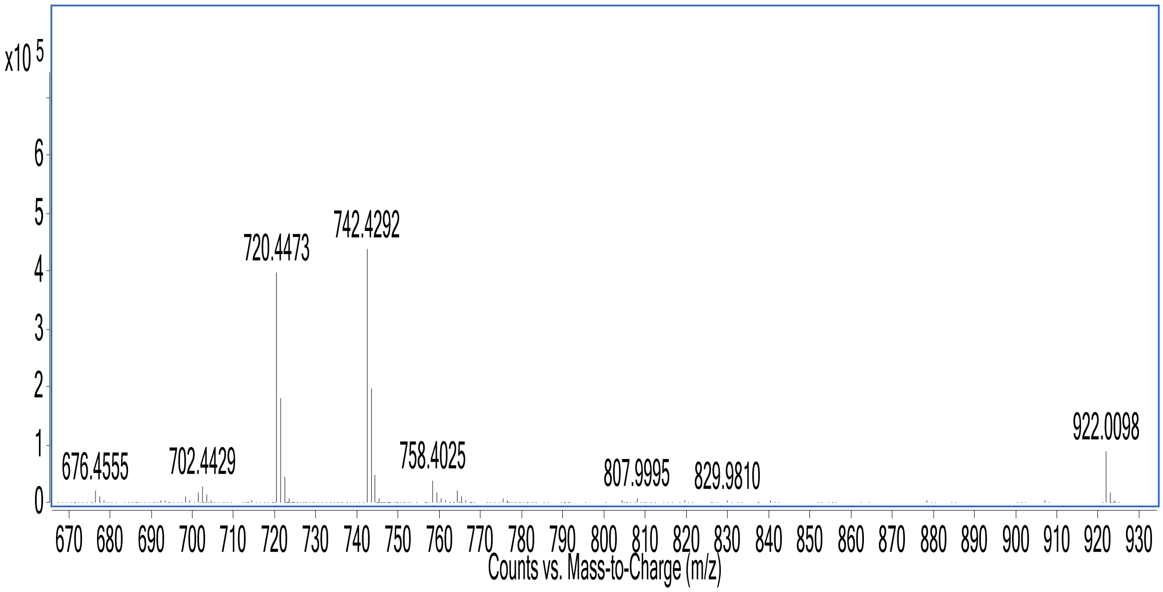

[M+H]$^+$: 720.4473 (calcd for $C_{43}H_{62}NO_8^+$, 720.4475), [M+Na]$^+$: 742.4292 (calcd for $C_{43}H_{61}NNaO_8^+$, 742.4295).

Figure S41. ^{1}H NMR spectrum of **1s**

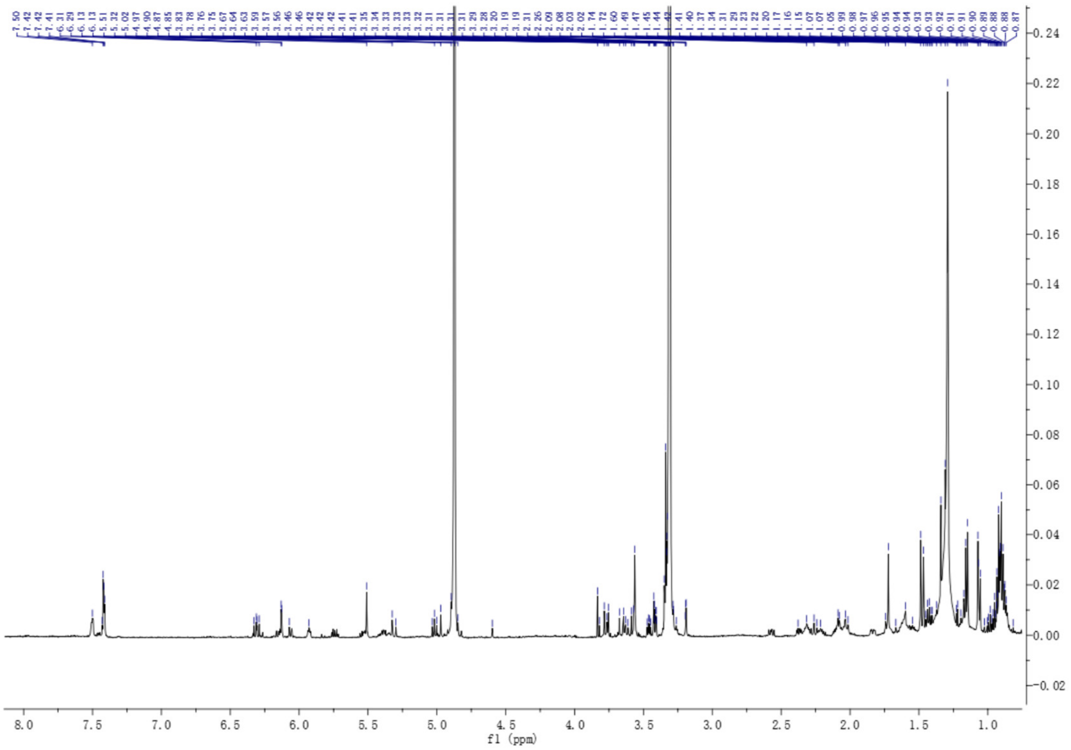

Figure S42. COSY NMR spectrum of **1s**

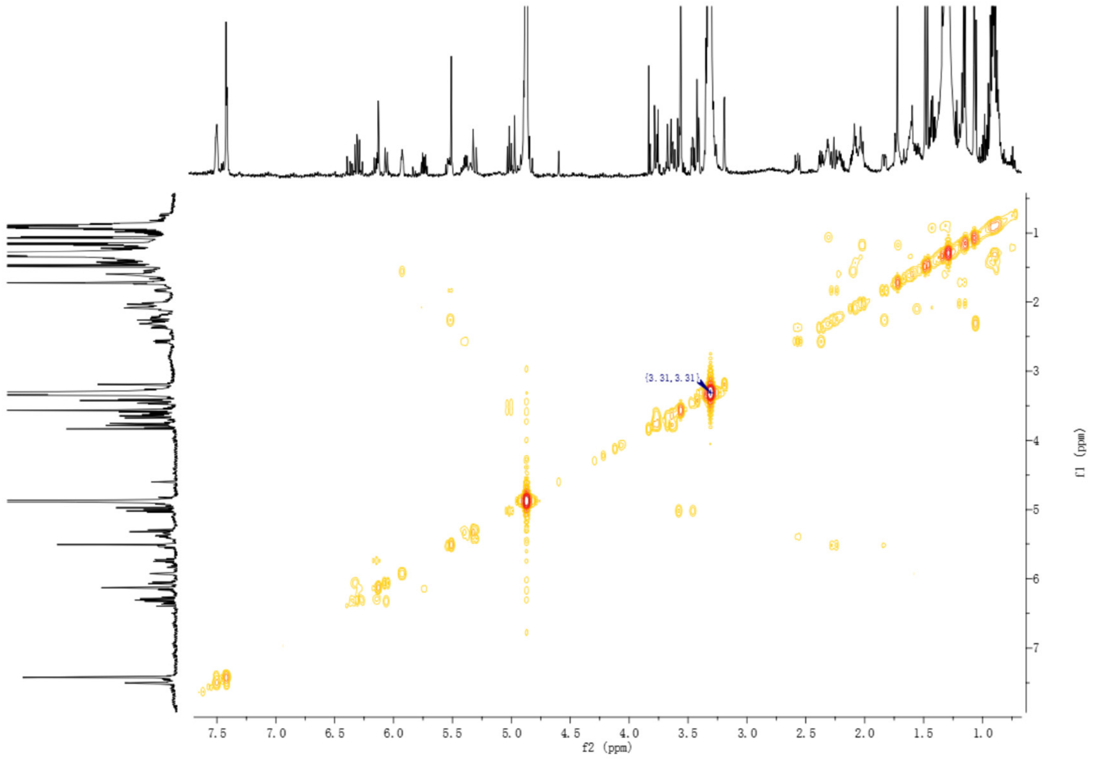

Figure S43. HRESIMS spectrum of **1s**

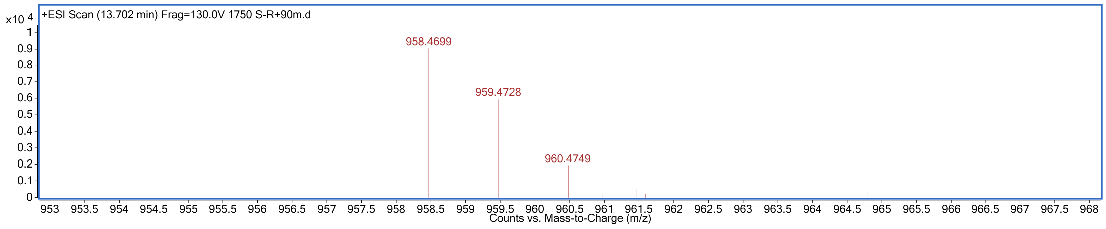

[M+Na]$^{+}$: 958.4699 (calcd for $C_{53}H_{68}F_{3}NNaO_{10}^{+}$, 958.4688).

Figure S44. ^{1}H NMR spectrum of **1r**

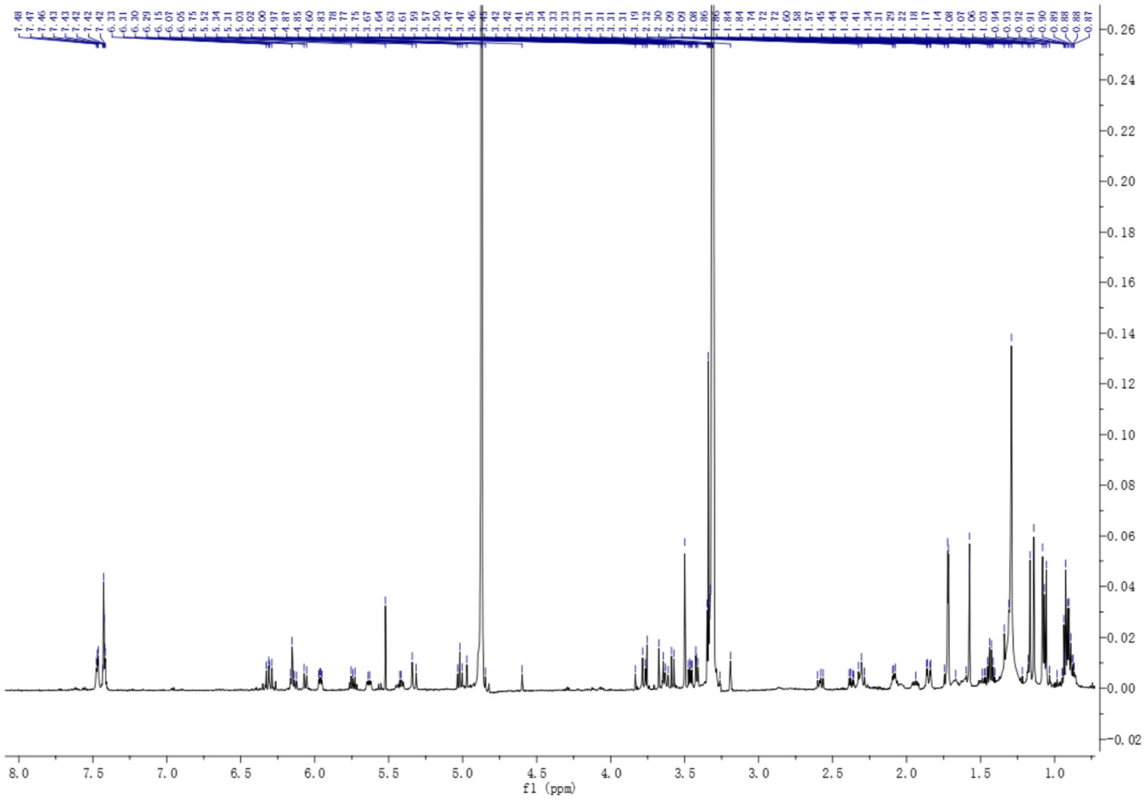

Figure S45. COSY spectrum of **1r**

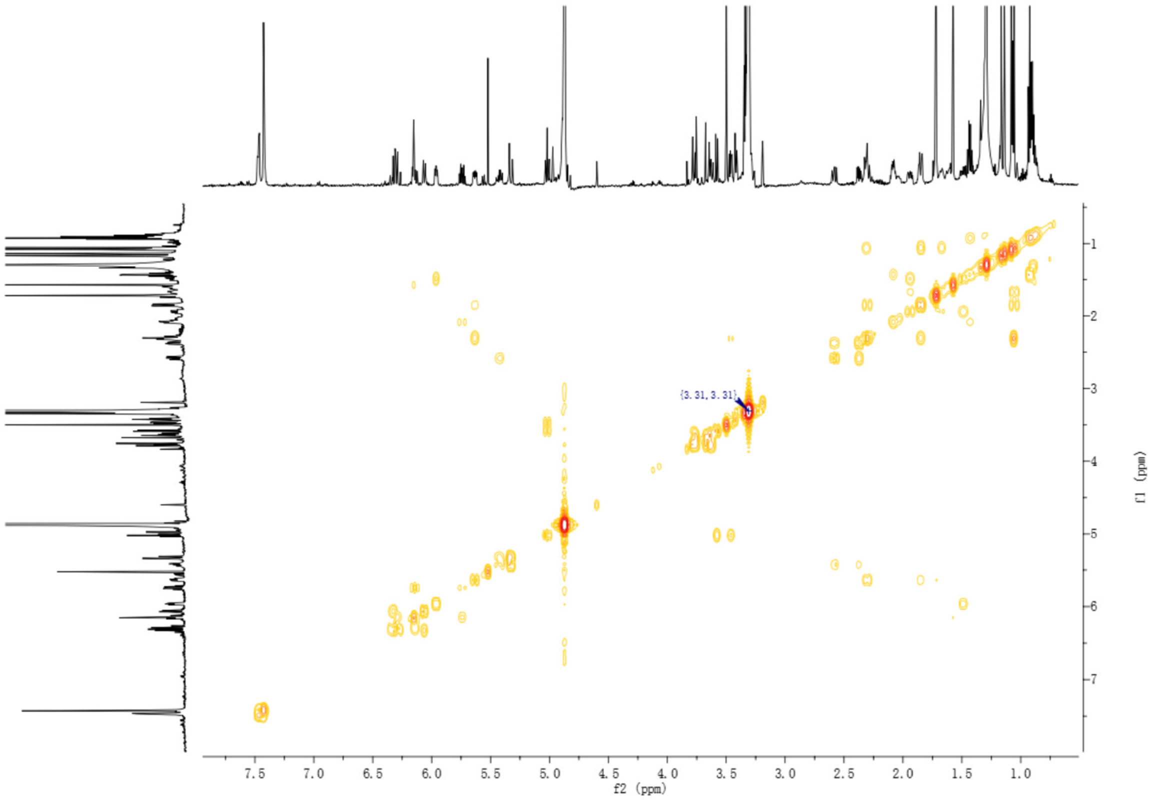

Figure S46. HRESIMS spectrum of **1r**

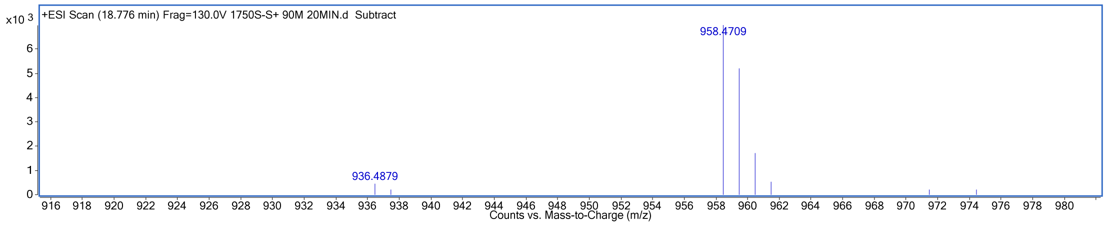

[M+H]$^+$: 936.4879 (calcd for $C_{53}H_{69}F_3NO_{10}^+$, 936.4868); [M+Na]$^+$: 958.4709 (calcd for $C_{53}H_{68}F_3NNaO_{10}^+$, 958.4688).

Figure S47. ^{1}H NMR spectrum of penilloid B (**2**, in DMSO-d_6)

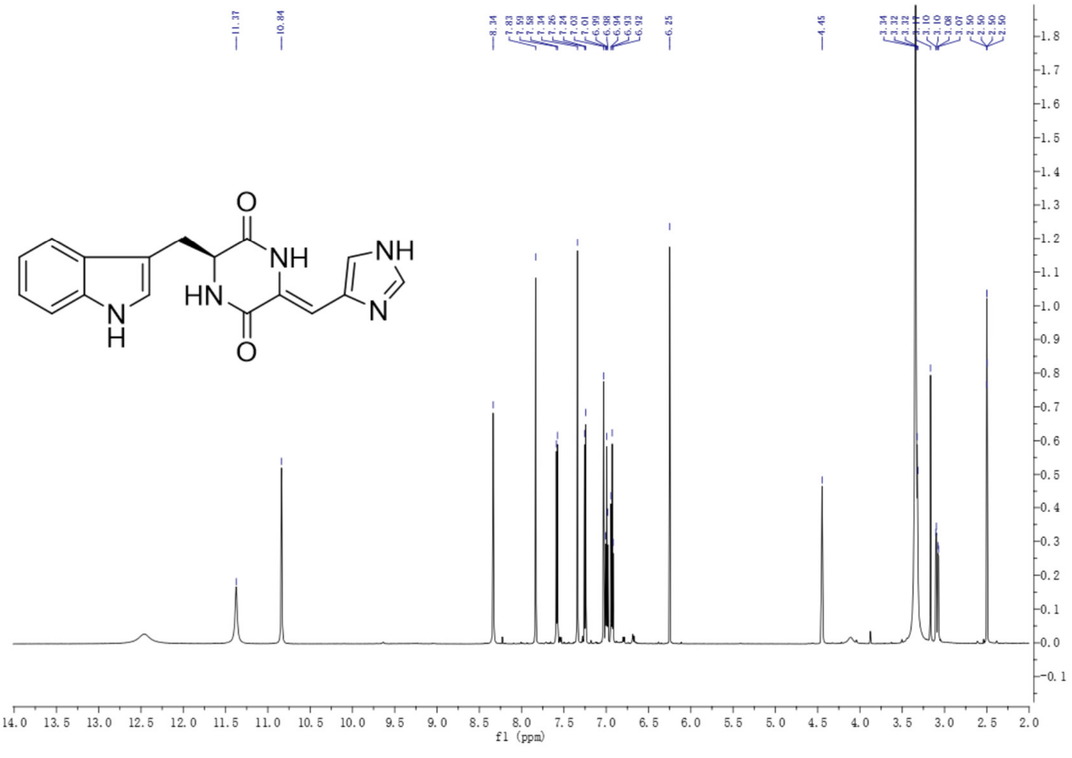

Figure S48. ^{1}H NMR spectrum of penilloid B (**2**, in DMSO-d_6)

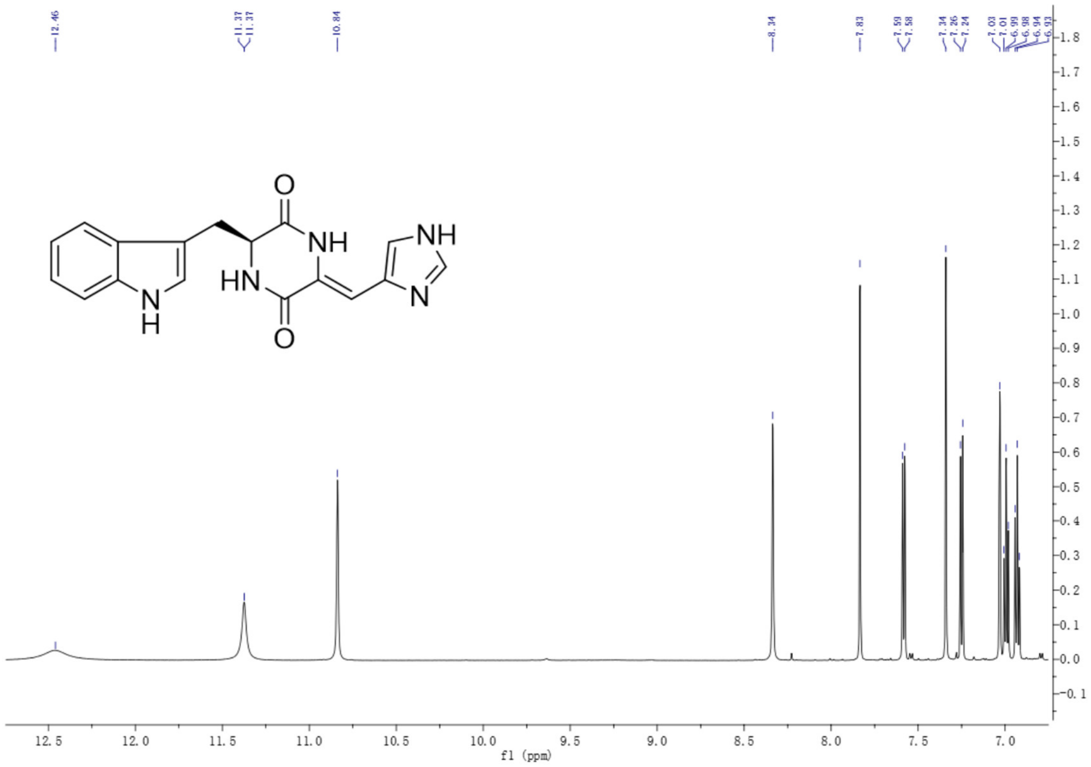

Figure S49. ^{1}H NMR spectrum of penilloid B (**2**, in DMSO-*d*$_6$)

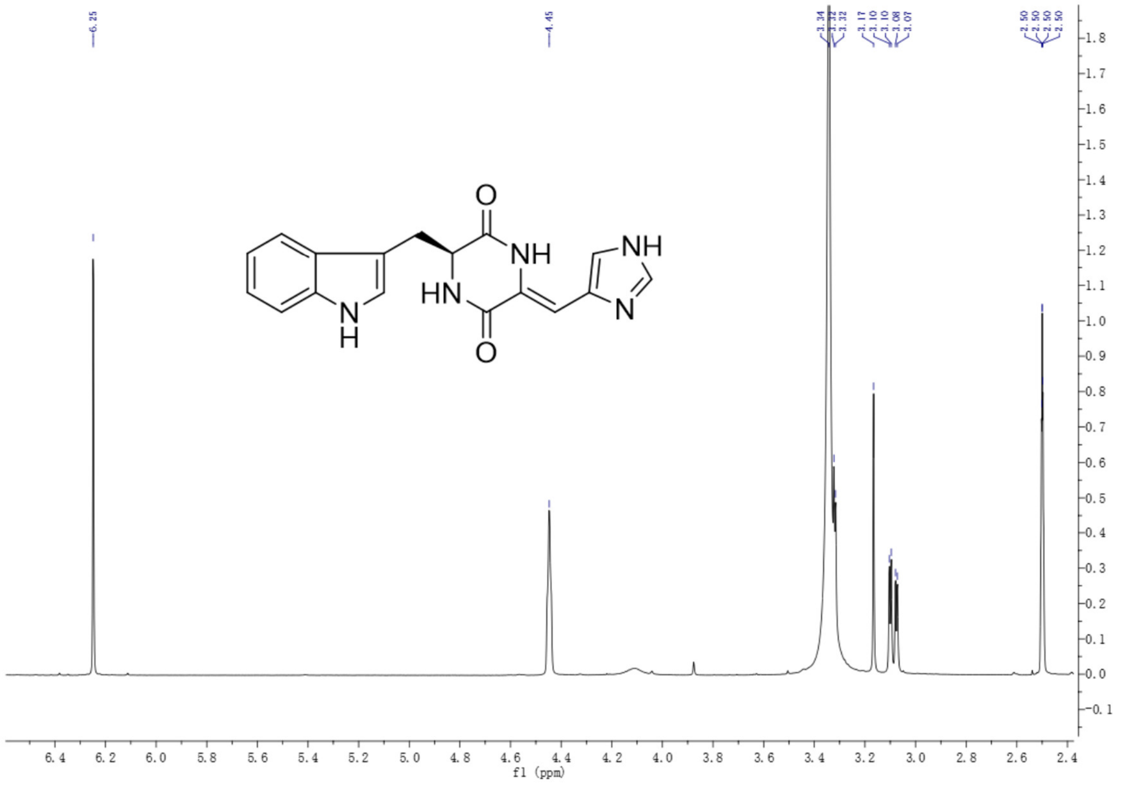

Figure S50. ^{13}C NMR spectrum of penilloid B (**2**, in DMSO-*d*$_6$)

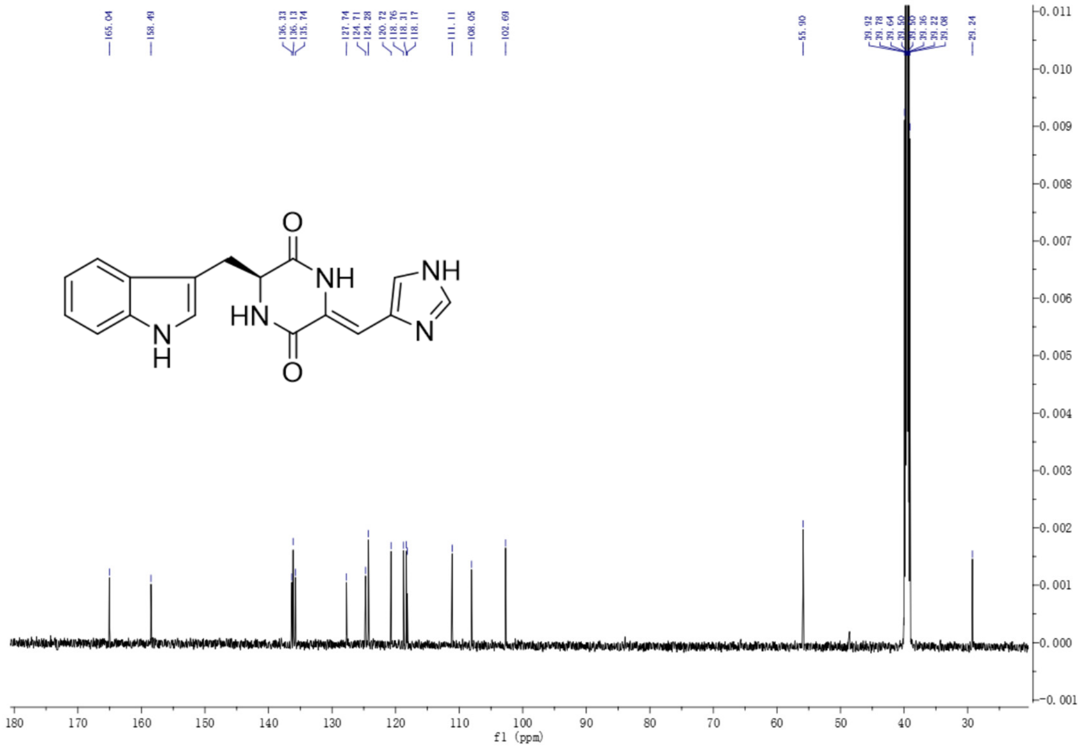

Figure S51. ^{13}C NMR spectrum of penilloid B (**2**, in DMSO-d_6)

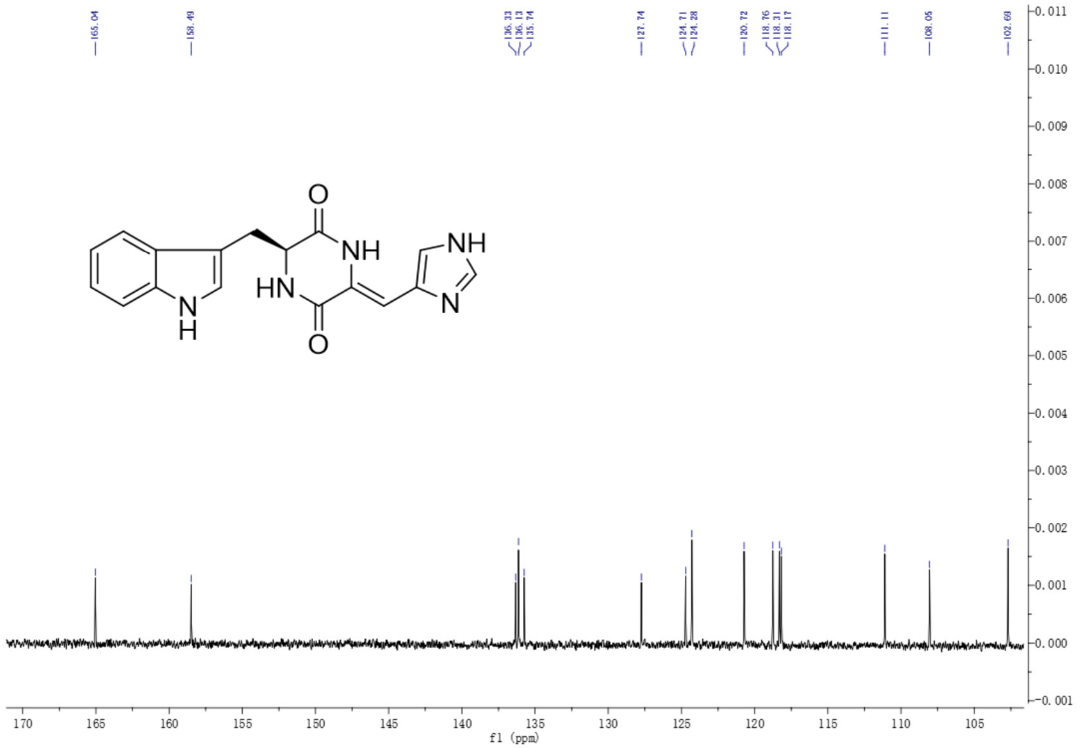

Figure S52. HMQC spectrum of penilloid B (**2**, in DMSO-d_6)

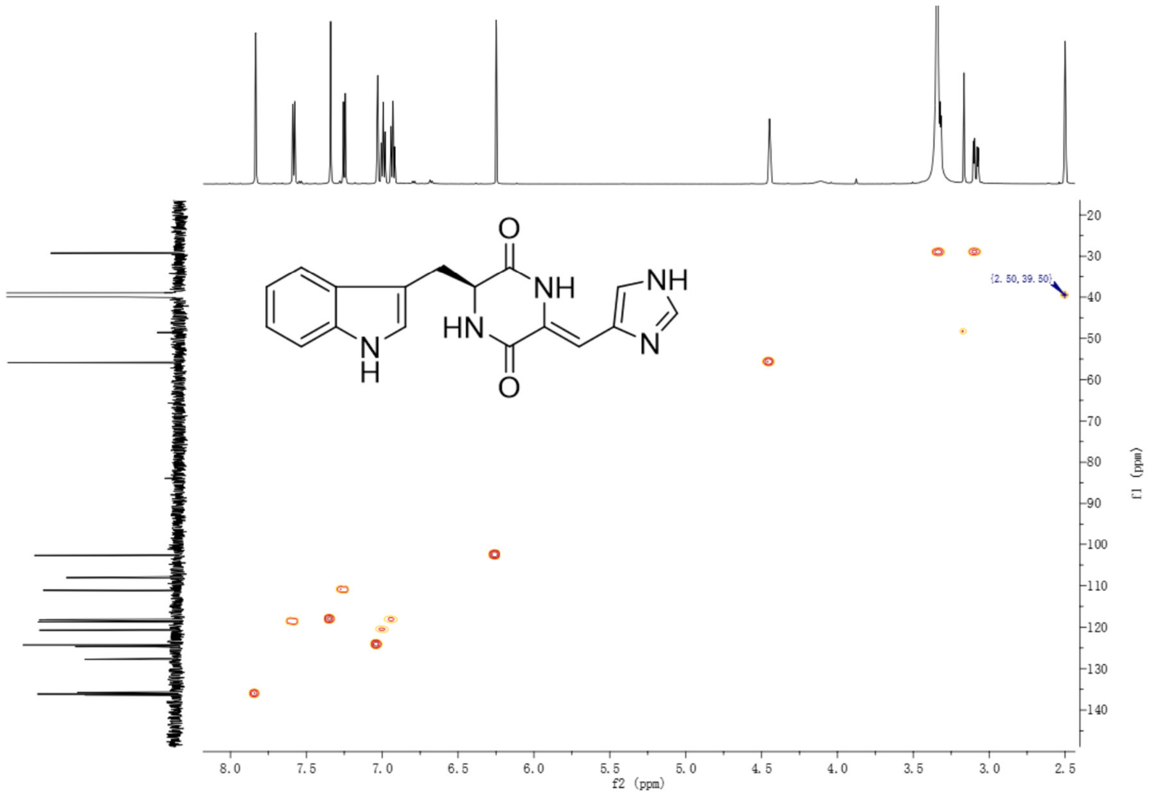

Figure S53. HMQC spectrum of penilloid B (**2**, in DMSO-d_6)

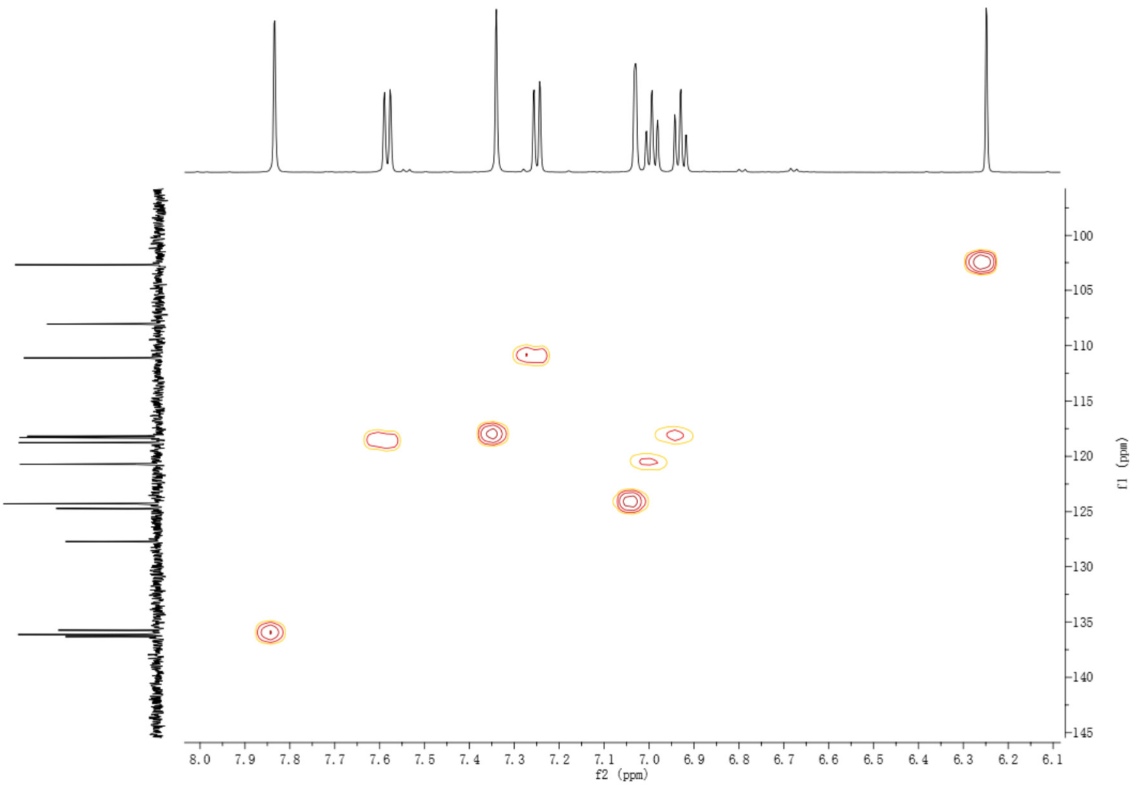

Figure S54. COSY spectrum of penilloid B (**2**, in DMSO-d_6)

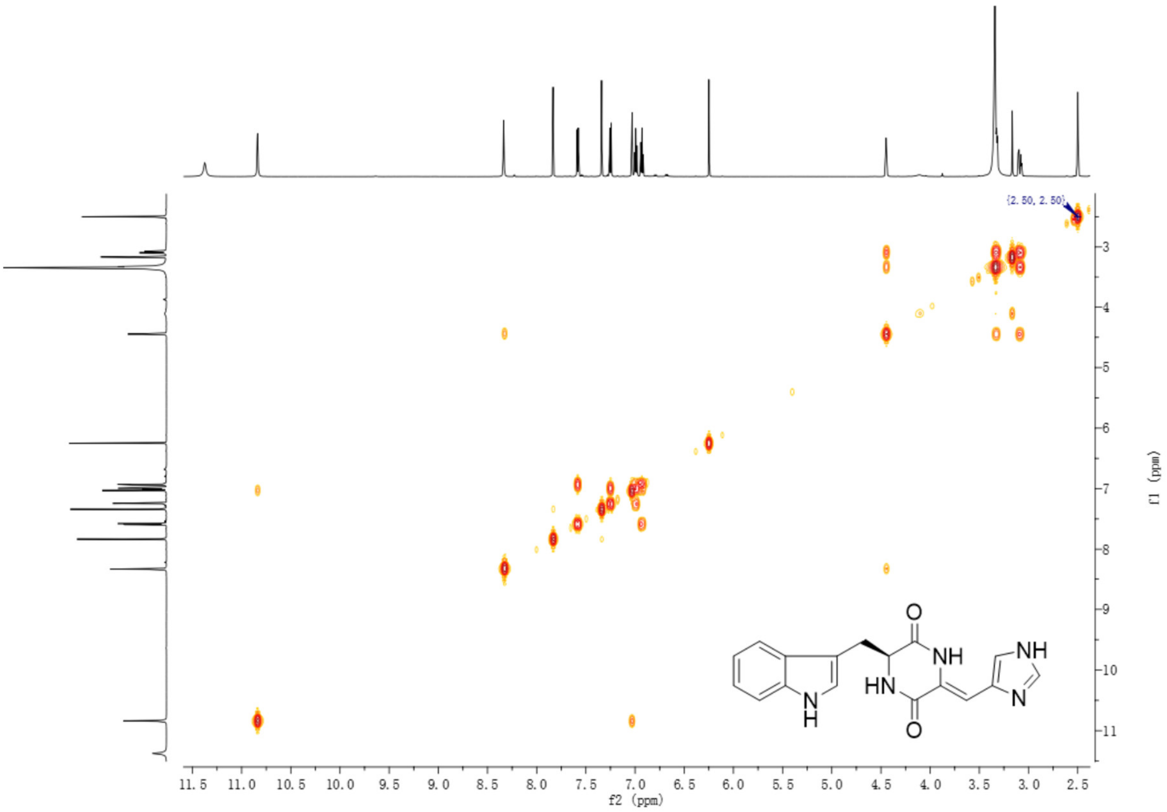

Figure S55. HMBC spectrum of penilloid B (**2**, in DMSO-d_6)

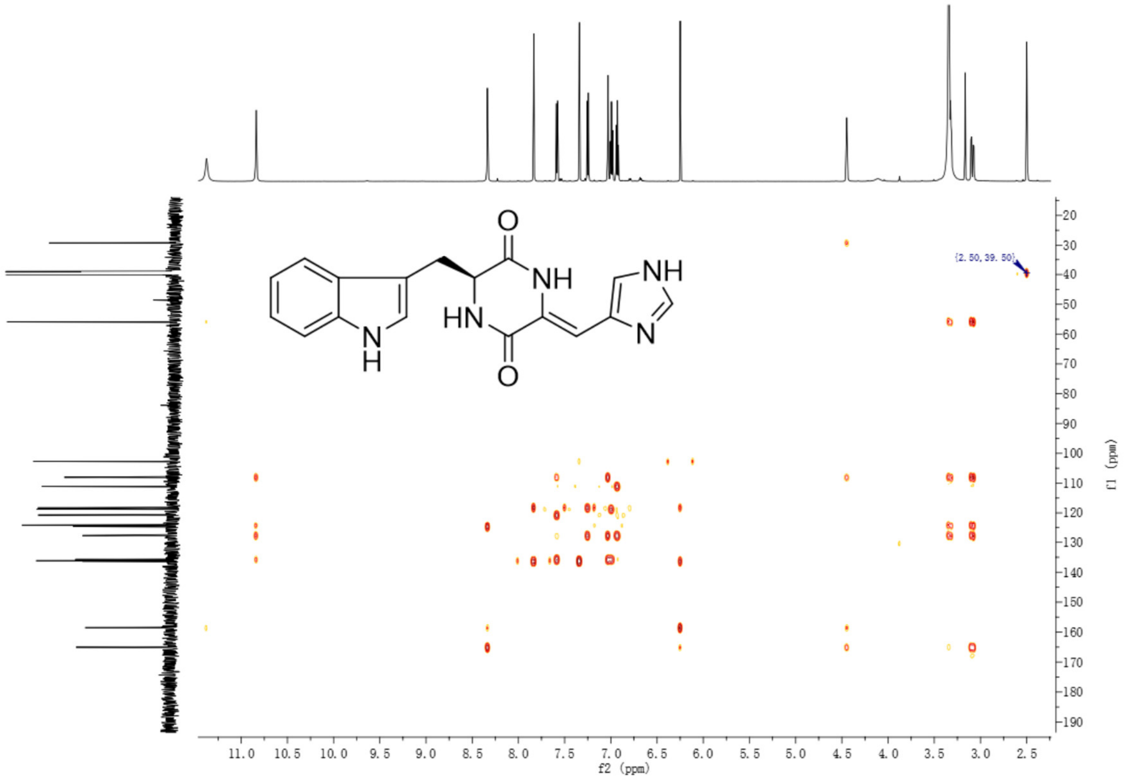

Figure S56. HMBC spectrum of penilloid B (**2**, in DMSO-d_6)

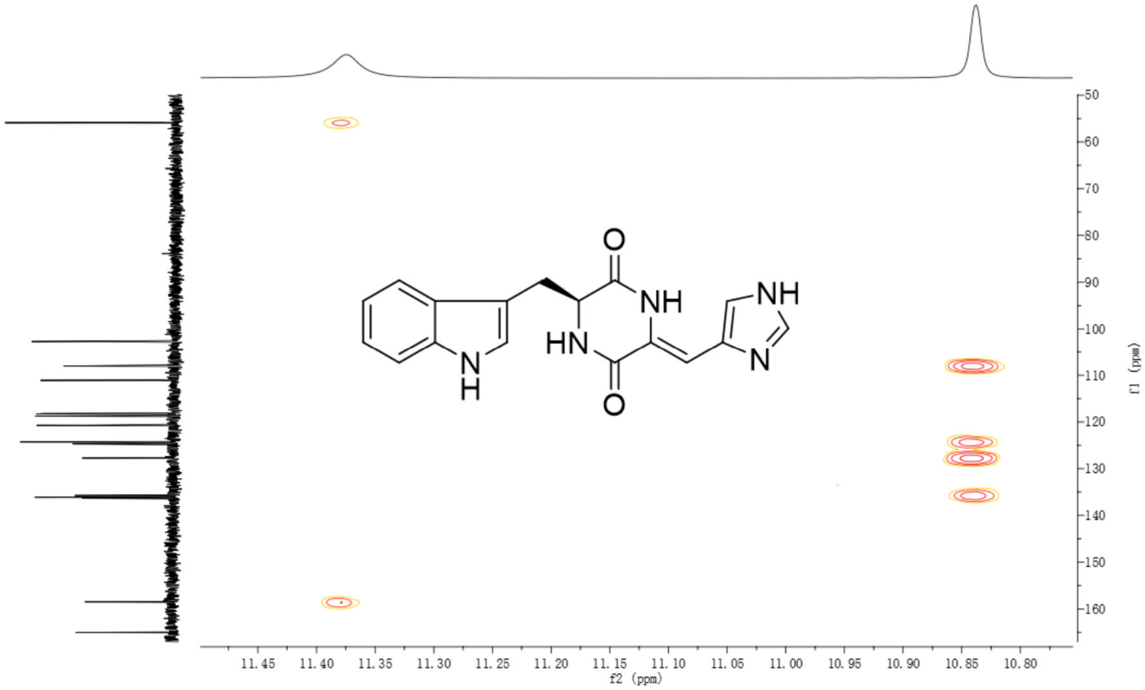

Figure S57. HMBC spectrum of penilloid B (**2**, in DMSO-*d*$_6$)

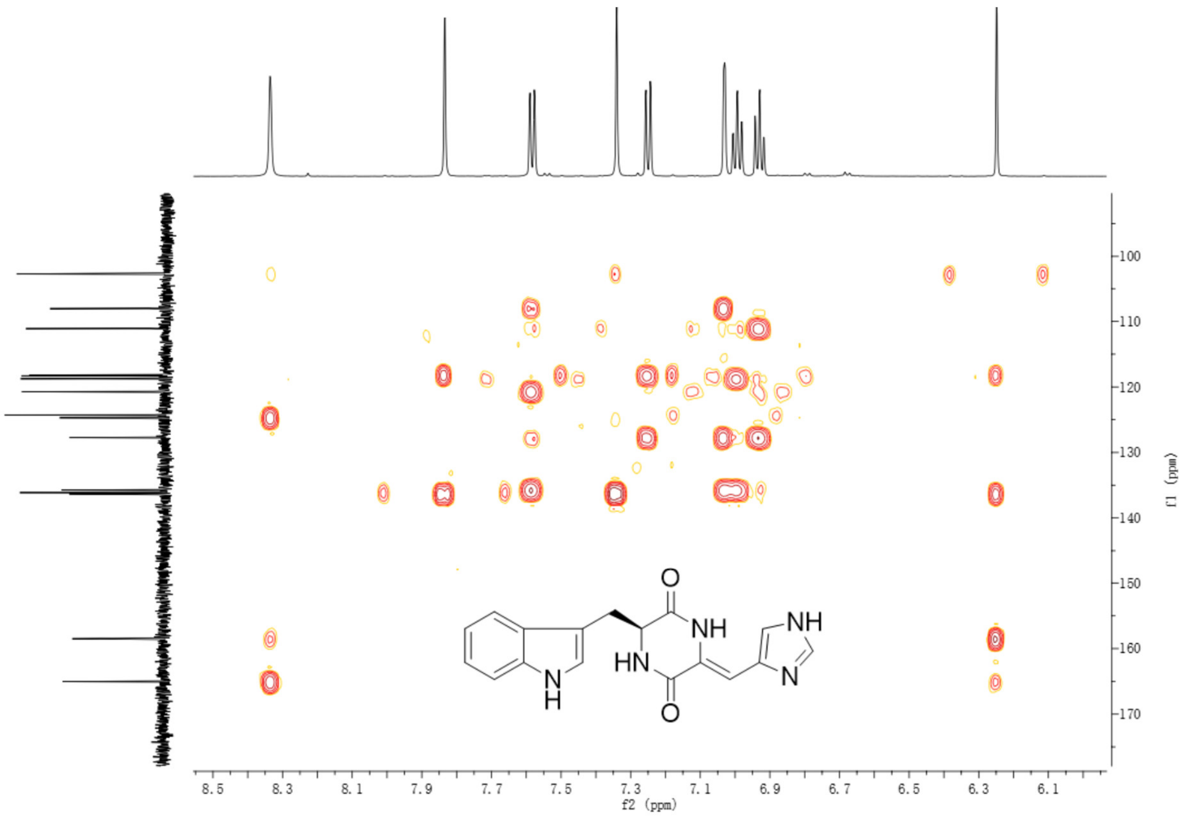

Figure S58. HMBC spectrum of penilloid B (**2**, in DMSO-*d*$_6$)

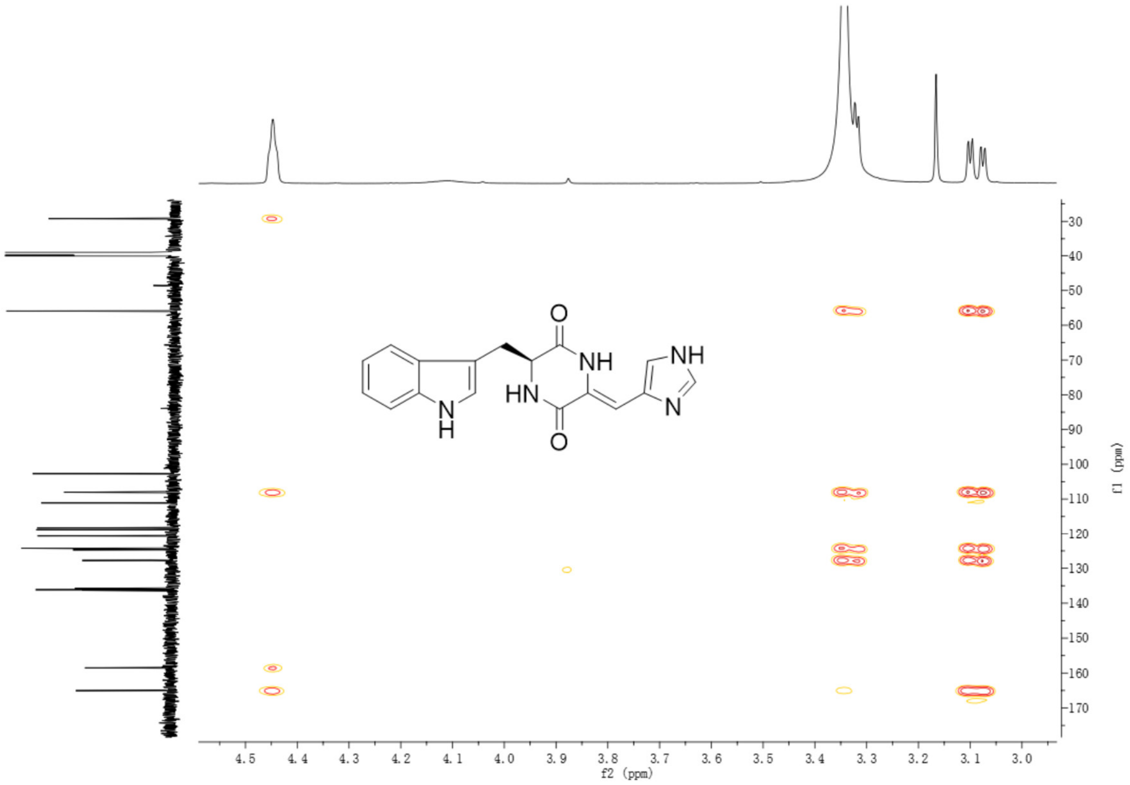

Figure S59. NOESY spectrum of penilloid B (**2**, in DMSO-d_6)

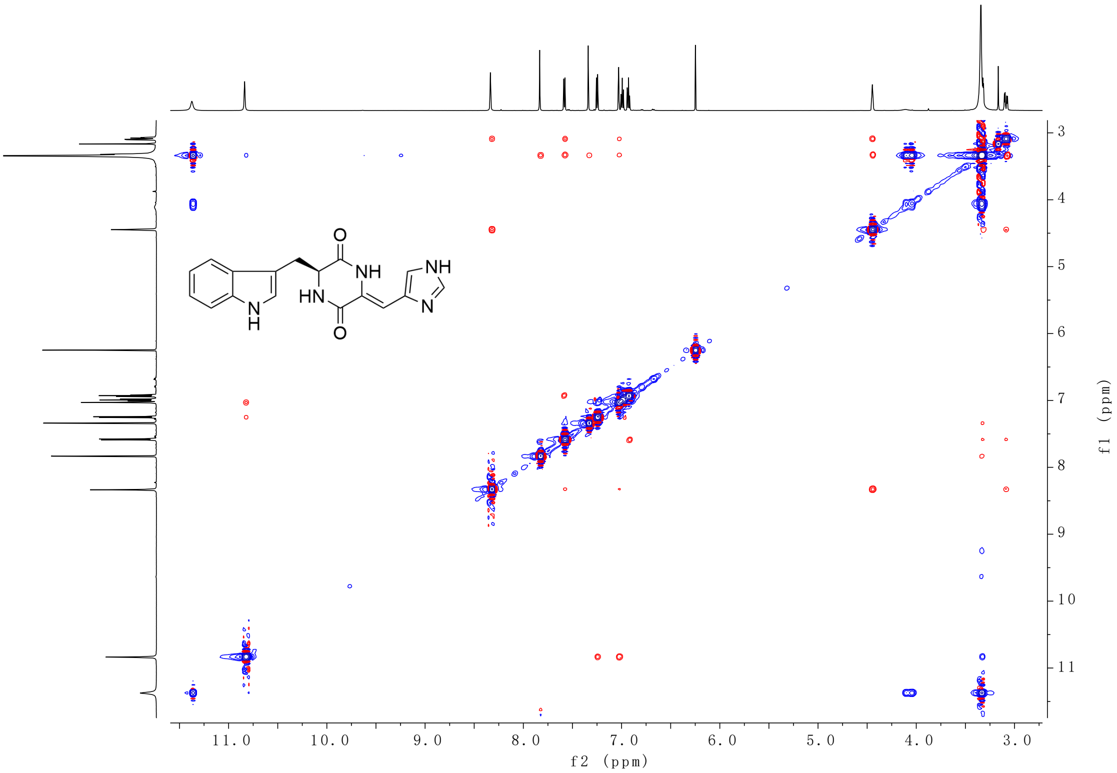

Figure S60. ^{1}H NMR spectrum of penilloid B (**2**, in MeOH-d_4)

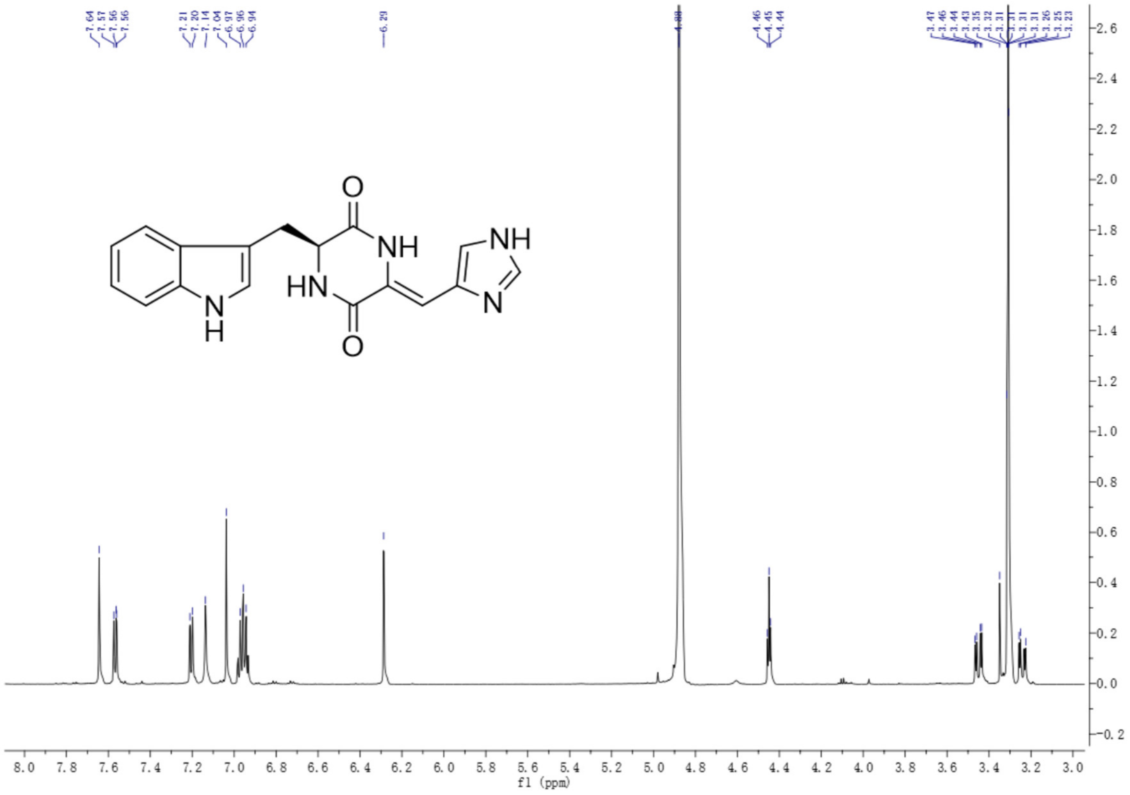

Figure S61. ^{1}H NMR spectrum of penilloid B (**2**, in MeOH-d_4)

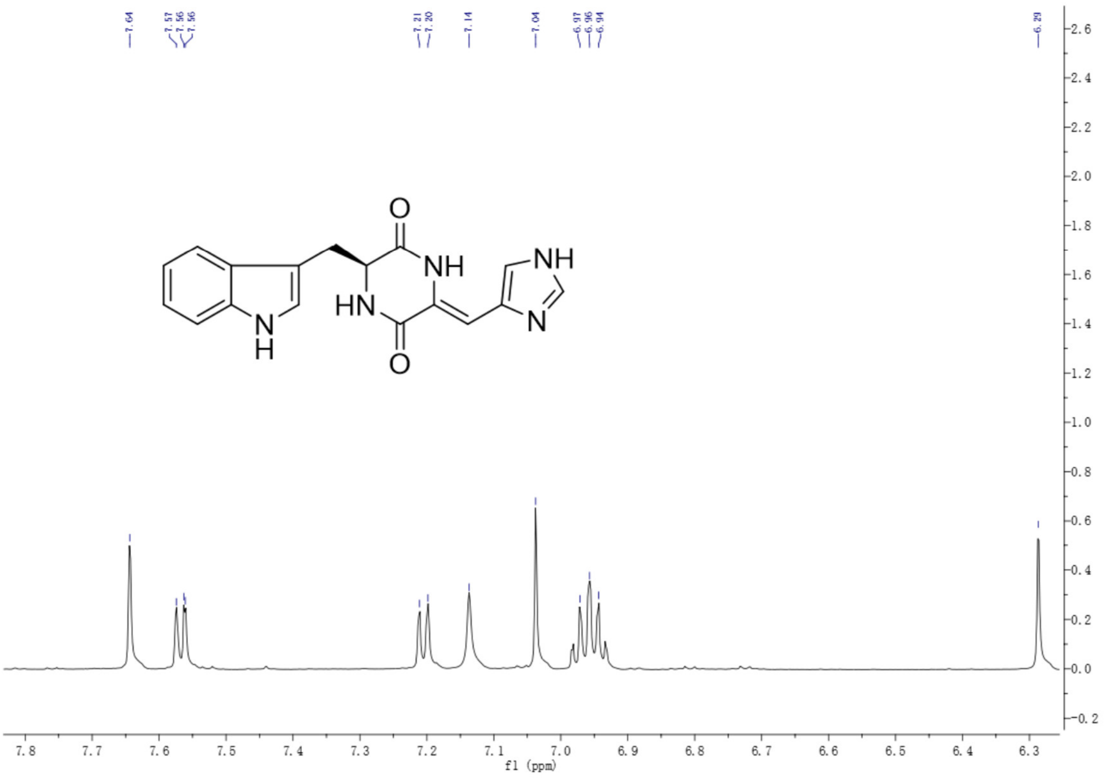

Figure S62. ^{1}H NMR spectrum of penilloid B (**2**, in MeOH-d_4)

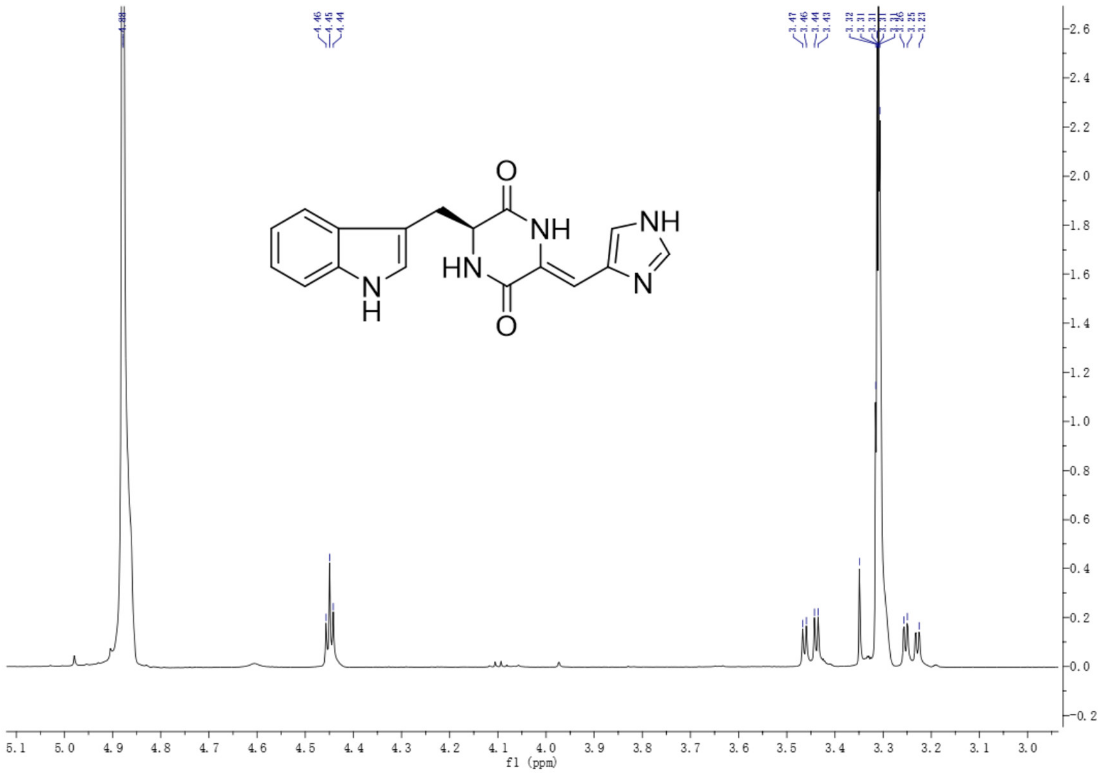

Figure S63. ^{13}C NMR spectrum of penilloid B (**2**, in MeOH-d_4)

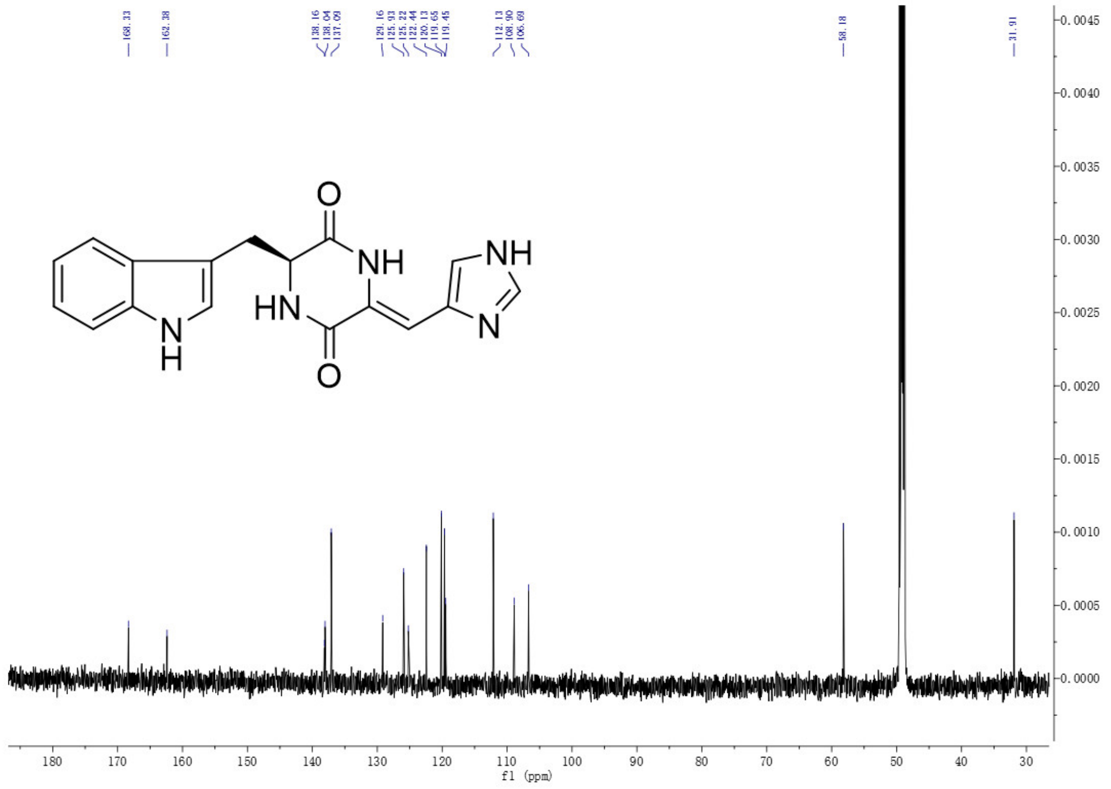

Figure S64. ^{13}C NMR spectrum of penilloid B (**2**, in MeOH-d_4)

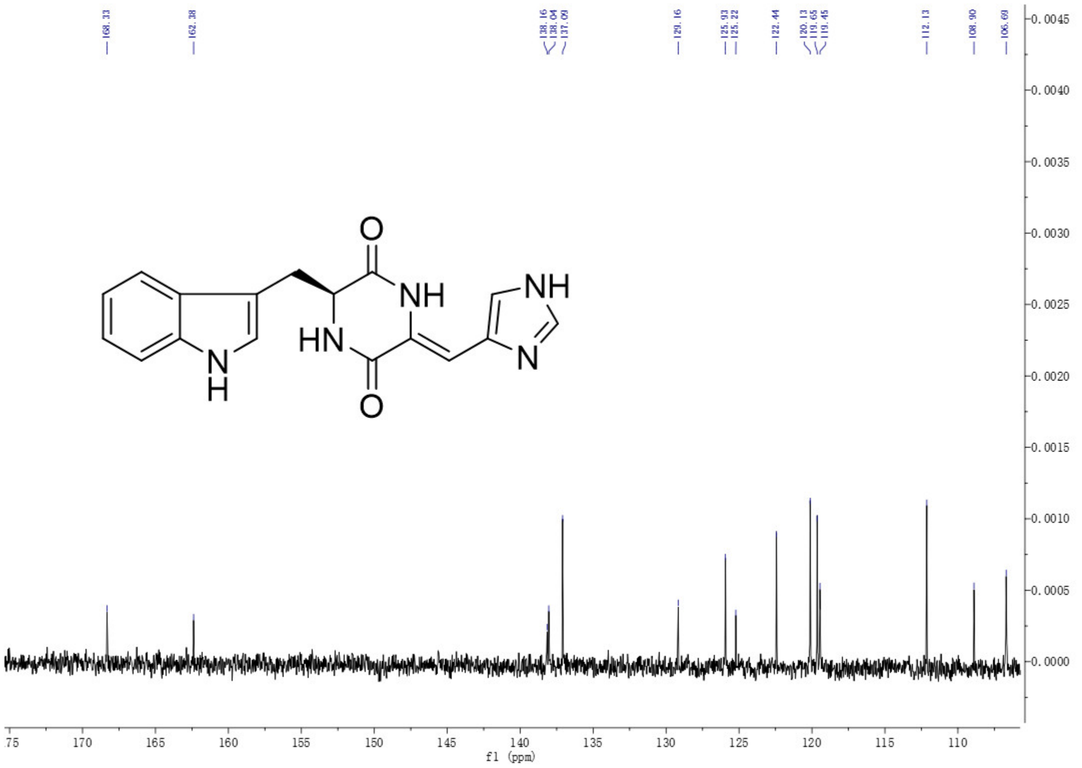

Figure S65. HRESIMS spectrum of penilloid B (**2**)

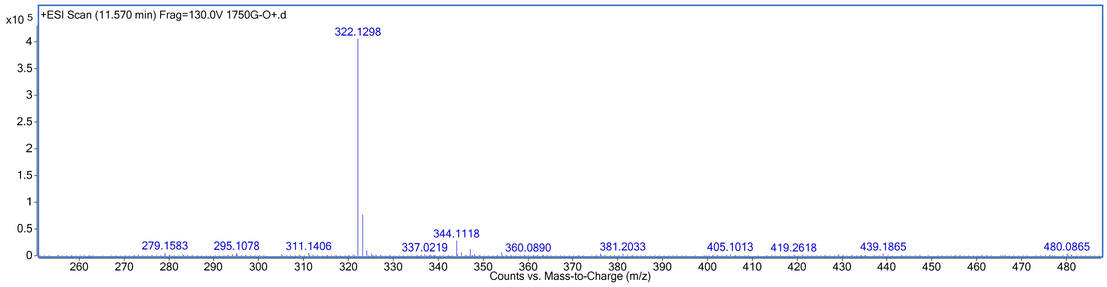

[M+H]$^+$: *m/z* 322.1298 (calcd for $C_{17}H_{16}N_5O_2^+$, 322.1299); [M+Na]$^+$ *m/z* 344.1118 (calcd for $C_{17}H_{15}N_5NaO_2^+$, 344.1118).

Figure S66. ^{1}H NMR spectrum of penilloid C (**3**, in DMSO-d_6)

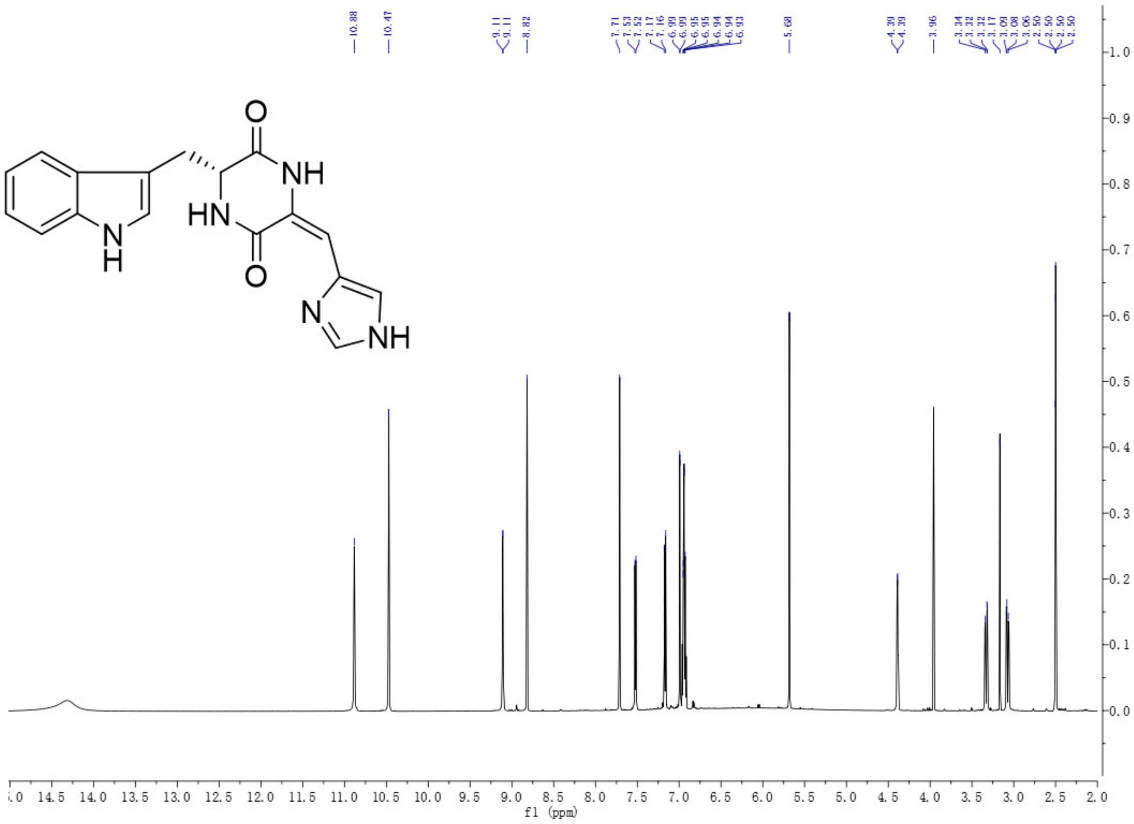

Figure S67. ^{1}H NMR spectrum of penilloid C (**3**, in DMSO-d_6)

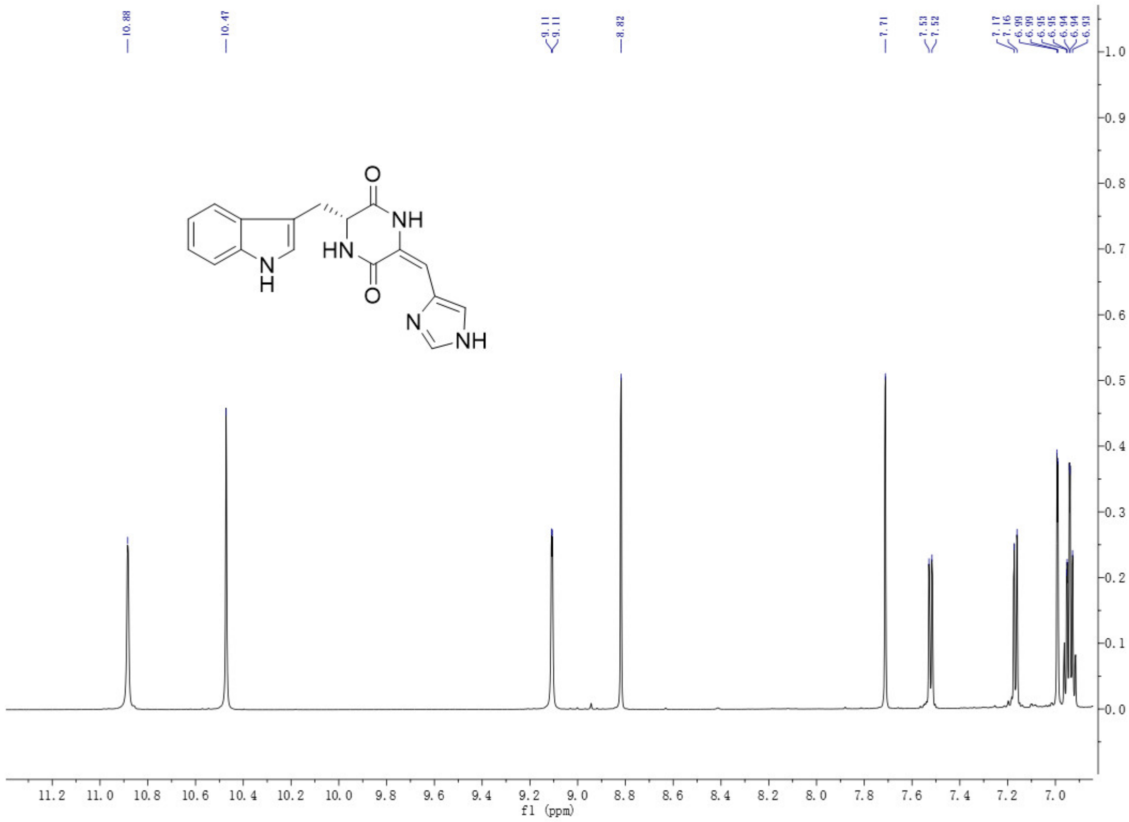

Figure S68. ^{1}H NMR spectrum of penilloid C (**3**, in DMSO-d_6)

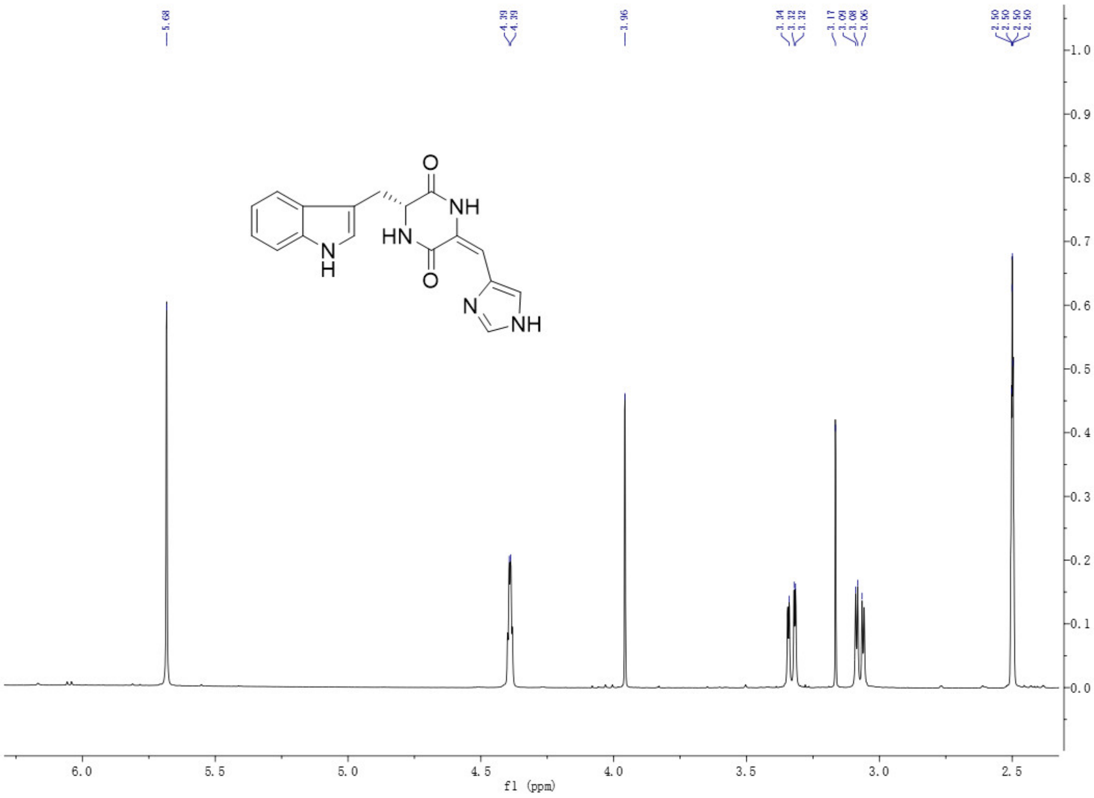

Figure S69. ^{13}C NMR spectrum of penilloid C (**3**, in DMSO-d_6)

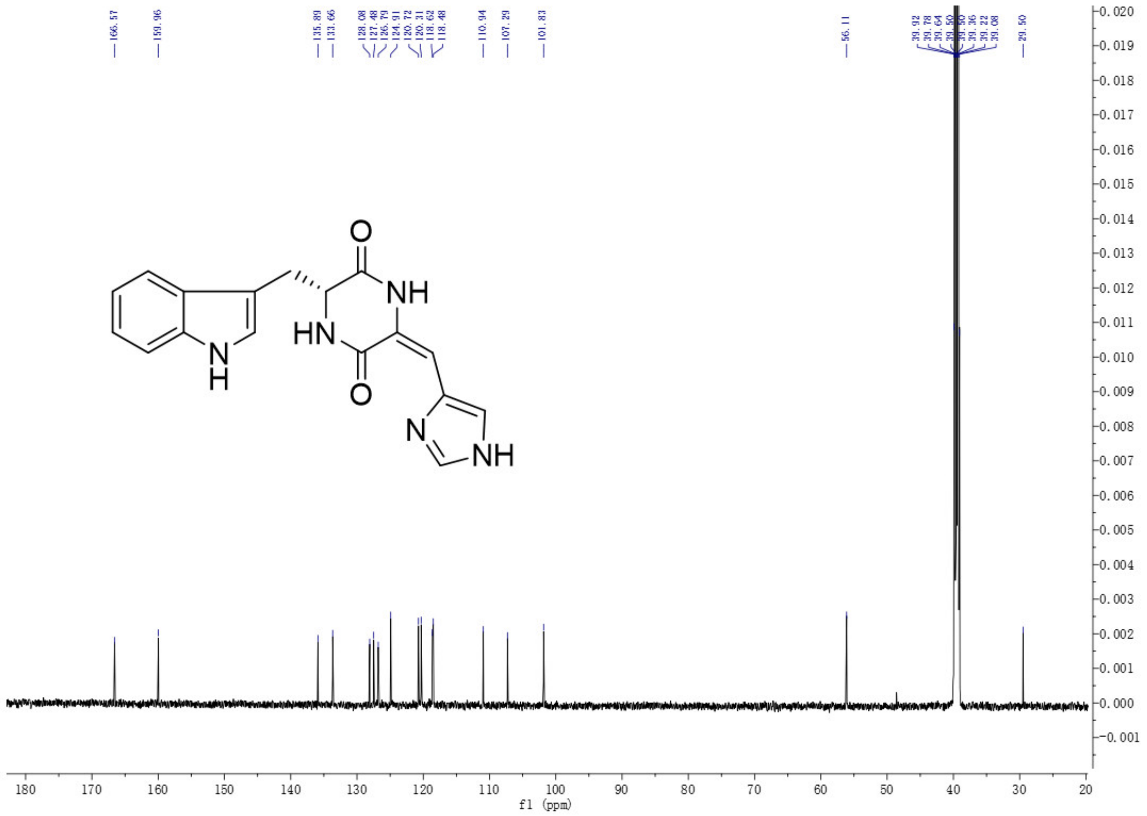

Figure S70. ^{13}C NMR spectrum of penilloid C (**3**, in DMSO-d_6)

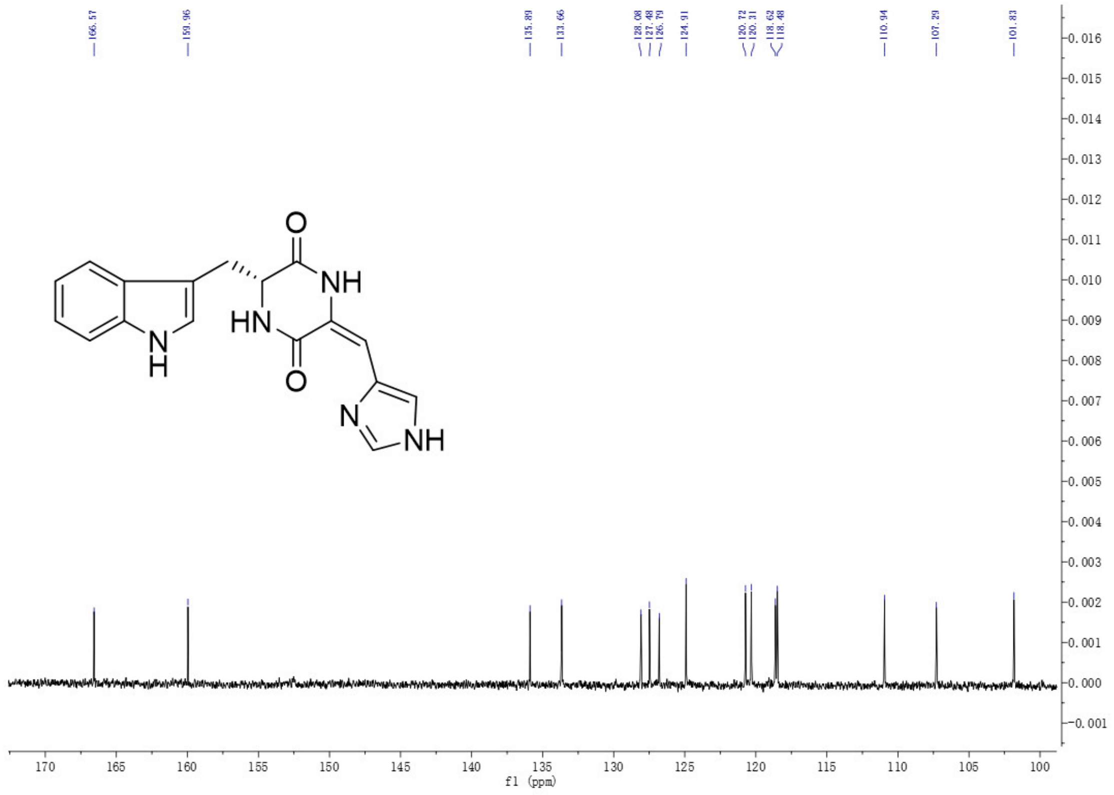

Figure S71. HMQC spectrum of penilloid C (**3**, in DMSO-d_6)

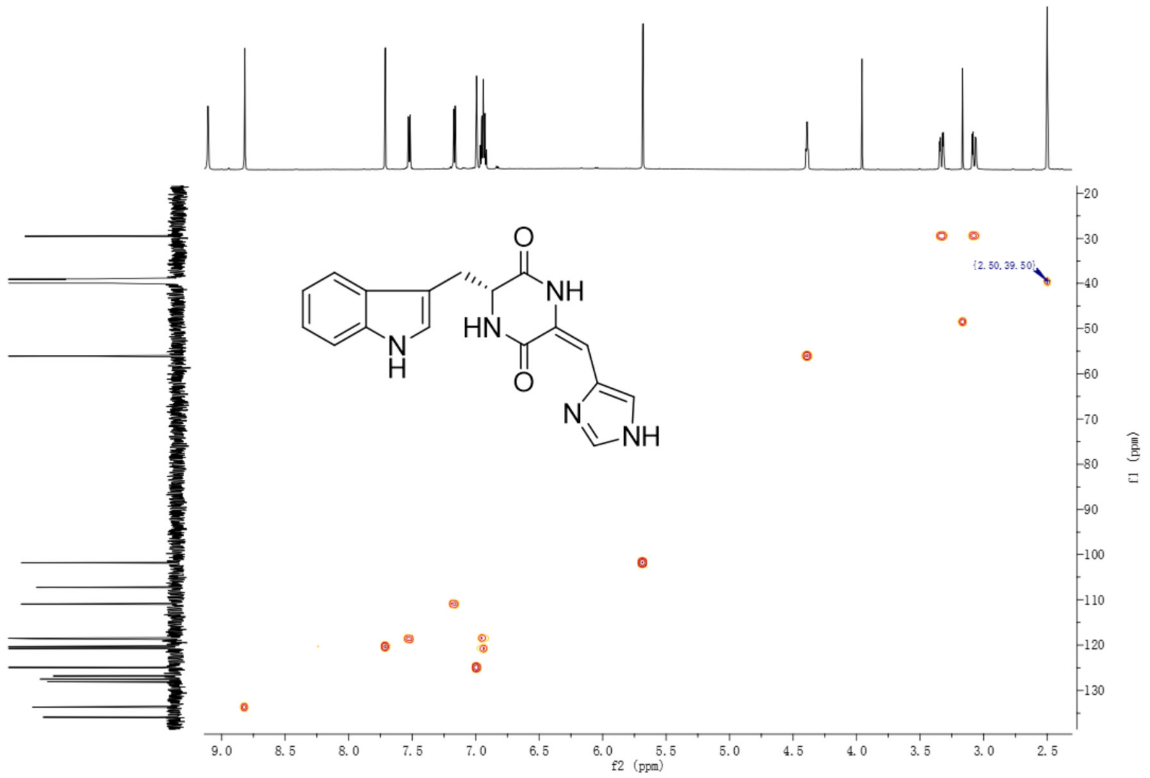

Figure S72. HMQC spectrum of penilloid C (**3**, in DMSO-d_6)

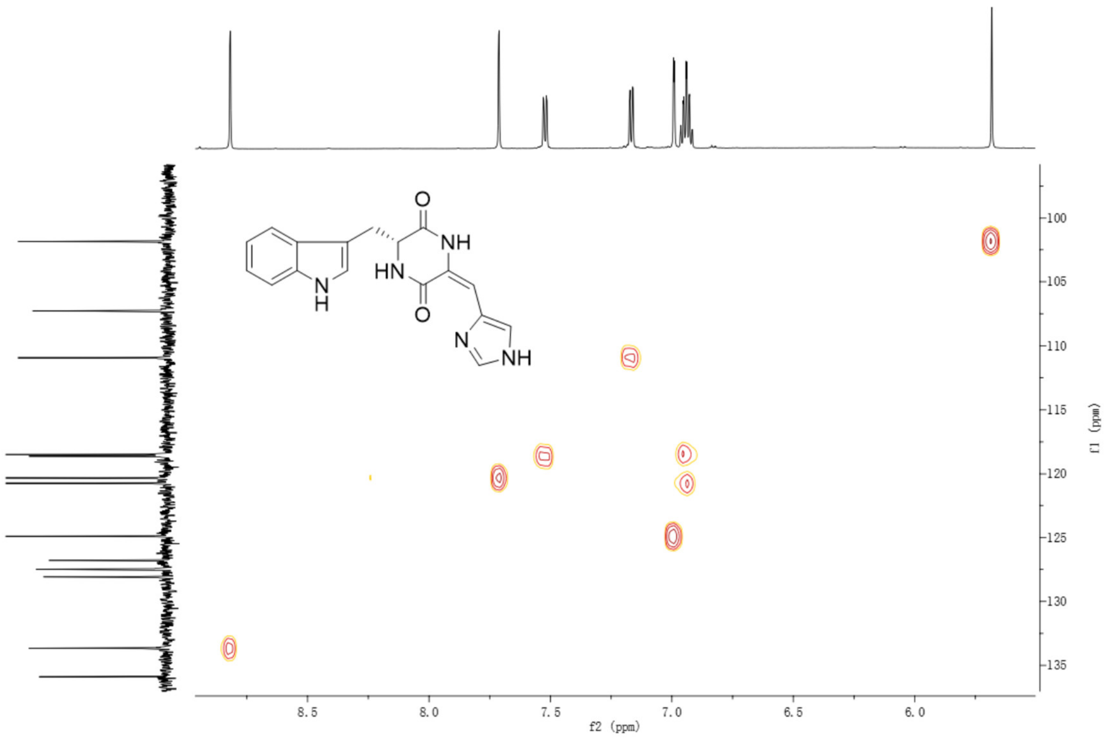

Figure S73. COSY spectrum of penilloid C (**3**, in DMSO-d_6)

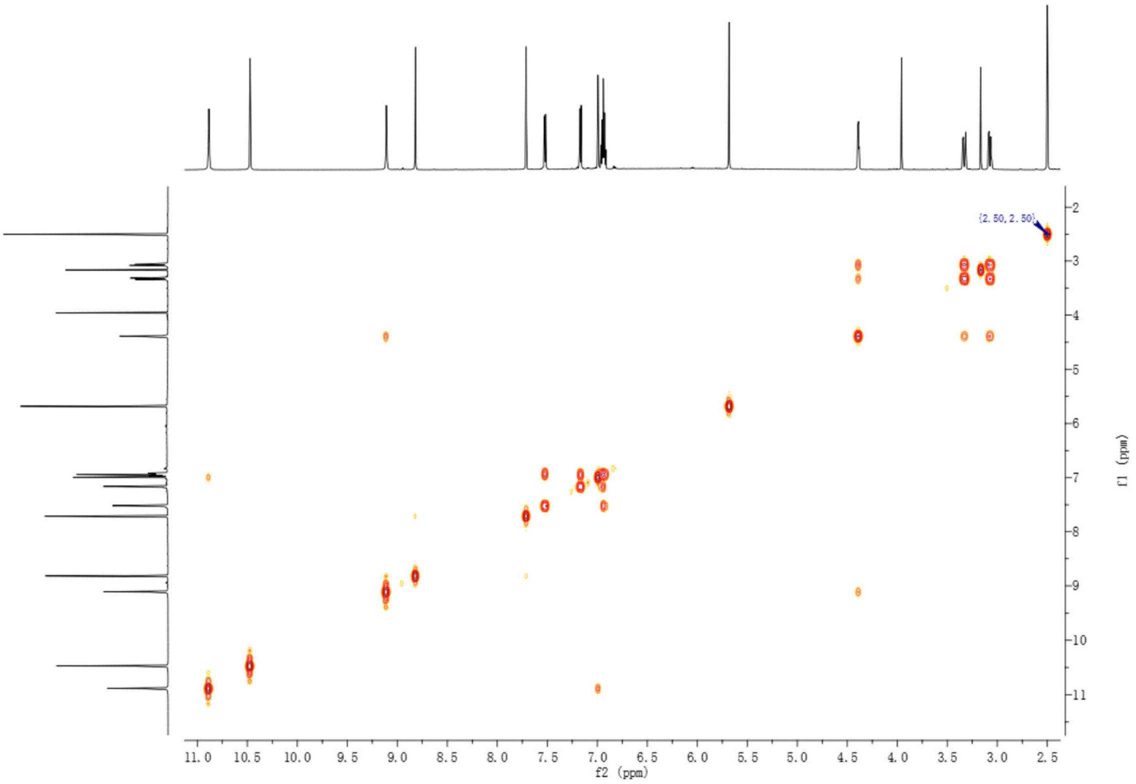

Figure S74. HMBC spectrum of penilloid C (**3**, in DMSO-d_6)

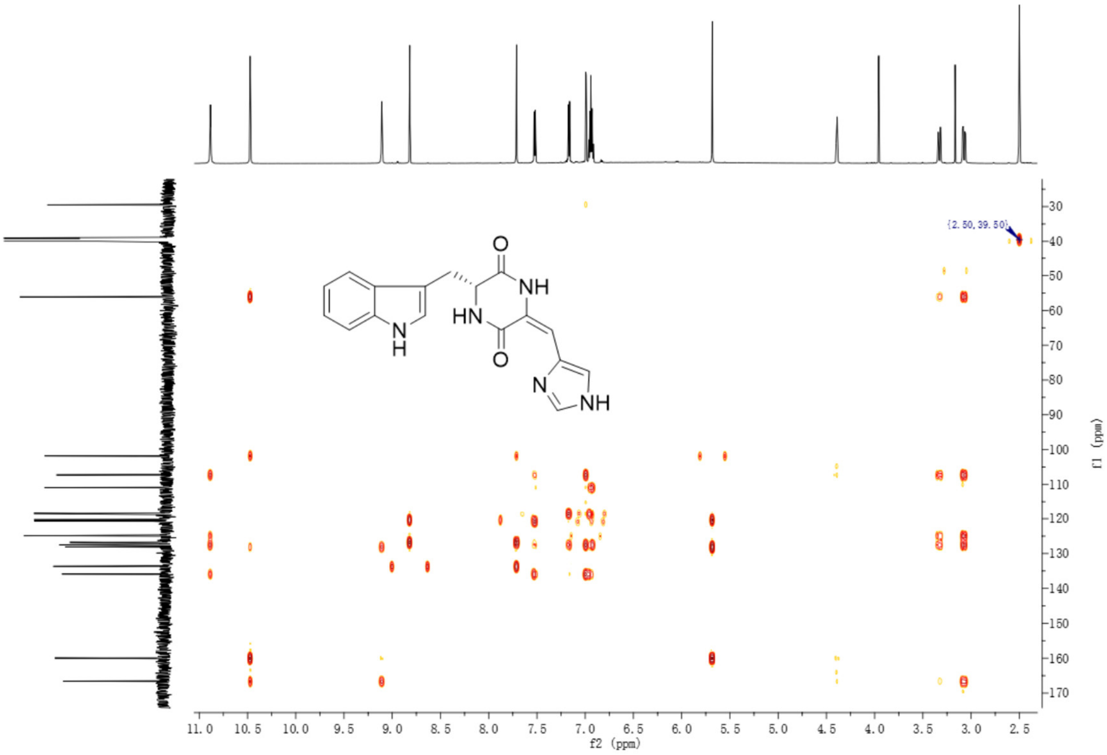

Figure S75. HMBC spectrum of penilloid C (**3**, in DMSO-d_6)

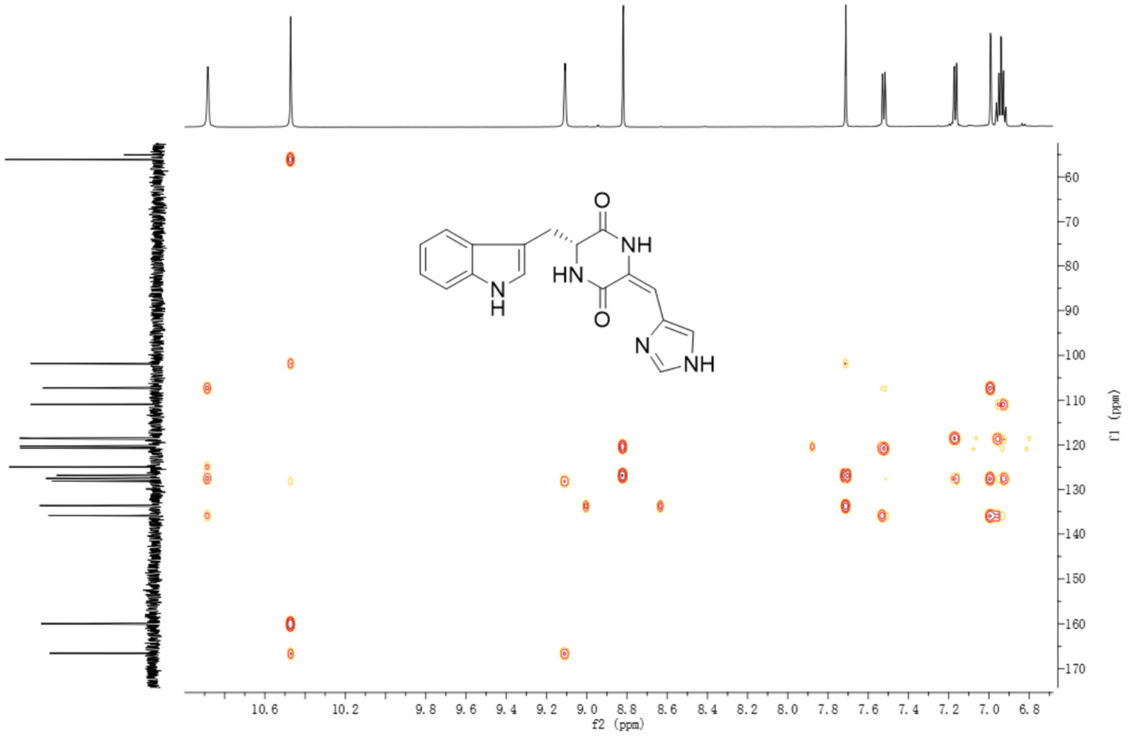

Figure S76. HMBC spectrum of penilloid C (**3**, in DMSO-d_6)

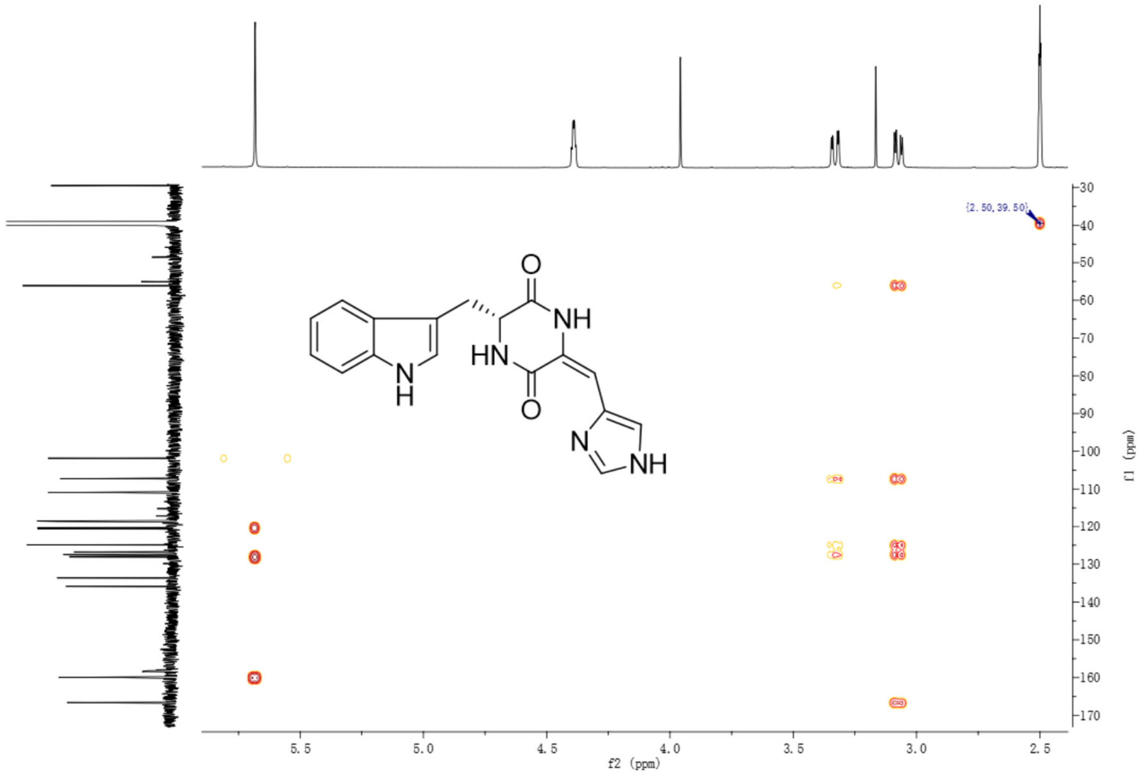

Figure S77. NOESY spectrum of penilloid C (**3**, in DMSO-d_6)

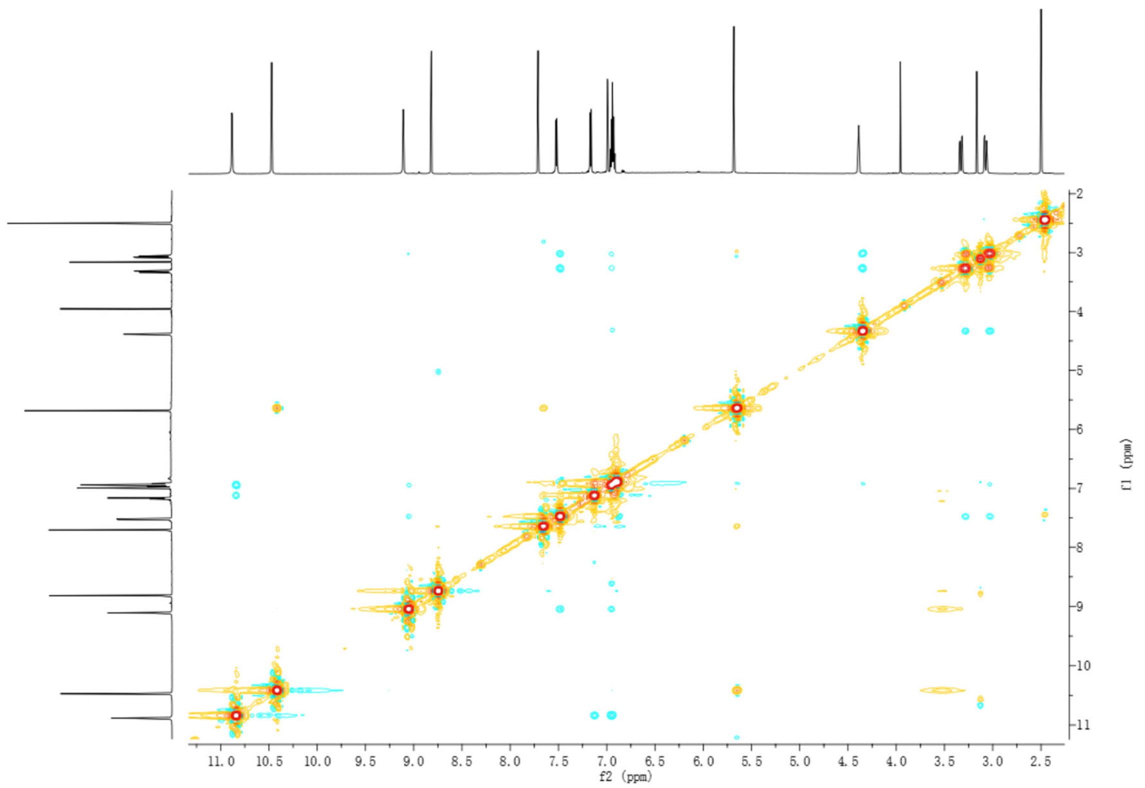

Figure S78. ^{1}H NMR spectrum of penilloid C (**3**, in MeOH-d_4)

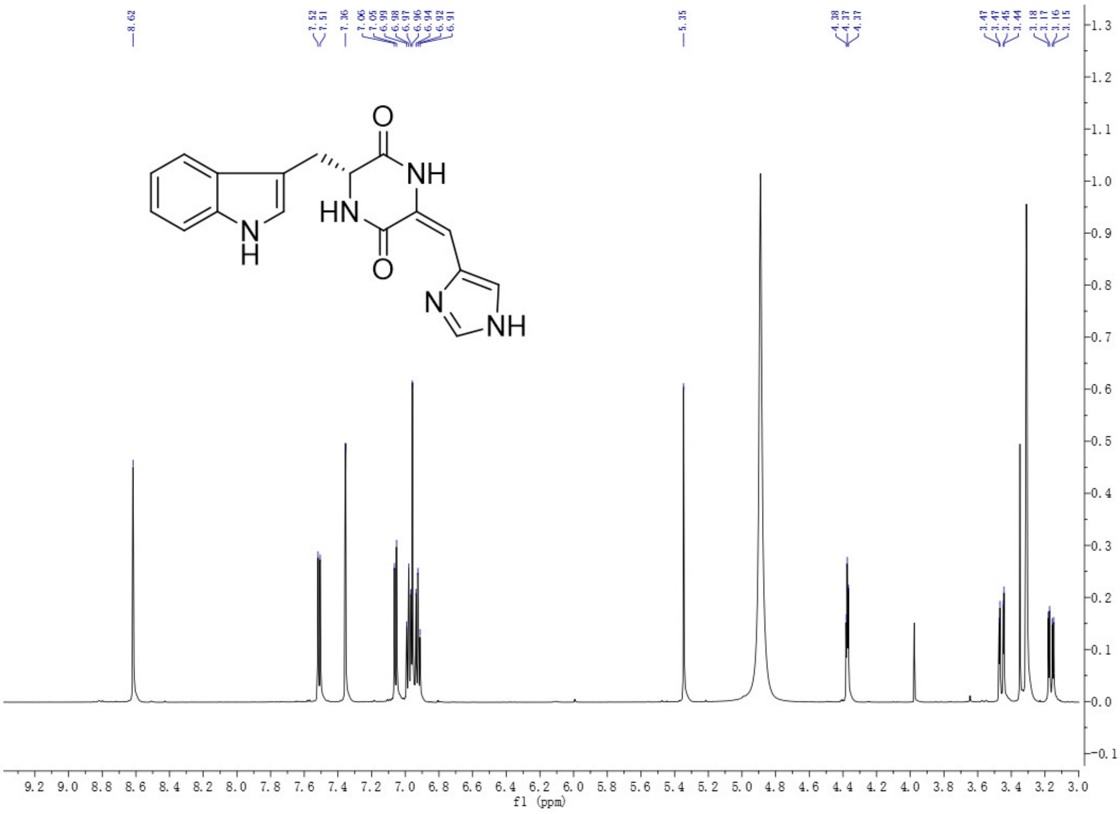

Figure S79. ^{1}H NMR spectrum of penilloid C (**3**, in MeOH-d_4)

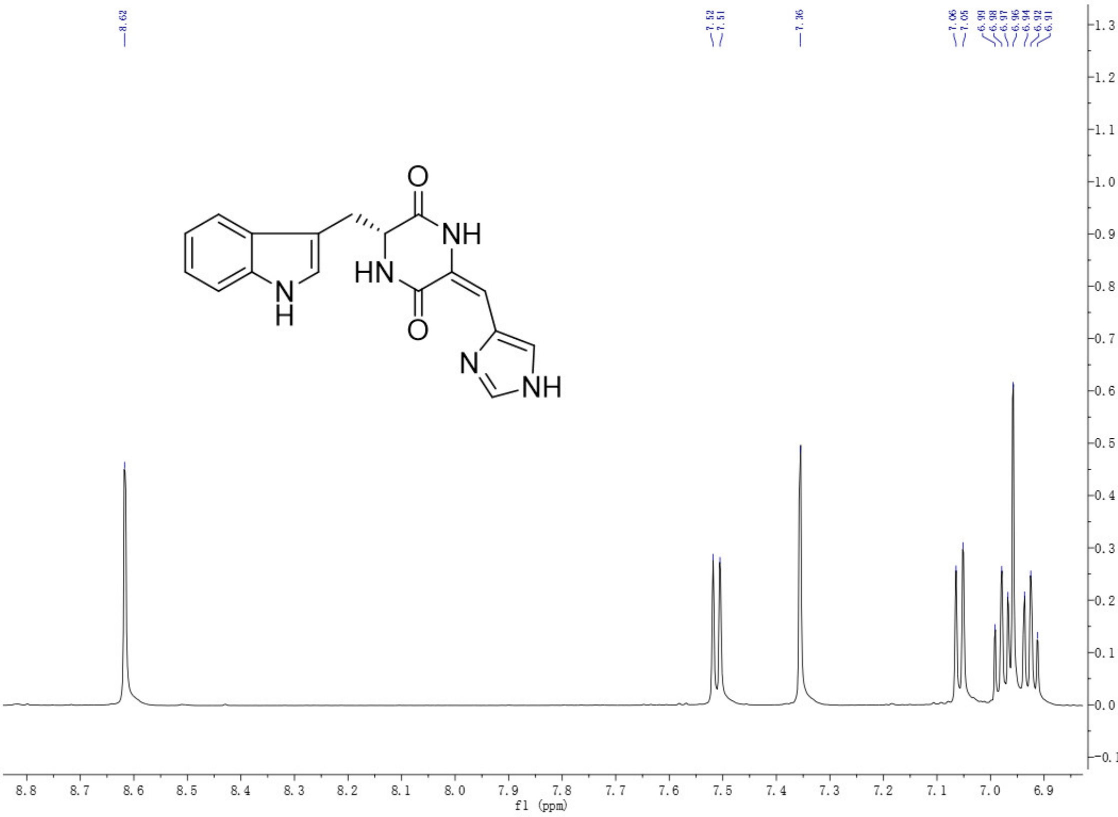

Figure S80. ^{1}H NMR spectrum of penilloid C (**3**, in MeOH-d_4)

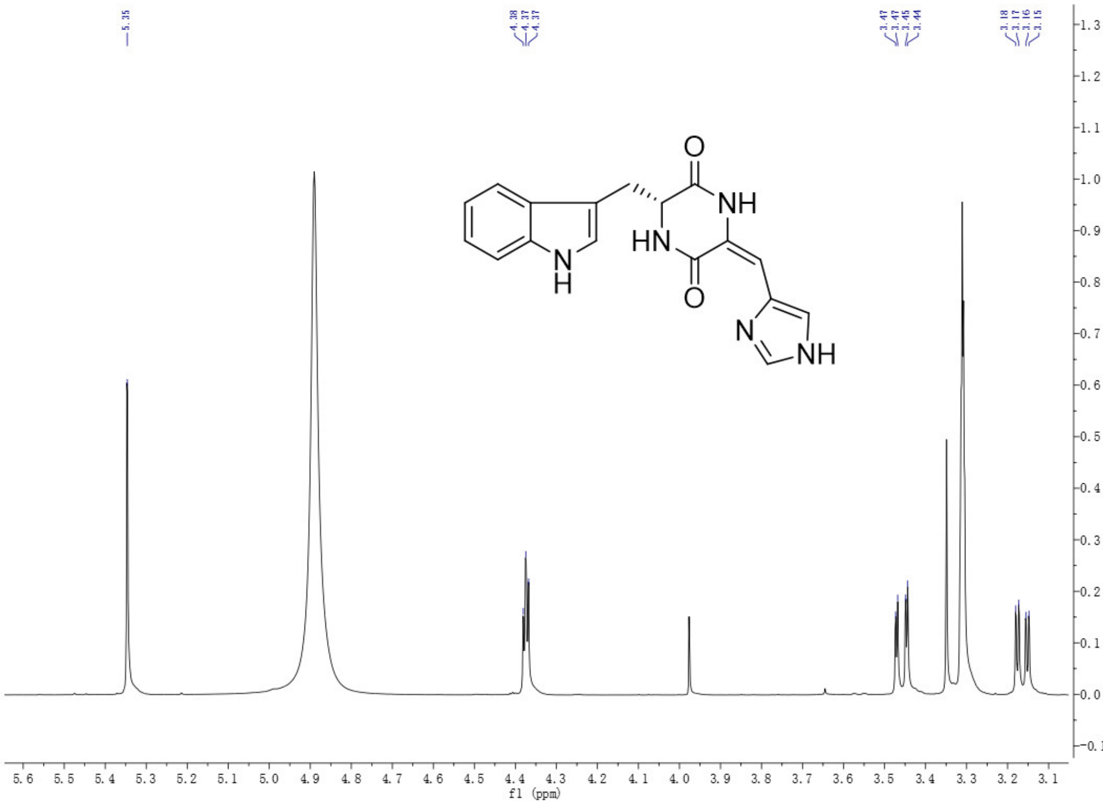

Figure S81. ^{13}C NMR spectrum of penilloid C (**3**, in MeOH-d_4)

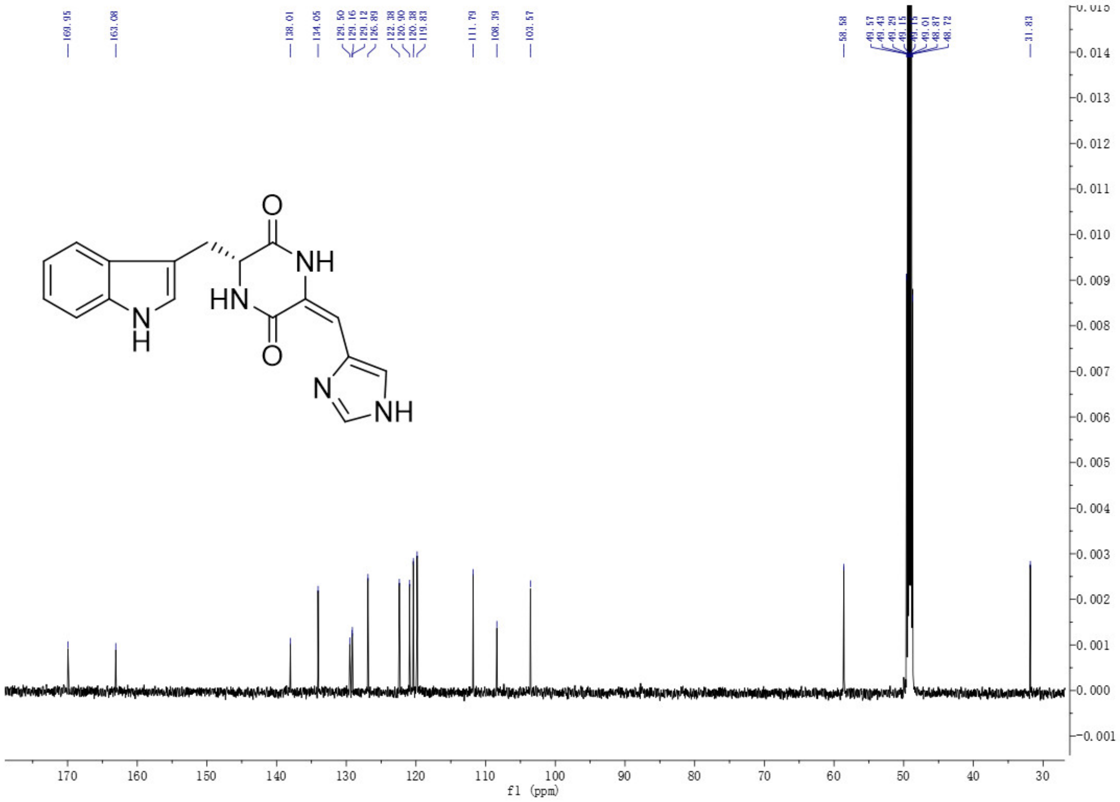

Figure S82. ^{13}C NMR spectrum of penilloid C (**3**, in MeOH-d_4)

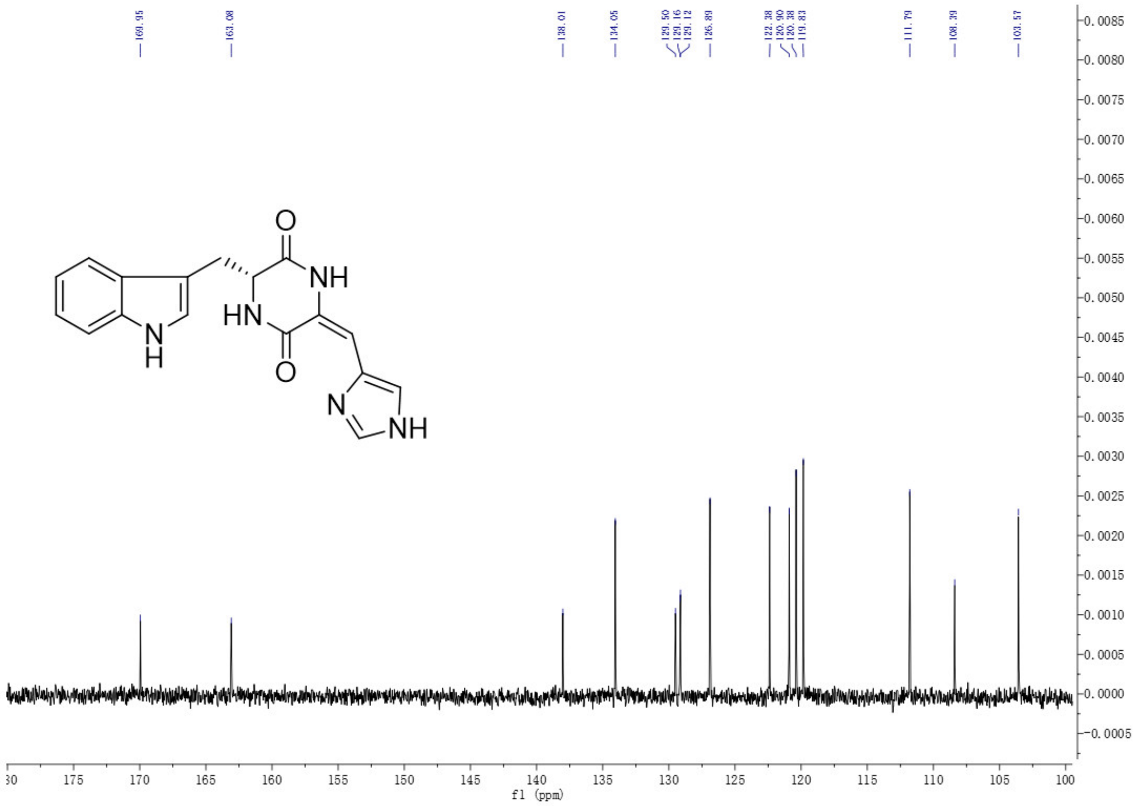

Figure S83. HRESIMS spectrum of penilloid C (**3**)

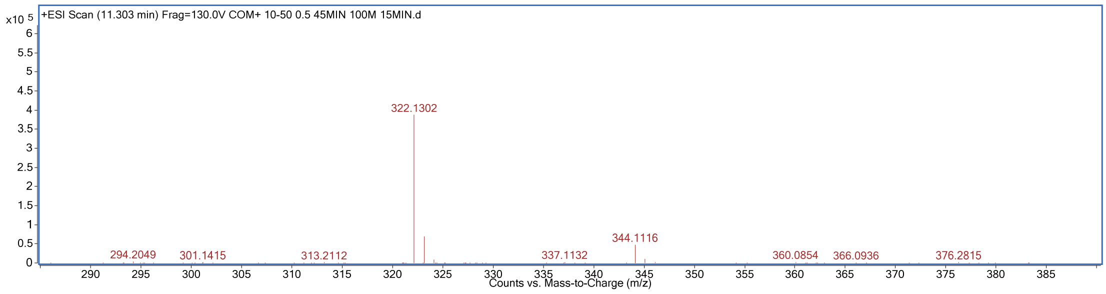

$[M+H]^+$: *m/z* 322.1302 (calcd for $C_{17}H_{16}N_5O_2^+$, 322.1299); $[M+Na]^+$ *m/z* 344.1116 (calcd for $C_{17}H_{15}N_5NaO_2^+$, 344.1118).
